# Quantifying Ultra-Low Pigment Solubilities by UV–Vis Spectroscopy: Implications for Toxicological Assessment

**DOI:** 10.1021/acsmeasuresciau.5c00214

**Published:** 2026-06-18

**Authors:** Lukas J. Patalag

**Affiliations:** Department Chemical and Product Safety, 27652 German Federal Institute for Risk Assessment (BfR), Max-Dohrn-Str. 8-10, Berlin 10589, Germany

**Keywords:** solubility, pigments, UV−vis spectroscopy, excitonic coupling, pp-LFER, bioavailability, tattoo

## Abstract

Accurate toxicological risk assessment of organic pigments is often hampered by their extremely low and poorly characterized solubilities, which obscure their true molecular bioavailability in biological systems. We present a comprehensive optical spectroscopic study of 18 pigments from 7 different structural classes, determining ultralow solubilities in DMSO and NMP through a novel iterative dilution procedure that deconvolutes particulate and molecular spectral signatures based on excitonic coupling theory. Rigorous exclusion of particulate contributions yielded true molecular extinction coefficients and optical band shapes. Results were consolidated by computational simulations of the molecular and crystalline pigment forms, respectively, at the TDDFT (PBE0/def2-SVP) level of theory. To translate these findings to biology, experimentally derived solubilities were extended to water and cytosol by integrating Abraham-type solvation models with composition-based pp-LFER frameworks. This combined approach revealed subnanomolar to subpicomolar solubilities in aqueous media and upper-bound cytosolic concentrations typically below 1 μM, providing a physicochemical basis to critically assess the relevance of toxicological end points, such as sensitization and genotoxicity, for tattooing and patch testing. Our results suggest that many negative toxicological findings may reflect insufficient bioavailability rather than a lack of intrinsic hazard.

## Graphical abstract

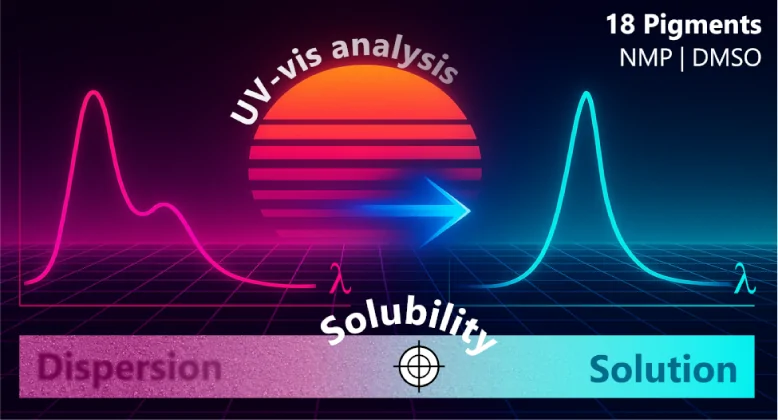

## Introduction

Color has been used by humans for a variety of aesthetic and communicative purposes throughout history and continues to play an essential role in our everyday lives.^1^ Color is employed to capture consumer attention in marketing and carries symbolic meanings, expressing social status, power, and the full spectrum of human emotions. The Industrial Revolution at the turn of the 19th and 20th centuries, along with scientific progress, enabled chemists to uncover the molecular origins of color and to derive colorants not from nature, but from synthetic laboratories and, ultimately, industrial processes.^2^ After Adolf von Baeyer revealed synthetic methods for producing indigo^3^ (now known as Pigment Blue 66) in 1865, one achievement that earned him the Nobel Prize in 1905, many more colorants were discovered. Their coloristic properties were increasingly tailored through molecular fine-tuning to cover the full range of hues. Some of today’s largest chemical companies, such as *BASF* and *Bayer*, grew and gained prominence through their work in colorant chemistry. Although many textile fabrics are colored using reactive dyes that form covalent bonds with the matrix,^4^ synthetic materials, paints, and printing inks typically obtain their color through a color-giving particulate additive known as pigment.^5^ The defining characteristic of a pigment is its near insolubility in the respective application medium or solvent, which results in the dispersion of pigment micro- or nanoparticles within the product. The general analytics of pigments^6^ such as structure identification, determination of purities,^7^ stabilities,^8^ solubilities and finally toxicities^9^ proved to be extremely challenging due to the incompatibility of these substances with most media.^10^ Accurate solubility data are critical for analytical identification, quantification and toxicological risk assessment of pigments. Current evaluations often rely on structural alerts without considering whether bioavailable concentrations reach toxicologically relevant thresholds. This leads to two problems: overestimation of risk based solely on structural information (e.g., azo group), or misinterpretation of negative assay results as proof of safety when bioavailability is simply insufficient. Resolving this challenge necessitates quantifying solubility in both organic and biological media, a task that remains analytically demanding for substances with extremely low solubilities.

Traditional procedures for determining solubilities rely on saturated solutions prepared with excess of analyte followed by gravimetric quantification after solvent removal (e.g., OECD Test Guideline 105, EU Method A.6 (REACH)).^11^ While regulatory frameworks focus on aqueous media, analogous equilibrium methods are applied to organic solvents. For pigments with extremely low solubility, three critical issues emerge: First, nanoparticles must be excluded from the measurement, yet centrifugation and filtration lose efficacy at lower-nanometer sizes. Second, contaminants, often far more soluble than the analyte, can dominate the dissolved fraction and distort the result. This is likely if no substance-specific identification precedes gravimetry or if the analyte cannot be verified because spectral or analytical data are scarce, as is common for pigments. And finally, suitable solvents (e.g., NMP, DMSO) usually have high boiling points (ca. 200 °C), rendering solvent removal incomplete and time-consuming.

The virtual insolubility of pigments and their existence as supramolecular aggregates are key not only to the persistence of a paint film or a tattoo pigment against clearance, but also to intrinsic color properties^12^ and (photo)­stability.^13^ Although individual pigment molecules within a crystal are not covalently linked and largely retain their π-electronic integrity, their close packing allows electronic excited states to interact via different coupling processes.^14^ These interactions, quantified by the coupling energy *J* profoundly modify the optical response of the material,^15^ producing new collective states that alter the UV–vis spectrum of the isolated molecule.^16^ A dominant interaction, known as excitonic coupling, is treated within the exciton framework originating with Frenkel (1931)^17,18^ and later formalized for molecular aggregates by Kasha (1959).^19^ It accounts for the characteristic H-type (blue-shifted) and J-type (red-shifted) spectral shifts originally observed by Jelley^20^ and Scheibe (1936/37).^21^ In many crystal structures, when more than one symmetry-inequivalent molecule resides in the unit cell, an additional fine structure (Davydov splitting) appears.^22^ Excitonic coupling can be further accompanied by charge-transfer (CT) contributions, giving rise to exciton-CT mixing.^23^ Figure 1 illustrates spectral shifts in a simple dimer model within a simplified excitonic framework.^24^ The magnitude of the shift Δ*E* depends on the amount of unit cells (or particle size) rapidly approaching the limiting value 2*J* according to $\Delta E=2J\cos\left(\frac{\pi }{N+1}\right)$ with *N* being the number of coupling units or interacting chromophores. Since even the smallest nanoparticles (10 nm in size) already consist of hundreds of pigment molecules the spectral shift appears widely constant and independent of particle size. For deeper insights into exciton theory, the reader is referred to the literature.^19,25^

**Figure 1:**
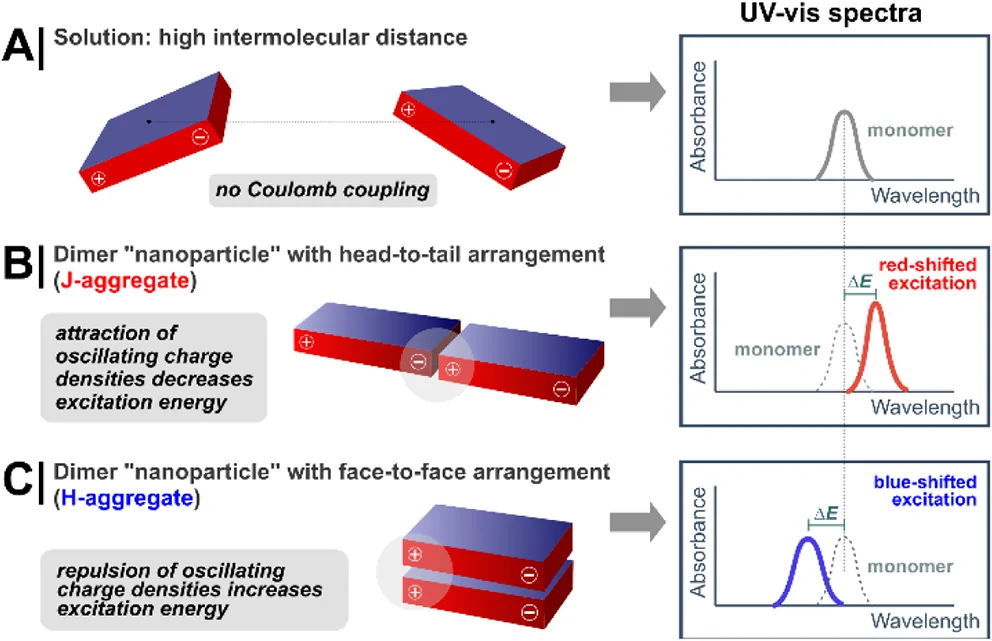
Simplified illustration of excitonic coupling (dimer model) and its effect on UV–vis spectra, restricted to the Coulomb coupling component. Pigment molecules are represented by cuboids. Shading and charges (±) depict a snapshot of the oscillating transition dipoles after excitation (cases B and C). A) shows the monomeric spectrum when molecules are far apart with negligible Coulomb coupling, as in dilute solutions. Due to the coplanar arrangement of the molecules, no spectral Davydov components are visible.

In this work we used the spectral response of exciton coupling to determine the transition point from dispersion to solution, accurately reflecting the solubility of a given substance. Crucially, progressive dilution eventually eliminates all H-/J-shifted bands until the spectrum collapses to the monomer reference, reflecting the absence of particulate aggregates. This transition provides a sensitive spectroscopic handle to determine the solubility limit. Only below this threshold is the sample truly homogeneous and thus compatible with the underlying assumptions of Lambert–Beer’s law, operationally indicated by spectral invariance under further dilution. Importantly, the solubility so determined reflects the thermodynamic equilibrium of the solvent-specific crystal phase at the transition point, independent of which polymorph was initially introduced, since any metastable phase will have converted to the equilibrium form at the small particle sizes and high surface-to-volume ratios encountered near this limit. In cases where solubility was exceptionally high and the extinction signal approached the limits of spectroscopic linearity, the scattering background at longer wavelengths and the complete decline of the excitonic band were used as an alternative though less sensitive indicator of the transition point. In parallel, we also performed DLS measurements across the dilution series, without obtaining reproducible or internally consistent results. We attribute these difficulties to the low refractive-index contrast in NMP/DMSO and residual absorption at the laser wavelength, which suppress scattering, distort autocorrelations, and let sporadic rare aggregates spuriously mimic particle presence (see Section 5 of the Supporting Information). To interpret dispersion spectra and assess the relative positions of absorption bands, we carried out TDDFT simulations both for isolated pigment molecules and for finite crystalline ensembles. Crystalline clusters (4–12 molecules, <500 atoms) were extracted from CCDC crystal structures, prioritizing π-π stacking and H-bonding interactions. Full computational details (specific cluster sizes and superstructures) are provided in the Supporting Information. Owing to the electronic complexity of such polymolecular systems, this level of theory can only provide the relative energy shift between the molecular and crystalline states (red- or blue-shift). This computational analysis draws on the pioneering contributions of M. U. Schmidt and J. Mizuguchi to crystal structure elucidation. Across all pigment classes investigated, crystal packing and hence solubility are governed by two principal types of intermolecular interactions: van der Waals forces (dipolar, dipolar-induction and London dispersion contributions, the latter scaling with molecular weight and contact area)^26,27^ and, where NH or CO groups are available, hydrogen bonding. Finally, it needs to be stated that the detected extinctions in this work, particularly in dispersion samples, go beyond simple absorption and arise from a complex interplay of various optoelectronic phenomena,^28^ some of which challenge the validity of Lambert–Beer’s law.^29^ In particular, local field effects described in the simplest case by the Lorentz–Lorenz relation^30^ can lead to spectral shifts both in energy and intensity, in addition to excitonic effects. Within particles, such corrections are an intrinsic property of the condensed phase that converges rapidly with particle size and is inseparable from the excitonic response captured by our TDDFT treatment. Between particles and among dissolved molecules, these effects scale with the pigment volume fraction and solute concentration, respectively, both of which remain orders of magnitude below the thresholds established for measurable deviations from Beer-type behavior. We have accordingly not found strong indications that these contributions play a major and decisive role, which will be taken up later in specific cases.

## Experimental Section

Pigments were obtained as fine chemicals of the highest purity available and are referred to throughout this work by their Color Index (C.I.) generic names, abbreviated according to the standard convention: PY = Pigment Yellow, PO = Pigment Orange, PR = Pigment Red, PV = Pigment Violet, PB = Pigment Blue, and PG = Pigment Green. The numerical suffix denotes the individual pigment within each color class (e.g., PR266 = Pigment Red 266). The pigments investigated in this study, identified by their Color Index (C.I.) generic name, C.I. constitution number, and CAS registry number, were: PR22 (C.I. 12315, CAS 6448-95-9), PR266 (C.I. 12474, CAS 36968-27-1), PR170 (C.I. 12475, CAS 2786-76-7), PY151 (C.I. 13980, CAS 31837-42-0), PO13 (C.I. 21110, CAS 3520-72-7), PY14 (C.I. 21095, CAS 5468-75-7), PY83 (C.I. 21108, CAS 5567-15-7), PV19 (C.I. 73900, CAS 1047-16-1), PR122 (C.I. 73915, CAS 980-26-7), PR202 (C.I. 73907, CAS 3089-17-6), PR255 (C.I. 561050, CAS 54660-00-3), PR254 (C.I. 56110, CAS 84632-65-5), PR264 (C.I. 561300, CAS 88949-33-1), PO73 (C.I. 561170, CAS 84632-59-7), PB60 (C.I. 69800, CAS 81-77-6), PY138 (C.I. 56300, CAS 30125-47-4), PB15 (C.I. 74160, CAS 147-14-8), and PG7 (C.I. 74260, CAS 1328-53-6). Reported purities often deviate and cannot be routinely verified, but according to ECHA registration data ranged from 90% to 98% for the samples used in this study. In some cases, slow degradation processes were observed and will be reported elsewhere. *N*-methylpyrrolidone (NMP, Roth, ≥99.8%) and dimethyl sulfoxide (DMSO, Honeywell, ≥99.9%) were employed as solvents and served as blanks in all spectroscopic measurements. Depending on the expected solubility, quartz cuvettes of 0.1, 0.5, 1.0, or 2.0 cm path length (Hellma QS, UV/vis (200–2500 nm)) were used to maintain instrumental linearity. Pigment stock suspensions were freshly prepared at 1 mg/mL, except for **PO73** and **PR255** (10 mg/mL), and dispersed for 5 min by mild ultrasonication at room temperature to minimize degradation while establishing sufficient deagglomeration. Stocks were stored in dark vials and diluted stepwise (1:10) down to 100 ng/mL, yielding five principal dilutions. Each of these was further diluted by factors of 0.5 and 0.2, resulting in 16 samples that spanned a broad solubility range. For accurate determination of the transition point, two additional pigment-specific dilution series with finer calibration (typically 10–12 samples) were prepared. Final molecular UV–vis spectra, used to derive extinction coefficients *ε*, were extracted from concentrations approximately 20% below the transition point to ensure the absence of particle contributions. Before spectral recording, all samples were equilibrated in the dark for 30 min at room temperature (293 ± 1 K). Spectra were acquired on a Shimadzu UV-1800 double-beam spectrometer (190–900 nm, 1 nm bandwidth, ±0.1 nm wavelength accuracy); sample heating during the short single scans (<2 min) was negligible, keeping the temperature within ±1 K of 293 K. The overall uncertainty of the solubility values combines random and systematic contributions. The random component was derived from the reproducibility of independent replicate series (at least three technical replicates per pigment, more for ultralow-solubility species such as PB15 and PB60), with deviations consistently within ±10%. It encompasses typical sources of random error, such as photometric noise at low absorbance, baseline drift, weighing and pipetting errors (including cumulative variance across iterative dilution steps), solvent-blank variability, cuvette positioning, and short-term temperature fluctuations within the climate-controlled laboratory environment (293 ± 1 K). Systematic contributions instead affect the absolute value and arise primarily from the undeclared pigment purity (90–98% per ECHA registration data) and potential degradation processes during stock preparation for some pigments. Individually difficult to quantify, they are bounded by the reported purity range and by the consistency of our data across NMP and DMSO. Combining both contributions, we estimate the overall uncertainty at ±20% for solubilities and ±10% for extinction coefficients, in line with standard literature values.

## Results and Discussion

### Experimental Solubility Determination

#### Monohydrazone Pigment Class

Pigments containing a single azo group are the most common representatives of the azo family.^5,31^ Their color range typically extends from yellow to red, with red species usually incorporating a central naphthol unit that enlarges the π-system and causes the red shift. Because this naphthol moiety is often substituted with a peripheral aromatic amide, the designation *Naphthol AS* (AS: *Amid der Säure* - amide of the acid) was introduced early on.^32^ The *Naphthol AS* unit, capable of accommodating different amide substituents,^33^ forms the essential building block for azo coupling. In this study we selected three widely used *Naphthol AS*-based pigments, **PR170**, **PR266** and **PR22** (Figure 2). **PR266** is a close analogue of **PR170** in which the ethoxy function is replaced by a methoxy group at the aromatic amide. As a non-*Naphthol AS* example, we included **PY151**, which carries an unusual carboxylic group that appears at first sight ill-suited for water-insoluble pigments. Here, the naphthol unit is formally replaced by an acetyl group that, in its enol form, is cross-conjugated with the azo and amide moieties.^34^

**Figure 2:**
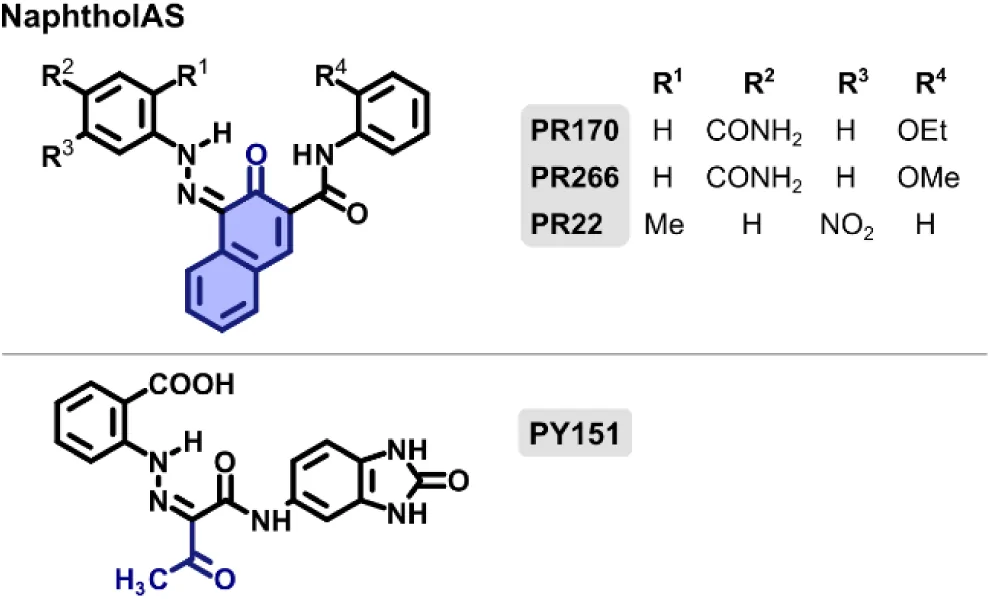
Chemical derivatizations of monohydrazone pigments investigated in this study. The moiety highlighted in blue represents the central bridging unit connecting the azo group with the amide peripheries (naphthol in the case of *Naphthol AS*; acetyl in **PY151**). Crystal structure analysis, together with computational results, indicates that the hydrazone tautomer shown is energetically favored over the azo form.

The UV–vis spectra of the *Naphthol AS* pigments display a characteristic double peak at ca. 500–550 nm (Figure 3), with **PR22** (Figures S11–S13) slightly blue-shifted and **PR170** (Figures S3–S5) and **PR266** virtually overlapping due to their structural similarity. By contrast, **PY151** shows a single absorption band near 400 nm without marked vibronic progression (Figure S18). This may result from the abundance of polar functional groups in **PY151** that interact strongly with the solvent environment, leading to broadened and indistinct excitation energies. While the *Naphthol AS* species exhibit a red-shifted excitonic shoulder superimposed with scattering, **PY151** lacks a clear excitonic fingerprint in its spectra (Figures S16–S17). Except for **PR170**, all three pigments dissolve remarkably well in NMP, particularly **PY151**. As a result, the dispersion-solution transition point could not be identified within the limits of spectroscopic linearity (extinction >1 even with 0.1 cm cuvettes), and the scattering signal had to be used instead.

**Figure 3:**
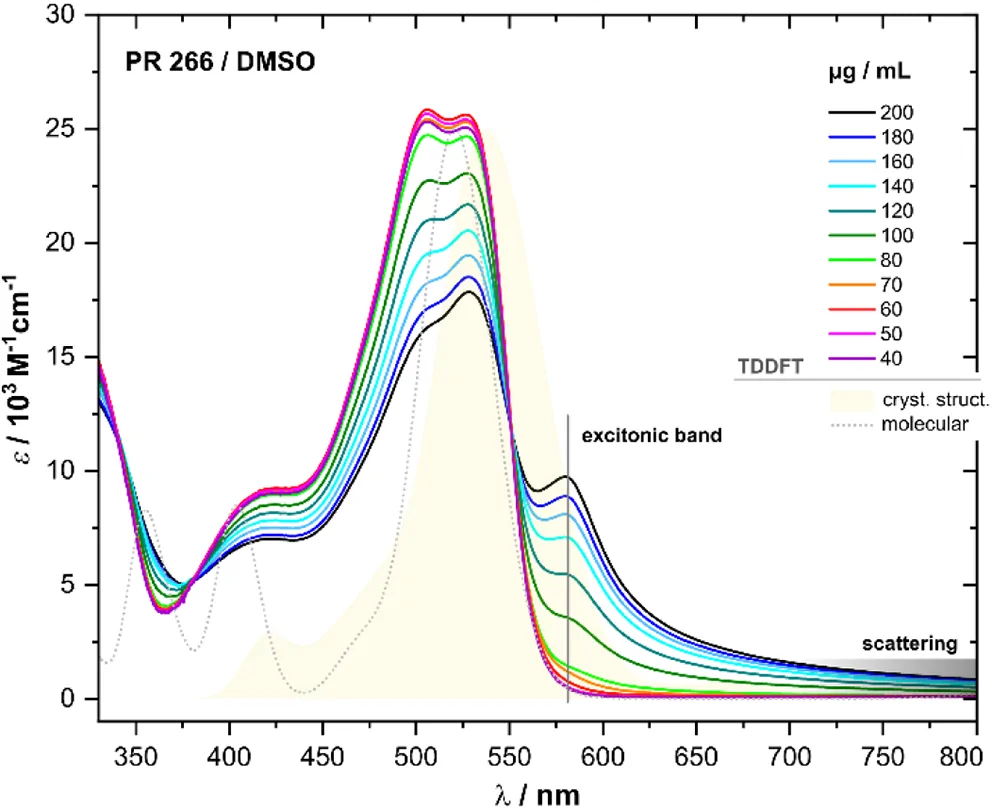
Iterative dilution of **PR266** in DMSO. The sequence begins with a dispersion (200 μg/mL) and follows the UV–vis spectral changes down to 40 μg/mL. The energy of the declining band (ca. 580 nm) remains constant, effectively ruling out local-field effects. Filled area represents the normalized TDDFT spectrum of a literature-reported crystal structure (Figure S7); the monomer simulation (dotted line) is referenced to the experimental main absorption peak.

In DMSO, however, the solubility of all *Naphthol AS* pigments was low enough to monitor the disappearance of the excitonic feature and determine the solubility precisely. In all three cases an isosbestic point appeared during dilution, suggesting the absence of newly formed, solvent-specific crystal structures and pointing to a common particle UV–vis signature independent of size and identity. Figure 3 illustrates the iterative dilution procedure of **PR266** in DMSO (for spectrum in NMP see Figure S8). At 200 μg/mL the spectrum consists of a main band at 500–550 nm accompanied by a red-shifted shoulder at about 580 nm. Upon dilution the main band grows while the shoulder vanishes below ca. 60 μg/mL, consistent with an excitonic contribution from residual particles. In line with the Lambert–Beer law, further dilution no longer increases the extinction coefficient but only reveals photometric noise (slight decrease in *ε*). The solubility can thus be estimated at ca. *S*_DMSO_ = 50 μg/mL. This example also highlights the superior signal-to-noise ratio of absorption compared to scattering: whereas the scattering signal at 80 μg/mL has essentially vanished, the additional extinction at 580 nm (excitonic shoulder) still indicates a remaining particle load. The TDDFT simulation reproduces the red-shift of the excitonic band relative to the monomer, confirming its particulate origin (see also Figure S10). Consistent with its slightly increased molecular weight, the solubility of **PR170** is lower (*S*_DMSO_ = 21 μg/mL) in both solvents. In contrast, **PY151** exhibits one of the highest solubilities in this study (*S*_DMSO_ = 720 μg/mL) and is the only pigment, for which *S*_DMSO_ exceeds *S*_NMP_. This can be attributed to its exceptionally high polarity, which is better stabilized in the more polar DMSO than in NMP. All solubilities are compiled in Table 1.

**Table 1:** Experimental Values Obtained in the UV–Vis Spectroscopic Analysis

Pigment		NMP				DMSO			
	MW^a^ (μg/μmol)	*S* ^b^ (μg/mL)	*S* ^c^ (μM)	λ_ma*x*_ ^d^ (nm)	*ε*_max_ ^e^ (10^3^ M^–1^ cm^–1^)	*S* ^d^ (μg/mL)	*S* ^c^ (μM)	λ_ma*x*_ ^d^ (nm)	*ε*_max_ ^e^ (10^3^ M^–1^ cm^–1^)
**PR22**	426.42	550	1290	501	19	40	94	500	19
**PR266** ^HB^	440.45	340	770	506; 528^f^	25	50	114	506; 526^f^	26
**PR170** ^HB^	454.48	140	308	506; 529^f^	26	21	46	507; 527^f^	26
**PY151** ^HB^	381.34	550	1440	380	23	720	1900	375	23
**PO13**	623.49	0.5	0.8	521	74	<0.03^g^	<0.05^g^	-	-
**PY14**	657.55	1.5	2.3	429	60	<0.1^g^	<0.15^g^	-	-
**PY83**	818.5	0.22	0.27	432	53	<0.02^g^	<0.024^g^	-	-
**PV19** ^HB^	312.32	270	860	519	13	80	260	523	13
**PR122** ^HB^	340.37	18	53	528	13	4.2	12.3	530	13
**PR202** ^HB^	381.21	2.2	5.8	526	13	0.3	0.8	530	11
**PR255** ^HB^	288.31	1800	6240	506	31	1050	3640	506	33
**PR254** ^HB^	357.19	17	48	516	33	5.7	16	517	35
**PR264** ^HB^	440.49	1.7	3.9	534	46	0.45	1.00	534	49
**PO73** ^HB^	400.51	4000	10000	511	37	700	1750	511	36
**PB60**	442.42	0.18	0.41	741	25	<0.01^g^	<0.023^g^	-	-
**PY138**	693.96	6	8.6	427	29	<0.05^g^	<0.070^g^	-	-
**PB15**	576.07	0.07	0.12	671	210	<0.01^g^	<0.017^g^	-	-
**PG7**	1127.18	<0.01^g^	<0.009^g^	-	-	<0.01^g^	<0.009^g^	-	-

a Molecular weight.

b Experimental solubility derived from UV–vis analysis.

c Experimental solubility derived from UV–vis analysis as molar concentration using MW.

d Wavelength of main, most red-shifted molecular absorption band.

e Extinction coefficient of main, most red-shifted molecular absorption band.

f Two main absorption peaks of similar dominance.

g Solubility could only be determined as upper bound due to instrumental limitations resulting in indefinable λ_ma*x*_ and *ε*_max_ values. A blue index *HB* indicates the presence of intermolecular hydrogen bonds in at least one published crystal structure. No crystal structure has been reported for **PR22** to date.

#### Dihydrazone Pigment Class

Dihydrazone pigments,^35^ here represented by diarylide congeners, are the most important organic yellow pigments with the highest annual production volume worldwide.^36^ Their application is largely focused on four-color printing resulting in a dominant appearance in magazines, newspapers and up to recently also tattooing.^37^ Many congeners incorporate 3,3′-dichlorobenzidine as the central building block (classified as a category 1B carcinogen under the EU CLP Regulation (EC) No. 1272/2008 on Classification, Labelling and Packaging of substances and mixtures), which raises toxicological^38–41^ and environmental^42^ concerns regarding their widespread use in close-contact consumer products. From a chemical point of view, the dihydrazone structure can be regarded as a dimer of two monohydrazone species, *para*-coupled via their hydrazone-bearing phenyl groups, yielding a biphenyl (benzidine) coupling motif (Figure 4). In solution, this motif is conformationally flexible with an energy minimum at a torsion angle of about 44°,^43,44^ which limits π-conjugation across the two phenyl rings and contributes to the comparatively high absorption energy (similar to **PY151**) and the resulting yellow hue, despite the otherwise extended π-system. In the crystal, by contrast, packing forces enforce a planar biphenyl geometry in both **PY14** and **PY83**, restoring full conjugation and likely contributing to the high molar extinction coefficients observed in the solid state.

**Figure 4:**
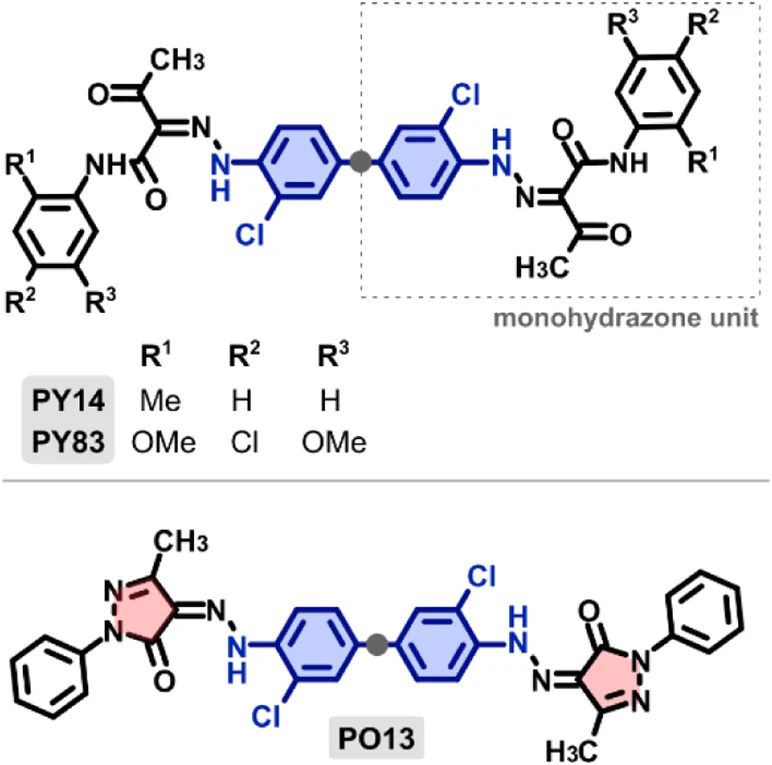
Chemical derivatizations of the dihydrazone pigments used in this study. The moiety highlighted in blue is 3,3′-dichlorobenzidine as the central bridging unit, pyrazolone is shown in red. The rectangle depicts the monohydrazone unit, from which the dihydrazone pigment class can be derived via homodimerization. The gray dot marks the π-conjugation node (dihedral angle ca. 44°).

In this study, we included two decorated representatives of the dihydrazone class, **PY14** and **PY83**, which differ in the polarity of their substitution patterns. In order to reveal the effect of additional aryl moieties, increased planarity in solution (aromaticity) and less degrees of rotational freedom (pyrazolone units), we added **PO13** as another commercially important congener. The solubilities of the dihydrazone species are disproportionately reduced in contrast to their monohydrazone counterparts.

While the molecular weight ratio is ca. 1.5 (dihydrazone/monohydrazone) the solubilities of the dihydrazone series are strongly reduced by a factor of ca. 500 on average (NMP). In fact, the solubilities of all three species in DMSO were too low to be resolved by our instrumental signal-to-noise ratio and could only be estimated by an upper bound. NMP proved to be the superior solvent for all pigments in our study (except **PY151**) and delivered a comprehensive data set. As expected, **PY83** with the highest molecular weight (MW = 819) showed the lowest solubility of the dihydrazone series (*S*_NMP_ = 0.22 μg/mL, Figures S29–S33), indicating that the additional, local polarity introduced by four methoxy groups enhances intermolecular van der Waals interactions more strongly than it improves solvent–solute stabilization in the NMP environment. The two additional chlorine substituents further reinforce this trend through their dispersion contribution to intermolecular van der Waals interactions. Interestingly, despite the lower molecular weight of **PO13** (MW = 623) compared to **PY14** (MW = 658), the solubility of **PO13** is significantly lower (*S*_NMP_ = 0.5 μg/mL) compared to **PY14** (*S*_NMP_ = 1.5 μg/mL). This arises from the rigidified **PO13** backbone imposed by its two pyrazolone units, which enhance intermolecular π-π stacking and associated dispersion interactions. The extinction coefficients *ε* of the dihydrazone series exceed the expected 2-fold value relative to comparable monohydrazone species (e.g., **PY151**), reaching ε = 53,000–74,000 M^–1^ cm^–1^, which we attribute to efficient intramolecular excitonic coupling between the monohydrazone subunits. **PO13** exhibits the highest value (ε = 74,000 M^–1^ cm^–1^), benefiting from enhanced π-conjugation within its pyrazolone units. Figure 5 exemplarily depicts the UV–vis spectral evolution of a **PO13** sample in NMP during the dilution process. The scattering signal intensity is extremely low in NMP and would not have allowed an accurate solubility analysis. It proves, however, the dominance of particularly small nanoparticles which might allow a quick equilibration of various crystal structures in the dispersion prior to the dilution process. This assumption is supported by the unique observation in our study that the dispersion UV–vis signatures differ strongly in DMSO and NMP (Figure S23). Another peculiarity of **PO13** is the blue-shifted UV–vis band of the dispersion relative to the solution in NMP. In contrast to the previously discussed pigments, no isosbestic points were observed, supporting the spectral contribution of more than two species. In fact, for **PO13** a broad crystal polymorphism is literature-known.^45,46^ While the α phase (parallel packing) is the outcome of the industrial synthetic process, the β phase (herringbone packing) is thermodynamically most stable. TDDFT calculations predict opposing spectral shifts: the α-phase exhibits a red-shift of the absorption maximum relative to the simulated monomer, while the β-phase shows a corresponding blue-shift. Our experimental spectra match the β-phase prediction. Within the limitations of our TDDFT approach for such complex polymolecular systems, these observations are consistent with, but do not prove, either: (1) β-phase dominance in the commercial sample used, or (2) rapid, solvent-mediated α → β equilibration during dispersion preparation and dilution. Crucially, these phase-related uncertainties do not compromise the solubility determination, which relies solely on the disappearance of aggregate spectral features at the transition point, regardless of which phase(s) are present.

**Figure 5:**
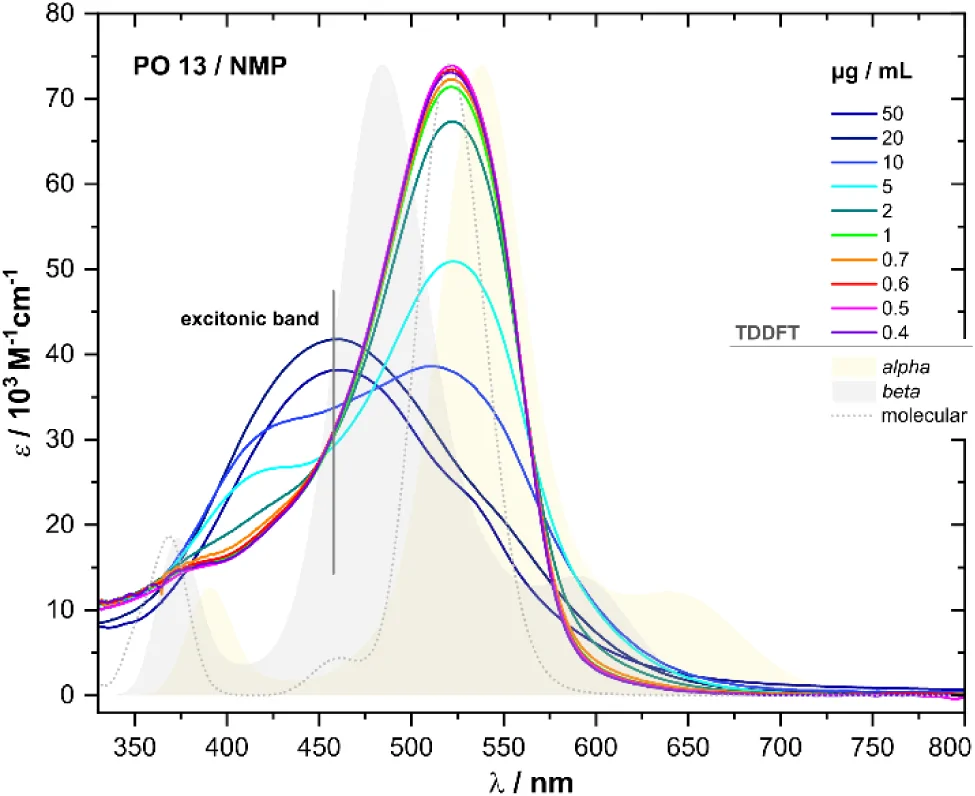
Iterative dilution procedure of **PO13** in NMP. The measurement sequence starts with a dispersion (50 μg/mL) and tracks the UV–vis spectral evolution down to 0.4 μg/mL. Filled areas represent normalized TDDFT spectra of literature-reported crystal structures (α and β phase, Figure S20); the monomer simulation (dotted line) is referenced to the experimental main absorption peak.

#### Quinacridone Pigment Class

The name quinacridone was first introduced by Niementowski in 1896 for compounds bearing two benzannulated 4-(1*H*)-pyridinone units.^47,48^ It took several decades until *DuPon*t started to commercialize quinacridone derivatives as pigments in 1955.^49^ Nowadays some species (**PV19**) belong to the most important pigments for red-violet shades in automotive finishes, powder coatings, paints, plastics and high-grade printing inks. Quinacridones form extensive hydrogen-bond networks in the crystalline state that substantially contribute to packing energy,^50^ and they are intrinsically strong fluorophores at the molecular level.^51,52^ In this study we chose **PV19** as the parent unsubstituted quinacridone prototype and investigated the impact of methyl (**PR122**) and chloride substituents (**PR202**) on solubility (Figure 6).

**Figure 6:**
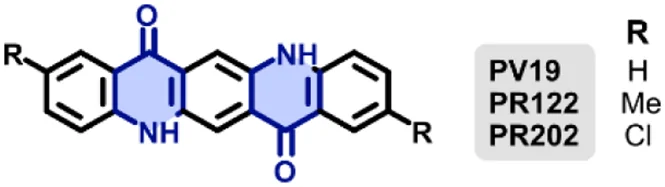
Chemical derivatizations of the quinacridone pigments used in this study. The characteristic 4-(1*H*)-pyridinone moiety is highlighted in blue.

In contrast to the azo pigments discussed before, quinacridones exhibit a higher molecular symmetry (*C*_*2h*_ point group for all three representatives) facilitating dense crystal packing and stronger intermolecular van der Waals interactions. The lack of rotational freedom along the extended π-system leads to a skeletal rigidity which impacts molecular UV–vis spectral signatures: All species studied show a dominant and solitary main absorption band at ca. 500–530 nm with a distinct and well-resolved vibronic progression. In dispersion samples, a pronounced red-shifted excitonic peak at ca. 575 nm is visible as a solid state effect in all three cases, which is the determining origin of the red to violet color spectrum of this pigment class. Compared to monohydrazone pigments, molecular extinction coefficients are consistently lower but of similar magnitude for all three species, not exceeding *ε* = 15,000 M^–1^ cm^–1^. However, despite their lower molecular weight in relation to red pigments of the *Naphthol AS* monohydrazone family from above (factor of ca. 0.8), their solubilities are disproportionally lower which can be attributed to their higher molecular rigidity and symmetry. Figure 7 exemplarily illustrates the spectral outcome of the dilution process for **PR202** in NMP as another example that underlines the superiority of the band shape analysis over simple scattering detection. Although the scattering signal at long wavelength has practically declined to zero already at 4 μg/mL, the excitonic band is still dominant and clearly indicates a remaining particle component. TDDFT simulations could confirm red-shifted absorption bands in the solid-state for two crystal structures (Figure S45). The solubility of **PR202** in NMP was determined to be ca. *S*_NMP_ = 2.2 μg/mL. Interestingly, the methyl substituted congener **PR122** exhibits a solubility ten times higher (ca. *S*_NMP_ = 18 μg/mL), both in DMSO and NMP, which might indicate either a superior dispersion donor quality of the chloride residues or a more compact crystal packing as result of the lower halogen van der Waals radius (Figures S40 and S45). A further, approximately order-of-magnitude increase in solubility (ca. 270 μg/mL in NMP) is observed when the core motif is left unsubstituted (**PV19**, Figures S34–S38). Similar to most dilution sequences in this study, the wavelength of the declining band (ca. 575 nm in this case) remains constant, effectively ruling out local field effects and supporting an excitonic origin.

**Figure 7:**
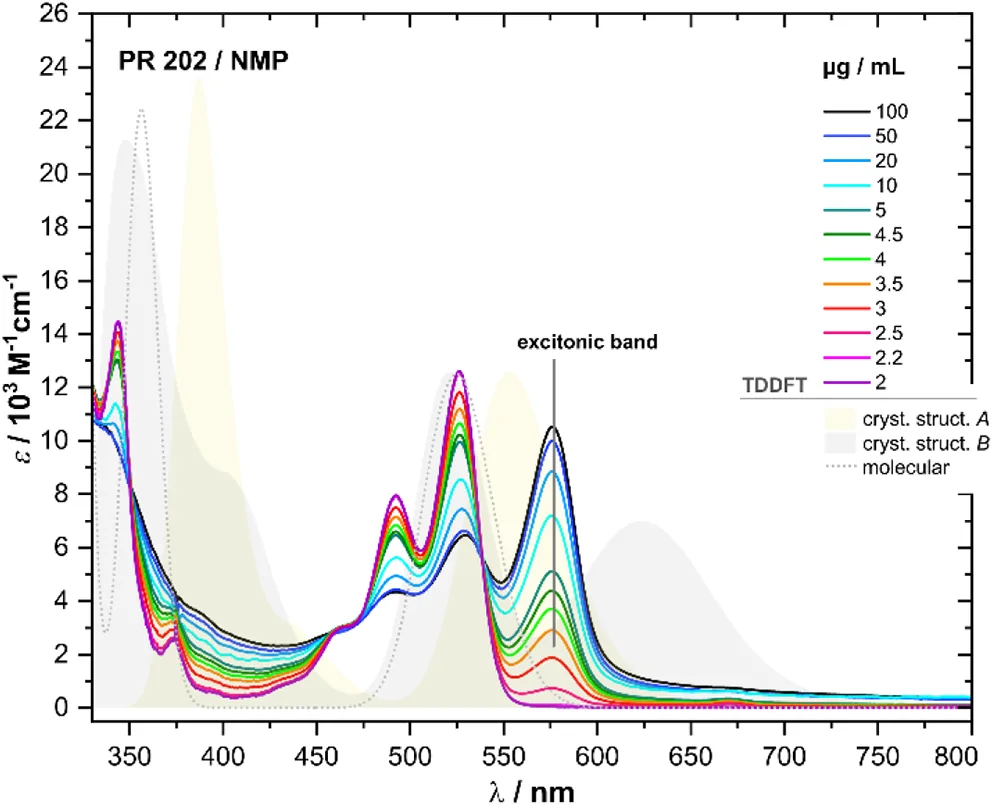
Iterative dilution procedure of **PR202** in NMP. The measurement sequence starts with a dispersion (100 μg/mL) and tracks the UV–vis spectral evolution down to 2 μg/mL. Filled areas represent normalized TDDFT spectra of literature-reported crystal structures (Figure S45); the monomer simulation (dotted line) is referenced to the experimental main absorption peak.

#### Diketopyrrolopyrrole Pigment Class

Diketopyrrolopyrrole (DPP) derived pigments are a modern class of colorants^49^ that were accidentally discovered in academia during a synthetic attempt to azetinones (1974)^53^ and later commercialized by *Ciba-Geigy*. Similar to quinacridones, the DPP skeleton is capable of strong photoluminescence in the molecular,^54^ but also in a sufficiently isolated, aggregated state.^55^ The most prominent DPP representative is the dichlorinated species **PR254** “Ferrari red”. Indeed, DPPs are widely used in applications requiring exceptional lightfastness and durability, such as automotive coatings. Within our pigment scope we started with the fully unsubstituted prototypical DPP **PR255** as reference (Figure 8). Three derivatives with differing substitution patterns were included allowing to compare the impact of chloride residues (**PR254**) vs phenyl rings (**PR264**) on solubility and elucidate the influence of *tert*-butyl groups as strong but bulky dispersion energy donors^56^ (**PO73**).

**Figure 8:**
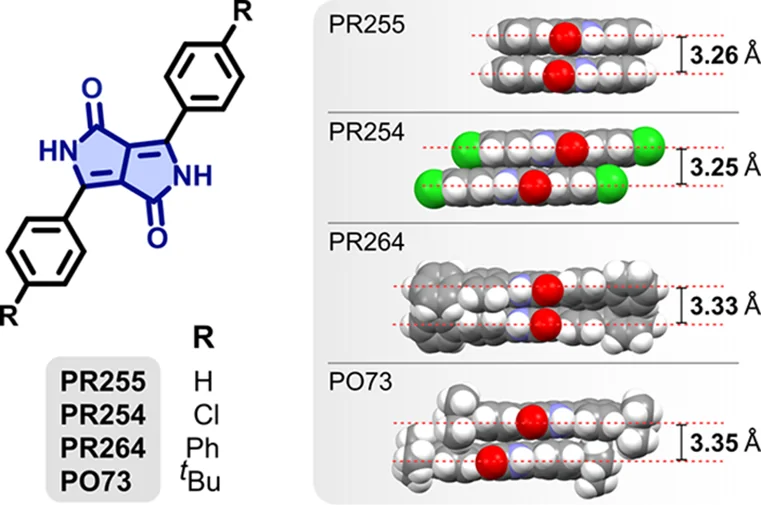
Chemical derivatizations of the DPP pigments used in this study. The characteristic diketopyrrolopyrrole moiety is highlighted in blue. Molecular structures illustrating interplanar distances in published crystal structures were taken as dimer cutouts. The pictorial representation uses atomic van der Waals radii.

DPP pigments are of high molecular symmetry (*C*_*2h*_ point group) and integrate electron-rich carbonyl functions in addition to NH groups of certain acidity. They are consistently more efficient chromophores than quinacridones reaching extinction coefficients above *ε* = 30,000 M^–1^ cm^–1^ based on a very compact chromophore backbone. All DPP species in this study, irrespective of derivatization, form sequential hydrogen bond patterns upon aggregation increasing the stability of the crystal lattice.^57^ Figure 9 illustrates the solubility determination of **PR254** in NMP. The scattering signal is comparatively strong and a dominant red-shifted excitonic band is present in dispersion, which is qualitatively reproduced by TDDFT simulations. The solubility was determined to ca. *S*_NMP_ = 17 μg/mL, a similar value as for **PR122** (quinacridone) which is of comparable molecular weight. Replacing the chloride residues by phenyl groups (**PR264**, Figures S59 and S60) reduces the solubility by 1 order of magnitude both in NMP (Figure S61) and DMSO (Figure S62), primarily by the gain in molecular weight (ca. 20%). This effect is interesting, because the sterical demand of the phenyl groups in **PR264** also increases the interplanar distance in the crystal lattice (from 3.25 Å to 3.33 Å, Figure 8) weakening the van der Waals interactions significantly. A similar effect is observed in case of **PO73**, however with dramatic consequences on solubility (Figures S64–S68). Here, the increase of interplanar distance (3.35 Å) triggered by the sterics of two voluminous *tert*-butyl groups and their rotational degrees of freedom leads to the highest solubility in this study of ca. *S*_NMP_ = 4 mg/mL and the third highest in DMSO (*S*_DMSO_ = 700 μg/mL). Despite being known as strong dispersion-energy donors, the *tert*-butyl groups appear to weaken interplanar van der Waals interactions by creating interlayer voids within the crystal lattice, unlike the planar phenyl groups of **PR264** which can maintain close-contact π-π stacking interactions across a comparable interplanar gap. This example illustrates how crystal packing strongly affects solubility, and how simple approximations, such as dispersion-energy donor counts or molecular weight, can yield misleading predictions. Even the solubility of the fully unsubstituted DPP **PR255** (Figures S49–S53) is ca. half as large (*S*_NMP_ = 1.8 mg/mL) despite its much lower molecular weight (65% of **PO73**).

**Figure 9:**
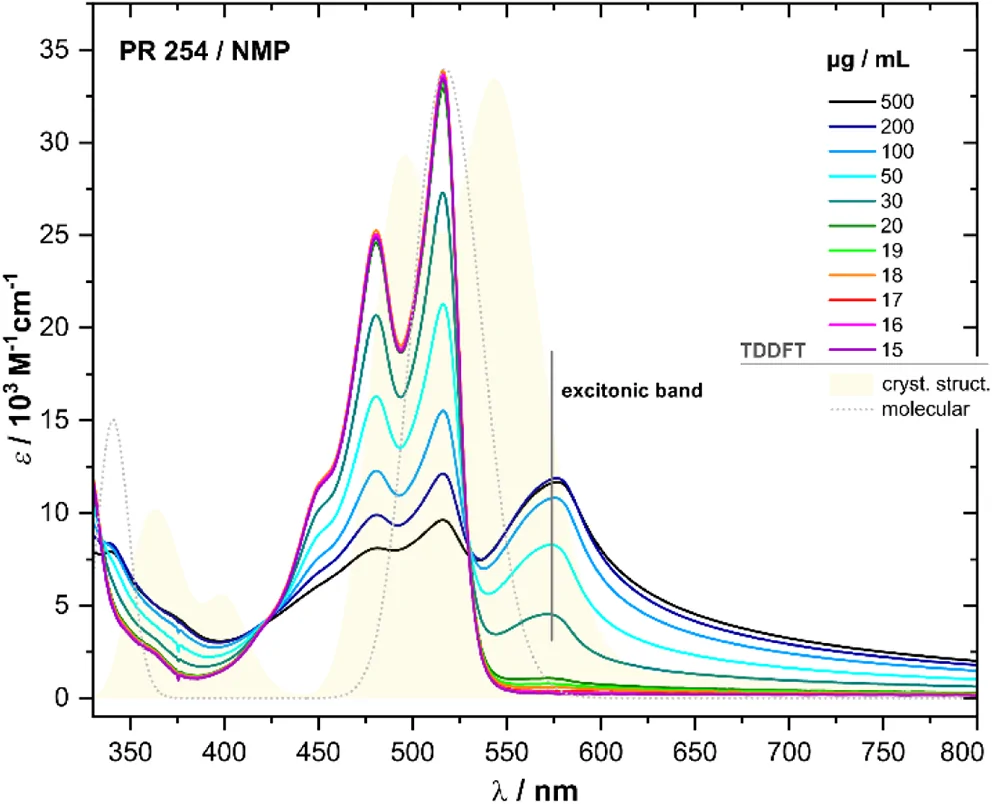
Iterative dilution procedure of **PR254** in NMP. The measurement sequence starts with a dispersion (500 μg/mL) and tracks the UV–vis spectral evolution down to 15 μg/mL. Filled area represents the normalized TDDFT spectrum of a literature-reported crystal structure (Figure S55); the monomer simulation (dotted line) is referenced to the experimental main absorption peak.

#### Phthalocyanine Pigment Class

The phthalocyanine pigments of this study possess the highest symmetry of the whole series (*D*_*4h*_ point group) and represent the only class featuring a chelating pocket for divalent metal ions. However, only the Cu­(II) derivatives are of relevance for the pigment industry.^5,49^ The phthalocyanine backbone can be derived as a benzannulated aza analog from natural porphyrins, while both modifications, benzannulation (aromatic stabilization and shielding) as well as aza replacement (lowering of oxidation potential) crucially contribute to the robustness of this pigment class. In the blue to green color range, copper phthalocyanine pigments are undisputed the major colorants with highest production volumes and the broadest application fields beginning from printing over paints to plastics and even cosmetics.^58^ They are characterized by excellent durability and lightfastness and an outstanding chemical, thermal and photo stability. Typical phthalocyanines exhibit sharp, well-defined absorption bands in their molecular state with extinction coefficients exceeding *ε* = 200,000 M^–1^ cm^–1^. Furthermore, a diverse crystal polymorphism has been disclosed for some members and is industrially exploited. We chose **PB15** and **PG7** as the most prominent representatives (Figure 10), omitting the diversely halogenated **PG36** on purpose because of its highly undefined molecular composition.

**Figure 10:**
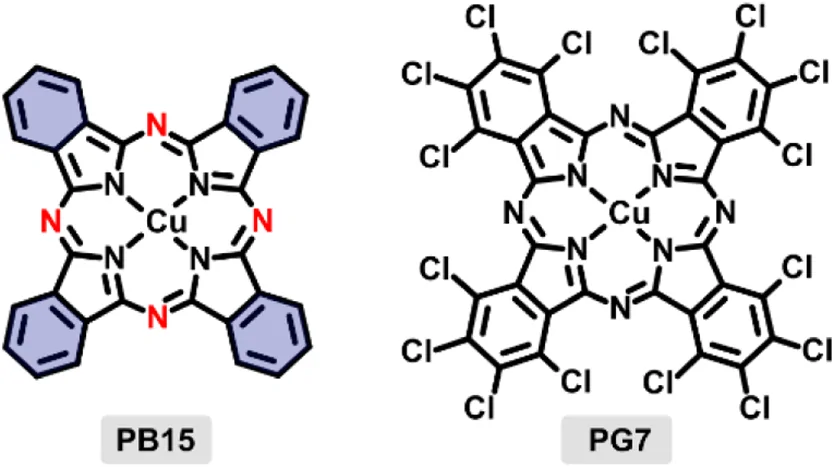
Copper phthalocyanine species used in this study. The thermodynamically most stable β phase of **PB15** (PB15:3) has been used for solubility analysis. The characteristic structural differences to porphyrins are highlighted in blue (benzannulation) and red (aza replacement).

Due to molecular symmetry, planarity and weight the solubilities of **PB15** and **PG7** are the lowest in this study and could only be obtained accurately for **PB15** in NMP and as upper bounds for **PG7** (Figures S74 and S75) and **PB15** in DMSO (Figure S72). These difficulties are well-known and have led to unreliable values and molecular bandshapes in the literature so far.^59^ Figure 11 illustrates the dilution process of **PB15** in NMP contrasted with a correct TDDFT prediction of its red-shifted excitonic band. At advanced dilution, the scattering signal becomes too weak to reliably identify a particle-free solution, a limitation further accompanied by pronounced photometric noise at these extremely low concentrations (<0.2 μg/mL). However, monitoring the excitonic band allowed us to accurately determine the solubility (*S*_NMP_ = 0.07 μg/mL) yielding the correct molecular band shape and extinction coefficient of ε = 210,000 M^–1^ cm^–1^. The solubility of **PG7** is at least an order of magnitude lower than that of **PB15**, as expected from its perchlorination, increased molecular weight and stronger van der Waals interactions.

**Figure 11:**
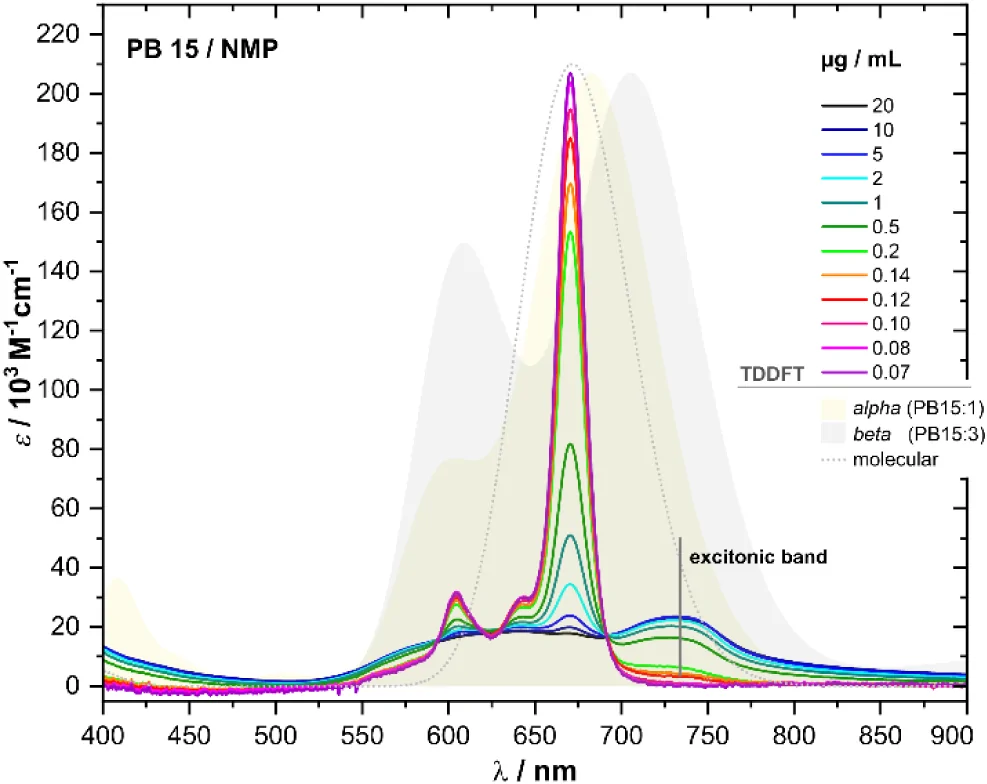
Iterative dilution procedure of **PB15** in NMP. The measurement sequence starts with a dispersion (20 μg/mL) and tracks the UV–vis spectral evolution down to 0.07 μg/mL. Filled areas represent normalized TDDFT spectra of literature-reported crystal structures (Figure S70); the monomer simulation (dotted line) is referenced to the experimental main absorption peak.

#### Quinophthalone and Anthraquinone Pigments

Both substance classes do not comprise a diversified pigment portfolio of industrial or consumer relevance and are thus included by their most prominent representatives **PY138** and **PB60** (Figure 12). However, due to regulatory measures in the EU both pigments currently gain increasing importance in tattooing.^60^ The parent quinophthalone structure is planar but of low symmetry (*C*_*s*_), integrating an electronic push–pull system, similar to merocyanines. Recently, a proton-exchange mechanism (ESIPT) in the excited state has been experimentally proven.^61^ In this elementary form the solubility in organic media is substantially high and would not justify the term pigment. However, the gain in molecular weight by perchlorination of the 1,3-indandion fragment and the derivatization with another perchlorinated phthalimide moiety finally leading to **PY138** decreases the solubility tremendously.^49^ The phthalimide moiety is not in plane with the quinophthalone backbone (Figure S76) and only weakly contributes to the color or UV–vis spectral signature of the pigment. In contrast to the pigments discussed so far, **PY138** possesses a significant net electron deficiency. It shares this quality with **PB60**, which was originally introduced as a Vat dye and a light-fast replacement for indigo by *BASF* under the name Indanthrene (1901). **PB60** is of high symmetry (*C*_*2h*_) and formally a dimerized amino-anthraquinone structure (Figure 12).^62^

**Figure 12:**
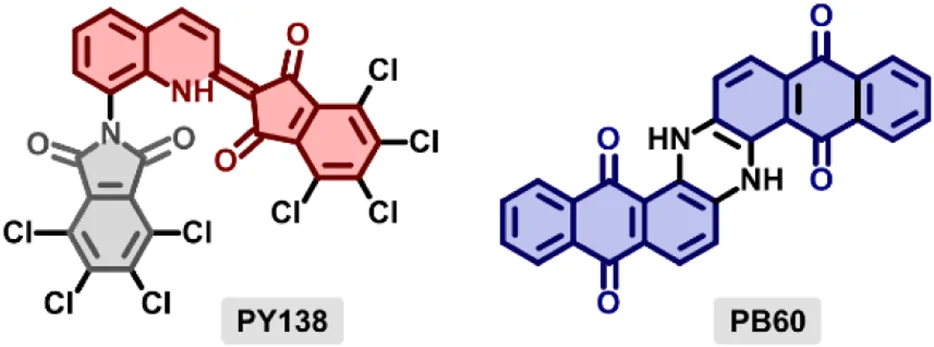
**PY138** and **PB60** as representatives of the quinophthalone and anthraquinone pigment class, respectively. The quinophthalone backbone is highlighted in red and the phthalimide residue in gray. The dimer structure of **PB60** is displayed with its anthraquinone building blocks in blue.

Even though both pigments incorporate compatible electron donor and acceptor groups, no intermolecular hydrogen bonds are present in their crystal structures (Figures S77 and S82). The solubilities, however, are extremely low and could only be determined accurately in NMP: *S*_NMP_ = 0.18 μg/mL (**PB60**, Figures S83–S85) and *S*_NMP_ = 6 μg/mL (**PY138**, Figures S78–S80). Given that the molecular weight of **PY138** exceeds that of **PB60** by about 60%, the comparison highlights how molecular symmetry and planarity (**PB60**) can offset the effect of increased molecular weight (**PY138**). The dilution procedure for **PB60** in NMP is shown in Figure 13, indicating a negligible scattering signal at 900 nm and revealing interconversion of multiple aggregate species upon dilution (no isosbestic point). **PB60** is a rare case in this study, exhibiting a dominant blue-shifted excitonic band, similar to **PO13**. Its relative position was, however, successfully reproduced by our TDDFT approach.

**Figure 13:**
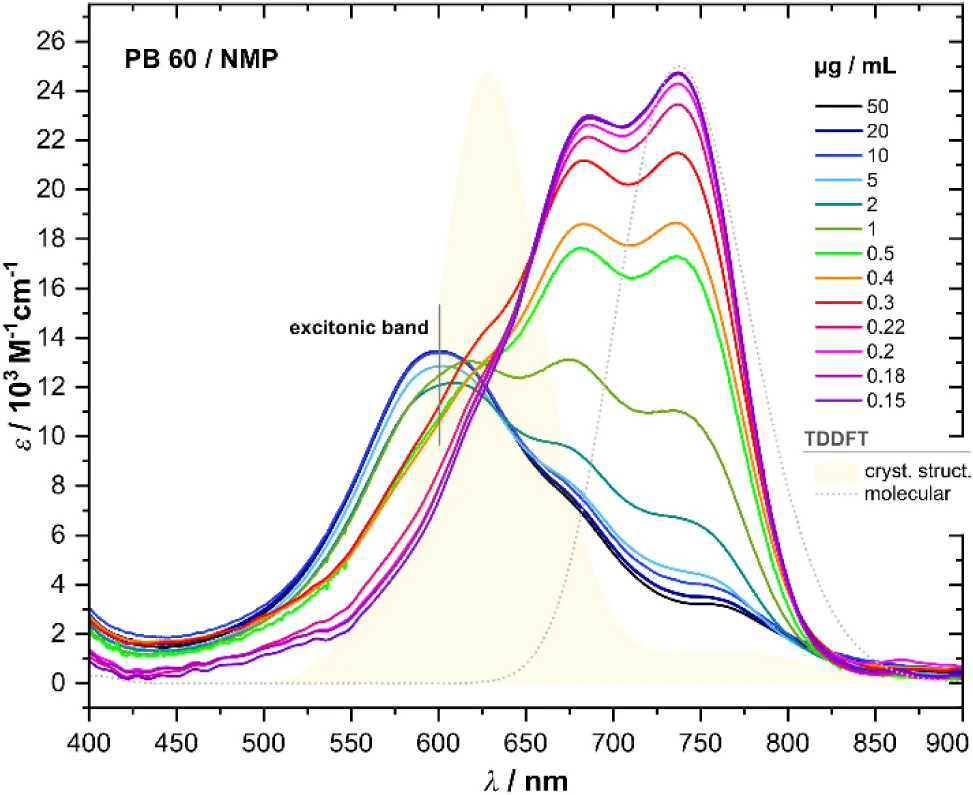
Iterative dilution procedure of **PB60** in NMP. The measurement sequence starts with a dispersion (50 μg/mL) and tracks the UV–vis spectral evolution down to 0.15 μg/mL. Filled area represents the normalized TDDFT spectrum of a literature-reported crystal structure (Figure S82); the monomer simulation (dotted line) is referenced to the experimental main absorption peak.

Table 1 summarizes all experimental solubilities determined in this study, contrasted with molecular weights, molecular extinction coefficients and the wavelengths of main molecular absorption bands.

Molecular weight is a common orientation value to predict physicochemical properties such as melting and boiling point, but also solubility. Despite the importance of specific molecular descriptors (molecular planarity, rigidity etc.) that were discussed above molecular weight is still a crucial contributor to decreasing solubilities irrespective of the solvent chosen, especially due to the increasing dispersion increment within the van der Waals interactions. Figure 14 contrasts experimental solubilities with molecular weights highlighting this overall trend. The comparison is more consistent when focusing on pigments within a class. The main outliers within their respective pigment classes are **PO73**, whose impaired planarity and rigidity lead to a solubility higher than expected, and **PO13**, which conversely exhibits higher structural rigidity and aromaticity (solubility lower than expected).

**Figure 14:**
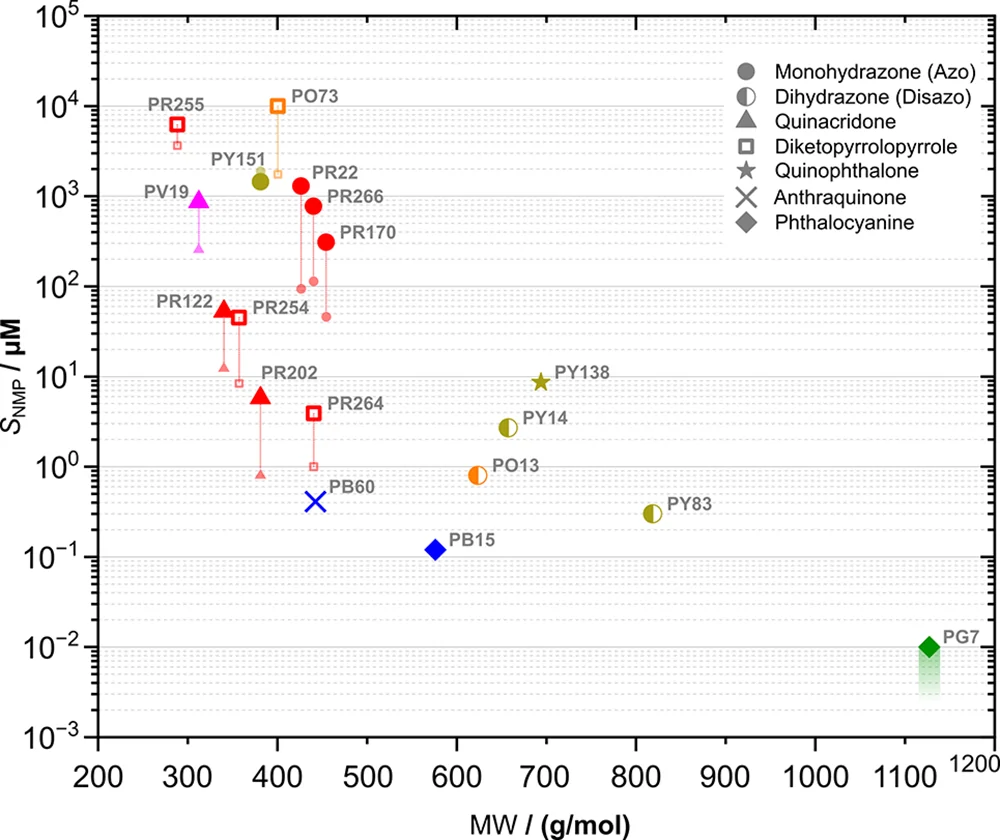
Experimental solubilities in NMP plotted against molecular pigment weights (MW). Shaded, smaller symbols display solubilities in DMSO, if available. The tailing in case of the **PG7** data point indicates its upper bound quality.

### Derivation of Water Solubilities

With the experimental data in hand, we also sought to estimate solubilities in aqueous media, owing to their toxicological relevance for migration processes^63–65^ (consumer products) and for tattooing.^66–68^ The Abraham polyparameter linear free-energy relationship (pp-LFER)^69–72^ expresses phase-pair partitioning as

1 logK=c+eE+sS+aA+bB+vV

where *E*, *S*, *A*, *B*, *V* are molecular solute descriptors (*E*: polarizability/dispersion, *S*: dipolarity, *A*: H-bond acidity, *B*: H-bond basicity, and *V*: McGowan volume) and *c*, *e*, *s*, *a*, *b*, *v* are phase-pair system coefficients calibrated for the solvent/water pair. Using this framework, water solubility *S*_*w*_ can be obtained from an experimental solvent solubility *S*_*exp*_ via

2 $$\log S_{w}=\log S_{exp}-\log K$$

using literature-based system coefficients according to *K* = *S*_*exp*_/*S*_*w*_.^73^ Pigments occupy a rather specialized region of chemical space: rigidified carbon skeleton, high planarity and symmetry, extended π-systems and consequently strong crystal packing. Within the pp-LFER framework, the crystal-lattice term Δ*G_subl_*, however, cancels when converting solubilities between two solvents, because the solution free energy Δ*G_sol_* decomposes as

3 $$\Delta G_{sol}=\Delta G_{subl}+\Delta G_{solv}$$

and, under the standard assumptions (same solid phase in both media and neutral solutes at saturation), Δ*G_subl_* is identical in both systems. Thus, the solvent-to-solvent transformation depends only on the solvation free energy difference Δ*G_solv_* in both solvents. Still, descriptor models are commonly trained on smaller, unrestrained organic molecules and may not capture all molecular signatures typical of pigments. In order to countervail a potential model bias and improve robustness, we therefore used two modern solute-descriptor models available in the free, open-source RMG software package:^74^ a group-contribution approach (*SoluteGC*) and a machine-learning model (*SoluteML*). The phthalocyanine pigments **PB15** and **PG7** had to be parametrized as their copper-free, protonated analogues (H_2_Pc) because neither descriptor model supports metal-containing solutes (see Section 3.1 of the Supporting Information for further details). To combine transparency with methodological conservatism, we applied model averaging per solvent and selected the data set yielding higher solubilities, providing conservative upper-bound estimates with clearer toxicological relevance. Scheme 1 illustrates the complete workflow from experimental solubilities to cytosolic estimates.

**Scheme 1:**
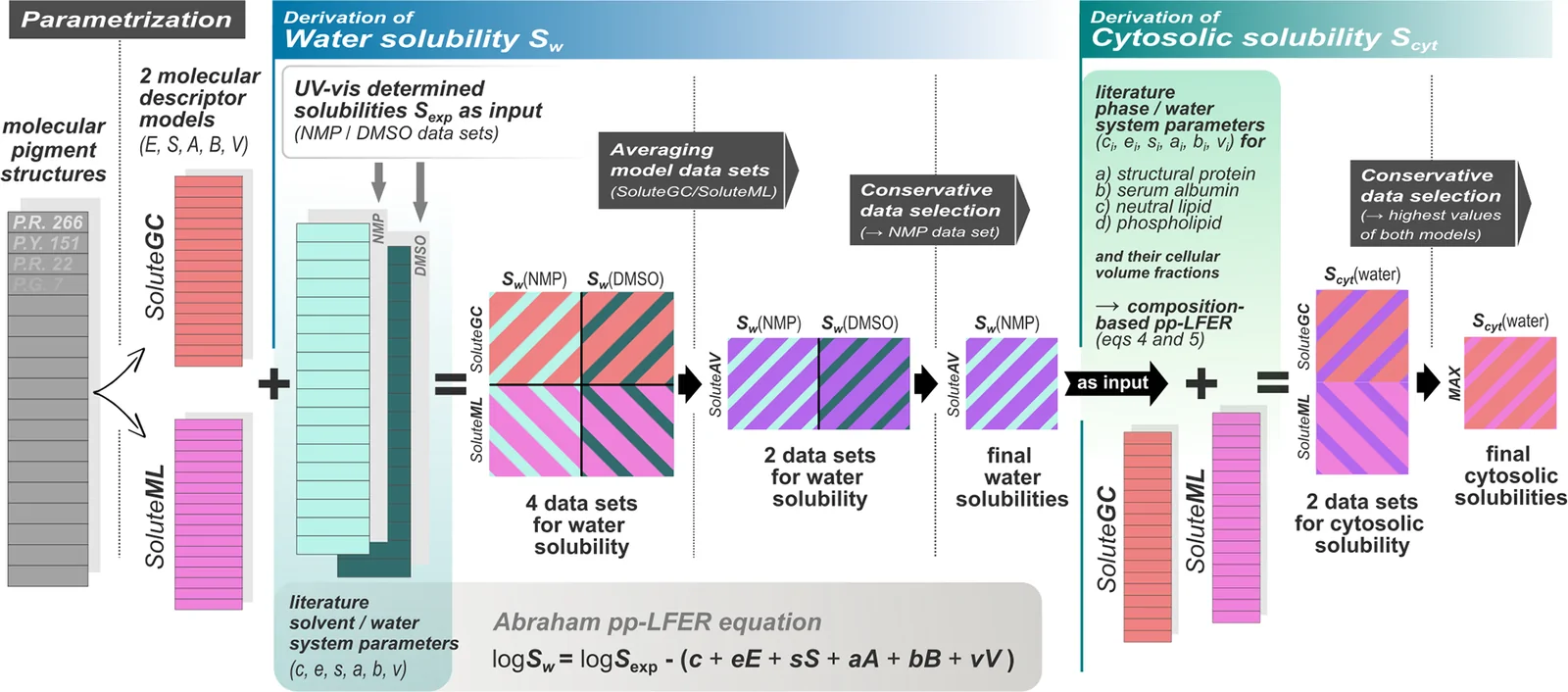
Workflow for Deriving Water and Cytosolic Solubilities from Experimental UV–Vis Data via Abraham-Type pp-LFER Conversions^a^

a The water-to-cytosol transformation follows the composition-based pp-LFER framework (eqs 4 and 5). Conservative data selection was applied at two stages: (1) the NMP-based data set was chosen over DMSO, as it consistently yielded higher water solubilities for all pigments (Figure 15) and (2) the highest values from both descriptor models were selected for the final cytosol solubilities.

For both solvent data sets (NMP and DMSO), the two descriptor models showed high correlations (r_NMP_ = 0.953, r_DMSO_ = 0.952; see Figures S86 and S87) underscoring the consistent treatment of all pigment classes by both algorithms. To reflect this compatibility, the *SoluteGC* and *SoluteML* values were averaged in log space (geometric mean) for each solvent, yielding the composite data sets *SoluteAV* for NMP and DMSO. Figure 15 shows a correlation plot between both solvents. Seven pigments (**PB60**, **PB15**, **PG7**, **PO13**, **PY138**, **PY14**, **PY83**) were omitted from the linear fit and correlation because their DMSO values were only obtained as upper limits as discussed above. On the remaining data set (n = 11) the NMP-derived water solubilities exceed the DMSO-derived values by 1.04 ± 0.26 log units (≈11-fold, 95% CI 7.7–15.7-fold). However, the censored DMSO points (plotted with left-pointing tails) still lie close to the regression line, indicating that the linear relationship persists into the subpicomolar range and that their exclusion does not bias the overall trend. Detailed water solubilities from both models along with their averaged counterparts are compiled in Table S5.

**Figure 15:**
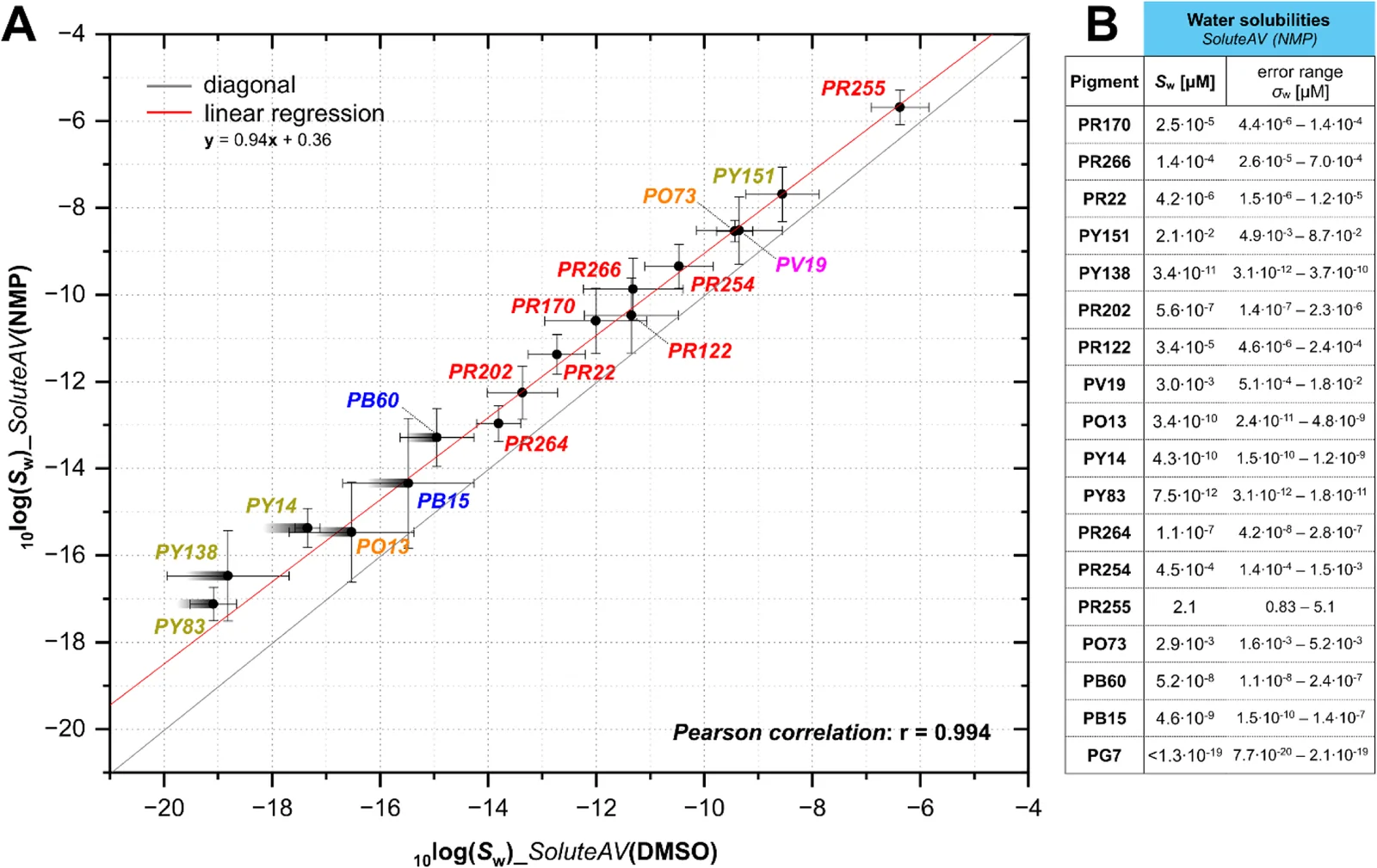
A) Correlation plot of the averaged descriptor data sets *SoluteAV* comparing NMP-derived and DMSO-derived logarithmic water solubilities (mol/L). Symmetric pigment-specific error bars reflect combined uncertainties from experimental reproducibility, pp-LFER calibration and descriptor model divergence (see Supporting Information, Section 4.2). Solid circles represent quantified values (11 of 18); symbols with a left-pointing tail mark DMSO points reported only as upper limits (instrumental censoring). **PG7** falls outside the displayed range and is omitted for clarity (see Table S5). For temperature dependence of modeled water solubilities see Table S7. B) Modeled water solubilities with combined uncertainties, derived from NMP-to-water conversions and averaged over both descriptor models (*SoluteAV*).

Overall, the derived water solubilities are extremely low, typically in the subnanomolar range. Only the lower-molecular-weight species **PV19**, **PR255**, **PO73**, and **PY151** are exceptions. Remarkably, all representatives of the dihydrazone and phthalocyanine families, as well as **PY138**, **PB60**, **PR202**, and **PR264**, exhibit subpicomolar modeled water solubilities. For illustration: if an average human cell contained only water (∼1–2 pL), then these solubilities would roughly correspond to a single dissolved molecule per cell volume on average (or less). An extreme example is **PG7** with *S*_w_ < 1.3 × 10^–19^ μM, which corresponds to a single molecule **PG7** per 13 L water after reaching dissolution equilibrium.

### Derivation of Bioavailability

Water provides only a crude chemical reference frame for toxicological assessment since true bioavailability is controlled by the intracellular milieu. We therefore subjected the conservatively derived water solubilities to a second-tier refinement and estimated solubilities in a composition-based skin-cytosol model. Quantifying the mobile protein and lipid pools that can actually reach a particle and contribute to its dissolution is nontrivial. Pigment particles that entered an organism (e.g., during tattooing) are frequently sequestered in the phagolysosomal lumen of macrophages,^75,76^ yet data on the biomolecular composition of this compartment are scarce. Moreover, most cellular protein is compartmentalized (e.g., within mitochondria), while a potential corona formation would withdraw additional protein and lipid from the free pool and locally increase their concentrations at the particle surface. These opposing uncertainties make detailed corrections speculative. In order to remain transparent and provide defensive and reproducible results, we adopted a literature-known average composition of human cell homogenates,^77^ assuming that the resulting values should represent a conservative upper bound for bioavailable concentrations.

Compositional data for human skin were partitioned into five dominant sorbing phases: bulk water (*f*_w_ = 62% v/v), serum albumin (*f*_sa_ = 0.3% v/v), other (structural) proteins (*f*_prot_ = 28% v/v), neutral lipids (*f*_nl_ = 8.7% v/v) and phospholipids (*f*_pl_ = 1% v/v). Proteins dominate the mass balance and are known to constitute a major hydrophobic sink for many xenobiotics.^78^ Neutral lipids are highly enriched in skin and, together with the smaller phospholipid pool, contribute disproportionately via the large *v*-term of the pp-LFER, which favors the dispersion-dominated interaction profile of pigments. Sugars and inorganic salts, being strongly hydrophilic, have negligible sorptive capacity and were omitted.

For each pigment, phase/water pp-LFERs provide

4 $$\log K_{i/w}=c_{i}+e_{i}E+s_{i}S+a_{i}A+b_{i}B+v_{i}V$$

with i = *prot, sa, nl, pl*

Analogous to the NMP/DMSO-to-water transformation *c*, *e*, *s*, *a*, *b*, *v* denote literature-based phase/water system coefficients that govern phase-partitioning in the pp-LFER.^73,74^

The apparent cytosolic solubility then follows the standard composition model

5 $$\log S_{cyt}=\log S_{w}+\log\left[f_{w}+f_{prot}K_{prot/w}+f_{sa}K_{sa/w}+f_{nl}K_{nl/w}+f_{pl}K_{pl/w}\right]$$

Similarly to the primary derivation of water solubilities, we again employed *SoluteGC* and *SoluteML* molecular descriptors separately and used the final, conservative water solubilities from Scheme 1 (*SoluteAV*, NMP-to-water) as input values. The correlation, however, was less robust for the water-to-cytosol transformation, giving a poor Pearson coefficient of r = 0.869 (Figure S88). Remarkably, pigments with high molecular weight, extended π-systems and hydrophobicity such as the dihydrazone and phthalocyanine series were treated inconsistently resulting in higher solubilities with the *SoluteML* model by ca. 2 orders of magnitude in these cases (Table S6). To consistently comply with a conservative toxicological approach, we took the highest values of both models, respectively. Finally, all pp-LFER system coefficients used in this work are calibrated at 298 K, while biological systems operate near 310 K. To assess this gap, we obtained solvation enthalpies in NMP and water for all 18 pigments via the free, open-source *RMG software package* (*DirectML* model)^74^ and estimated the resulting shift in log*S*_w_ via the van’t Hoff relation (Table S7). The corrections are consistently positive, ranging from 0.05 to 0.15 log units resulting in slightly higher water solubilities at 310 K. However, the propagated model uncertainties (averaged σ = 0.23) exceed the corrections themselves, rendering a numerical adjustment inconclusive. For the water-to-cytosol step, the temperature effect works in the opposite direction: partitioning into proteins and lipids is predominantly exothermic and therefore becomes less favorable at higher temperature, partially offsetting the increase in *S*_w_. The net correction on *S*_cyt_ is thus even smaller and not unambiguously predictable in sign. Taken together, the 298 → 310 K shift remains well within the intermodel divergence and does not alter the toxicological conclusions drawn below. Figure 16 presents the entire picture of experimental and pp-LFER transformed solubilities and contrasts trends within a pigment class.

**Figure 16:**
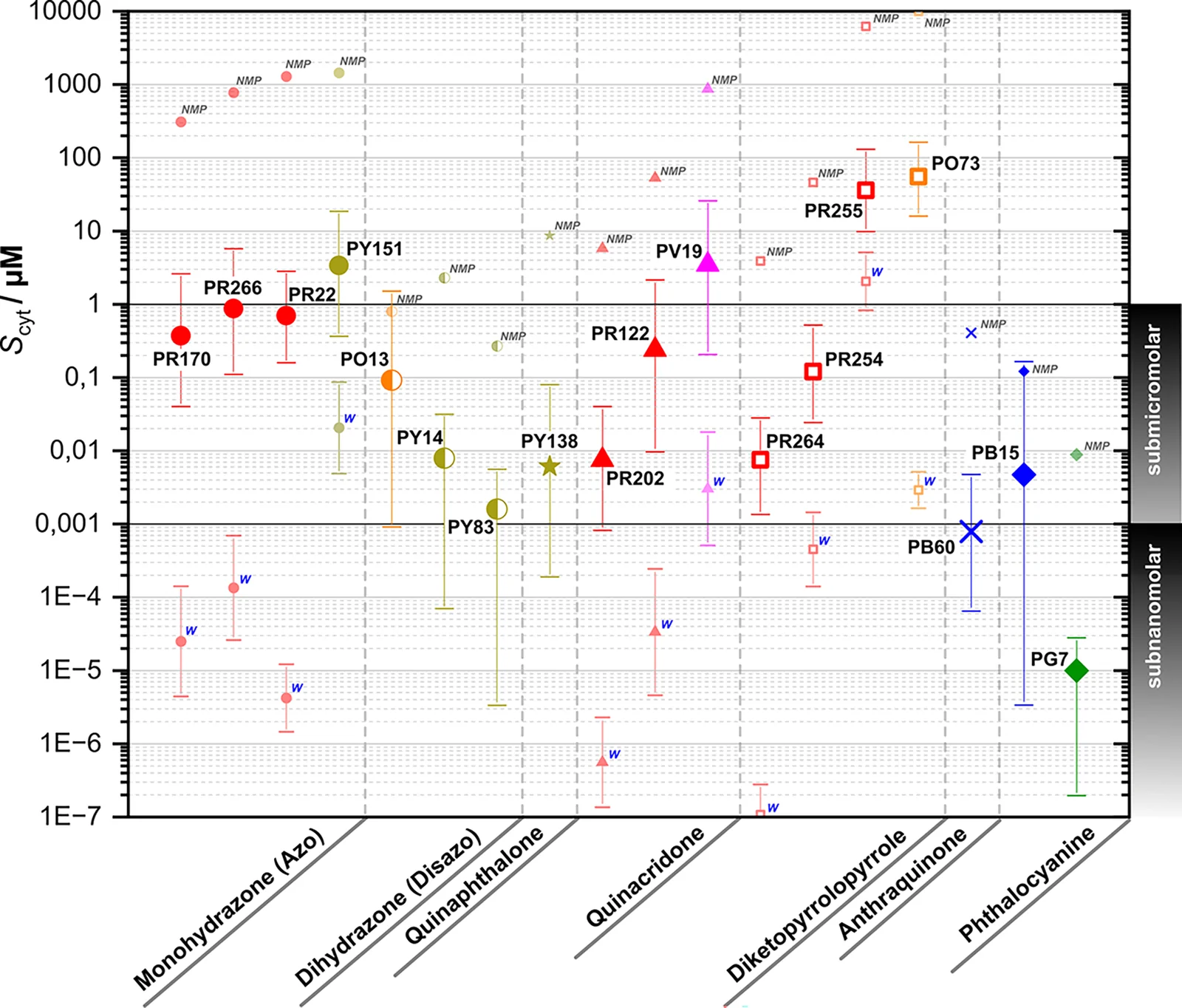
Cytosolic solubilities *S*_cyt_ derived from water-to-cytosol transformations (highest values of both models *SoluteGC* and *SoluteML*, respectively, are presented). Conservative NMP-to-water values (*SoluteAV*) were taken as input solubilities. Asymmetric error bars reflect propagated uncertainties from experimental reproducibility, pp-LFER calibration, and descriptor model divergence; downward bars additionally include the full *SoluteGC–SoluteML* divergence from the water-to-cytosol transformation (see Supporting Information, Section 4.3). Modeled cytosolic values are shown alongside experimental solubilities in NMP and with water counterparts (marked with a blue “W”). Modeled water solubilities of the dihydrazone series, **PY138**, **PB60** and the phthalocyanines are too low to be displayed (see Table S5 for full data).

The modeled cytosolic solubilities show excellent agreement with chemical reasoning and the underlying principles of the pp-LFER model, which merits brief discussion. Within the monohydrazone class, **PR22** does not follow the rising trend from **PR170** to **PY151** in NMP, both in cytosol and, even more dramatically, in water. Whereas **PR170**/**PR266** and **PY151** bear hydrophilic hydrogen-bond donor/acceptor motifs (e.g., amide in **PR170**/**PR266**, carboxylic acid and urea in **PY151**), **PR22** features only a nitro substituent with much lower Abraham *A* and *B* descriptors (H-bond acidity and basicity). Consequently, its reduced H-bonding capacity weakens the affinity to aqueous phases and leads to a lower solubility as the medium becomes more water-rich. Another noteworthy feature appears for **PO13** and **PY14** in the dihydrazone series. The experimental solubility of **PO13** in NMP is remarkably low, consistent with its increased rigidity, which enhance π–π stacking/crystal packing as outlined above. By contrast, **PY14** exhibits higher polarity and hydrogen-bonding capacity (reflected in its higher *S*, *A*, and *B* descriptors; Tables S1 and S2), reducing its affinity for the comparatively hydrophobic cytosolic sink (protein/lipid) relative to water, and thereby explaining its lower cytosolic solubility compared to **PO13**. Another instructive case is the extremely low modeled water solubility of **PO73**. While its NMP cytosolic solubilities follow the general trend of the series, the modeled water solubility of **PO73** is about 3 orders of magnitude lower than that of the congener **PR255**. This is the result of the bulky, hydrophobic *tert*-butyl groups, which substantially increase the McGowan molecular volume (*V* descriptor), strengthening dispersion interactions with organic phases (solvents, proteins, neutral lipids) and thereby markedly reducing affinity for water.

The inclusion of proteins and lipid components to the system strongly increases the solubility of pigments in a medium by many orders of magnitude, ranging from 2 (**PV19**, **PR255**, **PO73**, **PY151**) up to 13 (**PG7**) with a median increase of ca. 4 orders of magnitude. Three conservative steps were applied in deriving cytosolic solubilities: first, the NMP-to-water transformation was used, as it consistently yielded higher water solubilities than the DMSO-to-water conversion; second, the full biomolecular load of cell homogenates (e.g., full lipid fraction) was included for the water-to-cytosol step; and third, the highest resulting values from both models were adopted. Even under these deliberately overestimating conditions, most pigments still display modeled cytosolic solubilities in the submicromolar range. Additionally, their pronounced chemical inertness and the likely requirement for metabolic activation before exhibiting any biomolecular reactivity might preclude detectable responses in many in vitro assays, especially sensitization. The two major cell-based in vitro sensitization platforms: (i) ARE-Nrf2 keratinocyte reporters (KeratinoSens/LuSens), and (ii) dendritic-cell activation assays (h-CLAT/U-SENS/IL-8 Luc) detect points of departure (PoD) values at or above the 1 μM threshold for most potent benchmark compounds. For context, two comprehensive (ring) studies reported PoDs of 2.21 μM (h-CLAT; EC150@CD86)^79^ and 3.28 μM (KeratinoSens; EC1.5, lab average)^80^ for MCI (chlormethylisothiazolinone), and 2.3 μM (h-CLAT; EC150@CD86)^79^ and 2.4 μM (KeratinoSens; EC1.5, lab average)^80^ for DNCB (2,4-dinitrochlorobenzene). Only the solubilities of the low-molecular-weight species **PV19**, **PR255**, **PO73**, and **PY151** fall within a comparable detection range, yet they lack the strongly electrophilic reactive centers characteristic of most potent sensitizers.

Unlike sensitization, genotoxicity involves damage to DNA, a highly sensitive cellular target that is replicated on well-defined time scales, making it particularly vulnerable to single modification events. Therefore, PoDs can be inherently lower for genotoxins than for sensitizing agents. Aflatoxin B1, as an example of a highly potent genotoxic carcinogen, requires metabolic epoxidation to exert electrophilic reactivity toward DNA bases, a mechanism that might be furthermore promoted by a tendency for DNA intercalation.^81^ Increases in micronuclei have been observed already at 0.025 μM in 3D HepG2 liver cell models,^82^ concentrations that fall within the range of some modeled cytosolic solubilities reported in this study. The planar geometries of pigments may similarly promote affinity to DNA via intercalation and could induce noncovalent lesions even without metabolic activation. A related mode of action is reported for classical, neutral intercalators such as doxorubicin^83^ or psoralenes.^84^ However, harmful intercalation requires a finely tuned molecular structure and cannot be inferred solely from simple geometric parameters (e.g., planarity) or functional groups. Taken together, genotoxicity remains a toxicological end point that is, in principle, compatible with the modeled cytosolic solubilities reported here. Beyond direct chemical reactivity, pigment molecules in their electronically excited states may act as photosensitizers, capable of generating reactive oxygen species (ROS) or initiating radical-mediated reactions with biomolecules, especially in light-exposed applications such as tattooing. These mechanisms are well established in synthetic photocatalysis. In this context, the extremely low solubilities reported here may mitigate molecular ROS generation, largely restricting photosensitized pathways to the particulate phase.

It is of importance to note that all toxicological considerations are derived from modeling and are restricted to substances (pigments) of adequate purity and to experiments conducted in the absence of external stimuli, particularly of light. They do not account for ubiquitous contaminants, photodegradation products, or light-induced dissolution processes that could yield toxic concentrations relevant to both sensitization and genotoxicity.

## Conclusions

In this study we present a novel and straightforward methodology to obtain experimental solubilities for substances that are typically deemed insoluble. By combining an iterative dilution protocol with UV–vis spectroscopy, the approach can sensitively quantify solubility for any substances with a characteristic UV–vis signature. Eighteen pigments across seven classes were examined in NMP and DMSO, providing an overview of a diverse chemical space and including different solvent–solute interactions. Rigorous exclusion of particulate contributions, corroborated by computational simulations at the TDDFT level of theory, delivered unprecedented accuracy for solubilities and molecular UV–vis signatures (band shape and extinction coefficient), a facet largely overlooked in many studies. All experimental solubilities perfectly align with physicochemical expectations, underscoring both the consistency of the method and a sufficient purity and stability of the investigated materials in the media. The study confirms the inverse relation between molecular weight and solubility, while pinpointing molecular characteristics that systematically deviate from this simple rule. Experimental values were further used to infer solubilities in water and cytosol via Abraham-type solvation models and composition-based pp-LFER frameworks. In 14 of 18 cases, the modeled water solubilities fall in the subnanomolar range, questioning the occurrence of toxicologically relevant concentrations in water-dominated media that lack appropriate solubilizers. Incorporating biomolecules into the model framework increased solubility and bioavailability by several orders of magnitude, potentially reaching relevant levels in some cases (**PR255**, **PO73**). Nevertheless, most pigments, especially dihydrazone and phthalocyanine species, remain at concentrations likely below the sensitivity of many toxicological assays, particularly for sensitization end points. These findings align with previous observations and provide an additional perspective on why standard patch-testing protocols and genotoxicity assays often remain negative despite the presence of structural alerts such as (di)­hydrazone motifs. Importantly, our analyses do not account for impurities or degradation products, which may exceed critical solubility thresholds and thus contribute to observed toxicological effects.

## Supplementary Material



## Notes

Computational details, UV–vis spectra from dilution experiments, crystal cutouts used for TDDFT simulations, correlation plots, solute and system descriptors, comprehensive solubility data, and DLS data (PDF)
