# Supplementary material for: Quantifying Ultra-Low Pigment Solubilities by UV–Vis Spectroscopy: Implications for Toxicological Assessment
Source: ACS Meas Sci Au. 2026 Jun 18;6(4):957–71. doi: 10.1021/acsmeasuresciau.5c00214 (PMC13495719; doi:10.1021/acsmeasuresciau.5c00214)
Supplement: Supplementary file 1 [file tg5c00214_si_001.pdf]

# Supporting Information

## Quantifying Ultra-Low Pigment Solubilities by UV-Vis Spectroscopy: Implications for Toxicological Assessment

Lukas J. Patalag,[1,*]

[1] Department Chemical and Product Safety; German Federal Institute for Risk Assessment (BfR), Max-Dohrn-Str. 8-10, 10589 Berlin, Germany

## Table of content

1. **Computational Details** .......... **S2**
2. **Pigment Data**
   2.1 *Monohydrazone (Azo) Pigment Class*
      2.1.1 Pigment Red 170 .......... S3
      2.1.2 Pigment Red 266 .......... S5
      2.1.3 Pigment Red 22 .......... S7
      2.1.4 Pigment Red 151 .......... S9
   2.2 *Dihydrazone (Disazo) Pigment Class*
      2.2.1 Pigment Orange 13 .......... S11
      2.2.2 Pigment Yellow 14 .......... S13
      2.2.3 Pigment Yellow 83 .......... S15
   2.3 *Quinacridone Pigment Class*
      2.3.1 Pigment Violet 19 .......... S18
      2.3.2 Pigment Red 122 .......... S20
      2.3.3 Pigment Red 202 .......... S22
   2.4 *Diketopyrrolopyrrole Pigment Class*
      2.4.1 Pigment Red 255 .......... S24
      2.4.2 Pigment Red 254 .......... S26
      2.4.3 Pigment Orange 73 .......... S30
   2.5 *Phthalocyanine Pigment Class*
      2.5.1 Pigment Blue 15 .......... S32
      2.5.2 Pigment Green 7 .......... S34
   2.6 *Quinophthalone and Anthraquinone Pigments*
      2.6.1 Pigment Yellow 138 .......... S35
      2.6.2 Pigment Blue 60 .......... S37
3. **Solvation Modelling**
   3.1 *Solute Descriptors* .......... S41
   3.2 *Solvent Descriptors* .......... S42
   3.3 *Tissue Descriptors* .......... S42
   3.4 *SoluteGC-SoluteML Correlation Plots* .......... S42
   3.5 *Water Solubilities* .......... S44
   3.6 *Cytosol Solubilities* .......... S45
4. **Uncertainty Analysis of pp-LFER Solubility Conversions**
   4.1 *Propagated Uncertainties in Descriptor Model Comparisons* .......... S46
   4.2 *Pigment-Specific Uncertainties in Water Solubility Estimates* .......... S46
   4.3 *Pigment-Specific Asymmetric Uncertainties in Cytosolic Solubility Estimates* .......... S46
   4.4 *Solvation and Transfer Enthalpies for Temperature Adjustment (298 K → 310 K)* .......... S47
5. **DLS Archive** .......... **S48**
6. **References** .......... **S68**

## 1. Computational Details

Computations were run on a Desktop Workstation under Windows with the ORCA 6.0 program package,[1] except for PB15, for which the JUSTUS 2 cluster (bwHPC, Linux) was used due to computational demands. Crystal structures were obtained from the publicly available Cambridge Crystallographic Data Centre (CCDC) database. Since commercial pigment samples typically do not specify their crystal phase (except PB15), all available literature-reported crystal structures for each pigment were computed to cover potential polymorphic variability.

A representative ensemble of monomers within each crystal structure (4-12 monomers, ca. <500 atoms total) was extracted to capture dominant intermolecular coupling interactions. The selection followed systematic criteria: (a) π-π stacking interactions were prioritized due to their strongest contribution to excitonic coupling; (b) hydrogen-bonding interactions were included when present; and (c) a balanced representation of head-to-tail and lateral molecular arrangements was maintained, considering the closest proximity between transition dipole moments based on the specific molecular structure of each pigment. Specific cluster sizes for individual pigments are documented in the respective pigment sections below. Isolated monomer structures were extracted from crystal structures and geometrically optimized using Grimme's $r^2$SCAN-3c composite electronic structure method,[2] which intrinsically accounts for dispersion interactions. This optimization removed crystal packing strain to approximate solution-phase geometries. To maintain computational efficiency across 18 pigments and their polymorphs, all calculations were performed in vacuo without explicit solvent models.

Adiabatic vertical excitation energies were computed using time-dependent density functional theory (TDDFT) with the PBE0 functional[3] and the def2-SVP basis set,[4] employing the Tamm-Dancoff approximation (TDA). The PBE0/def2-SVP combination was selected after preliminary testing on representative pigments (including CAM-B3LYP, ωB97X-D3, and M06-2X), which identified PBE0/def2-SVP as providing the best balance between consistency and computational cost for our diverse pigment series. PBE0 has also demonstrated outperforming performance for similar extended π-systems in the literature.[5-7] No explicit dispersion corrections were applied to the TDDFT calculations, as dispersion primarily affects ground-state properties rather than vertical excitation energies at fixed geometries. However, due to the omitted solvent model, lack of vibronic refinement, and inherent systematic deviations in TDDFT-derived excitation energies, computed absolute energies were expected to deviate from experimental values by approximately 0.3-0.5 eV, as commonly reported for organic chromophores.[7] To address this systematic shift and focus on relative spectral changes (excitonic red- or blue-shifts), an empirical energy alignment procedure was applied. For each pigment, the energy difference between the computed monomer main band and the experimentally observed main band in NMP or DMSO was calculated:

$$\Delta E^{\text{shift}} = E^{\text{exp}}(\text{monomer}) - E^{\text{calc}}(\text{monomer, gas phase})$$

This pigment-specific shift was then uniformly applied to the corresponding crystal cluster spectrum, enabling direct visualization of the relative position of the aggregate spectrum with respect to the experimental monomer reference. This approach validates whether excitonic coupling produces red-shifts (J-aggregates) or blue-shifts (H-aggregates) without requiring absolute quantitative accuracy in transition energies. All TDDFT results were processed and visualized using UCSF ChimeraX. Simulated spectra were plotted as Gaussian-broadened stick spectra (FWHM = 0.2 eV) and normalized for comparison with experimental UV-vis data.

## 2. Pigment Data

### 2.1 Monohydrazone (Azo) Pigment Class

#### 2.1.1 Pigment Red 170 (PR170)

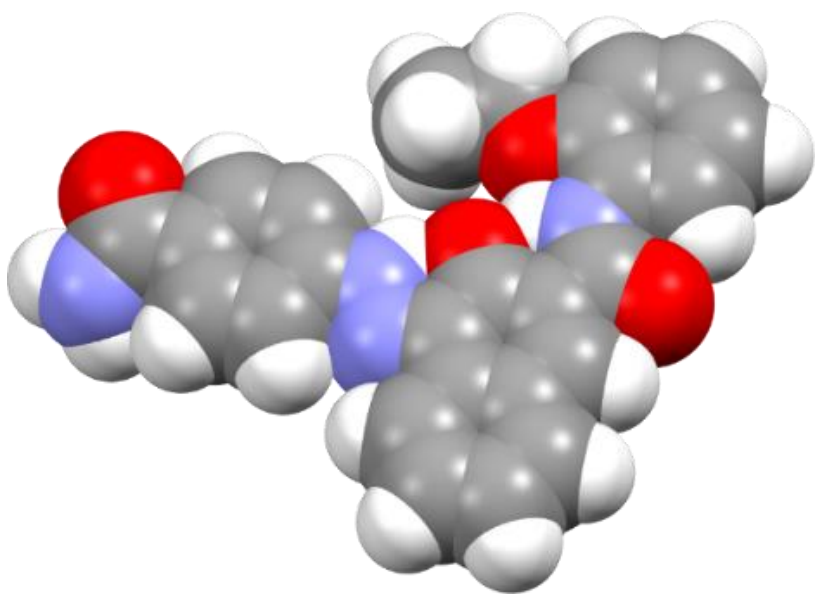

**Figure S1**. Molecular structure of PR170 after geometrical optimization in vacuo (DFT: r$^2$SCAN-3c).

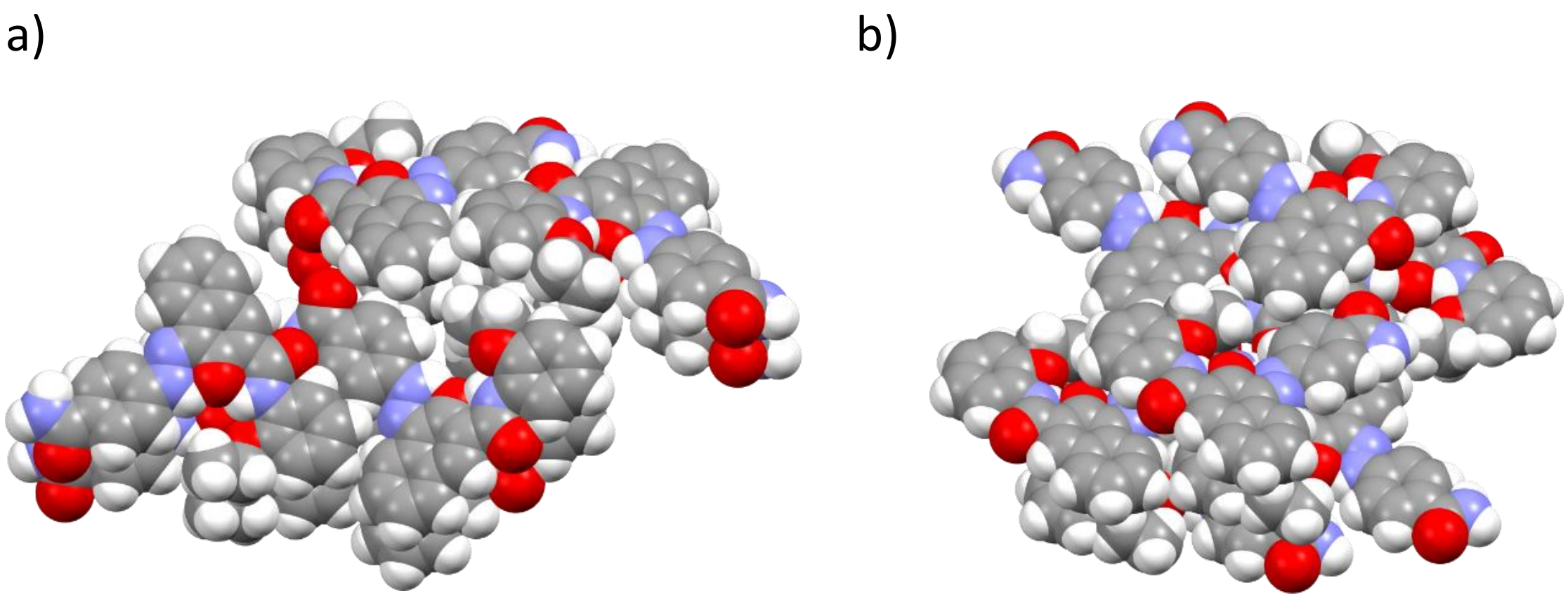

**Figure S2**. Pigment molecule ensembles (PR170) extracted from respective literature-known crystal structures, a) *alpha* structure: CCDC-270077[8], b) *gamma* structure: CCDC-276530[8]. Depicted cut-outs (8 pigment molecules each) were used for TDDFT simulations, see Computational Details.

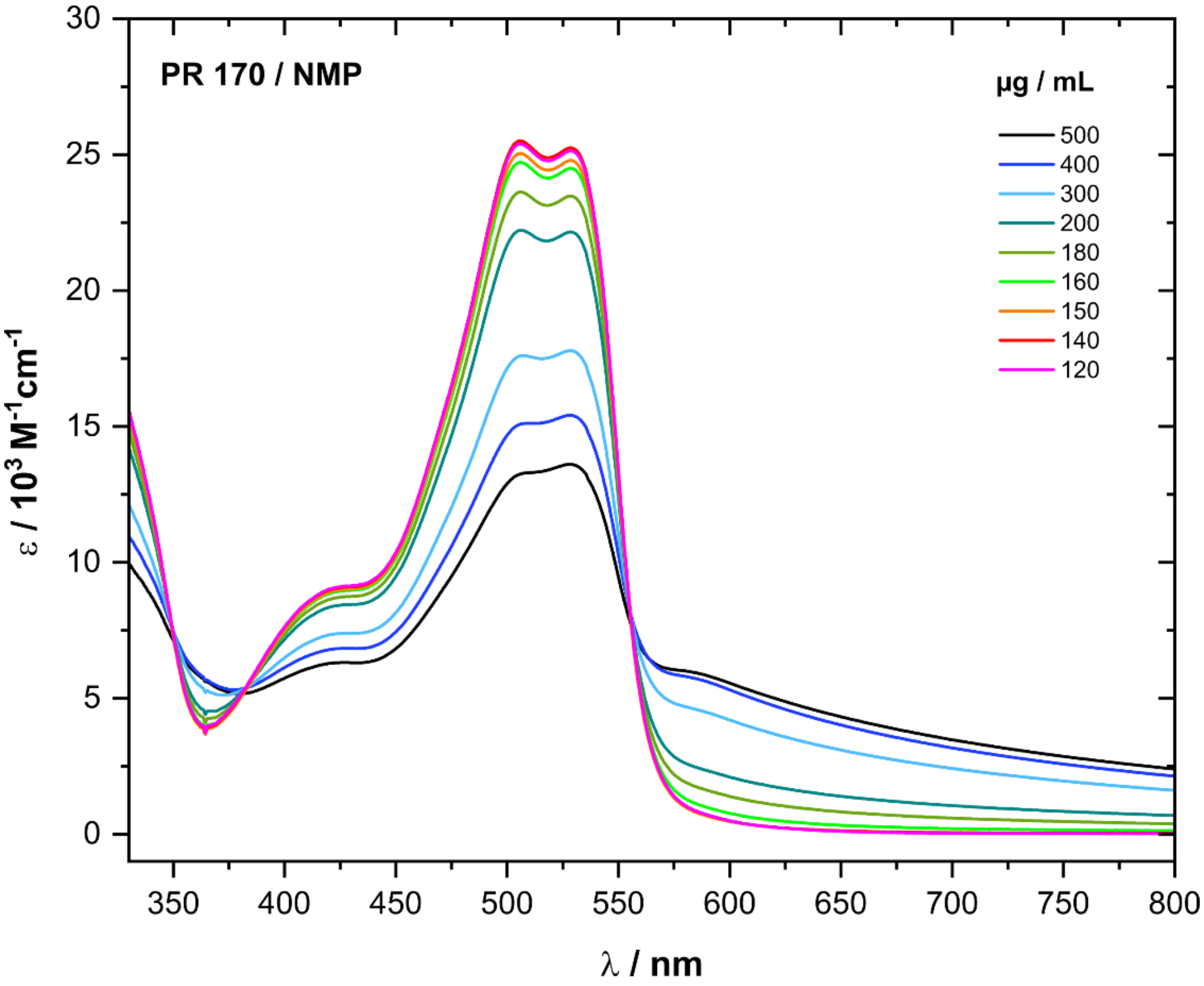


**Figure S3**. Iterative dilution procedure of PR170 in NMP, measured in a 0.1 cm quartz cuvette. The measurement sequence starts with a dispersion (500 µg/mL) and tracks the UV-vis spectral evolution down to 120 µg/mL.

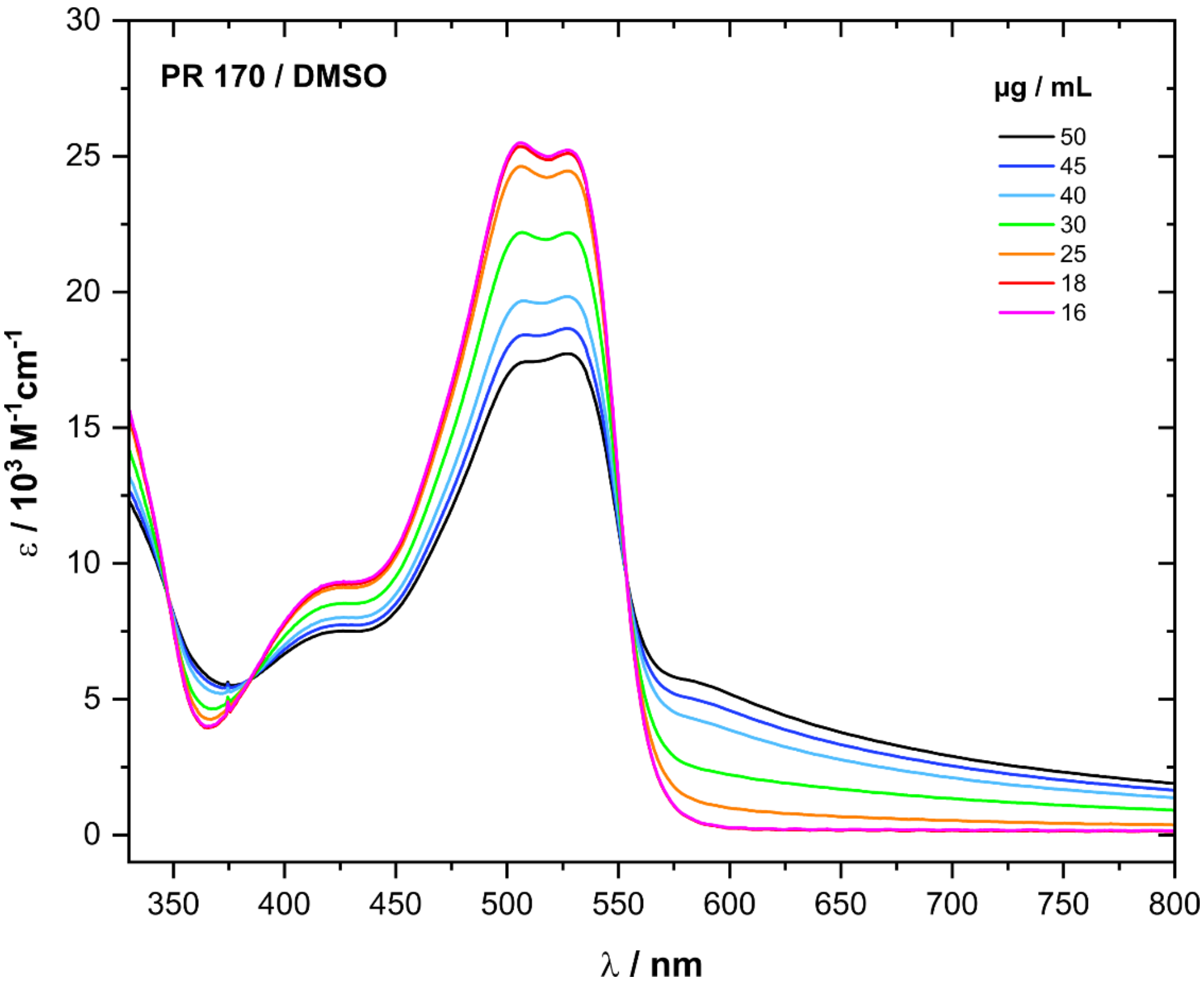


**Figure S4**. Iterative dilution procedure of PR170 in DMSO, measured in a 0.5 cm quartz cuvette. The measurement sequence starts with a dispersion (50 µg/mL) and tracks the UV-vis spectral evolution down to 16 µg/mL.

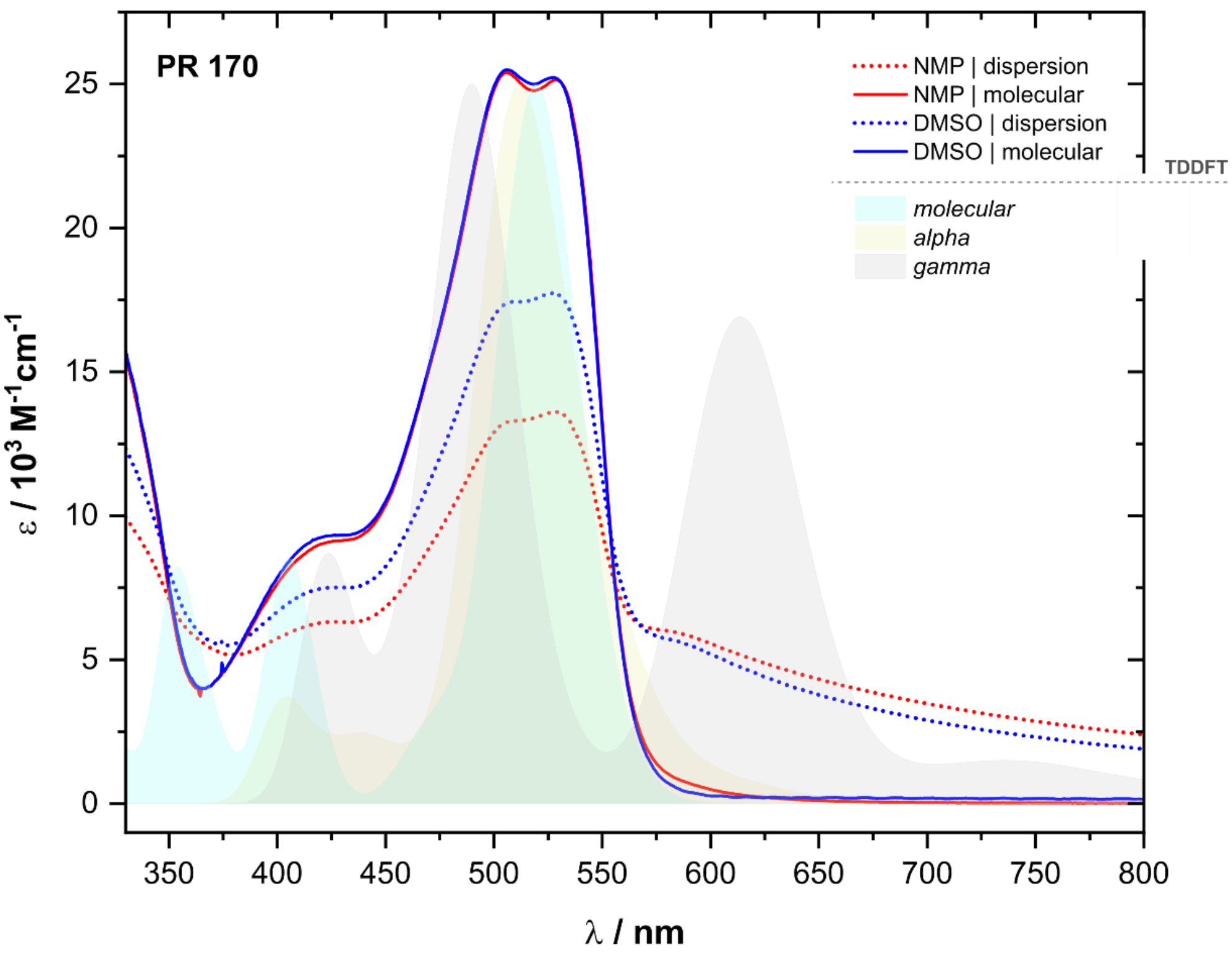


**Figure S5**. Final molecular UV-vis spectra of PR170 in NMP and DMSO (solid lines), contrasted with corresponding dispersion spectra and normalized TDDFT simulations. TDDFT simulations were shifted by -0.68 eV, respectively; peak type is Gaussian, 0.2 eV FWHM.

### 2.1.2 Pigment Red 266 (PR266)

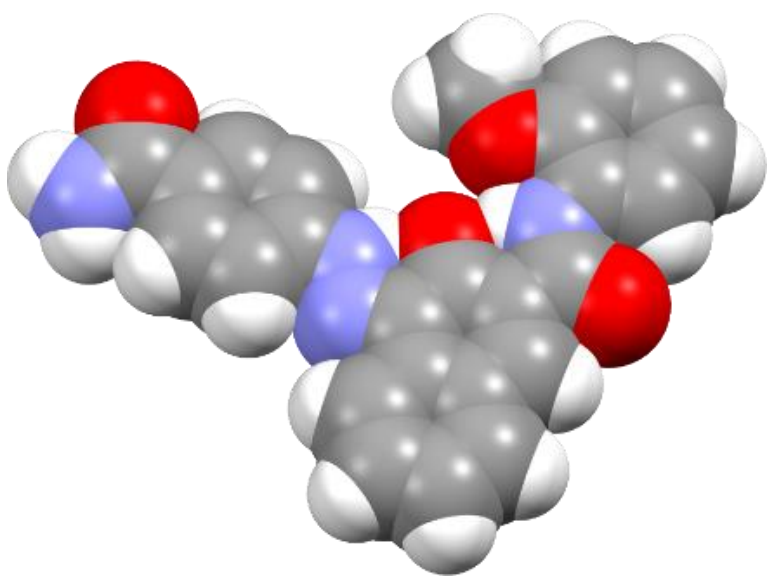

**Figure S6**. Molecular structure of PR266 after geometrical optimization in vacuo (DFT: r$^2$SCAN-3c).

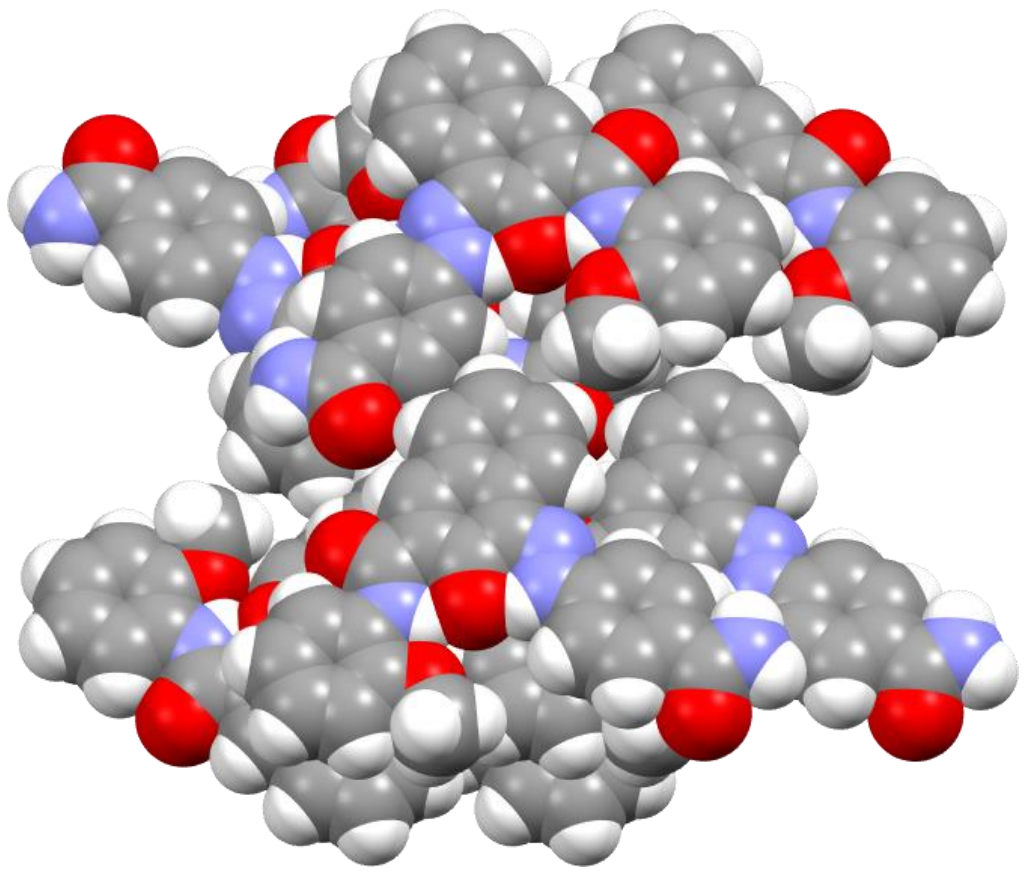

**Figure S7**. Pigment molecule ensemble (PR266) extracted from respective literature-known crystal structures: CCDC-224651.[9] Depicted cut-out (8 pigment molecules) was used for TDDFT simulations, see Computational Details.

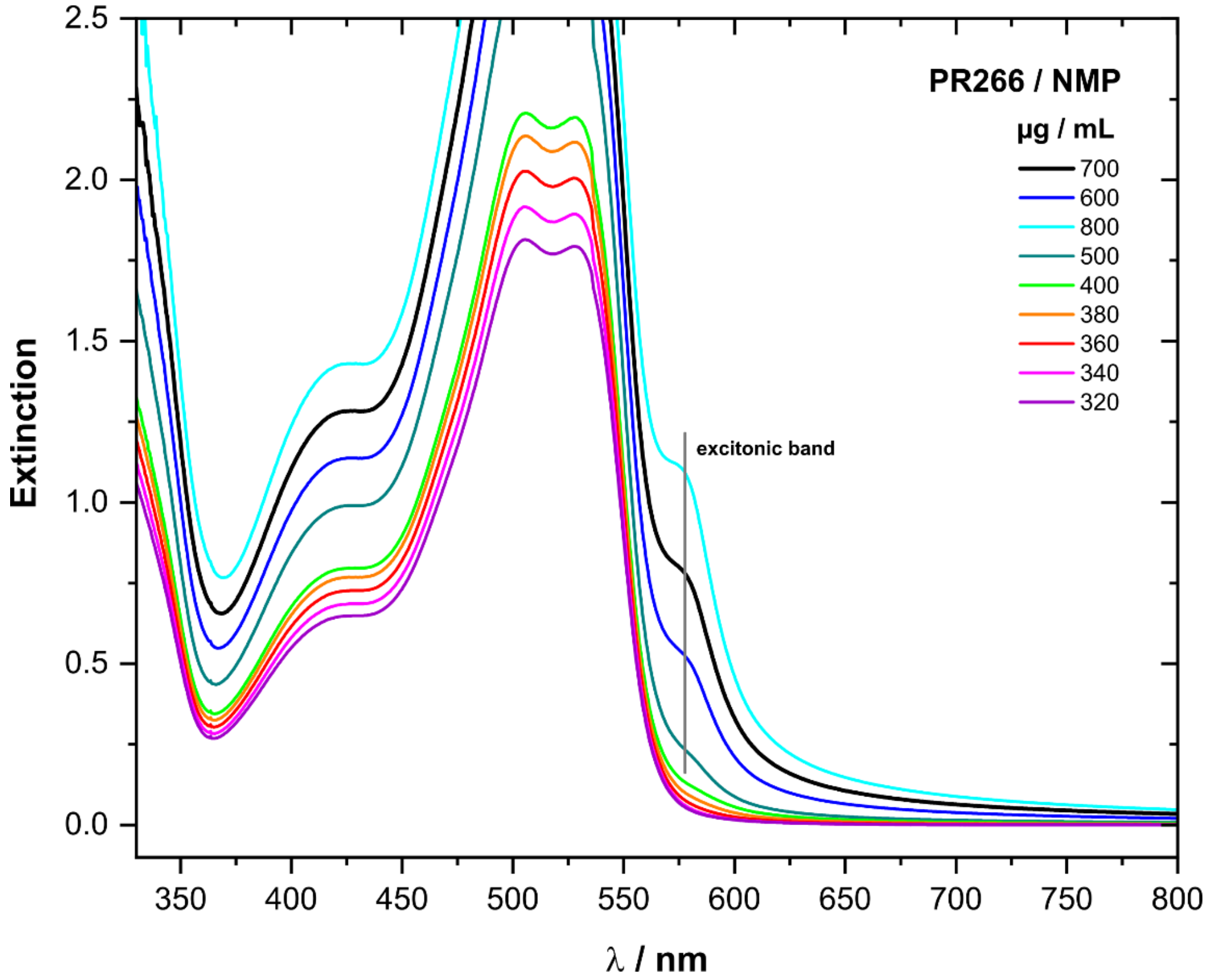


**Figure S8**. Iterative dilution procedure of PR266 in NMP, measured in a 0.1 cm quartz cuvette. Despite the lowest optical pathlength available (0.1 cm) the extinction exceeds the threshold of 1. The decline of the particle or excitonic band at ca. 580 nm was used as indicator for the transition point instead. The measurement sequence starts with a dispersion (700 µg/mL) and tracks the UV-vis spectral evolution down to 320 µg/mL.

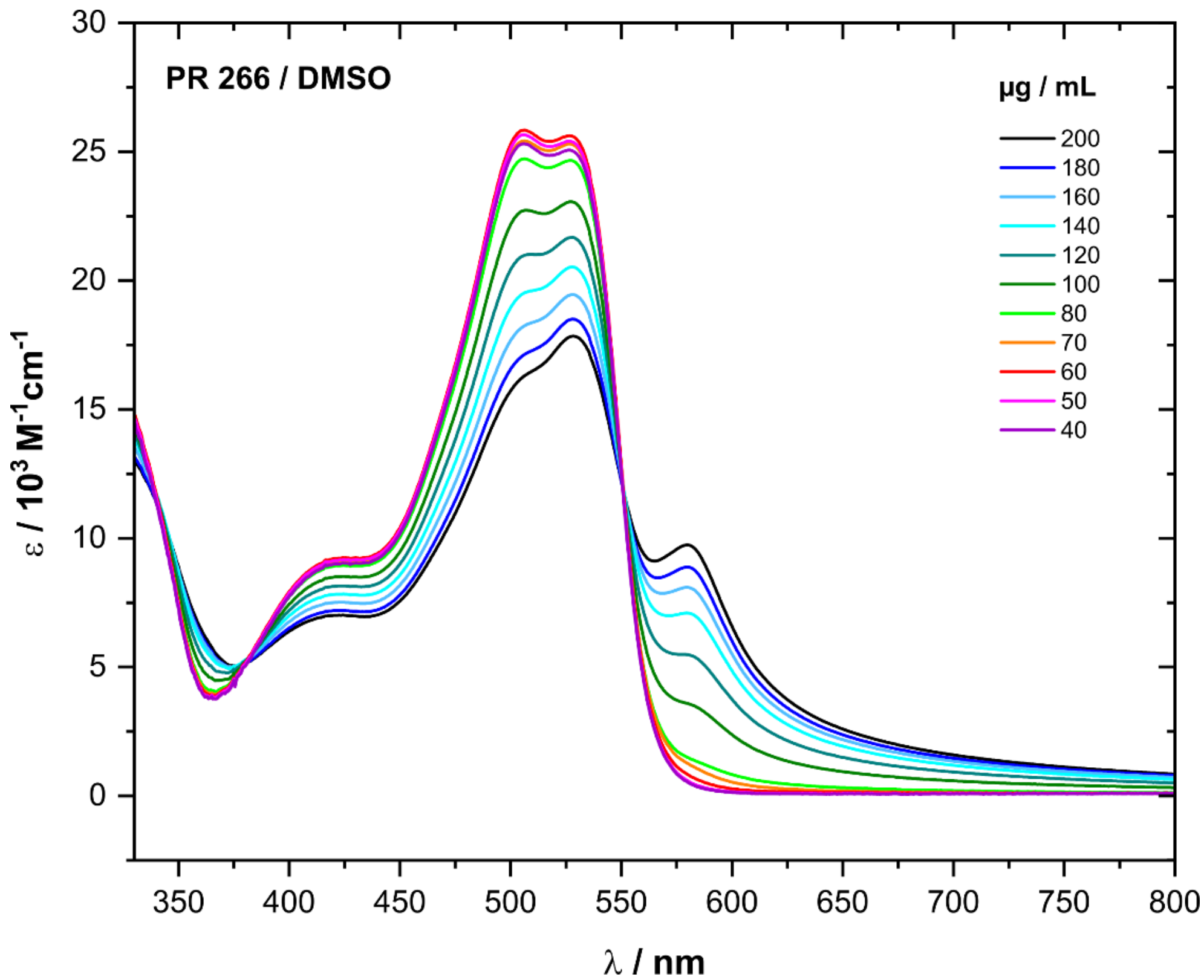


**Figure S9**. Iterative dilution procedure of PR266 in DMSO, measured in a 0.1 cm quartz cuvette. The measurement sequence starts with a dispersion (200 µg/mL) and tracks the UV-vis spectral evolution down to 40 µg/mL.

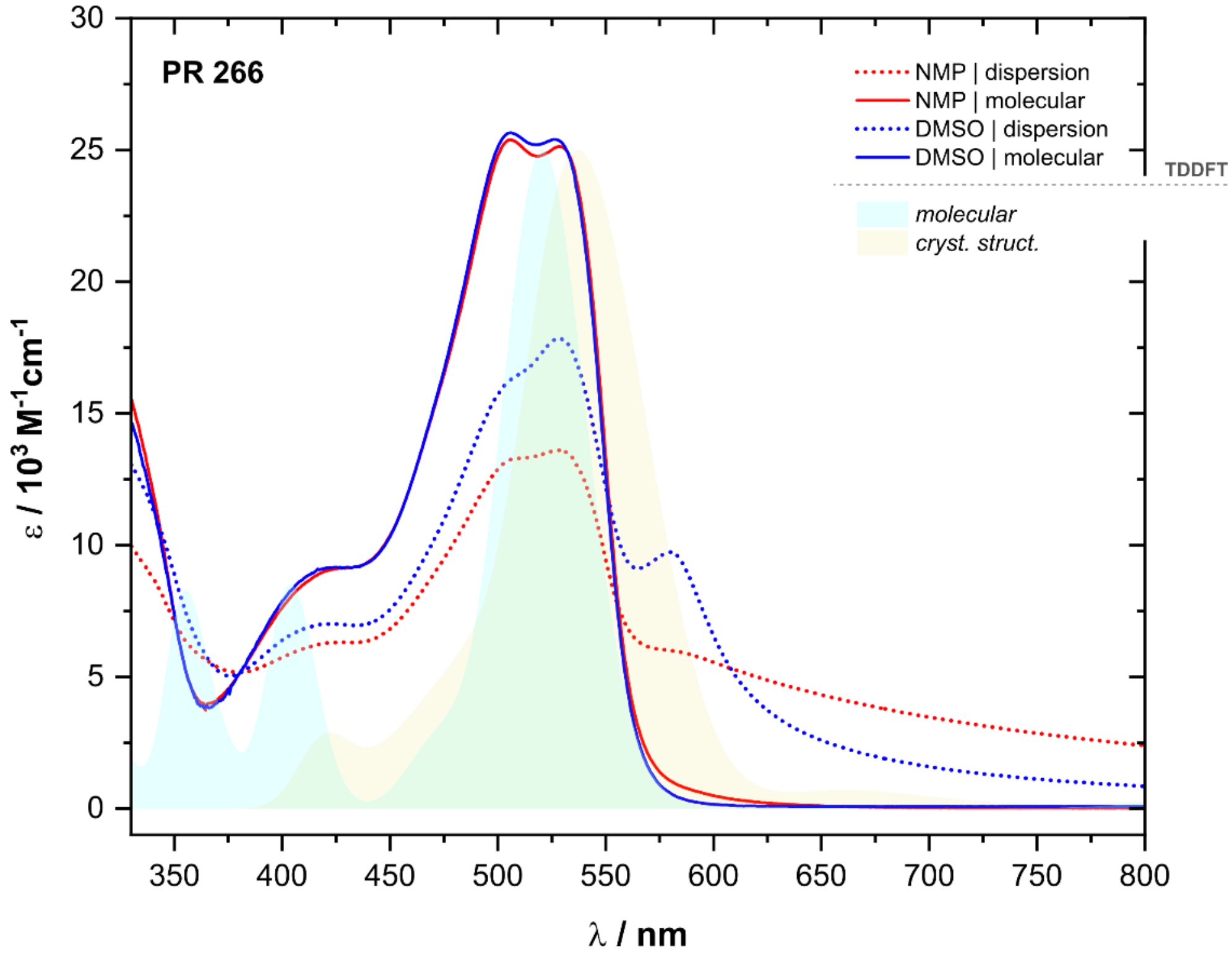


**Figure S10**. Final molecular UV-vis spectra of PR266 in NMP (at ca. 10 µg/mL) and DMSO (solid lines), contrasted with corresponding dispersion spectra and normalized TDDFT simulation. TDDFT simulations were shifted by -0.69 eV, respectively; peak type is Gaussian, 0.2 eV FWHM.

### 2.1.3 Pigment Red 22 (PR22)

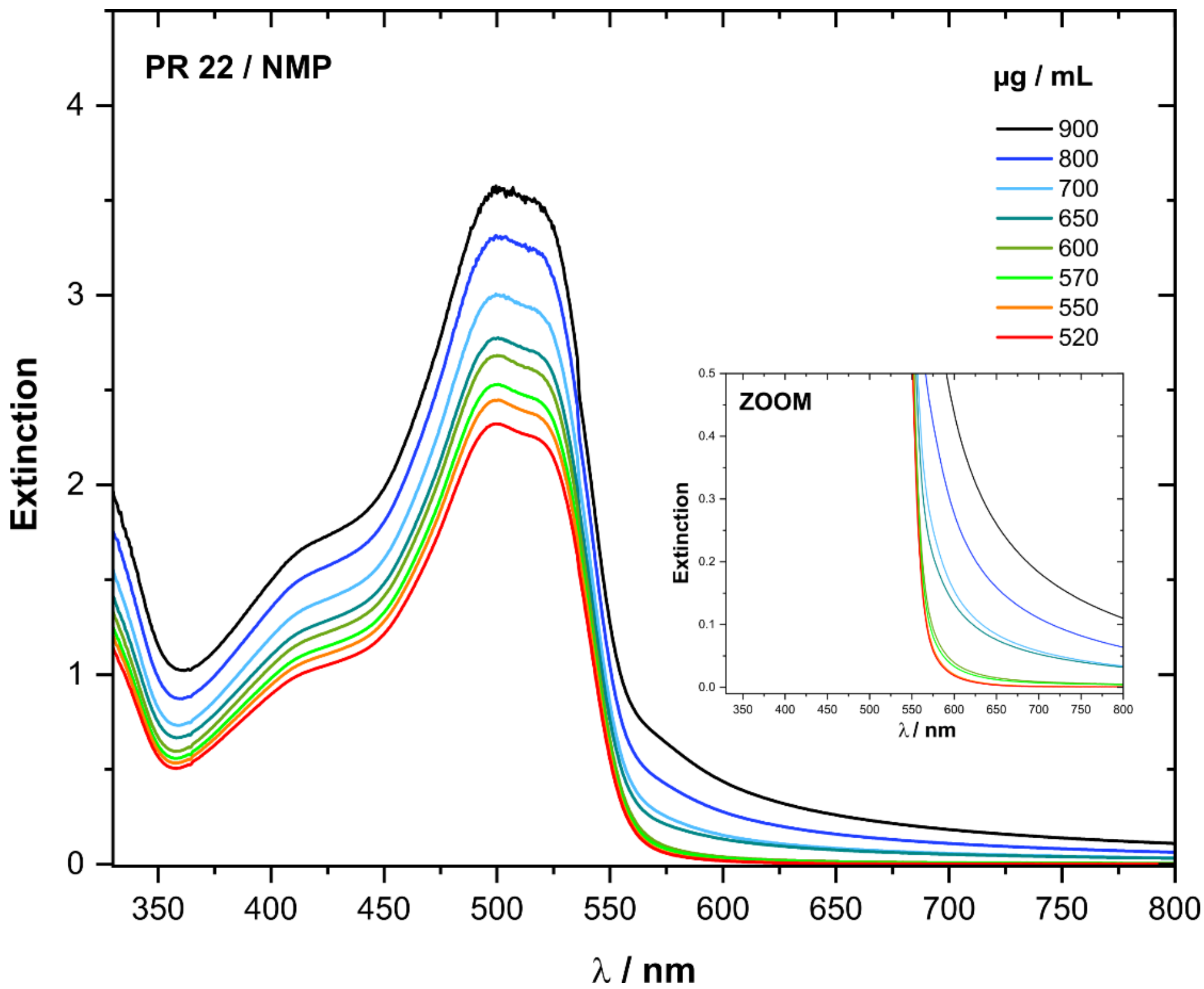


**Figure S11**. Iterative dilution procedure of PR22 in NMP, measured in a 0.1 cm quartz cuvette. The extinction exceeds the threshold of 1. The decline of the scattering signal was used as indicator for the transition point instead. The measurement sequence starts with a dispersion (900 µg/mL) and tracks the UV-vis spectral evolution down to 520 µg/mL.

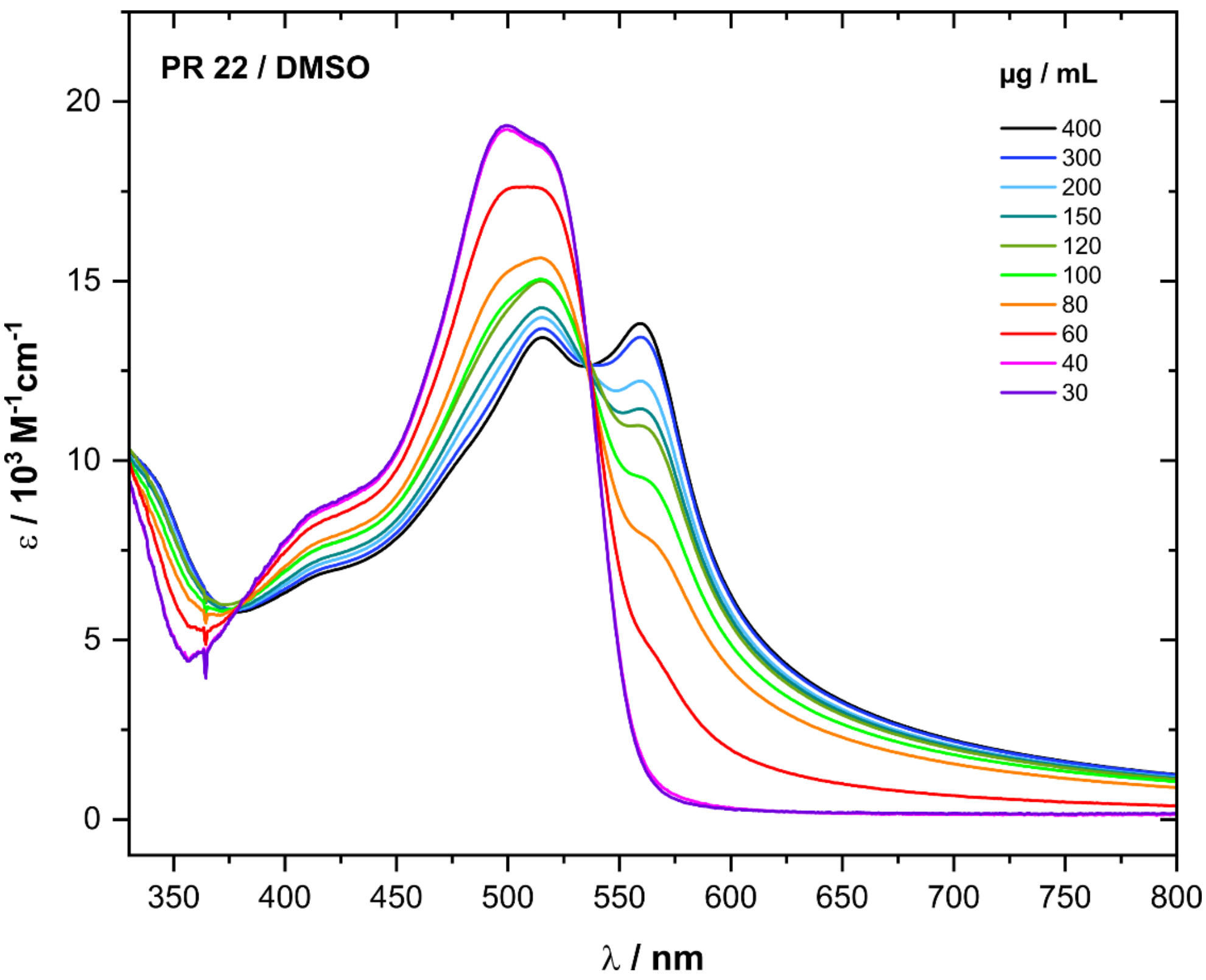


**Figure S12**. Iterative dilution procedure of PR22 in DMSO, measured in a 0.1 cm quartz cuvette. The measurement sequence starts with a dispersion (400 µg/mL) and tracks the UV-vis spectral evolution down to 30 µg/mL.

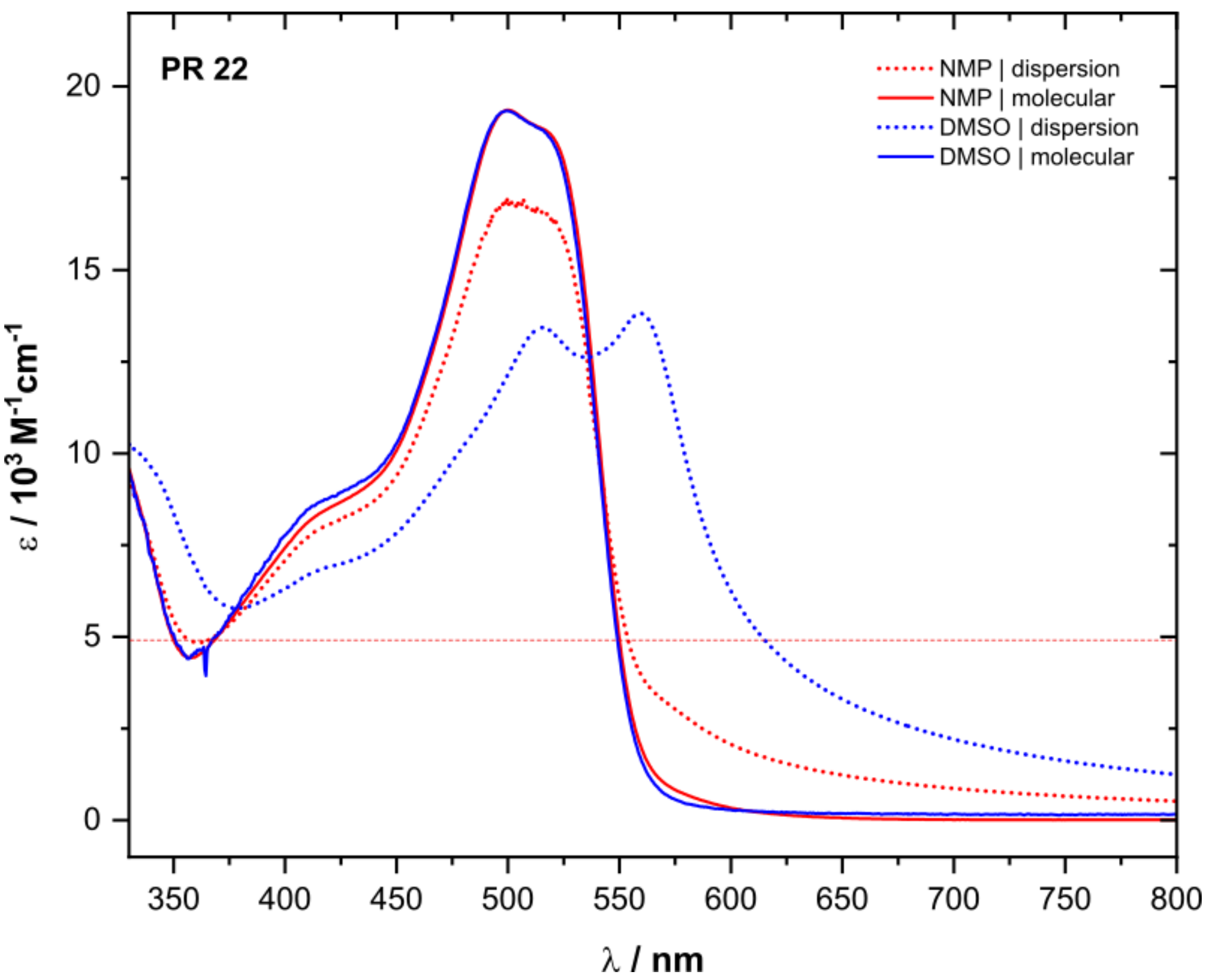


**Figure S13**. Final molecular UV-vis spectra of PR266 in NMP (at ca. 10 µg/mL) and DMSO (solid lines), contrasted with corresponding dispersion spectra. The dispersion spectrum in NMP exceeds the extinction of 1 above the dotted horizontal line. No available crystal structures were found for TDDFT simulations.

### 2.1.4 Pigment Yellow 151 (PY151)

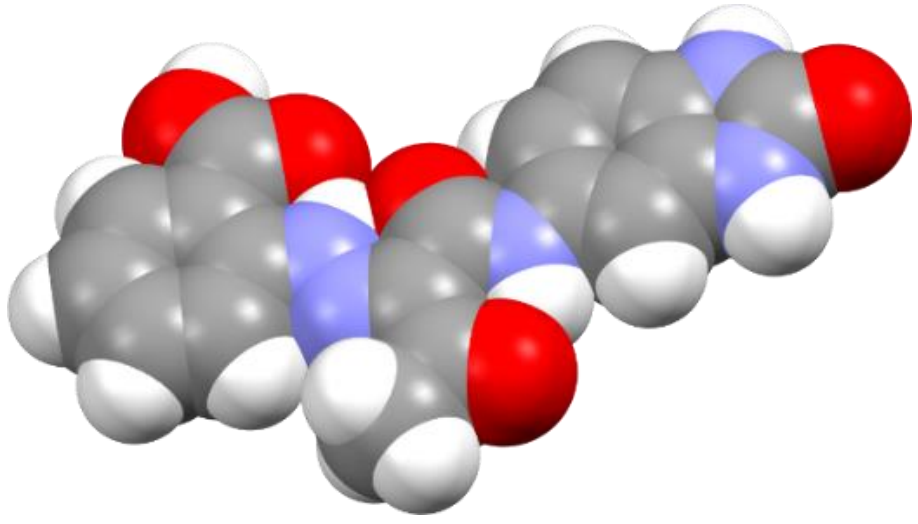

**Figure S14**. Molecular structure of PY151 after geometrical optimization in vacuo (DFT: r$^2$SCAN-3c).

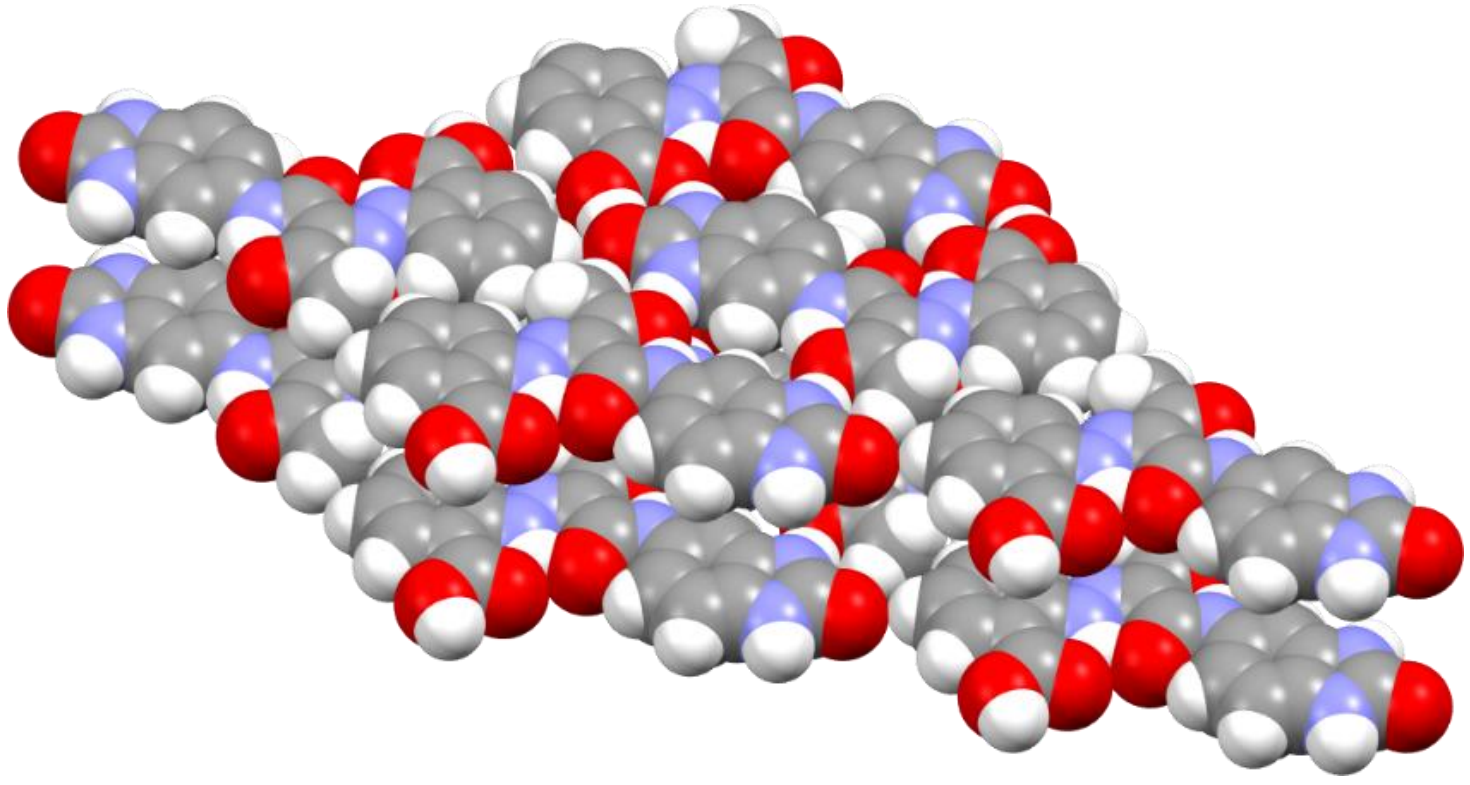

**Figure S15**. Pigment molecule ensemble (PY151) extracted from respective literature-known crystal structure: CCDC-737343.[10] Depicted cut-out (9 pigment molecules) was used for TDDFT simulation, see Computational Details.

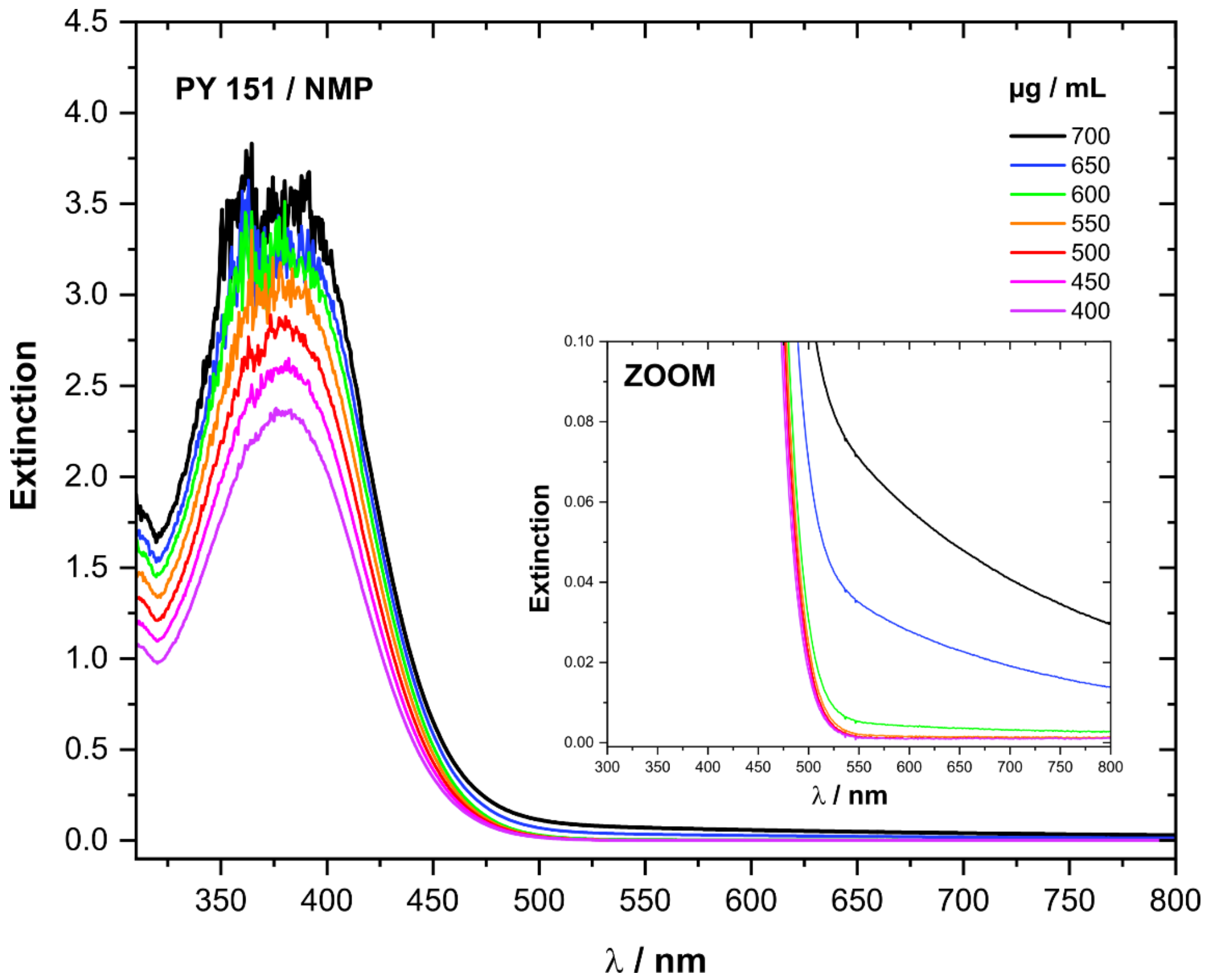


**Figure S16**. Iterative dilution procedure of PY151 in NMP, measured in a 0.1 cm quartz cuvette. The extinction exceeds the threshold of 1. The decline of the scattering signal was used as indicator for the transition point instead. The measurement sequence starts with a dispersion (700 µg/mL) and tracks the UV-vis spectral evolution down to 400 µg/mL.

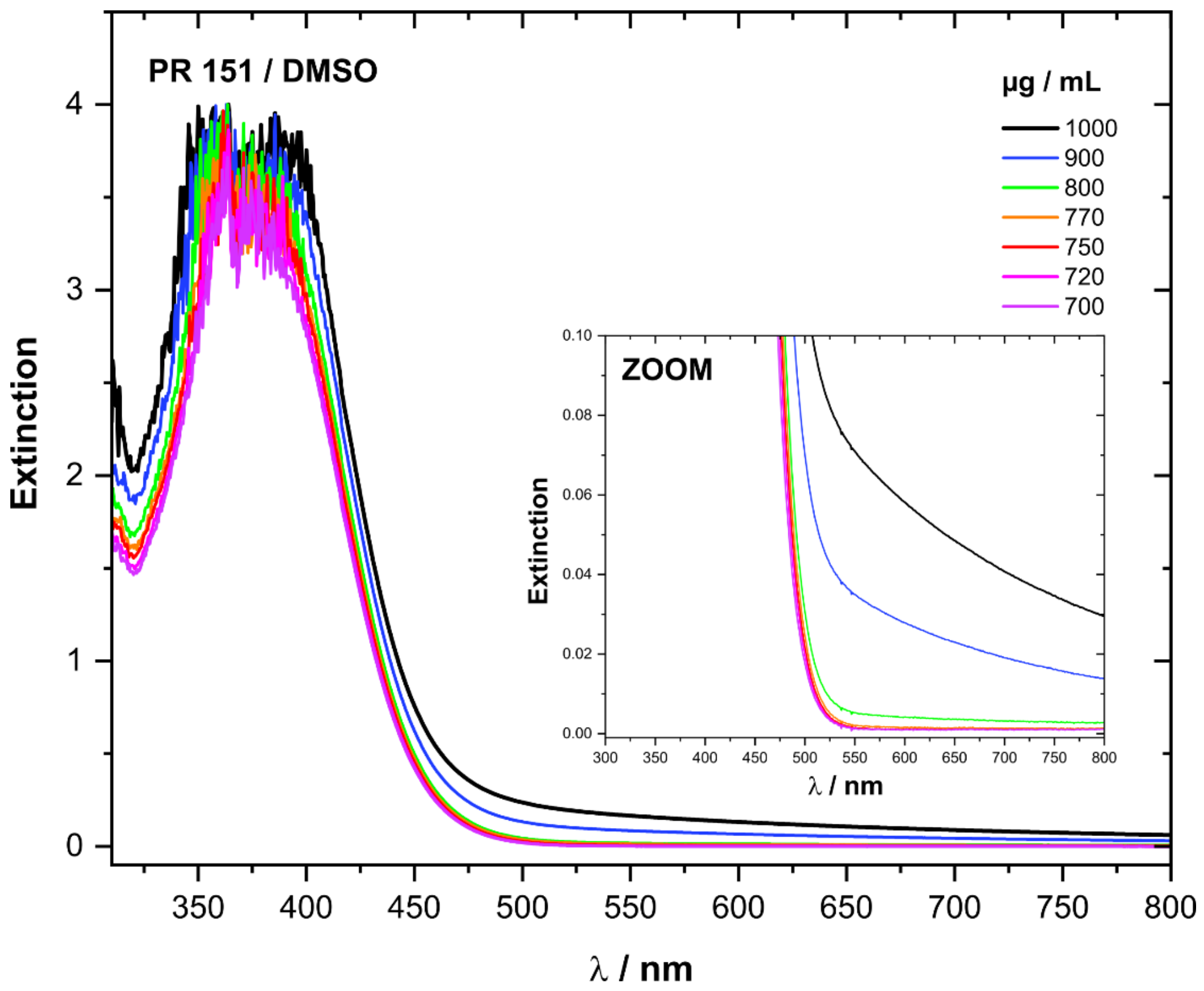


**Figure S17**. Iterative dilution procedure of PY151 in DMSO, measured in a 0.1 cm quartz cuvette. The extinction exceeds the threshold of 1. The decline of the scattering signal was used as indicator for the transition point instead. The measurement sequence starts with a dispersion (1000 µg/mL) and tracks the UV-vis spectral evolution down to 700 µg/mL.

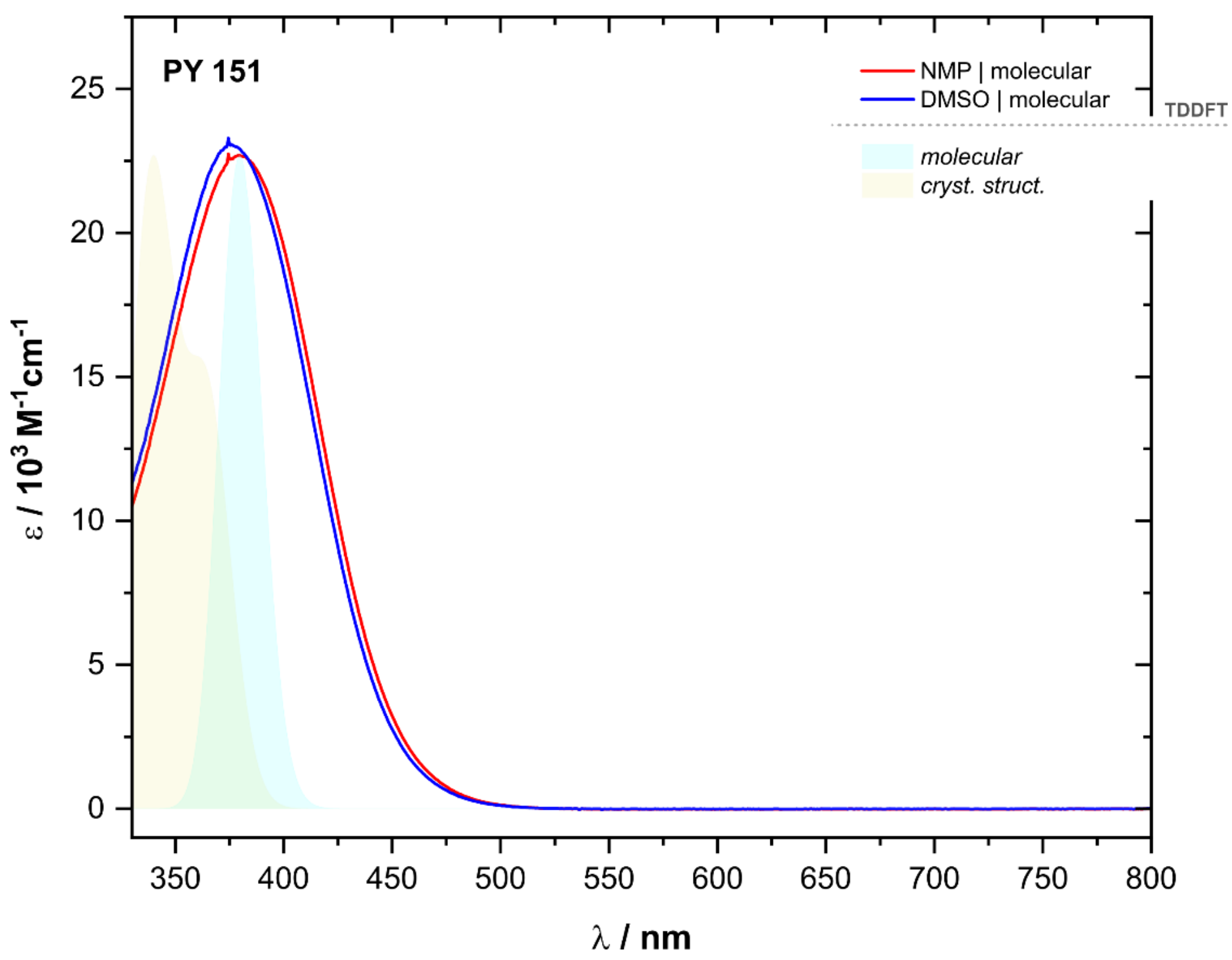


**Figure S18**. Final molecular UV-vis spectra of PY151 in NMP and DMSO (solid lines) at ca. 10 µg/mL, contrasted with normalized TDDFT simulation. TDDFT simulations were shifted by +0.54 eV, respectively; peak type is Gaussian, 0.2 eV FWHM.

## 2.2 Dihydrazone (Disazo) Pigment Class

### 2.2.1 Pigment Orange 13 (PO13)

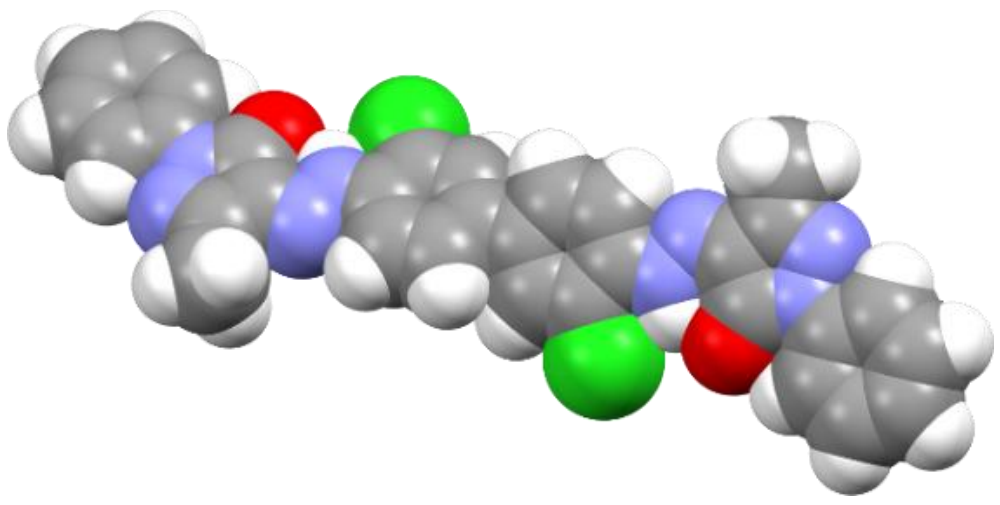

**Figure S19**. Molecular structure of PO13 after geometrical optimization in vacuo (DFT: $r^2$SCAN-3c).

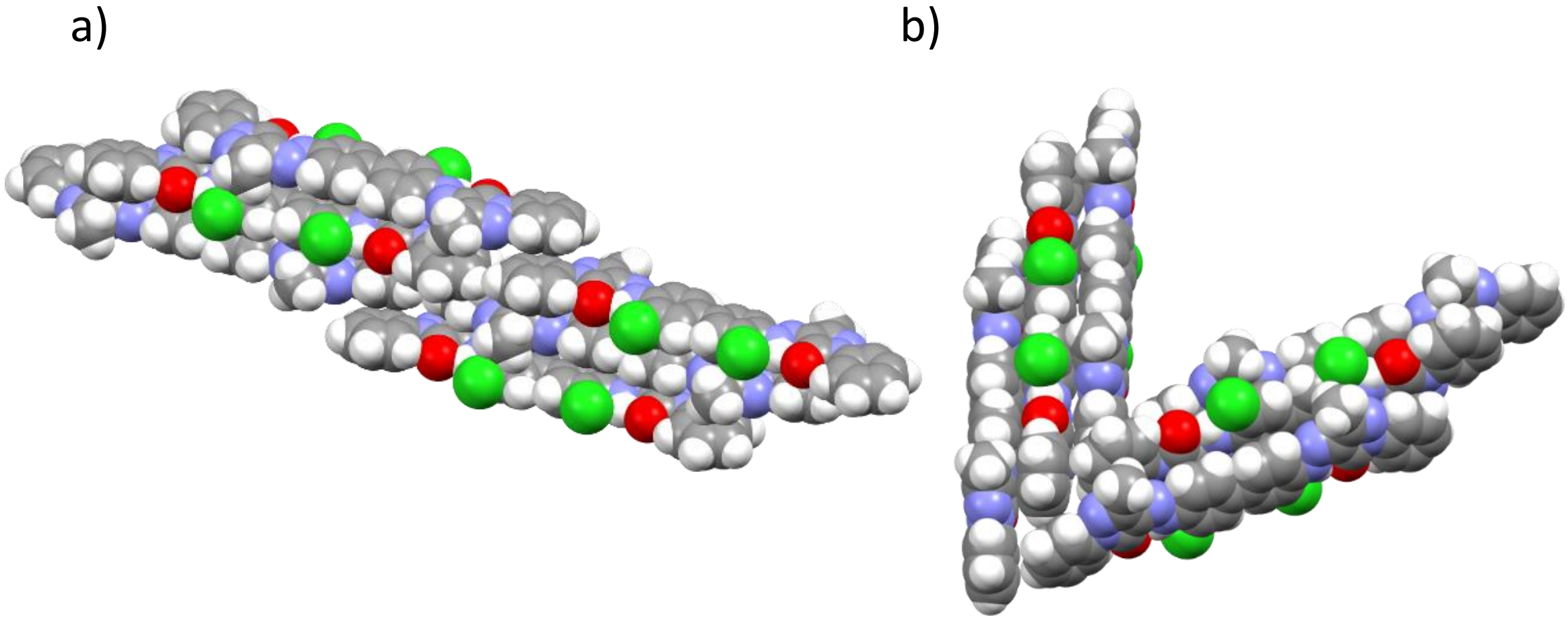


**Figure S20**. Pigment molecule ensembles (PO13) extracted from respective literature-known crystal structures, a) *beta* structure: CCDC-2160372,[11] b) *gamma*$_1$ structure: CCDC-2160371.[11] Depicted cut-outs (6 pigment molecules each) were used for TDDFT simulations, see Computational Details.

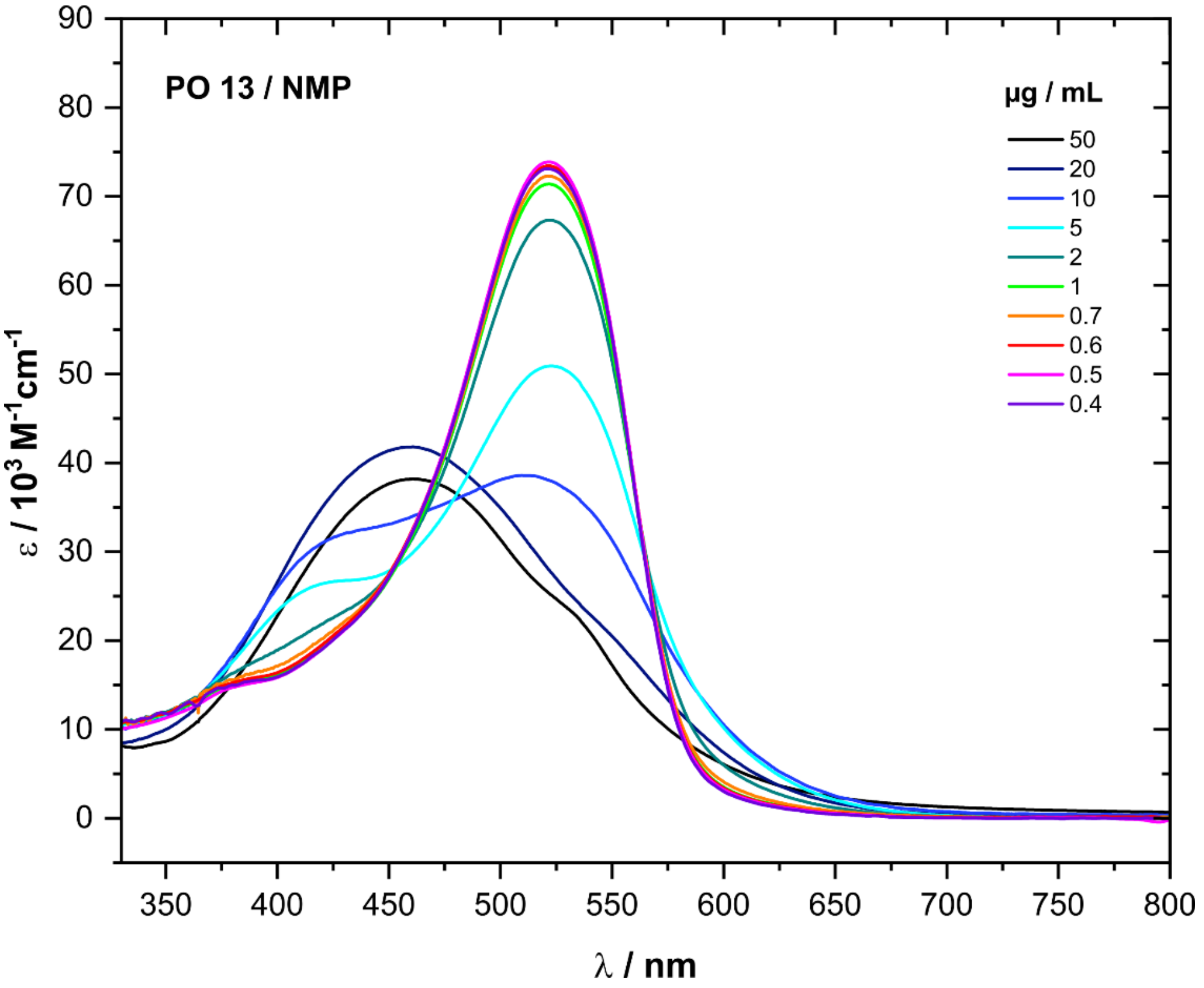


**Figure S21**. Iterative dilution procedure of PO13 in NMP, measured in 0.5 and 2 cm quartz cuvettes. The measurement sequence starts with a dispersion (50 µg/mL) and tracks the UV-vis spectral evolution down to 0.4 µg/mL.

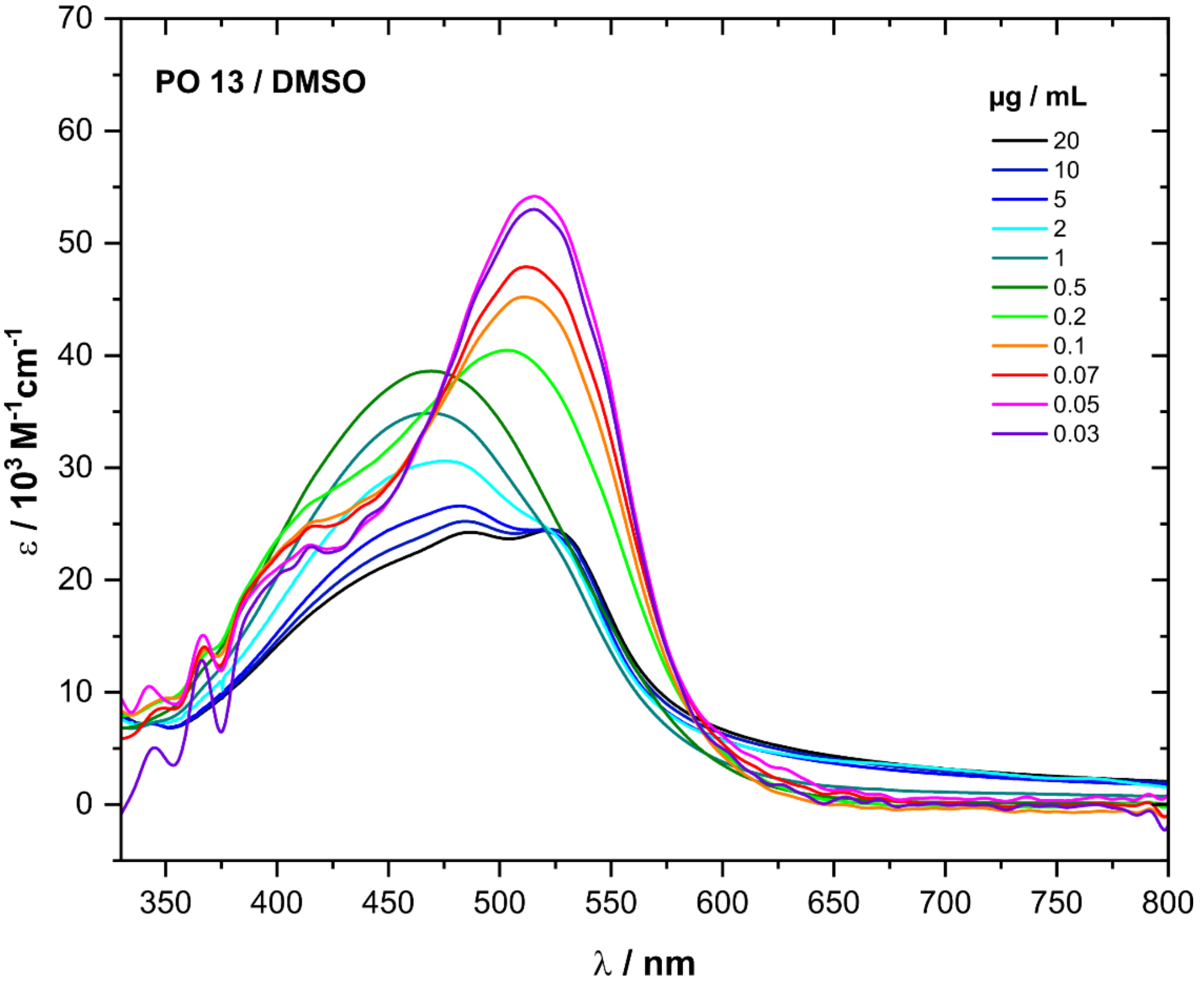


**Figure S22**. Iterative dilution procedure of PO13 in DMSO, measured in 2 cm quartz cuvette. The measurement sequence starts with a dispersion (20 µg/mL) and tracks the UV-vis spectral evolution down to 0.4 µg/mL. Due to the low concentrations the sample reaches the instrumental detection limits before the transition point. Solubility could only be determined as an upper bound (<0.03 µg/mL).

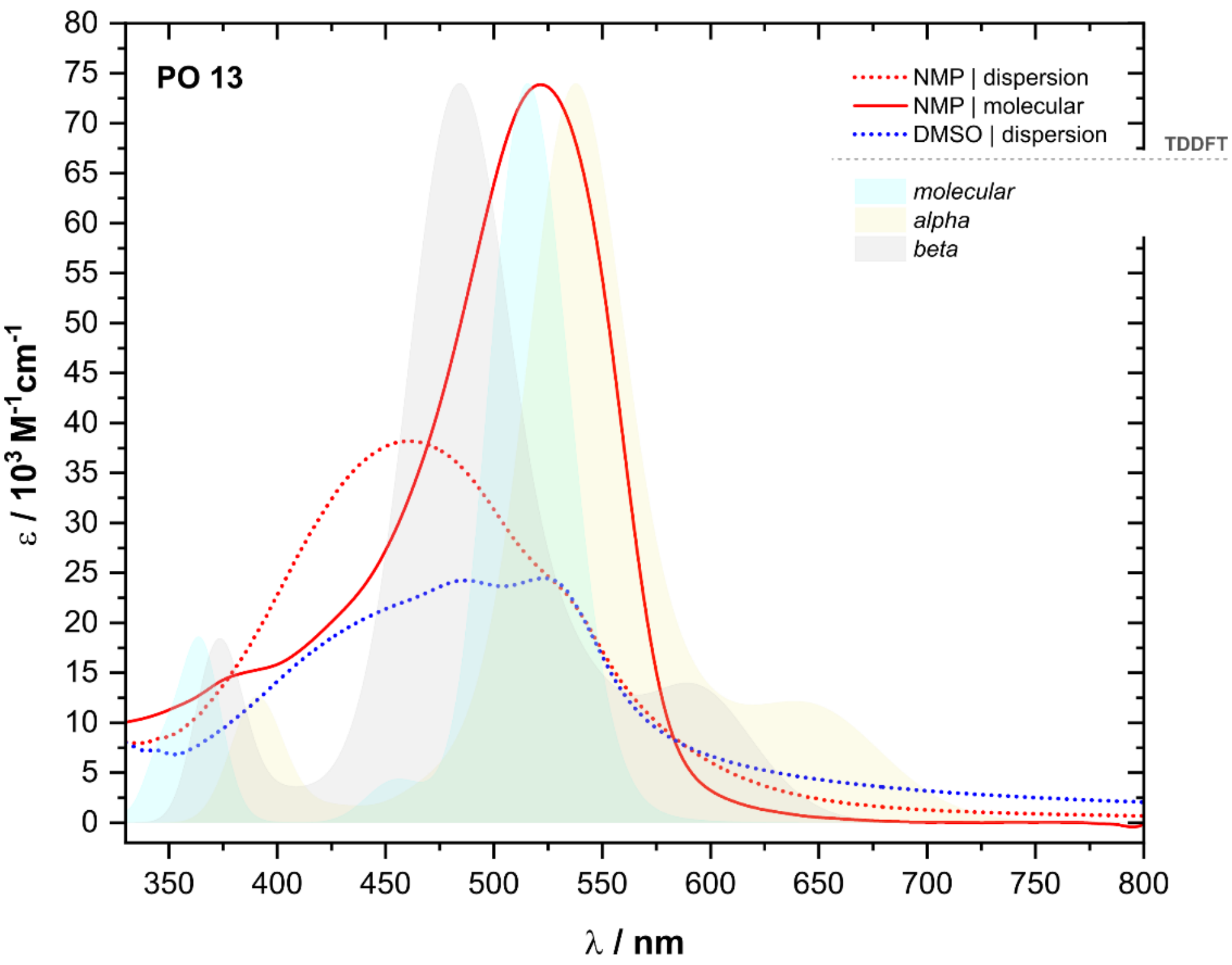


**Figure S23**. Final molecular UV-vis spectrum of PO13 in NMP (solid line), contrasted with corresponding dispersion spectra and normalized TDDFT simulations. TDDFT simulations were shifted by -0.58 eV, respectively; peak type is Gaussian, 0.2 eV FWHM.

### 2.2.2 Pigment Yellow 14 (PY14)

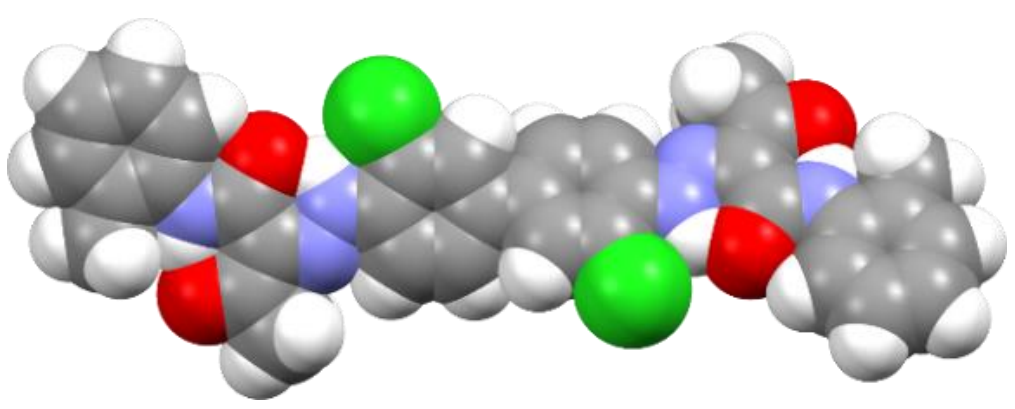

**Figure S24**. Molecular structure of PY14 after geometrical optimization in vacuo (DFT: $r^2$SCAN-3c).

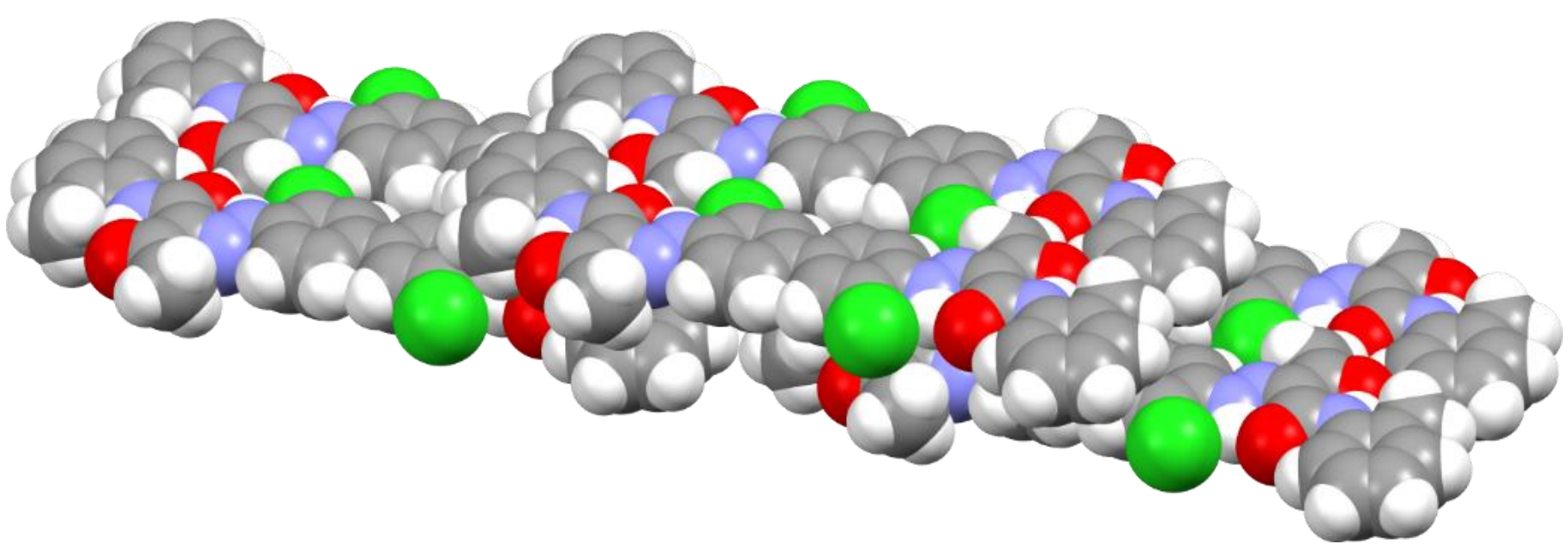

**Figure S25**. Pigment molecule ensemble (PY14) extracted from respective literature-known crystal structure: CCDC-170804.[12] Depicted cut-out (6 pigment molecules) was used for TDDFT simulation, see Computational Details.

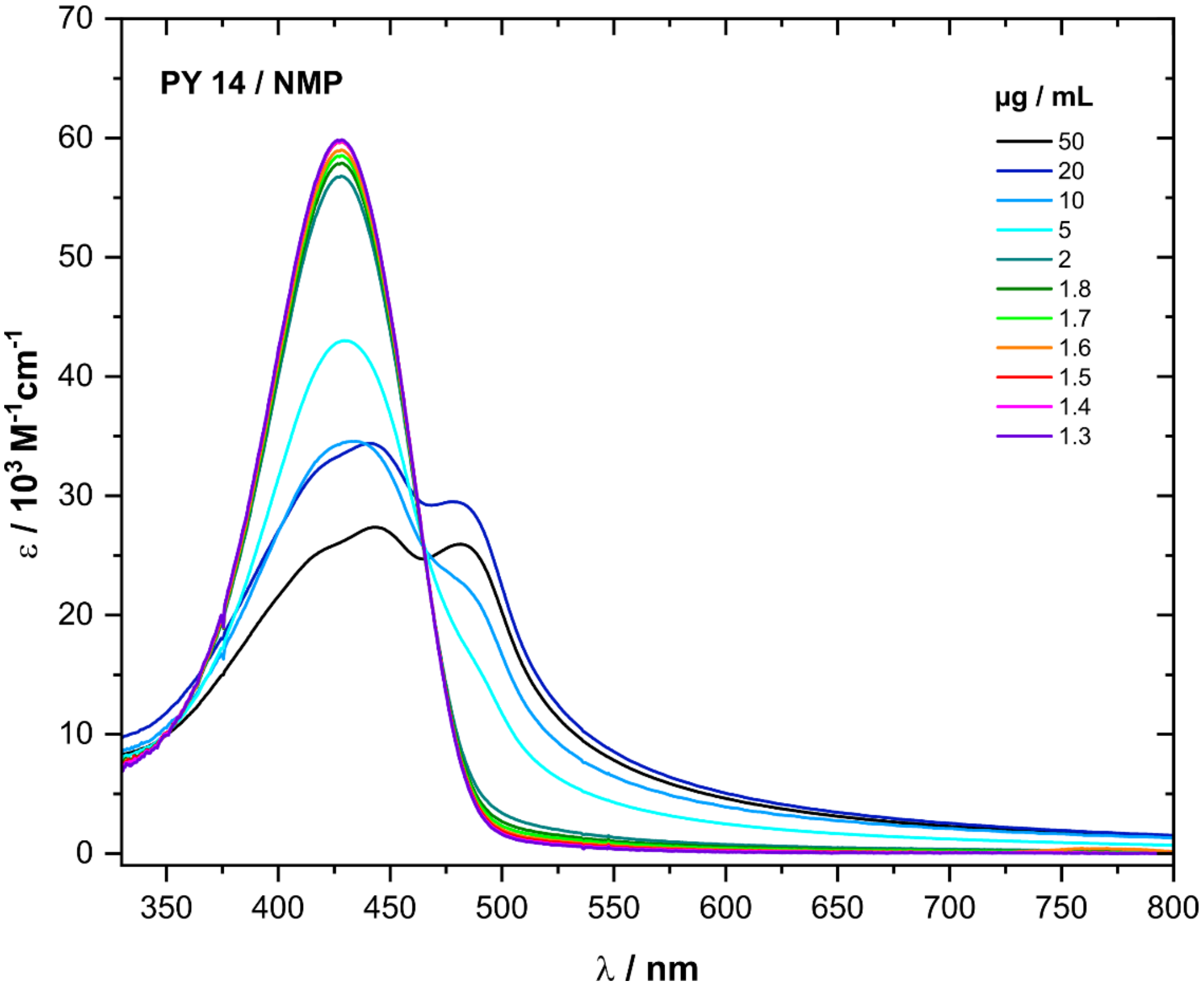


**Figure S26**. Iterative dilution procedure of PY14 in NMP, measured in 0.5 and 2 cm quartz cuvettes. The measurement sequence starts with a dispersion (50 µg/mL) and tracks the UV-vis spectral evolution down to 1.3 µg/mL.

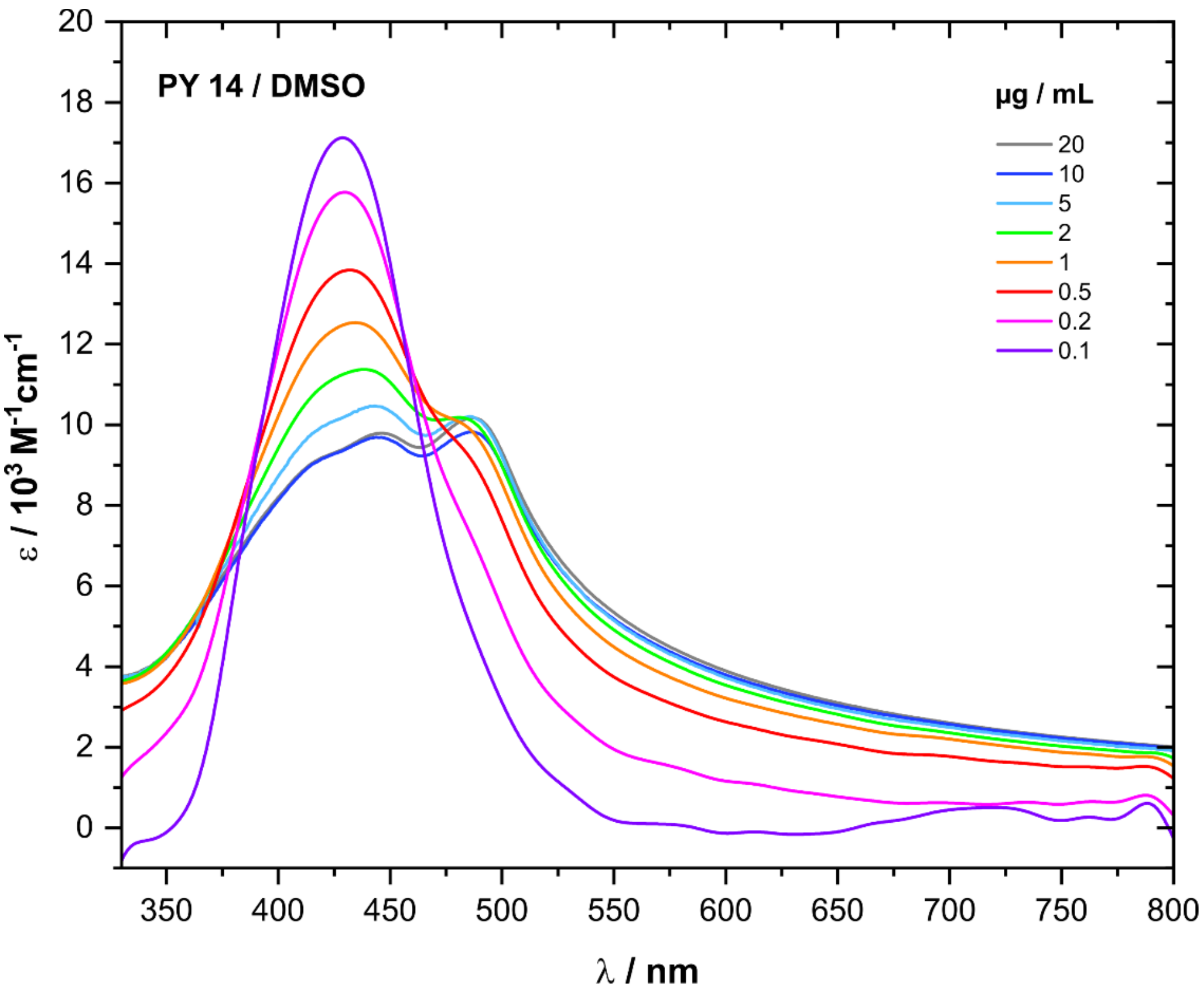


**Figure S27**. Iterative dilution procedure of PY14 in DMSO, measured in 2 cm quartz cuvette. The measurement sequence starts with a dispersion (20 µg/mL) and tracks the UV-vis spectral evolution down to 0.1 µg/mL. Due to the low concentrations the sample reaches the instrumental detection limits before the transition point. Solubility could only be determined as an upper bound (<0.1 µg/mL).

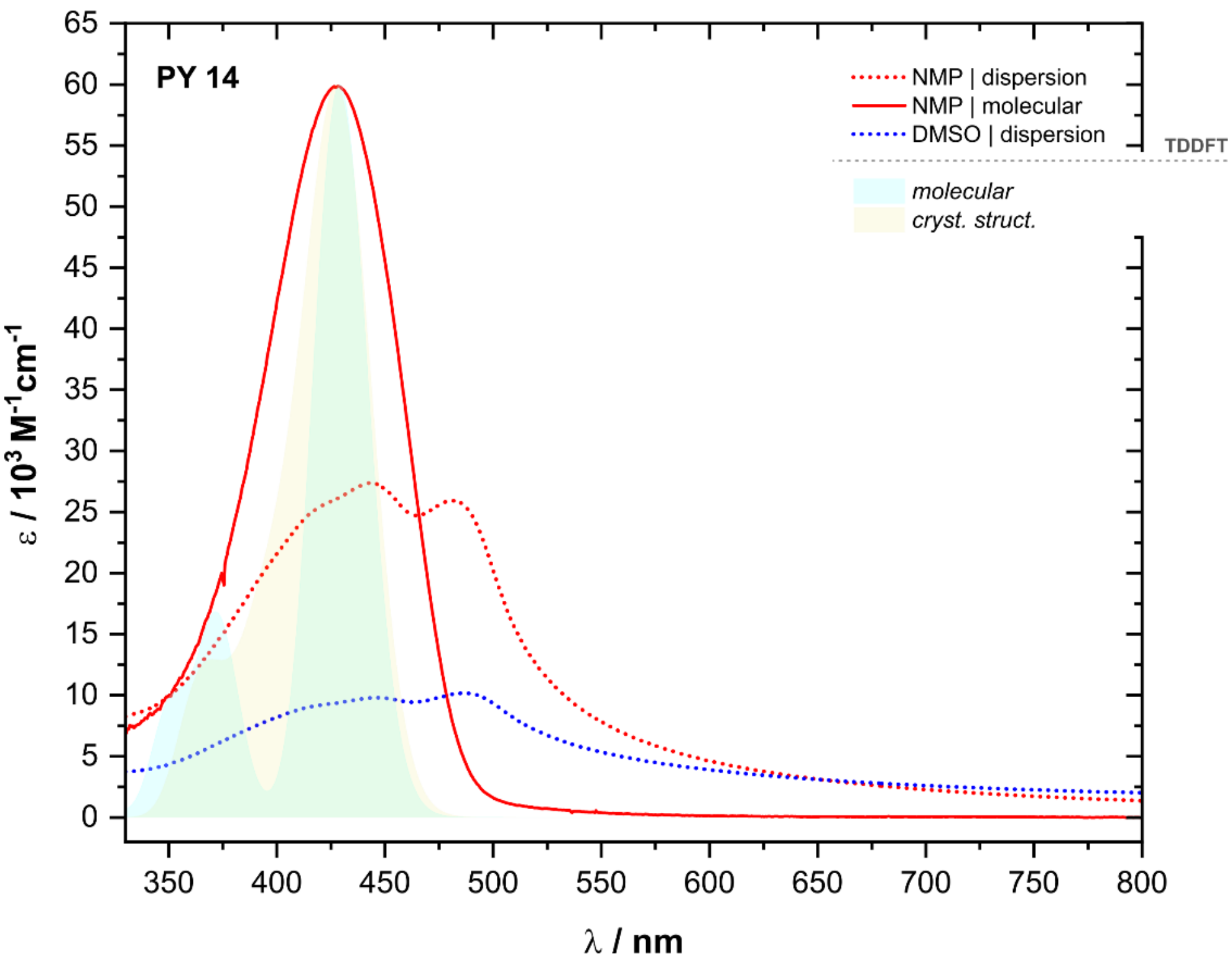


**Figure S28**. Final molecular UV-vis spectrum of PY14 in NMP (solid line), contrasted with corresponding dispersion spectra and normalized TDDFT simulations. TDDFT simulations were shifted by -0.05 eV, respectively; peak type is Gaussian, 0.2 eV FWHM.

### 2.2.3 Pigment Yellow 83 (PY83)

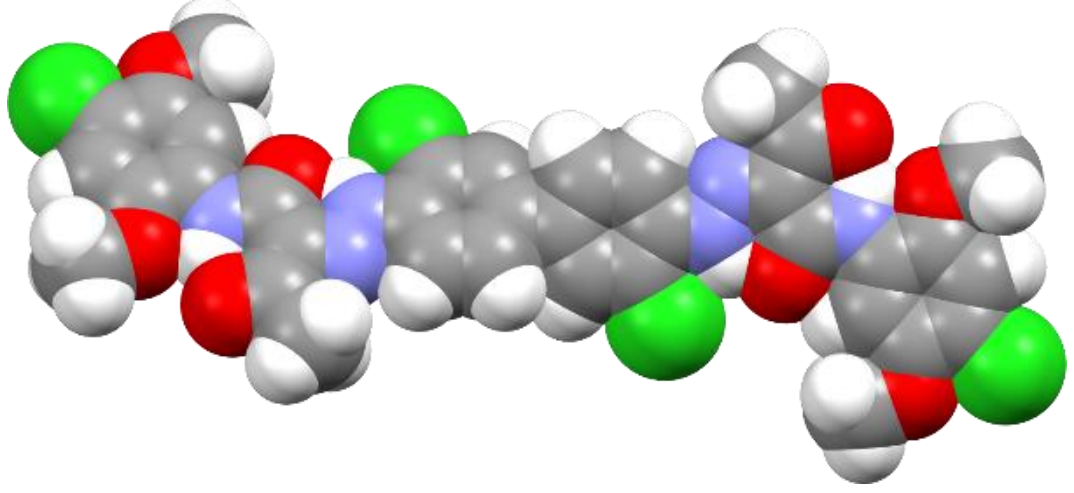

**Figure S29**. Molecular structure of PY83 after geometrical optimization in vacuo (DFT: r$^2$SCAN-3c).

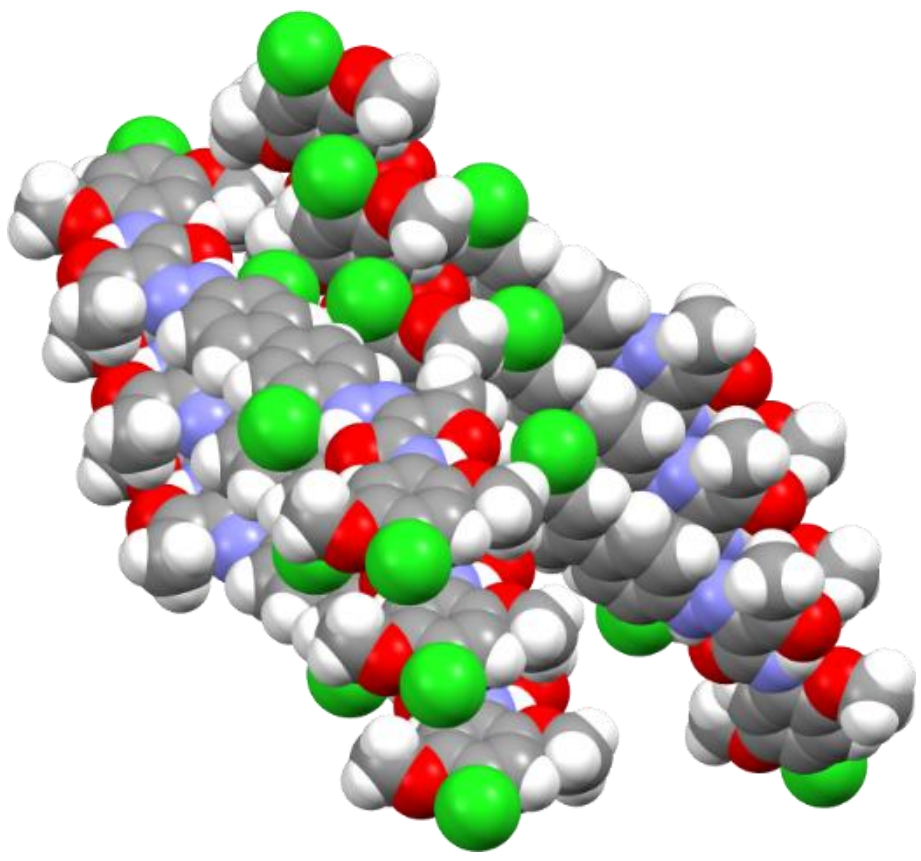

**Figure S30**. Pigment molecule ensemble (PY83) extracted from respective literature-known crystal structure: CCDC-170805.[12] Depicted cut-out (6 pigment molecules) was used for TDDFT simulation, see Computational Details.

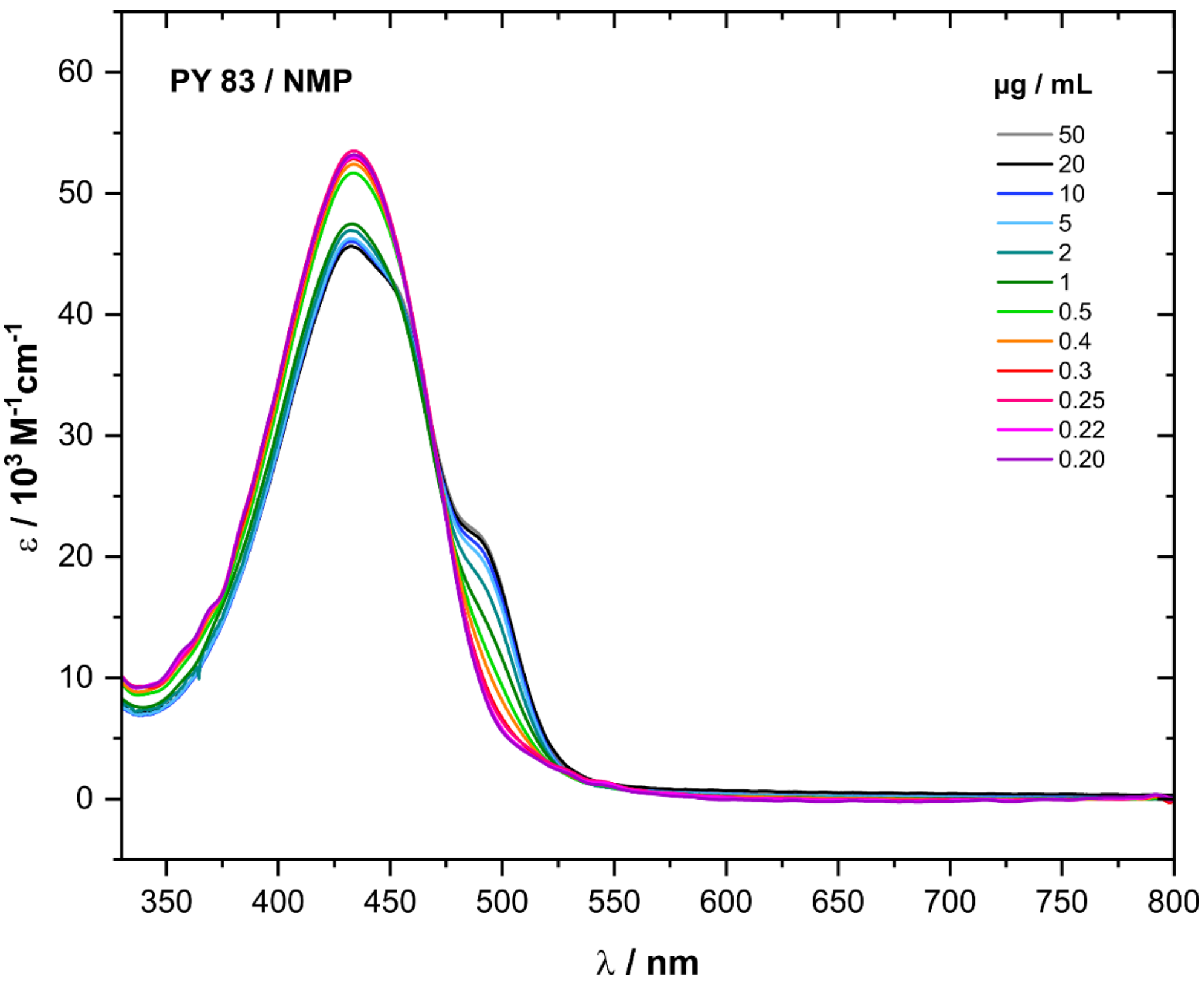


**Figure S31**. Iterative dilution procedure of PY83 in NMP, measured in 0.5 and 2 cm quartz cuvettes. The measurement sequence starts with a dispersion (50 µg/mL) and tracks the UV-vis spectral evolution down to 0.20 µg/mL.

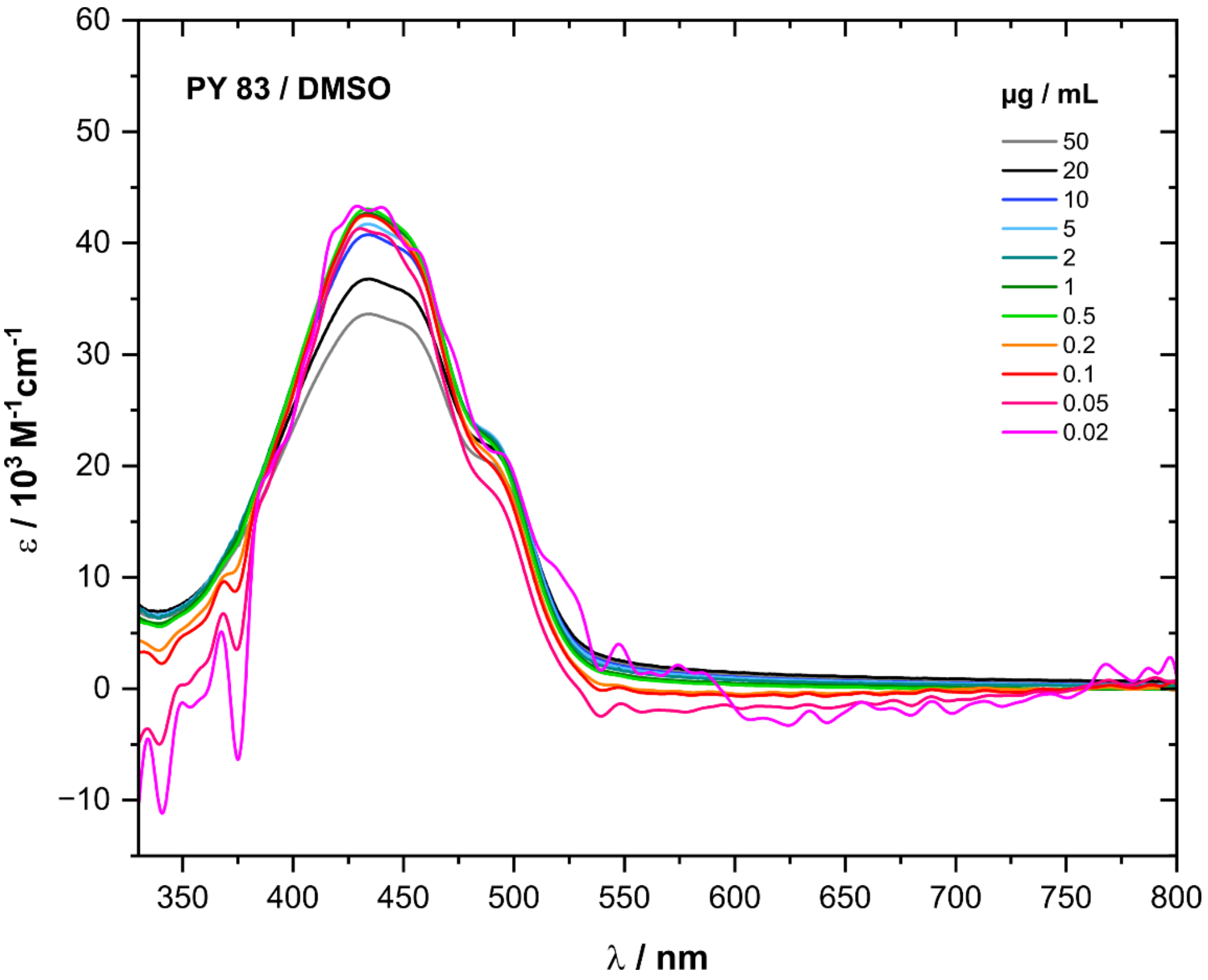


**Figure S32**. Iterative dilution procedure of PY83 in DMSO, measured in 2 cm quartz cuvette. The measurement sequence starts with a dispersion (50 µg/mL) and tracks the UV-vis spectral evolution down to 0.02 µg/mL. Due to the low concentrations the sample reaches the instrumental detection limits before the transition point. Solubility could only be determined as an upper bound (<0.02 µg/mL).

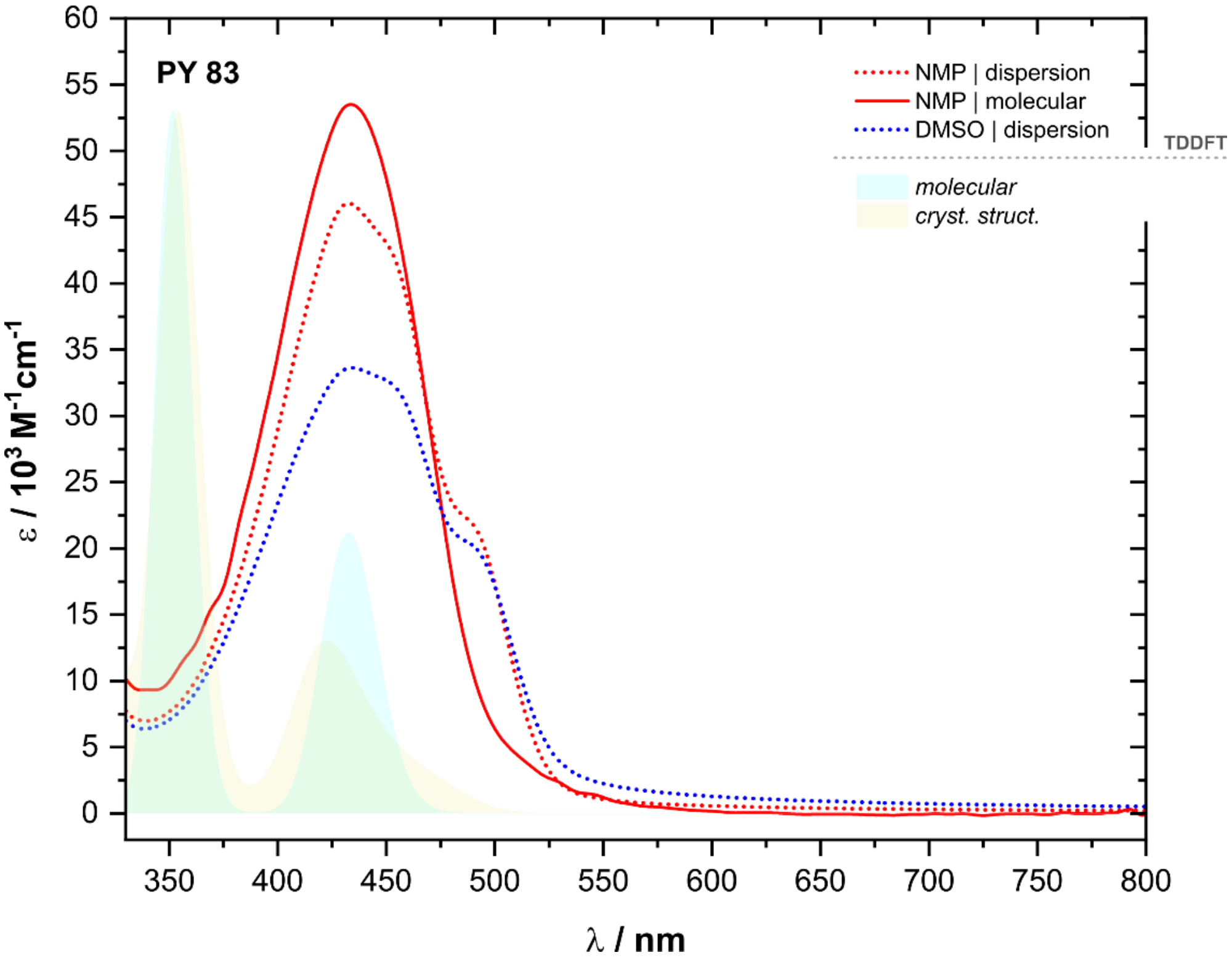


**Figure S33**. Final molecular UV-vis spectrum of PY83 in NMP (solid line), contrasted with corresponding dispersion spectra and normalized TDDFT simulations. TDDFT simulations were shifted by +0.32 eV, respectively; peak type is Gaussian, 0.2 eV FWHM.

## 2.3 Quinacridone Pigment Class

### 2.3.1 Pigment Violet 19 (PV19)

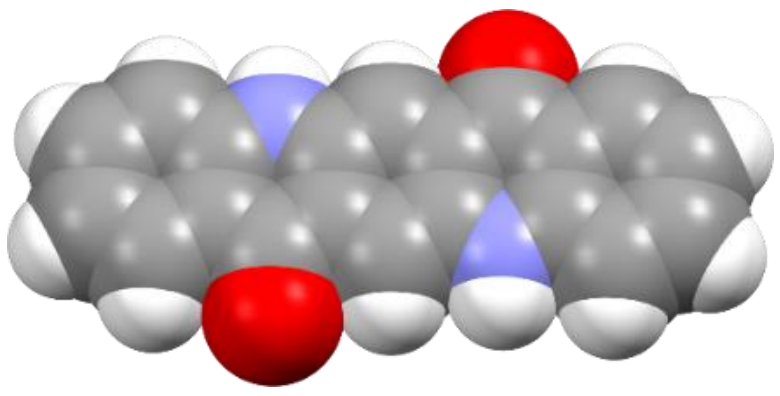

**Figure S34**. Molecular structure of PV19 after geometrical optimization in vacuo (DFT: r$^2$SCAN-3c).

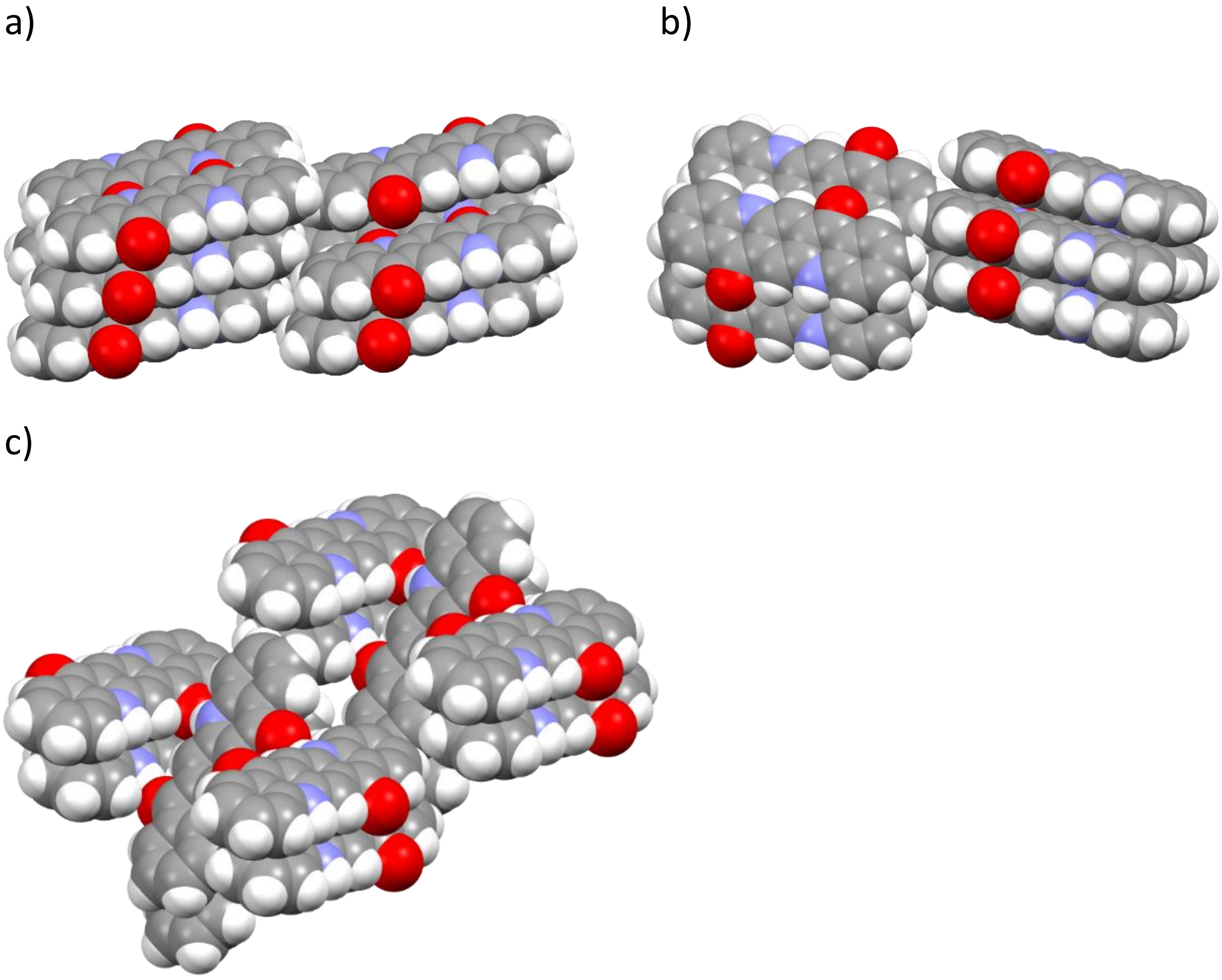


**Figure S35**. Pigment molecule ensembles (PV19) extracted from respective literature-known crystal structures, a) *alpha$_1$* structure: CCDC-620257, b) *beta* structure: CCDC-620258, c) *gamma* structure CCDC-620259.[13] Depicted cut-outs (10 pigment molecules for *alpha$_1$*, 8 for *beta*, 12 for *gamma*) were used for TDDFT simulations, see Computational Details.

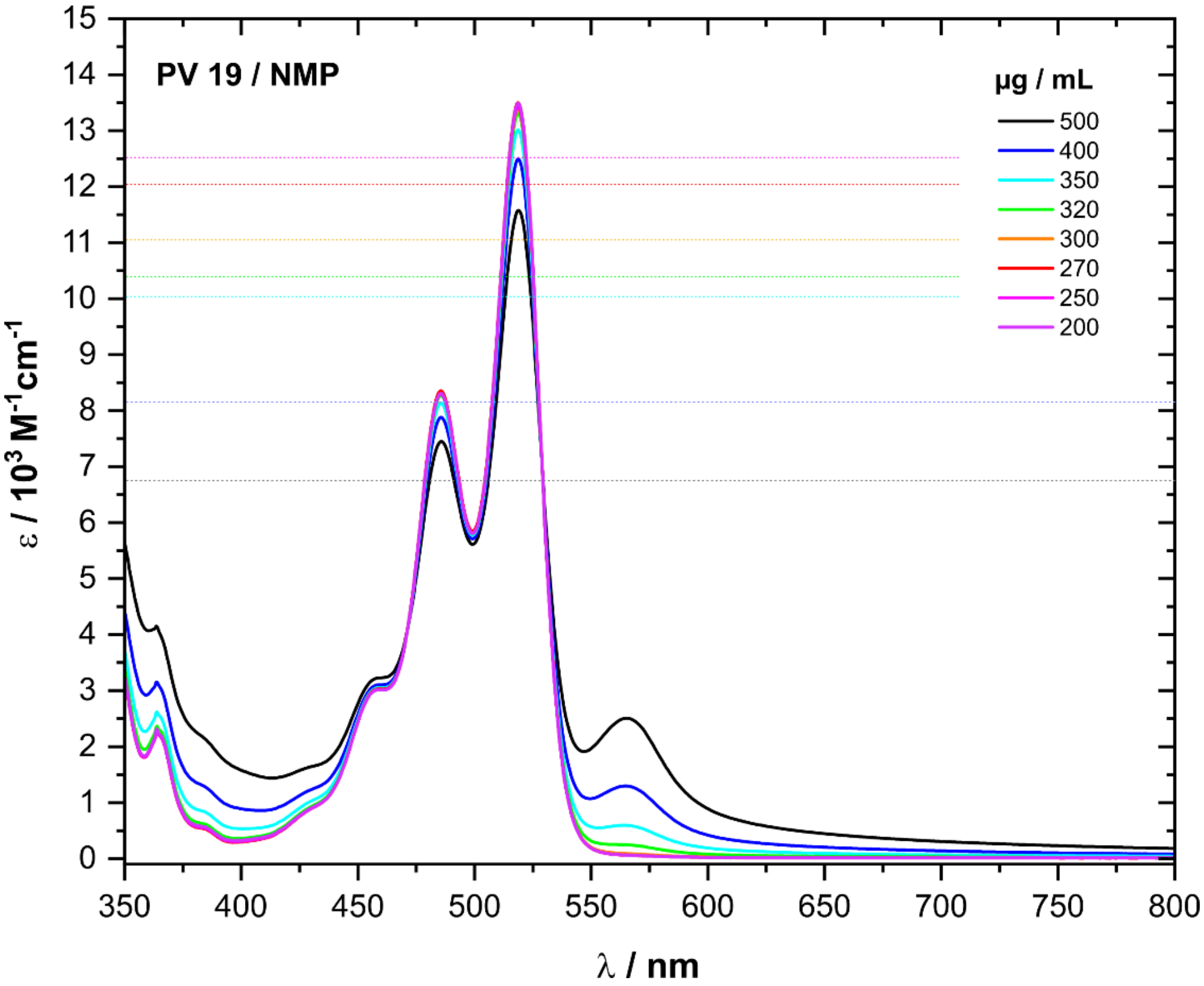


**Figure S36**. Iterative dilution procedure of PV19 in NMP, measured in 0.1 quartz cuvettes. The measurement sequence starts with a dispersion (500 µg/mL) and tracks the UV-vis spectral evolution down to 200 µg/mL. Horizontal lines indicate the instrumental extinction limit of 1 for each concentration. Spectral data points above that limit should be used with caution.

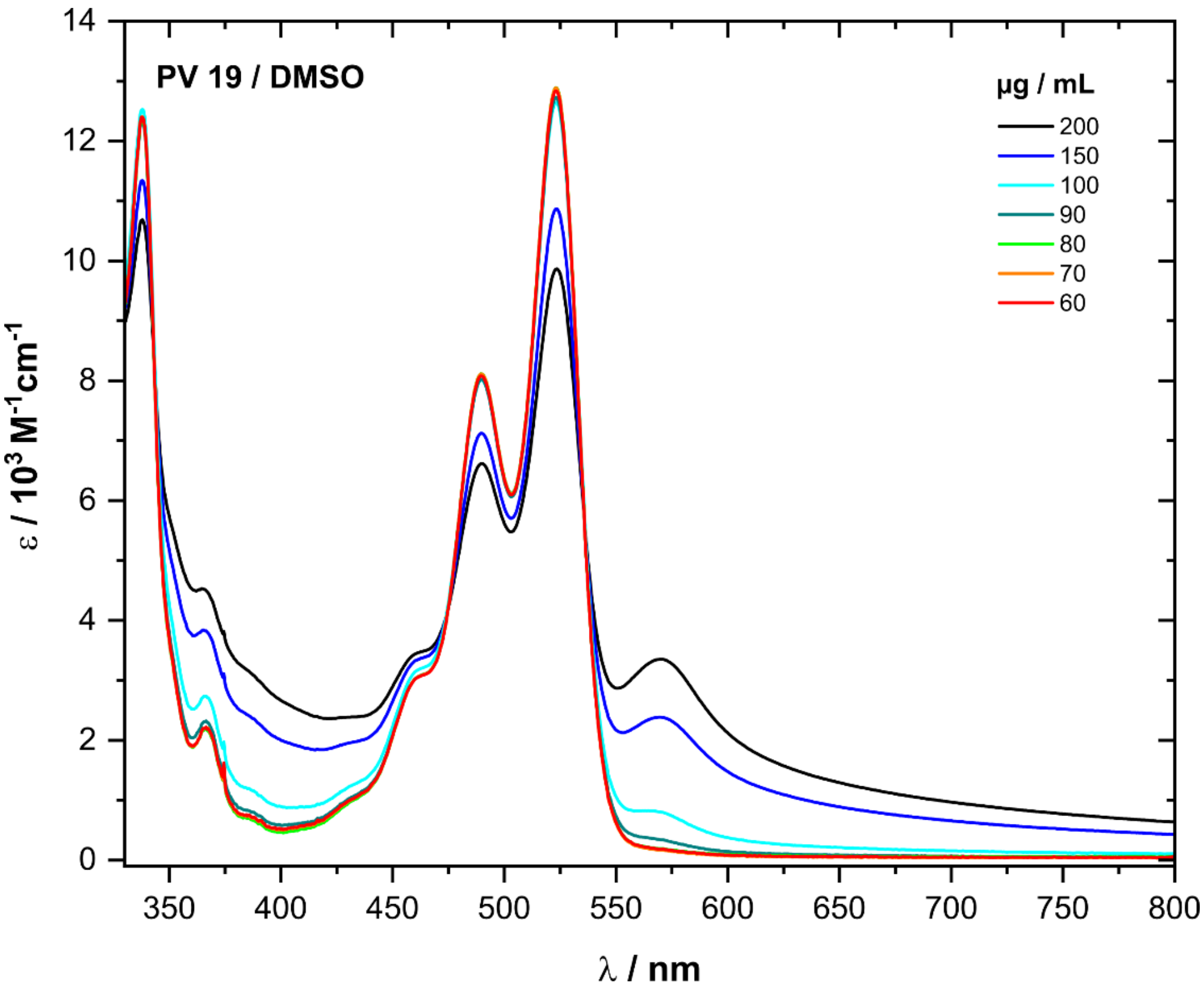


**Figure S37**. Iterative dilution procedure of PV19 in DMSO, measured in 0.1 and 0.5 cm quartz cuvettes. The measurement sequence starts with a dispersion (200 µg/mL) and tracks the UV-vis spectral evolution down to 60 µg/mL.

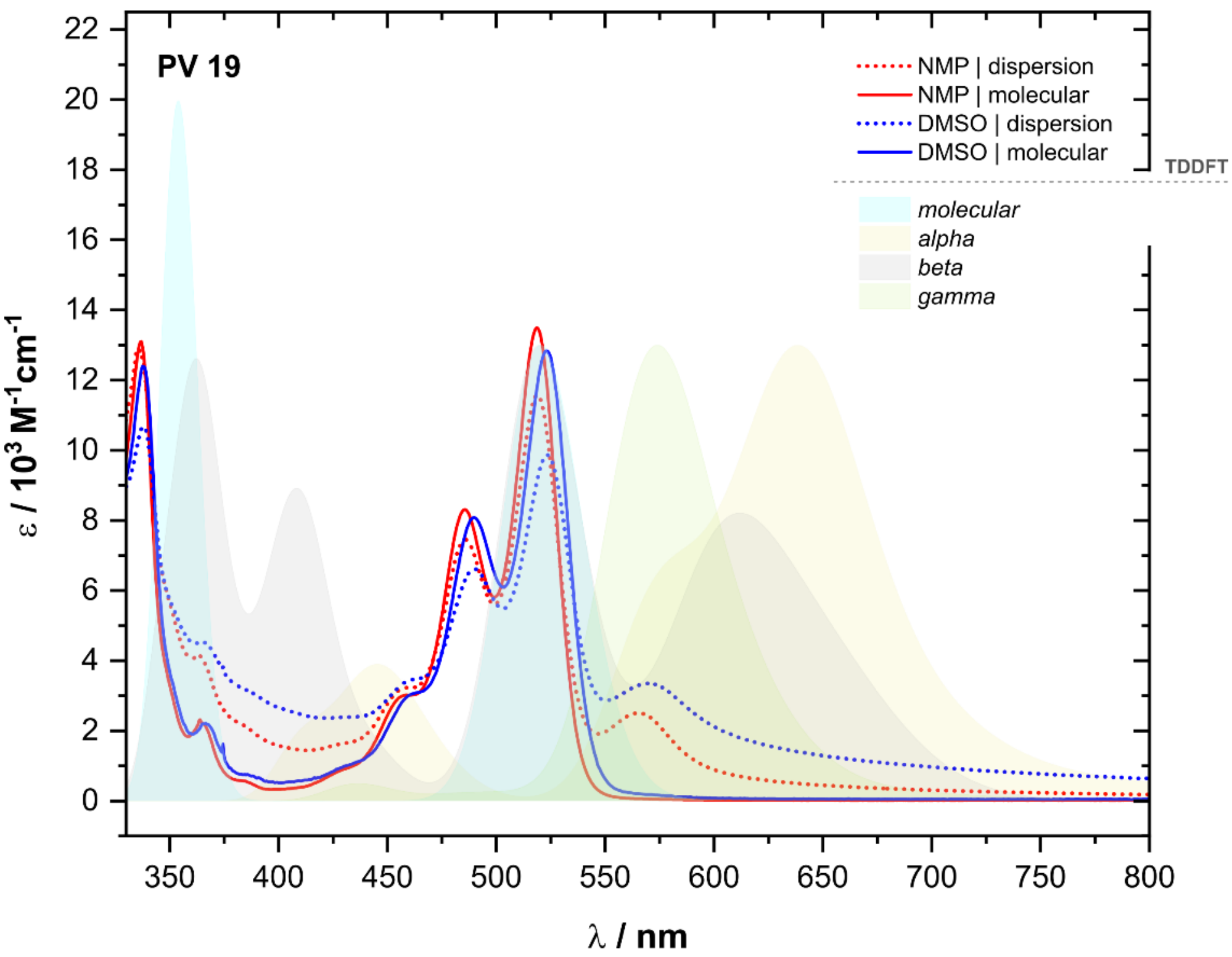


**Figure S38**. Final molecular UV-vis spectrum of PV19 in NMP and DMSO (solid lines) at ca. 10 µg/mL, contrasted with corresponding dispersion spectra and normalized TDDFT simulations. TDDFT simulations were shifted by -0.63eV, respectively; peak type is Gaussian, 0.2eV FWHM.

### 2.3.2 Pigment Red 122 (PR122)

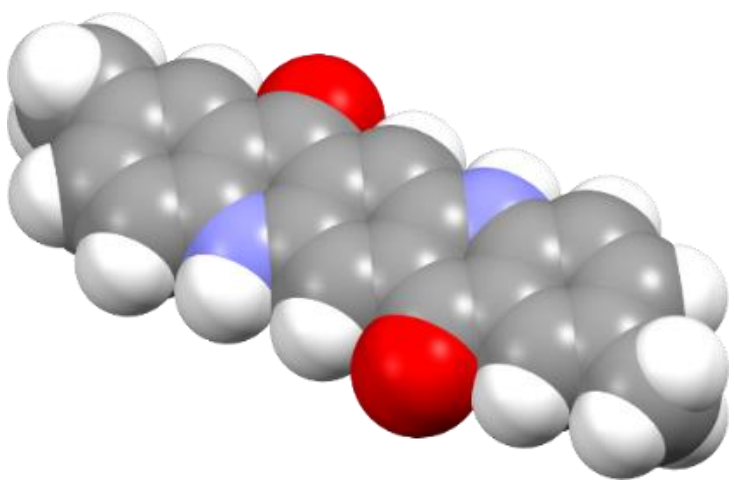

**Figure S39**. Molecular structure of PR122 after geometrical optimization in vacuo (DFT: r$^2$SCAN-3c).

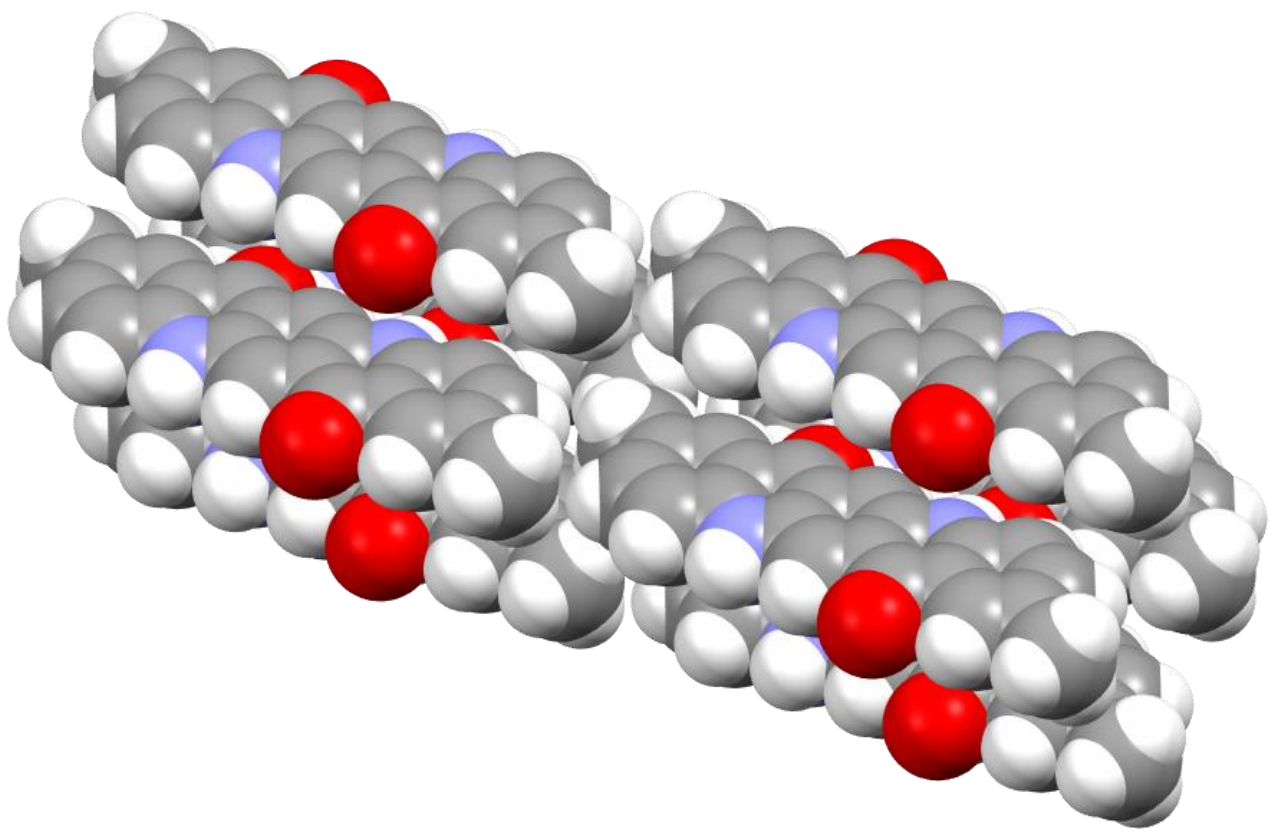

**Figure S40**. Pigment molecule ensemble (PR122) extracted from respective literature-known crystal structure: CCDC-203604.[14] Depicted cut-out (8 pigment molecules) was used for TDDFT simulations, see Computational Details.

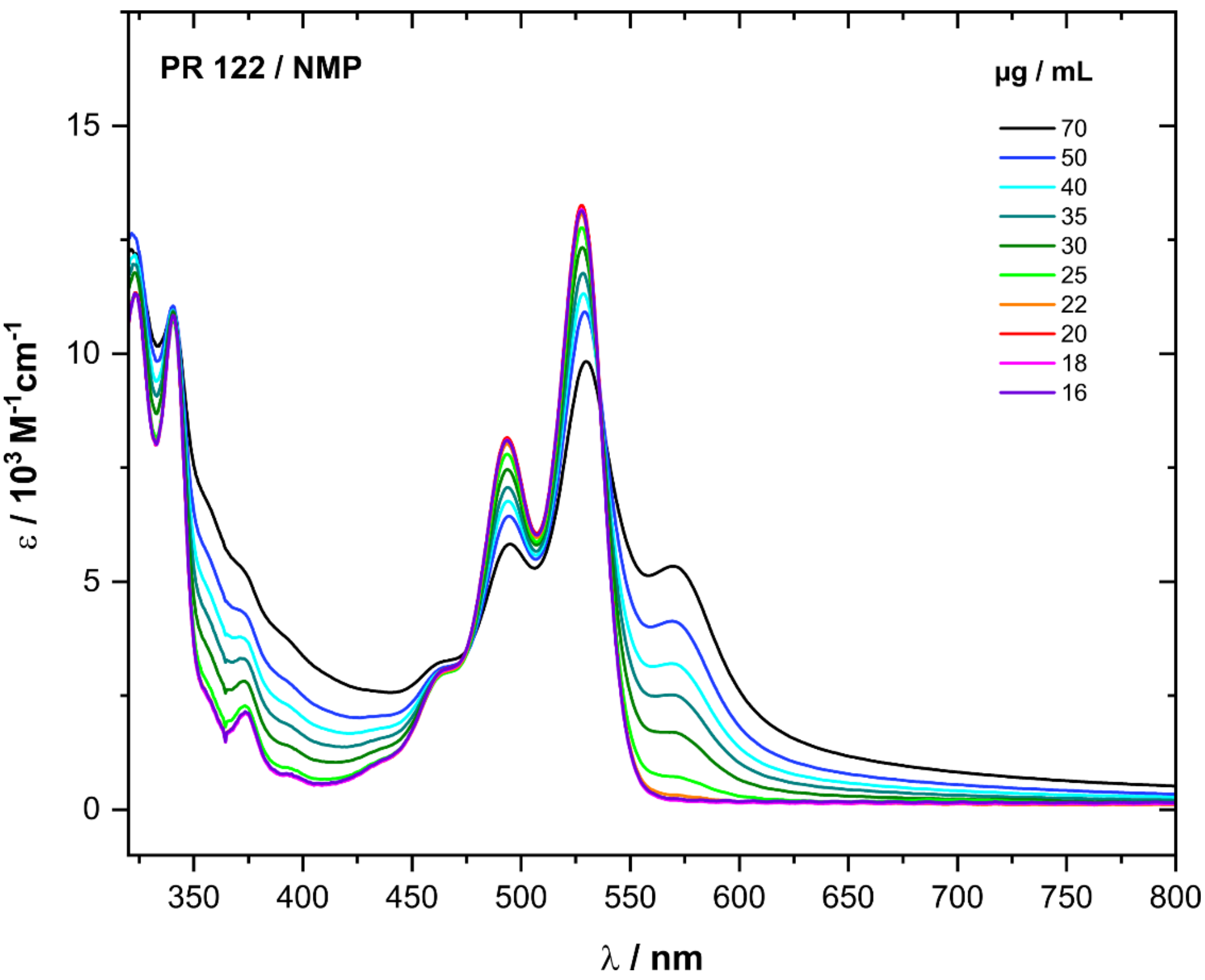


**Figure S41**. Iterative dilution procedure of PR122 in NMP, measured in 0.5 quartz cuvette. The measurement sequence starts with a dispersion (70 µg/mL) and tracks the UV-vis spectral evolution down to 16 µg/mL.

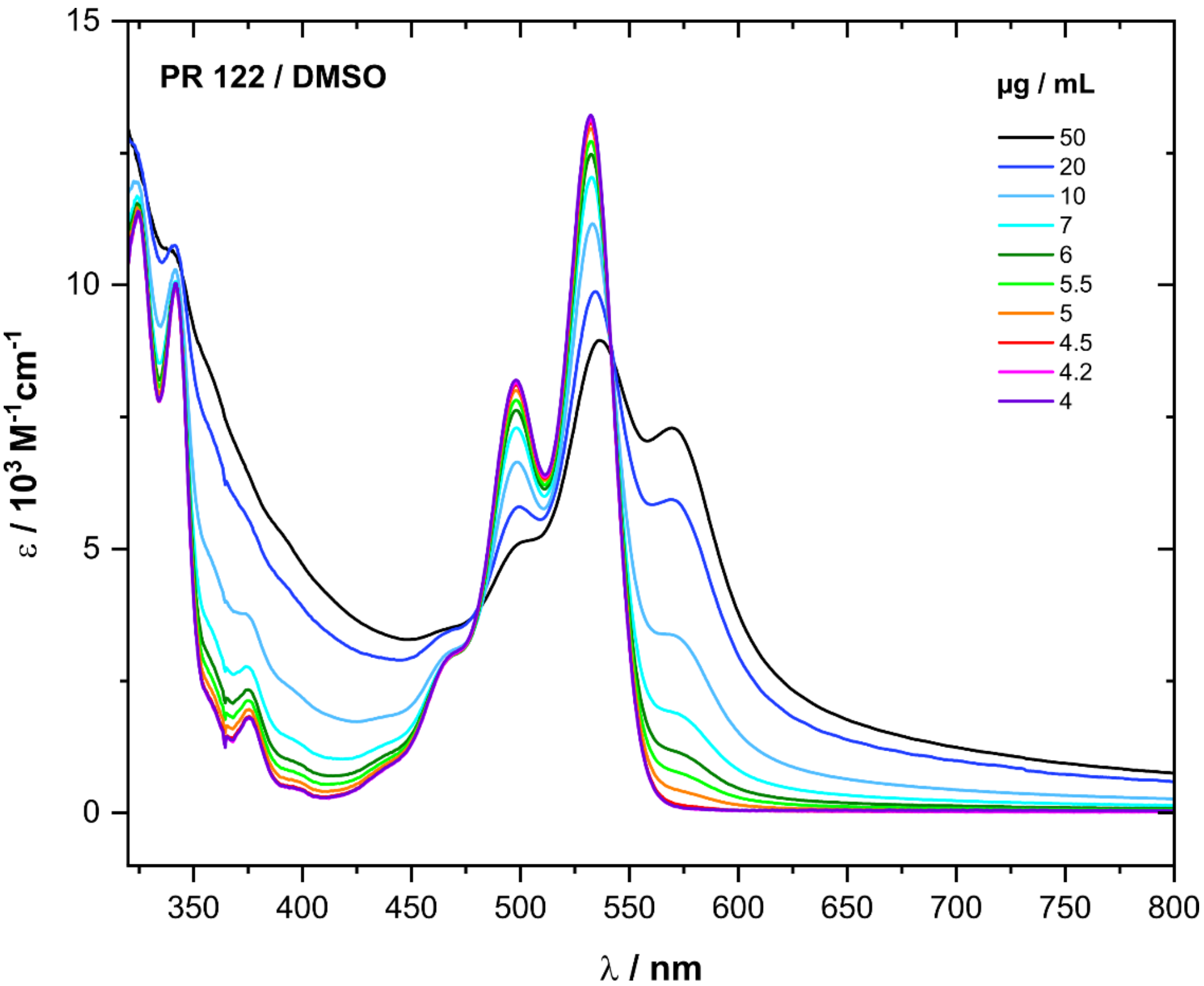


**Figure S42**. Iterative dilution procedure of PR122 in DMSO, measured in 0.5 and 2 cm quartz cuvettes. The measurement sequence starts with a dispersion (50 µg/mL) and tracks the UV-vis spectral evolution down to 4 µg/mL.

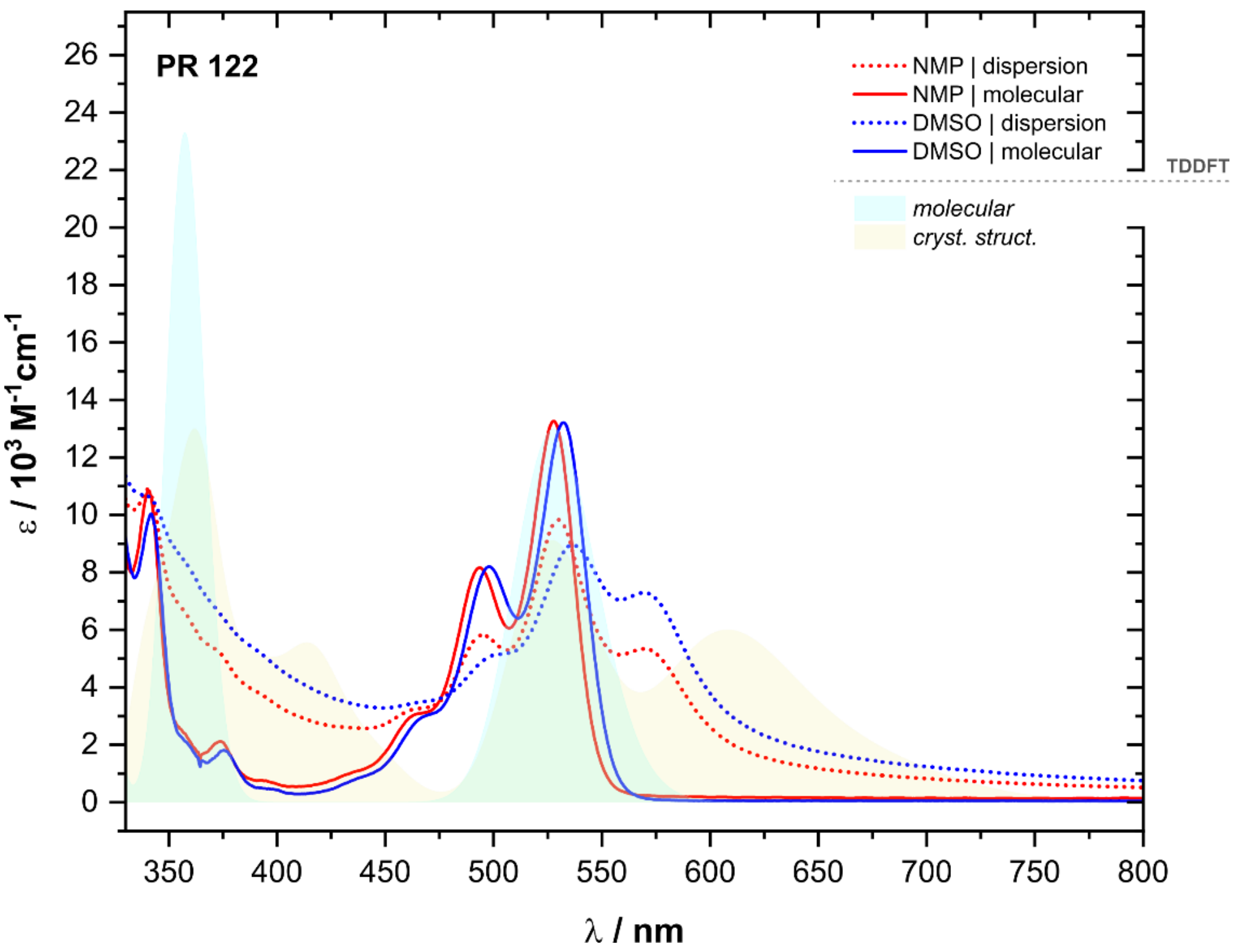


**Figure S43**. Final molecular UV-vis spectra of PR122 in NMP and DMSO (solid lines), contrasted with corresponding dispersion spectra and normalized TDDFT simulations. TDDFT simulations were shifted by -0.62 eV, respectively; peak type is Gaussian, 0.2 eV FWHM.

### 2.3.3 Pigment Red 202 (PR202)

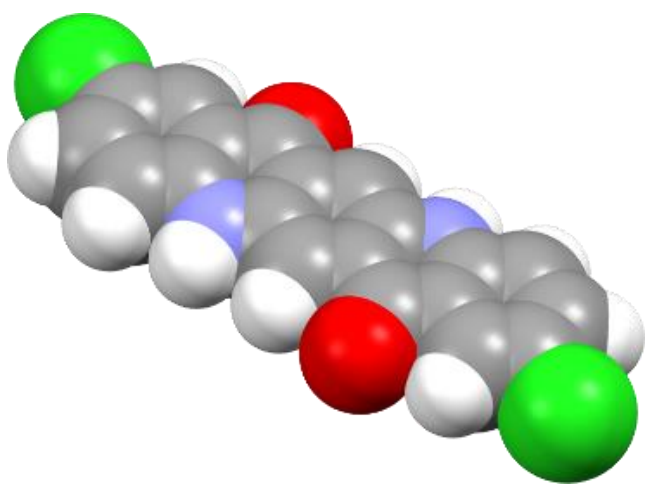

**Figure S44**. Molecular structure of PR202 after geometrical optimization in vacuo (DFT: r$^2$SCAN-3c).

a) b)

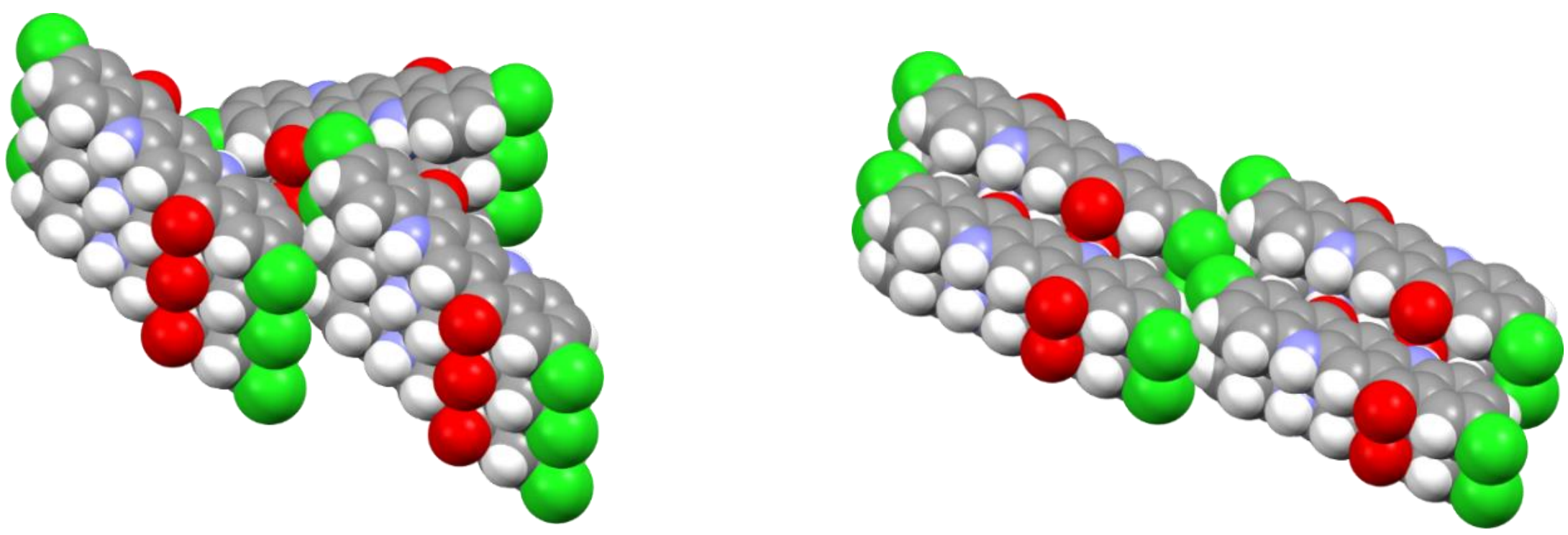

**Figure S45**. Pigment molecule ensembles (PR202) extracted from respective literature-known crystal structures, a) crystal structure A: CCDC-270501,[15] b) crystal structure B: CCDC-282570.[16] Depicted cut-outs (9 pigment molecules for A and 8 molecules for B) were used for TDDFT simulations, see Computational Details.

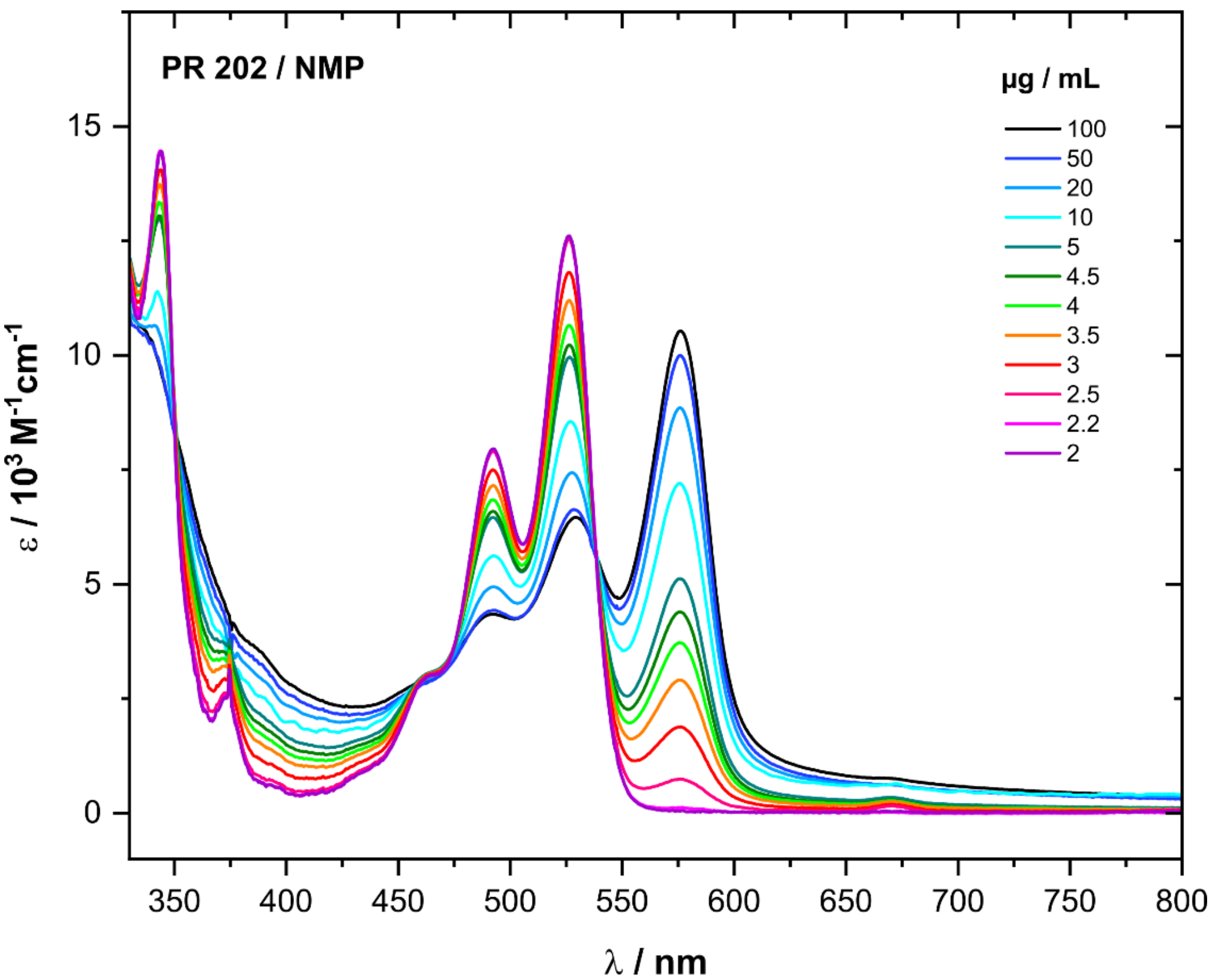


**Figure S46**. Iterative dilution procedure of PR202 in NMP, measured in 0.1 and 2 cm quartz cuvettes. The measurement sequence starts with a dispersion (100 μg/mL) and tracks the UV-vis spectral evolution down to 2 μg/mL.

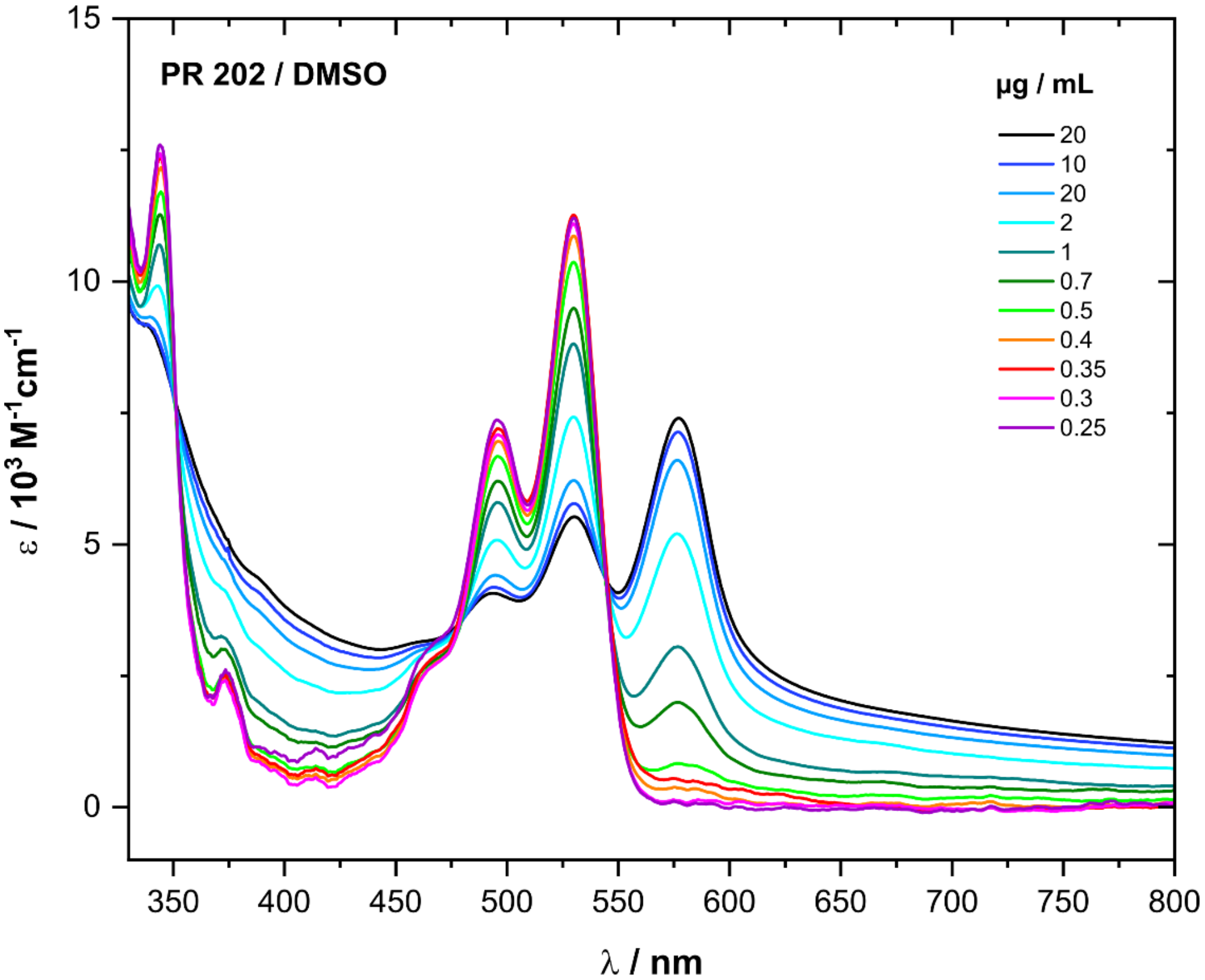


**Figure S47**. Iterative dilution procedure of PR202 in DMSO, measured in 2 cm quartz cuvette. The measurement sequence starts with a dispersion (20 μg/mL) and tracks the UV-vis spectral evolution down to 0.25 μg/mL.

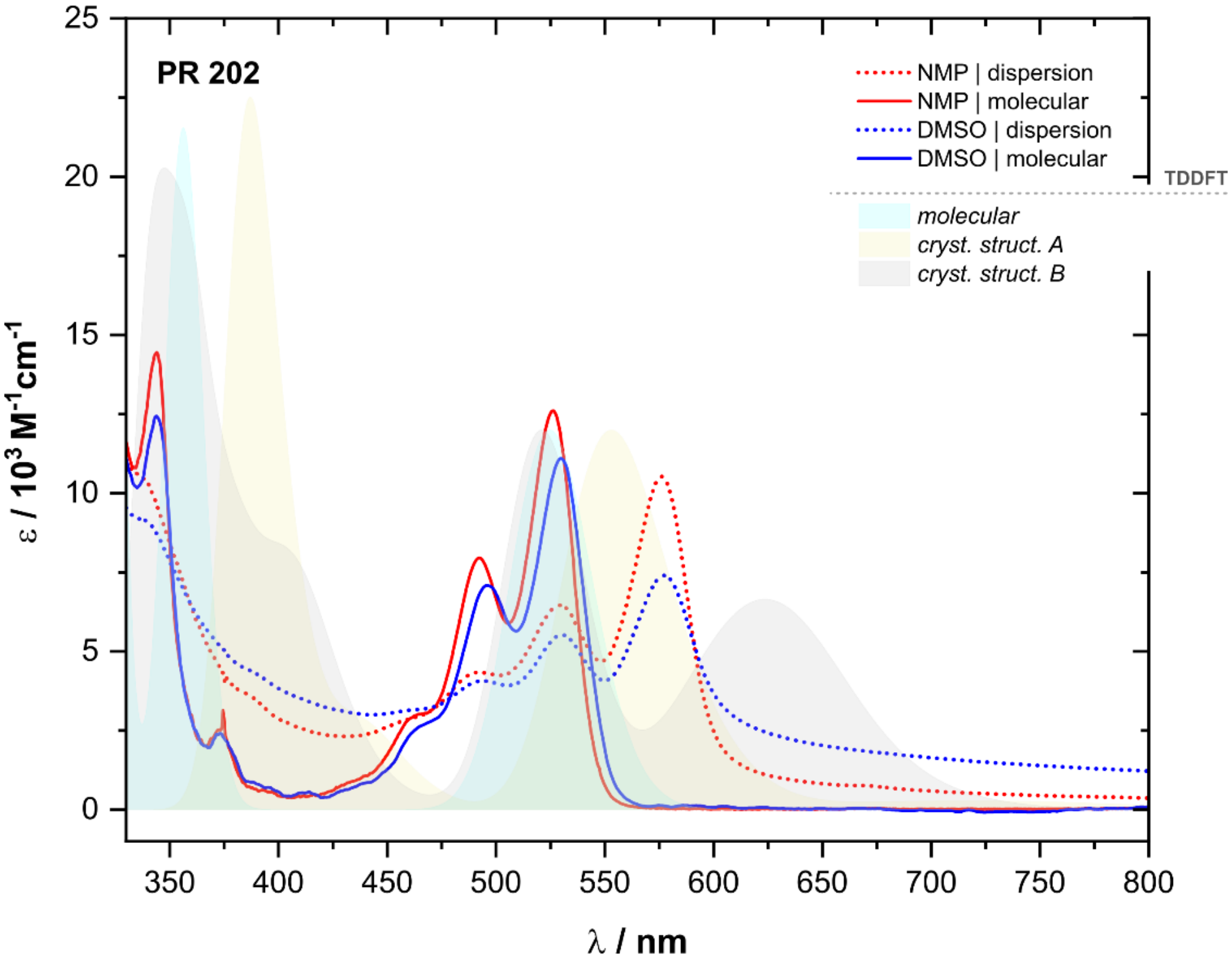


**Figure S48**. Final molecular UV-vis spectra of PR202 in NMP and DMSO (solid lines), contrasted with corresponding dispersion spectra and normalized TDDFT simulations. TDDFT simulations were shifted by -0.57 eV, respectively; peak type is Gaussian, 0.2 eV FWHM.

## 2.4 Diketopyrrolopyrrole Pigment Class

### 2.4.1 Pigment Red 255 (PR255)

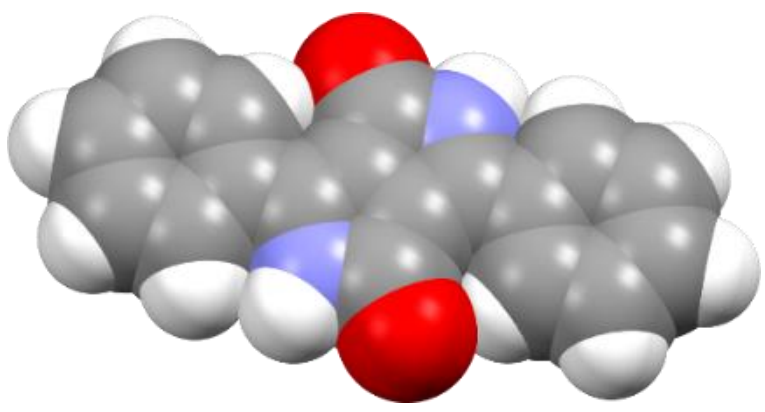

**Figure S49**. Molecular structure of PR255 after geometrical optimization in vacuo (DFT: r²SCAN-3c).

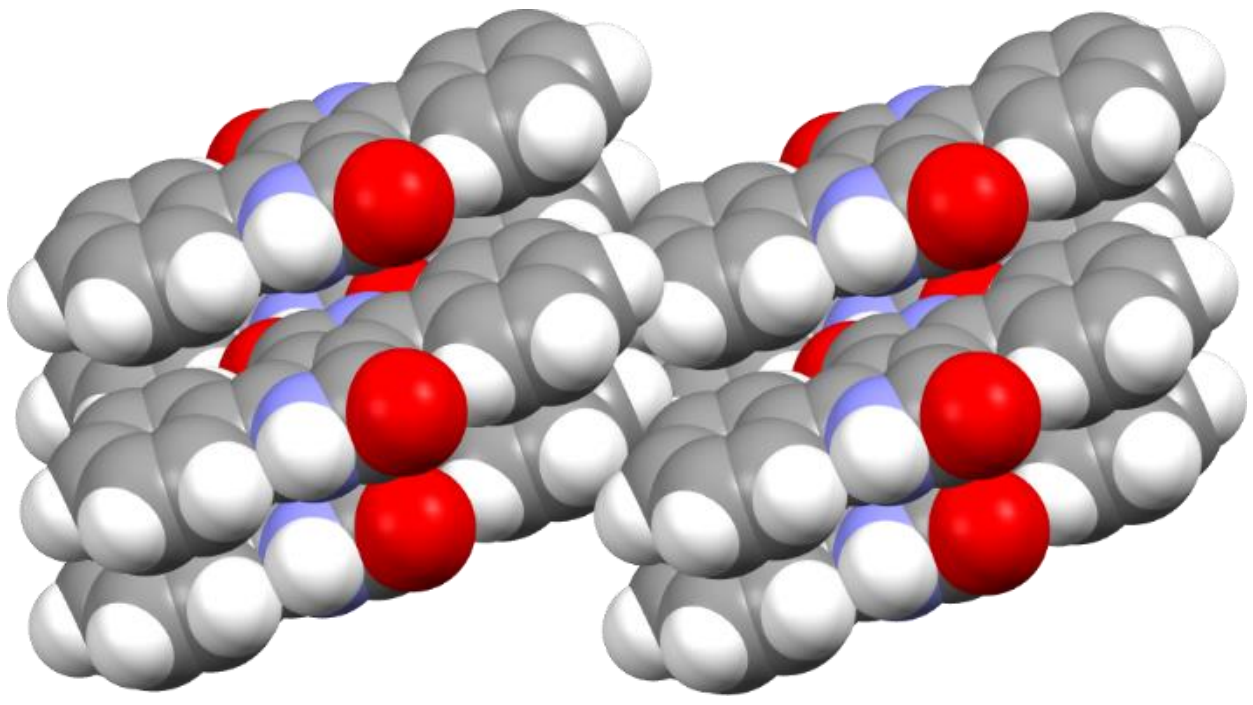

**Figure S50**. Pigment molecule ensemble (PR255) extracted from respective literature-known crystal structure CCDC-1254905.[17] Depicted cut-out (8 pigment molecules) was used for TDDFT simulations, see Computational Details.

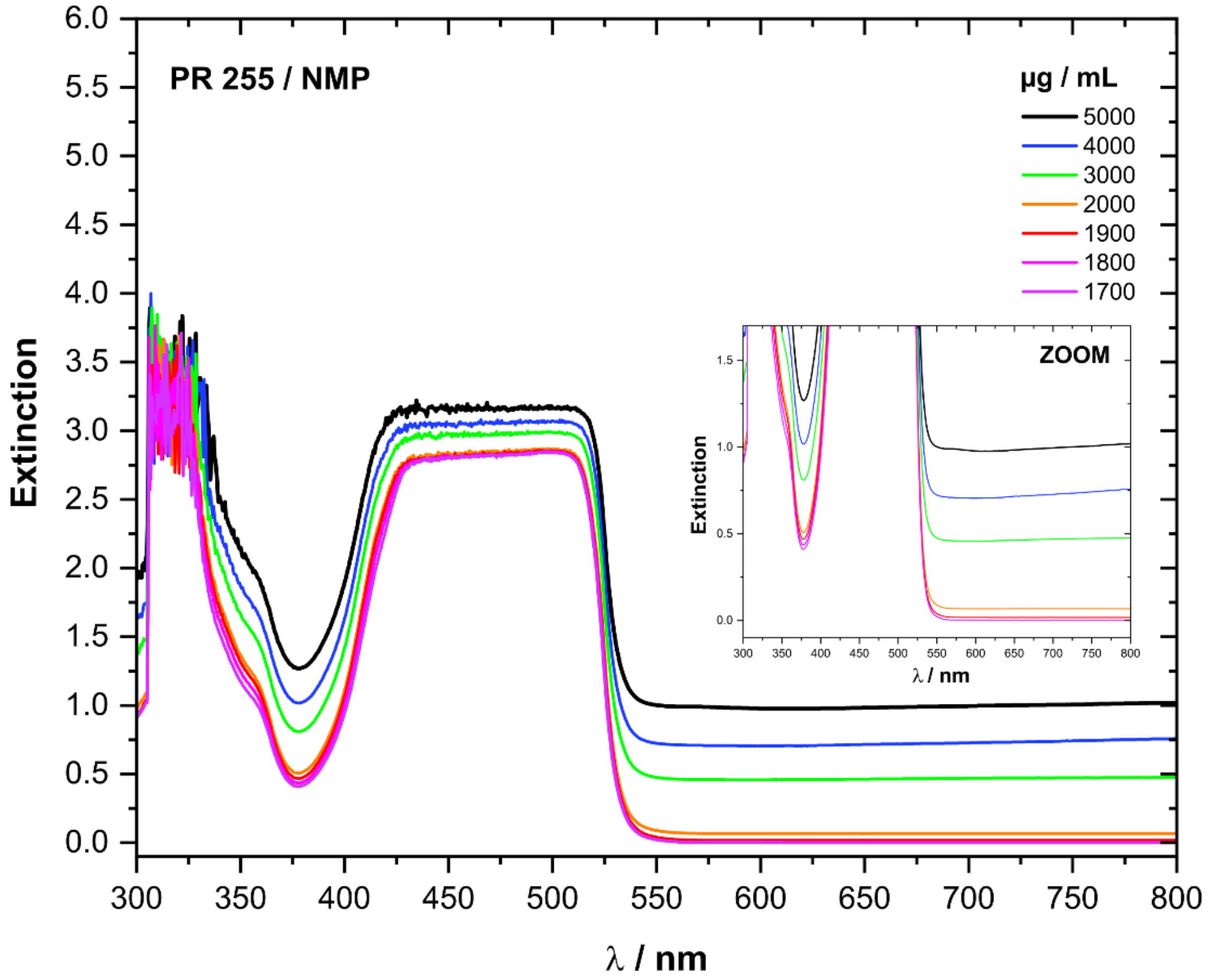


**Figure S51**. Iterative dilution procedure of PR255 in NMP, measured in a 0.1 cm quartz cuvette. The extinction exceeds the threshold of 1 and reaches instrumental saturation, data points above 1 should be regarded only as instrumental read-out. The decline of the scattering signal was used as indicator for the transition point instead. The measurement sequence starts with a dispersion (5000 µg/mL) and tracks the UV-vis spectral evolution down to 1700 µg/mL.

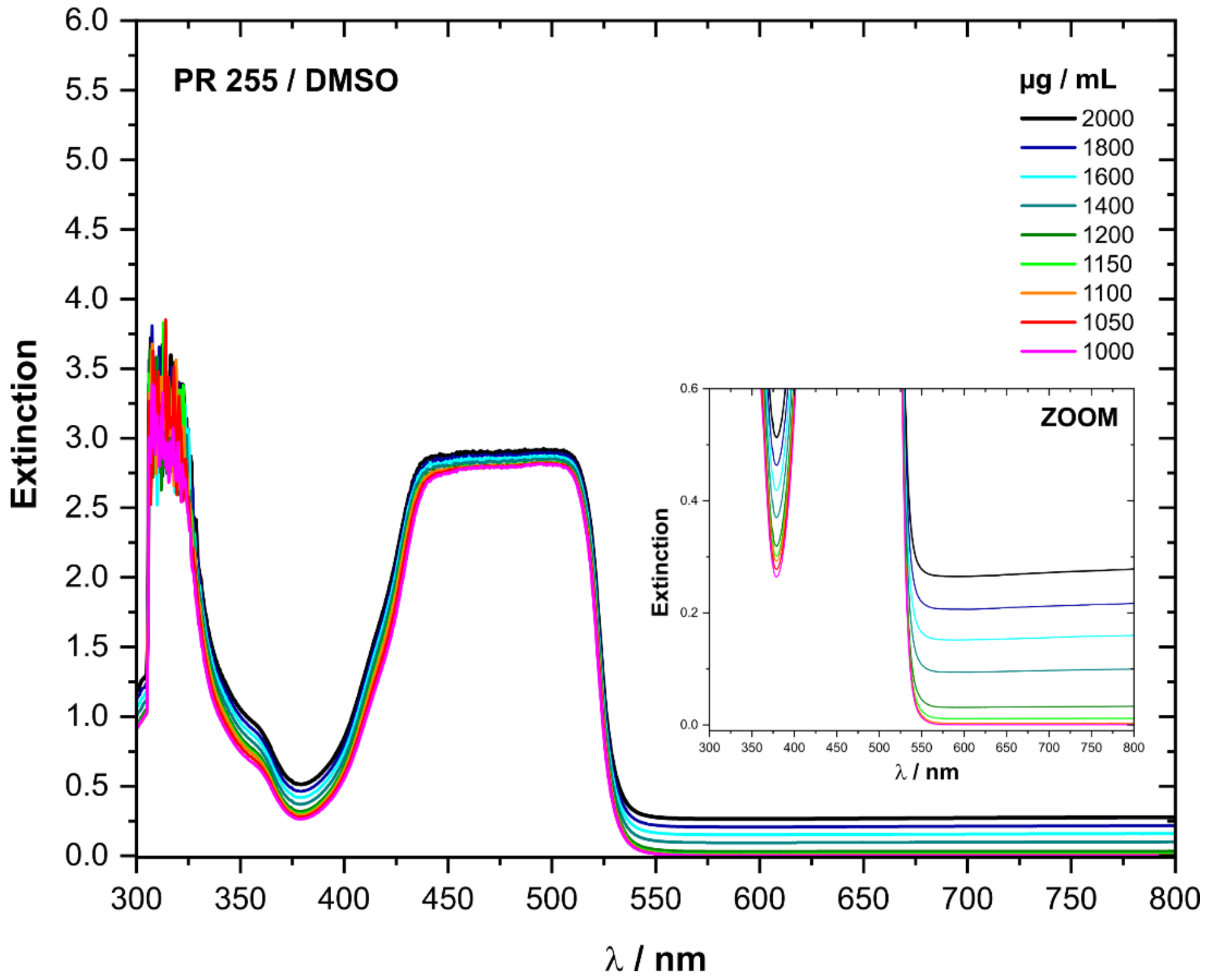


**Figure S52**. Iterative dilution procedure of PR255 in DMSO, measured in a 0.1 cm quartz cuvette. The extinction exceeds the threshold of 1 and reaches instrumental saturation, data points above 1 should be regarded only as instrumental read-out. The decline of the scattering signal was used as indicator for the transition point instead. The measurement sequence starts with a dispersion (2000 µg/mL) and tracks the UV-vis spectral evolution down to 1000 µg/mL.

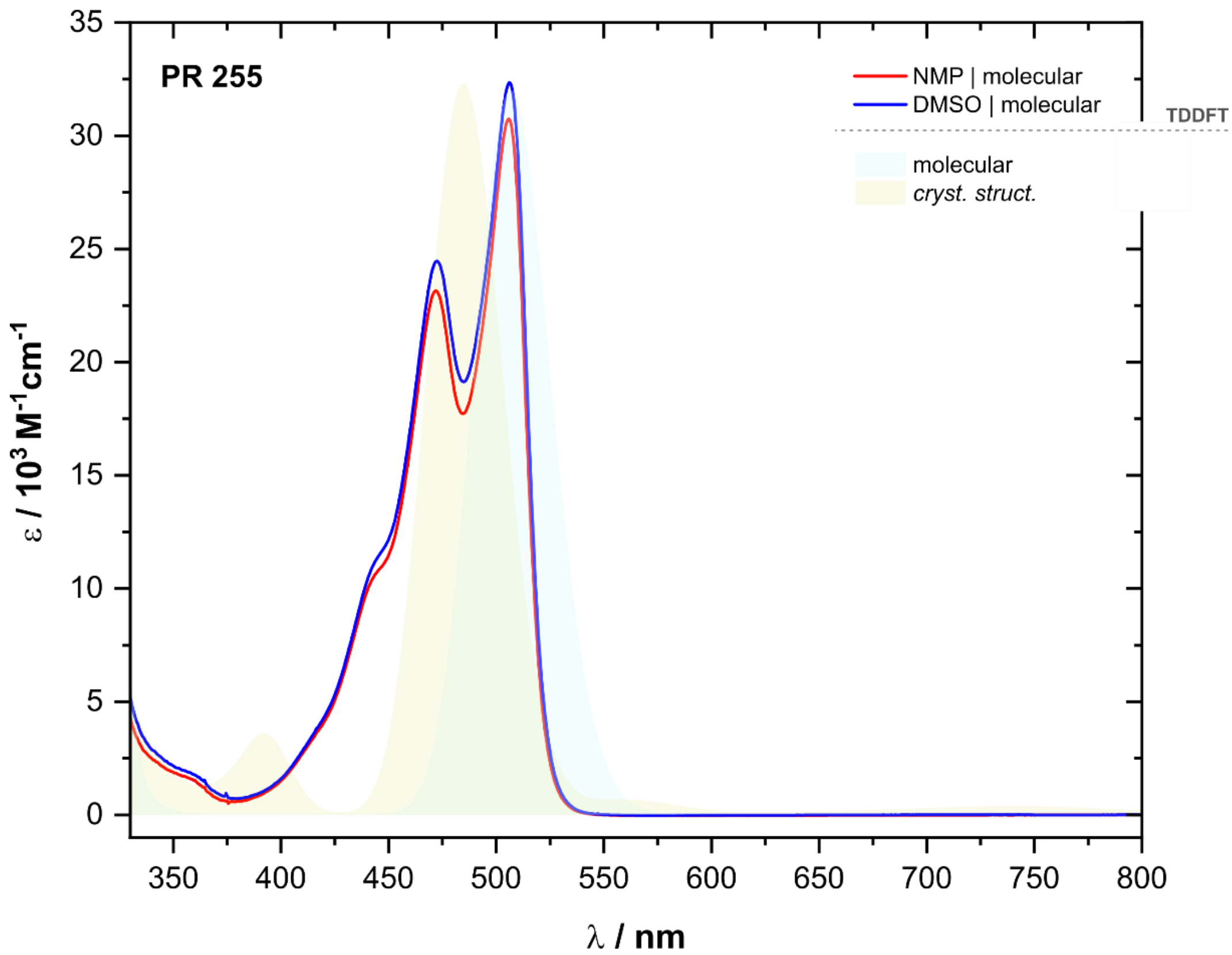


**Figure S53**. Final molecular UV-vis spectra of PR255 in NMP and DMSO (solid lines) at ca. 10 µg/mL, contrasted with normalized TDDFT simulations. TDDFT simulations were shifted by -0.56 eV, respectively; peak type is Gaussian, 0.2 eV FWHM.

### 2.4.2 Pigment Red 254 (PR254)

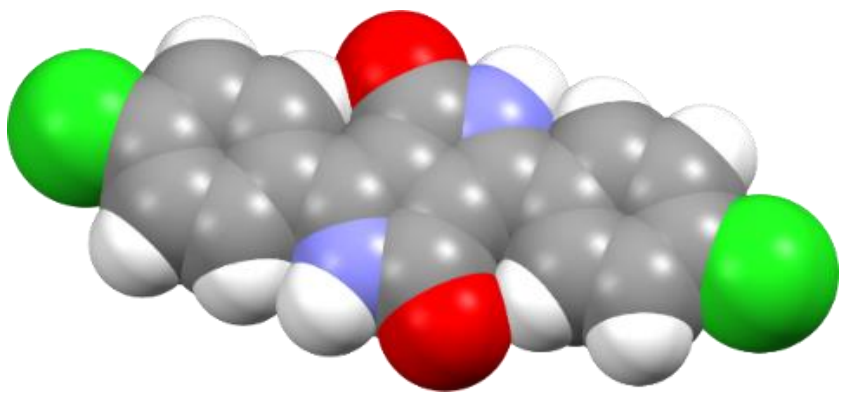

**Figure S54**. Molecular structure of PR254 after geometrical optimization in vacuo (DFT: r$^2$SCAN-3c).

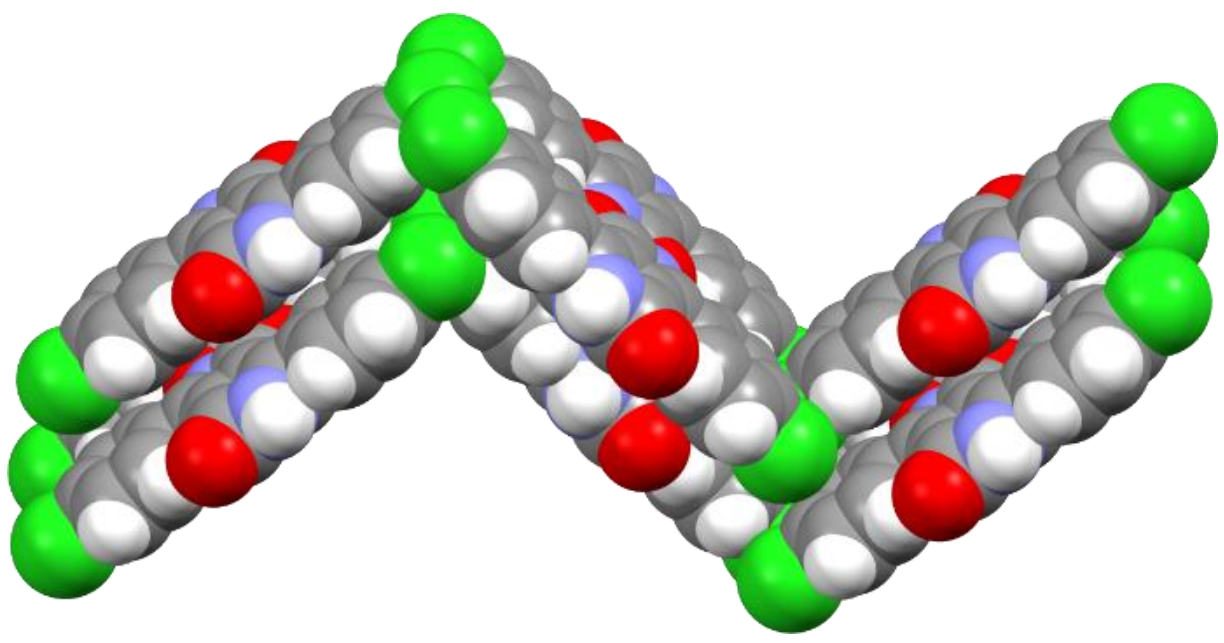

**Figure S55**. Pigment molecule ensemble (PR254) extracted from respective literature-known crystal structure CCDC-1291032.[18] Depicted cut-out (9 pigment molecules) was used for TDDFT simulations, see Computational Details.

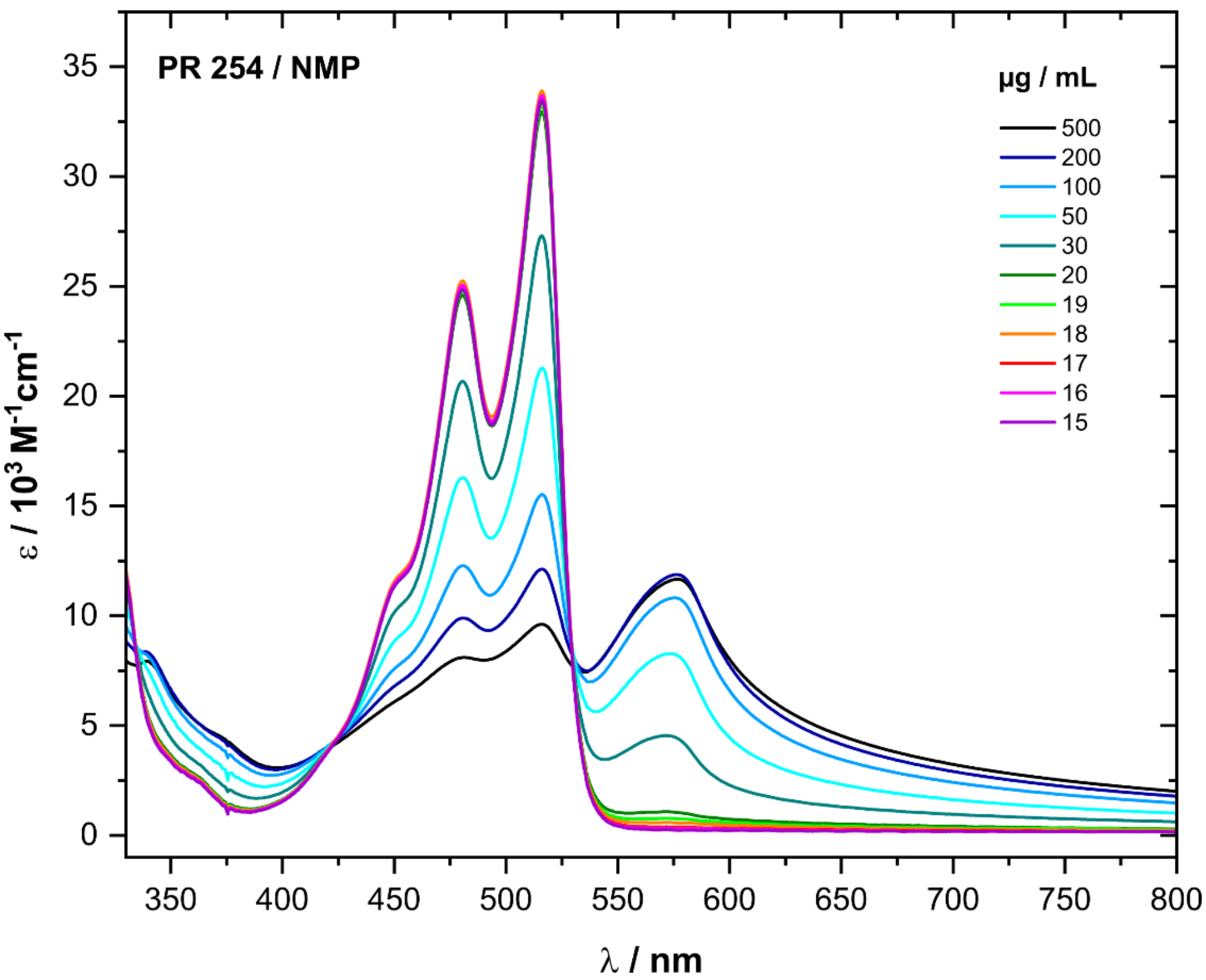


**Figure S56**. Iterative dilution procedure of PR254 in NMP, measured in 0.1 and 0,5 cm quartz cuvettes. The measurement sequence starts with a dispersion (500 µg/mL) and tracks the UV-vis spectral evolution down to 15 µg/mL.

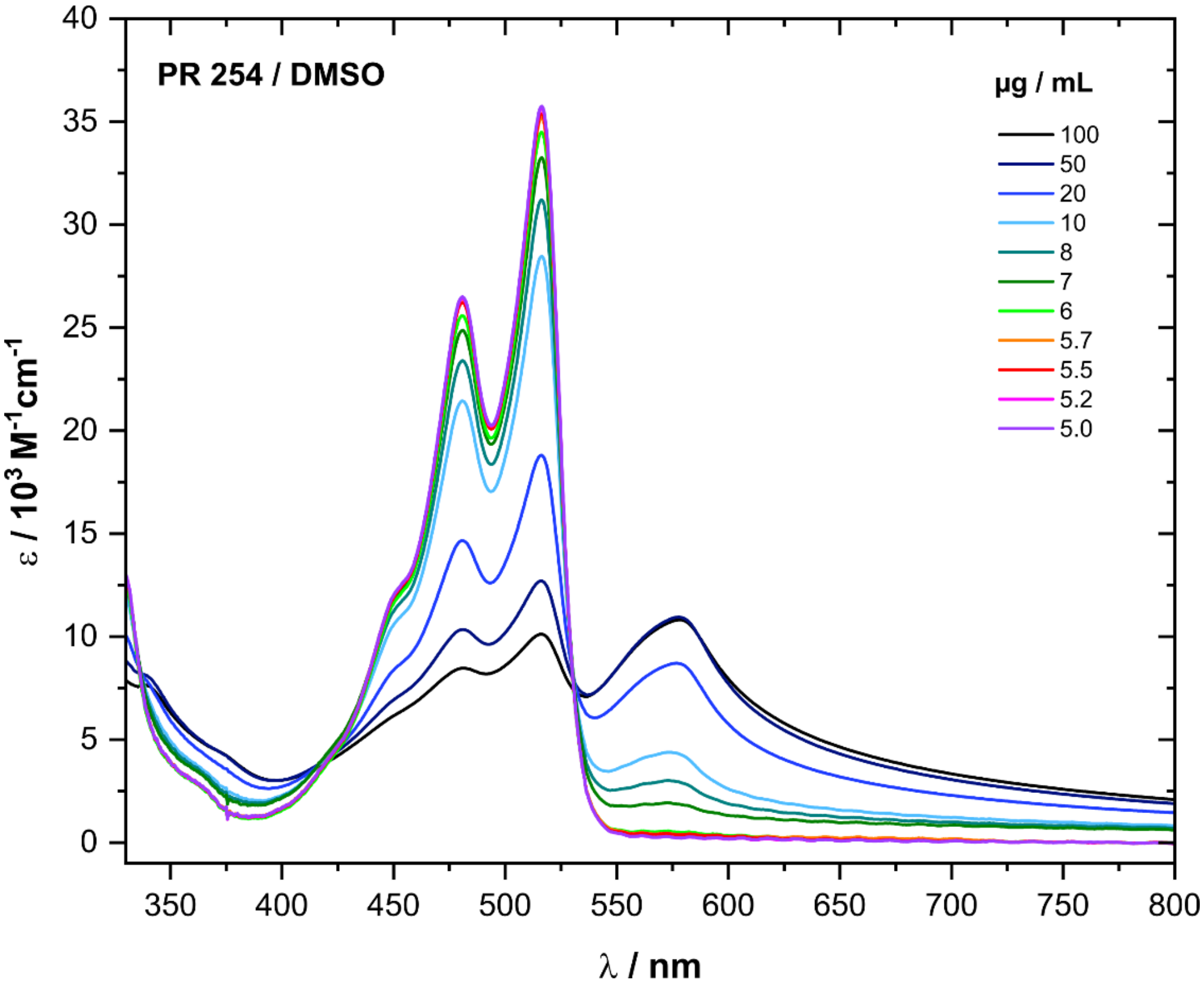


**Figure S57**. Iterative dilution procedure of PR254 in DMSO, measured in 0.5 cm quartz cuvette. The measurement sequence starts with a dispersion (100 µg/mL) and tracks the UV-vis spectral evolution down to 5.0 µg/mL.

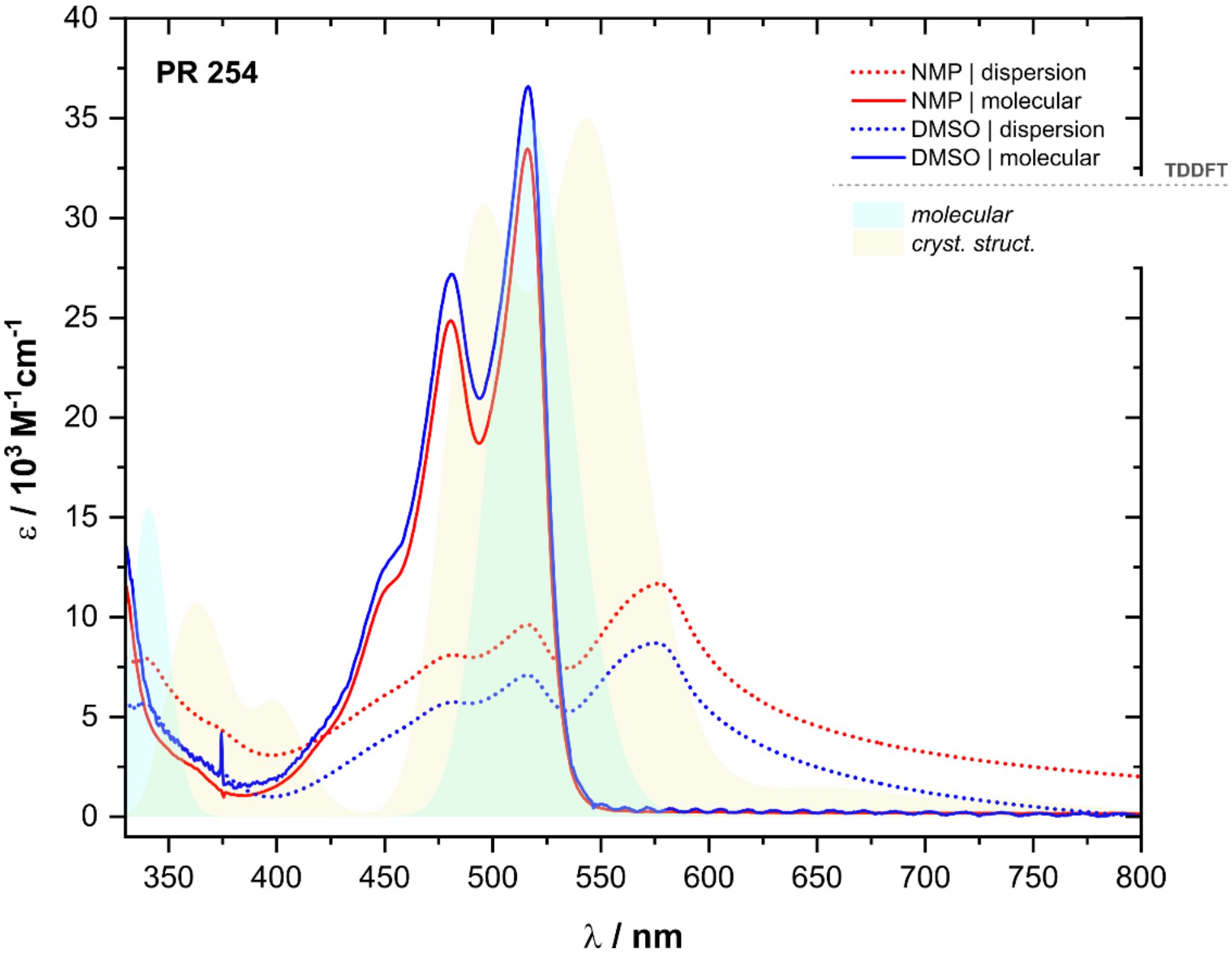


**Figure S58**. Final molecular UV-vis spectra of PR254 in NMP and DMSO (solid lines), contrasted with corresponding dispersion spectra and normalized TDDFT simulations. TDDFT simulations were shifted by -0.54 eV, respectively; peak type is Gaussian, 0.2 eV FWHM.

### 2.4.3 Pigment Red 264 (PR264)

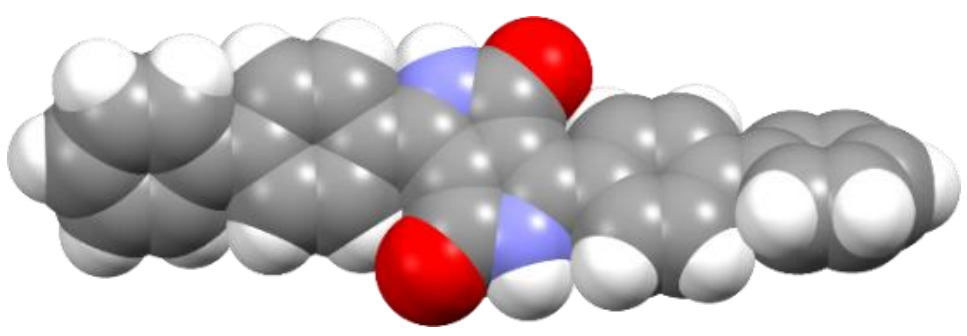

**Figure S59**. Molecular structure of PR264 after geometrical optimization in vacuo (DFT: r$^2$SCAN-3c).

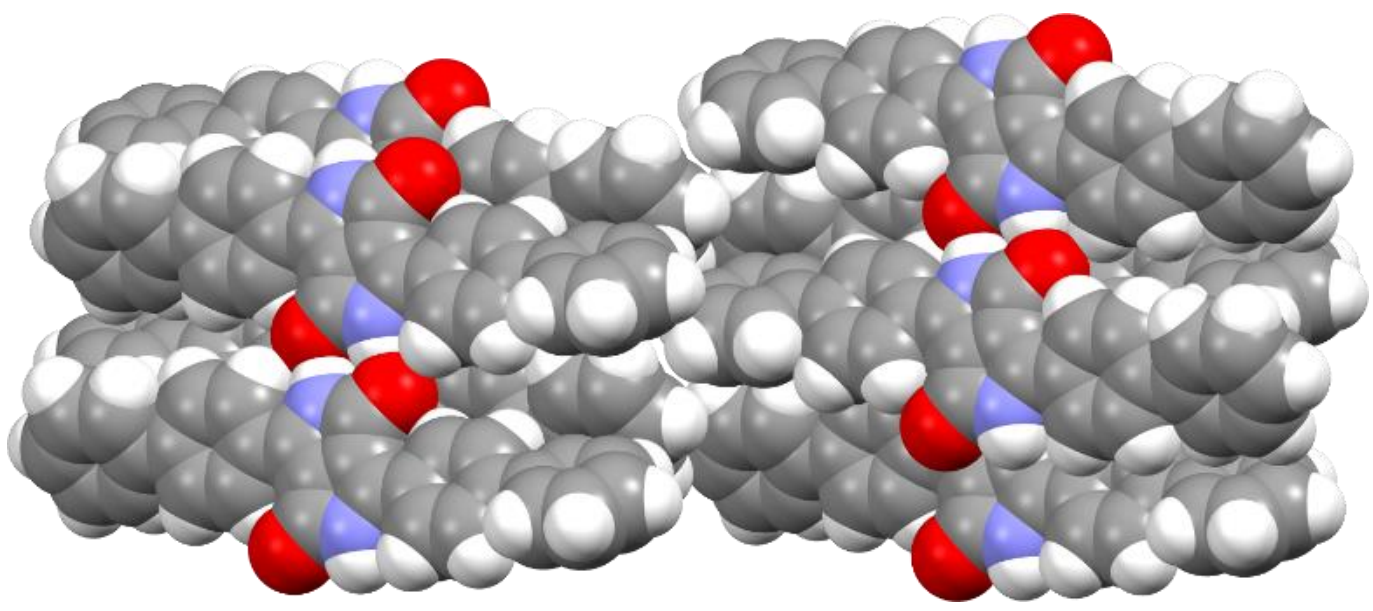

**Figure S60**. Pigment molecule ensemble (PR264) extracted from respective literature-known crystal structure CCDC-187610.[19] Depicted cut-out (8 pigment molecules) was used for TDDFT simulations, see Computational Details.

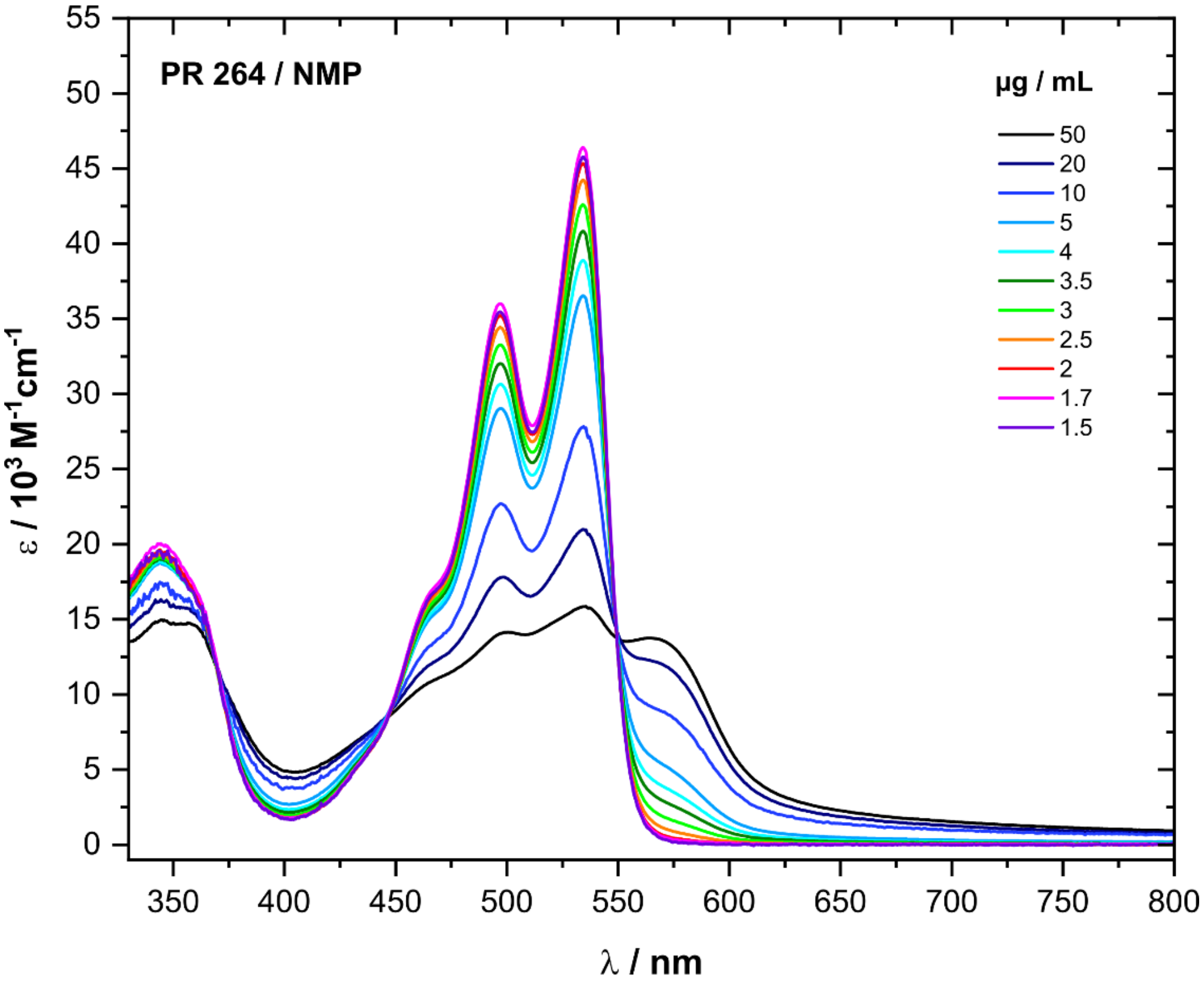


**Figure S61**. Iterative dilution procedure of PR264 in NMP, measured in 0.1 and 2 cm quartz cuvettes. The measurement sequence starts with a dispersion (50 μg/mL) and tracks the UV-vis spectral evolution down to 1.5 μg/mL.

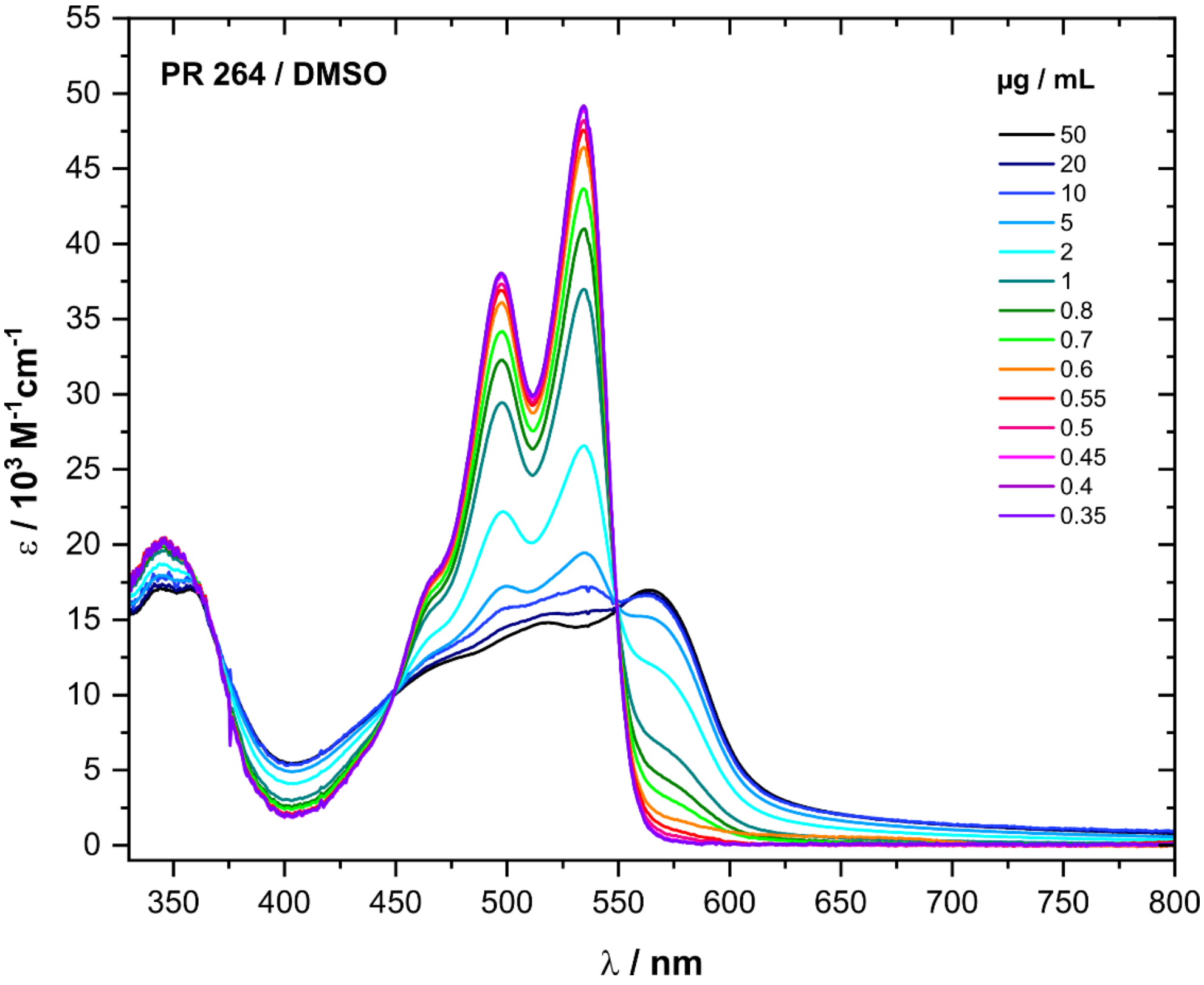


**Figure S62**. Iterative dilution procedure of PR264 in DMSO, measured in 0.1 and 2 cm quartz cuvettes. The measurement sequence starts with a dispersion (50 μg/mL) and tracks the UV-vis spectral evolution down to 0.35 μg/mL.

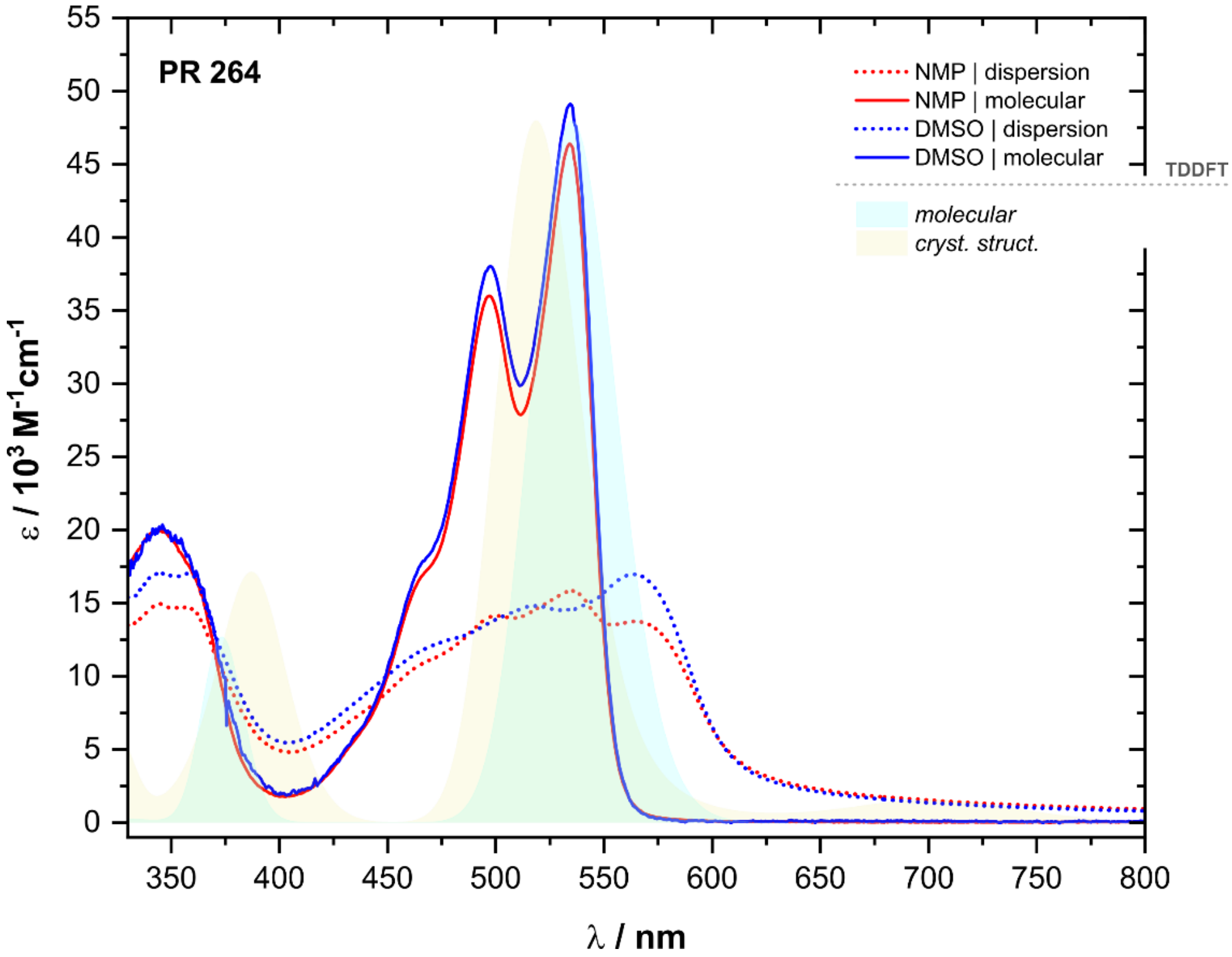


**Figure S63**. Final molecular UV-vis spectra of PR264 in NMP and DMSO (solid lines), contrasted with corresponding dispersion spectra and normalized TDDFT simulations. TDDFT simulations were shifted by -0.40 eV, respectively; peak type is Gaussian, 0.2 eV FWHM.

### 2.4.4 Pigment Orange 73 (PO73)

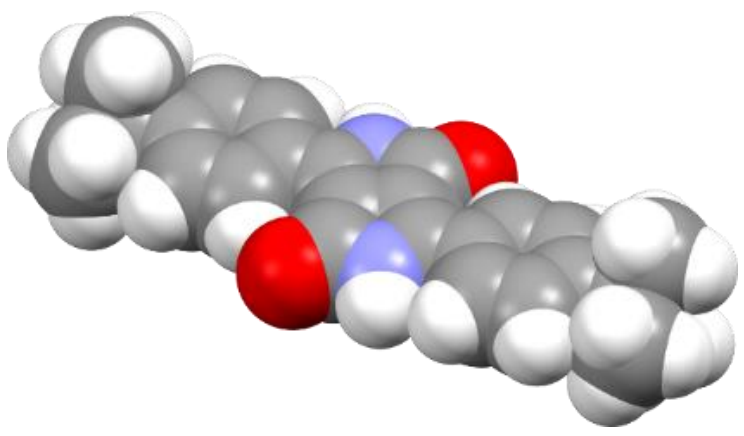

**Figure S64**. Molecular structure of PO73 after geometrical optimization in vacuo (DFT: r$^2$SCAN-3c).

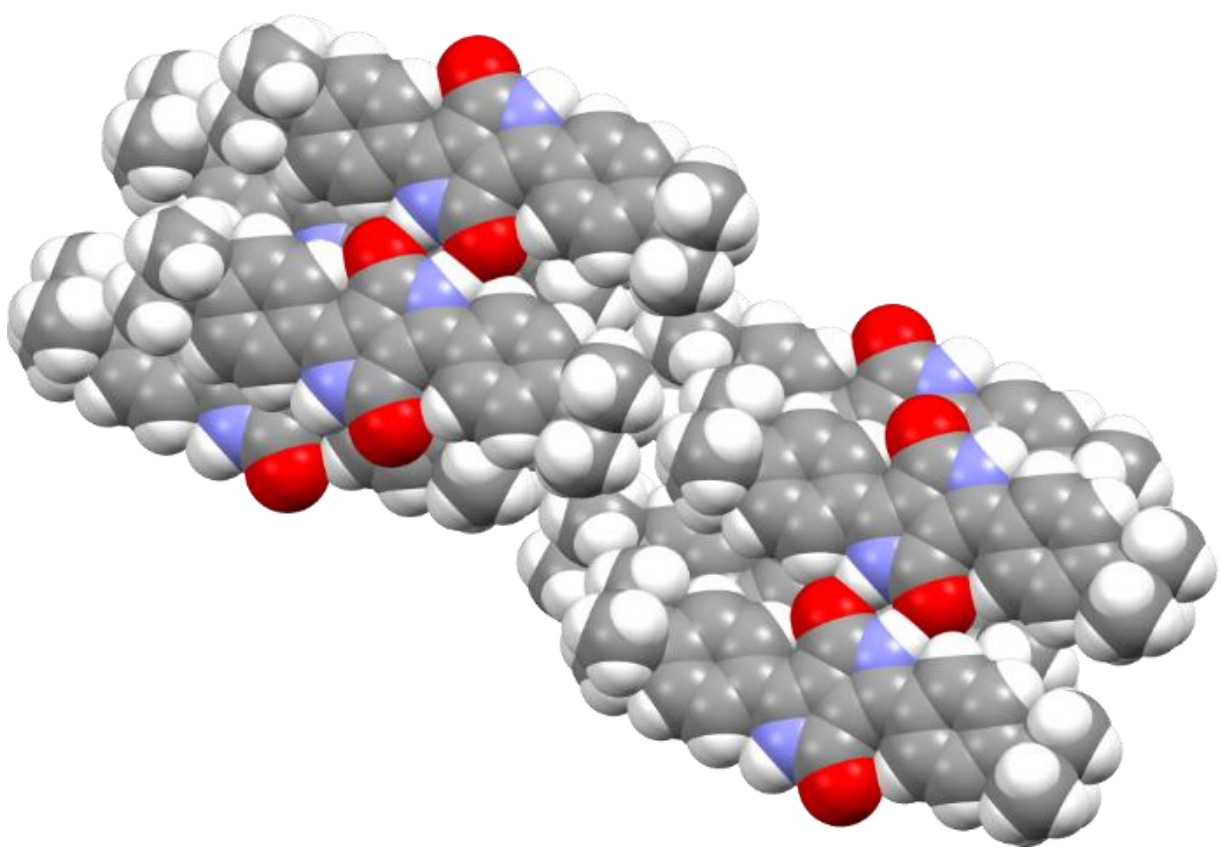

**Figure S65**. Pigment molecule ensemble (PO73) extracted from respective literature-known crystal structure CCDC-130508.[20] Depicted cut-out (8 pigment molecules) was used for TDDFT simulations, see Computational Details.

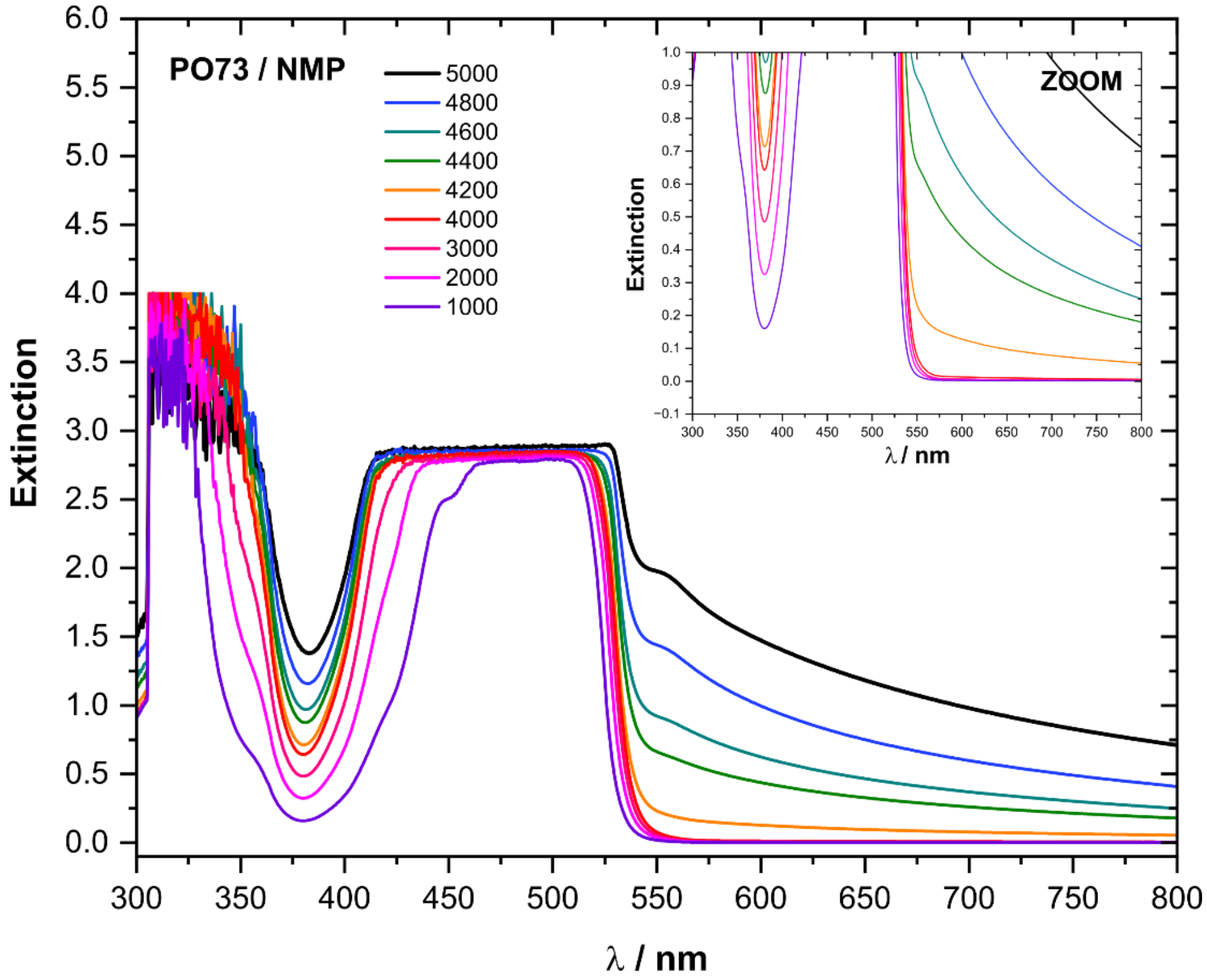


**Figure S66**. Iterative dilution procedure of PO73 in NMP, measured in a 0.1 cm quartz cuvette. The extinction exceeds the threshold of 1 and reaches instrumental saturation, data points above 1 should be regarded only as instrumental read-out. The decline of the scattering signal was used as indicator for the transition point instead. The measurement sequence starts with a dispersion (5000 µg/mL) and tracks the UV-vis spectral evolution down to 1000 µg/mL.

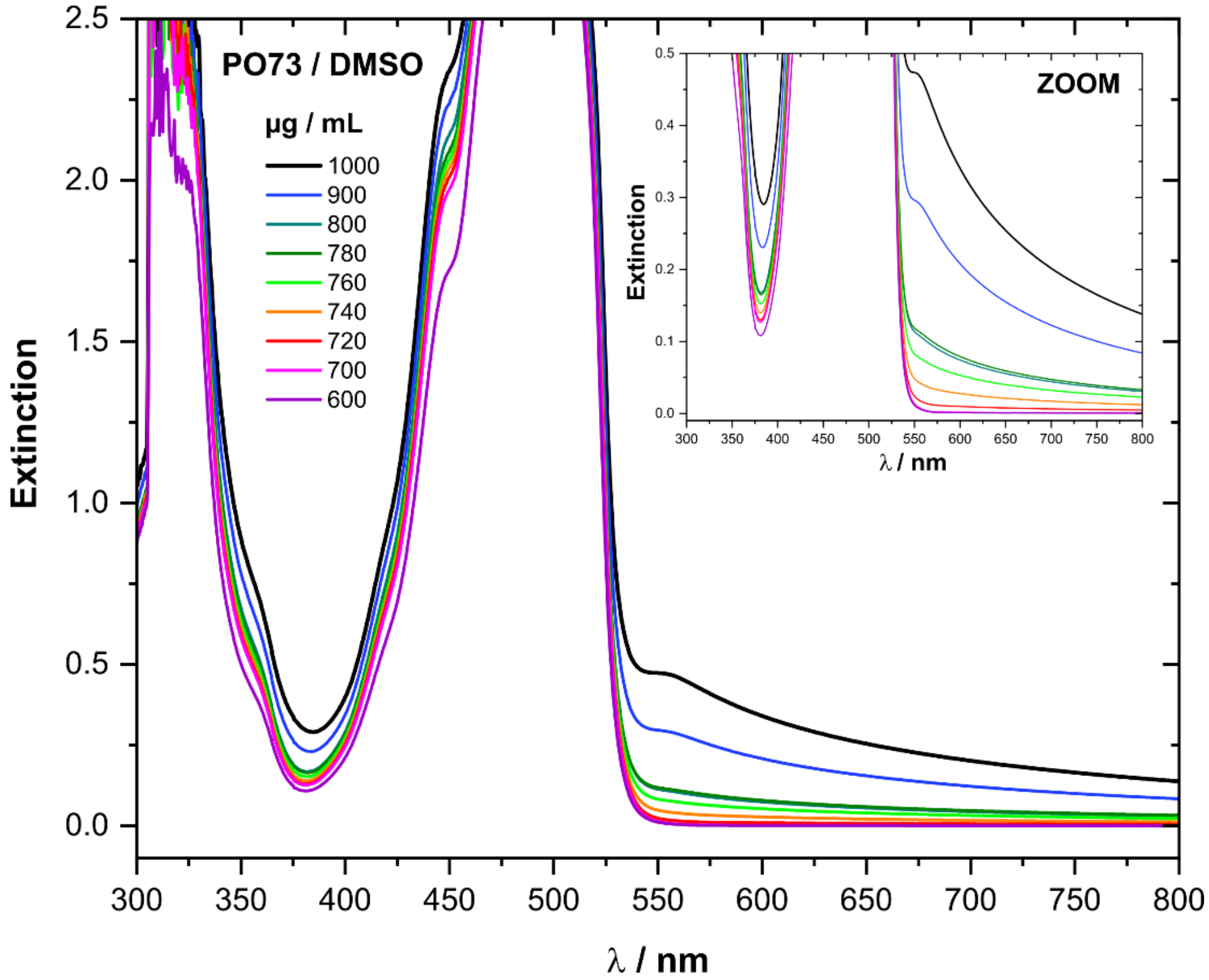


**Figure S67**. Iterative dilution procedure of PO73 in DMSO, measured in a 0.1 cm quartz cuvette. The extinction exceeds the threshold of 1 and reaches instrumental saturation, data points above 1 should be regarded only as instrumental read-out. The decline of the scattering signal was used as indicator for the transition point instead. The measurement sequence starts with a dispersion (1000 µg/mL) and tracks the UV-vis spectral evolution down to 600 µg/mL.

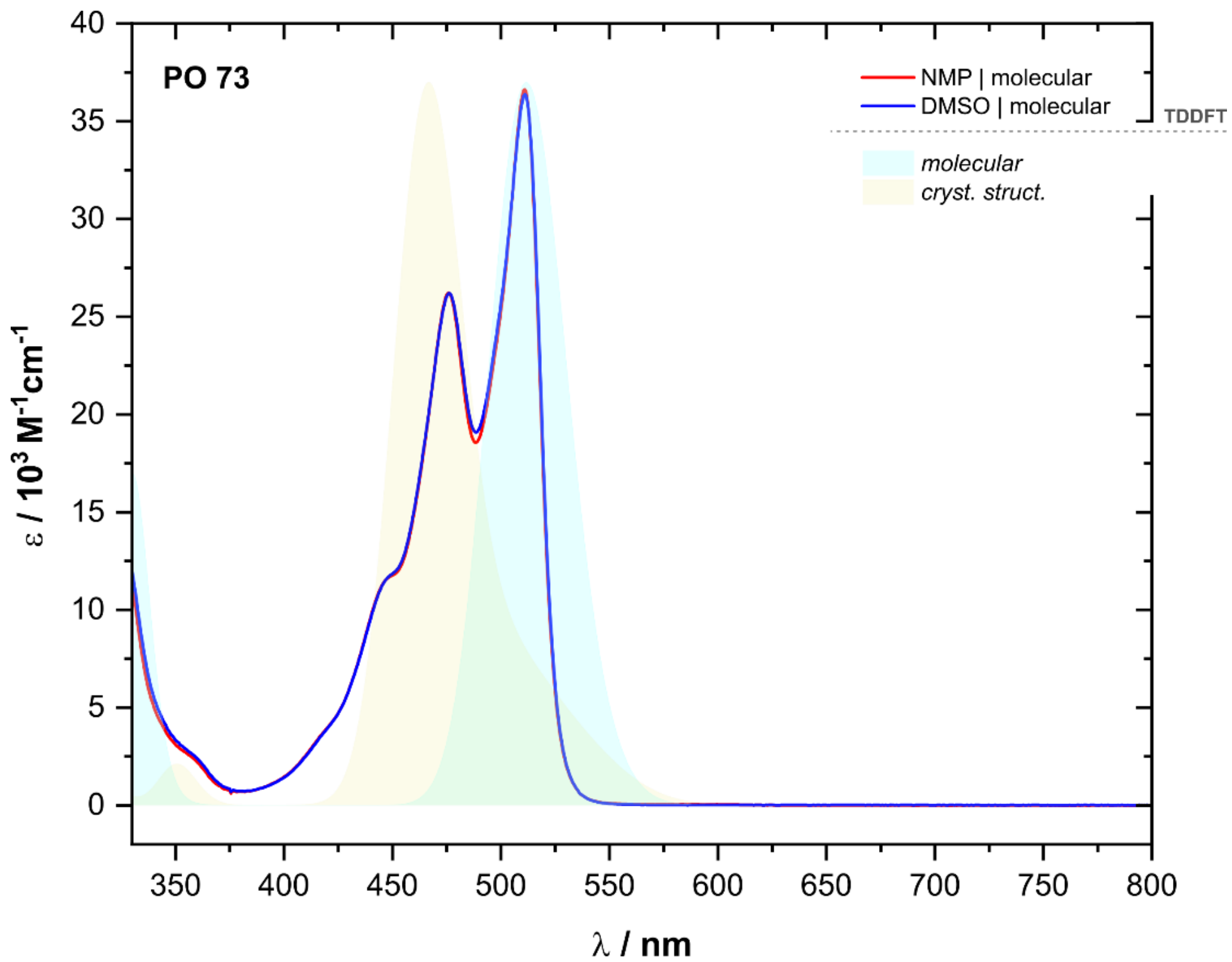


**Figure S68**. Final molecular UV-vis spectra of PO73 in NMP and DMSO (solid lines) at ca. 10 µg/mL, contrasted with normalized TDDFT simulations. TDDFT simulations were shifted by -0.54 eV, respectively; peak type is Gaussian, 0.2 eV FWHM.

## 2.5 Phthalocyanine Pigment Class

### 2.5.1 Pigment Blue 15 (PB15)

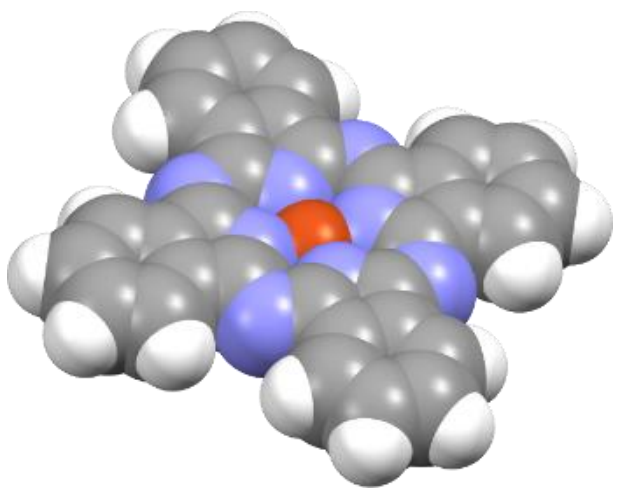

**Figure S69**. Molecular structure of PB15 after geometrical optimization in vacuo (DFT: r$^2$SCAN-3c).

a)

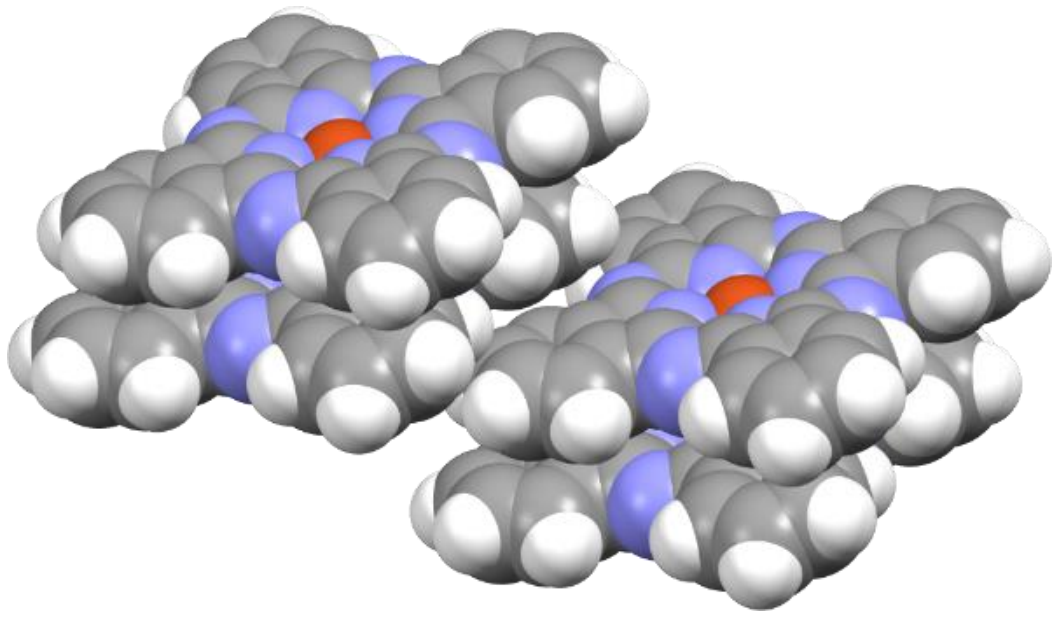

b)

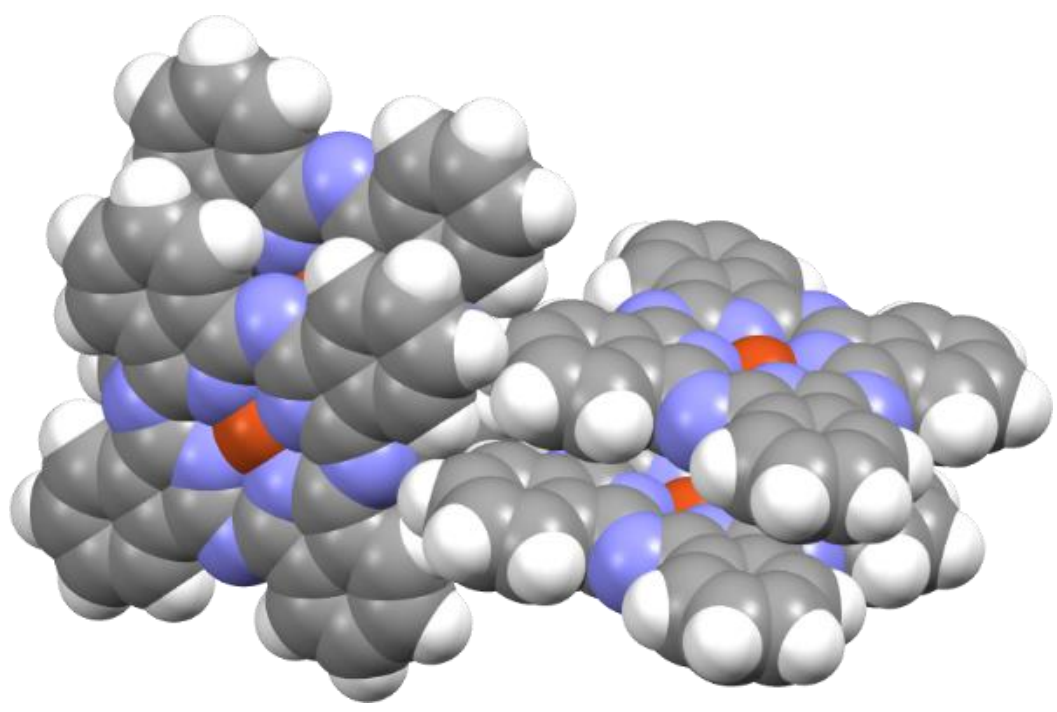

**Figure S70**. Pigment molecule ensembles (PB15) extracted from respective literature-known crystal structures, a) crystal structure *alpha* (PB15:1): CCDC-219250,[23] b) crystal structure *beta* (PB15:3): CCDC-282570.[24] Depicted cut-outs (4 pigment molecules each) were used for TDDFT simulations, see Computational Details.

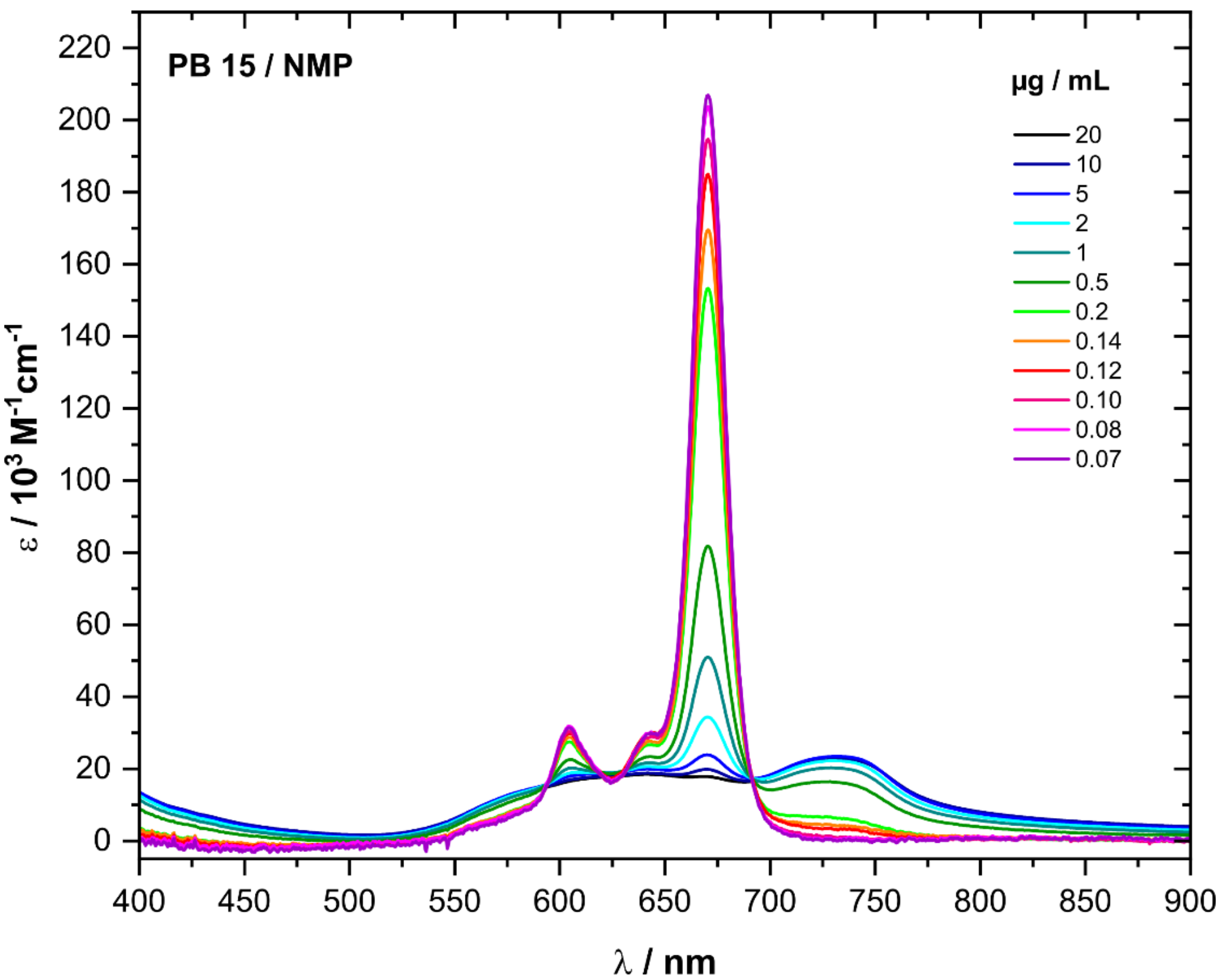


**Figure S71**. Iterative dilution procedure of PB15 in NMP, measured in a 2 cm quartz cuvette. The measurement sequence starts with a dispersion (20 µg/mL) and tracks the UV-vis spectral evolution down to 0.07 µg/mL. PB15:3 has been used for analysis, the solubility however does not depend on the type of polymorph (after sufficient equilibration).

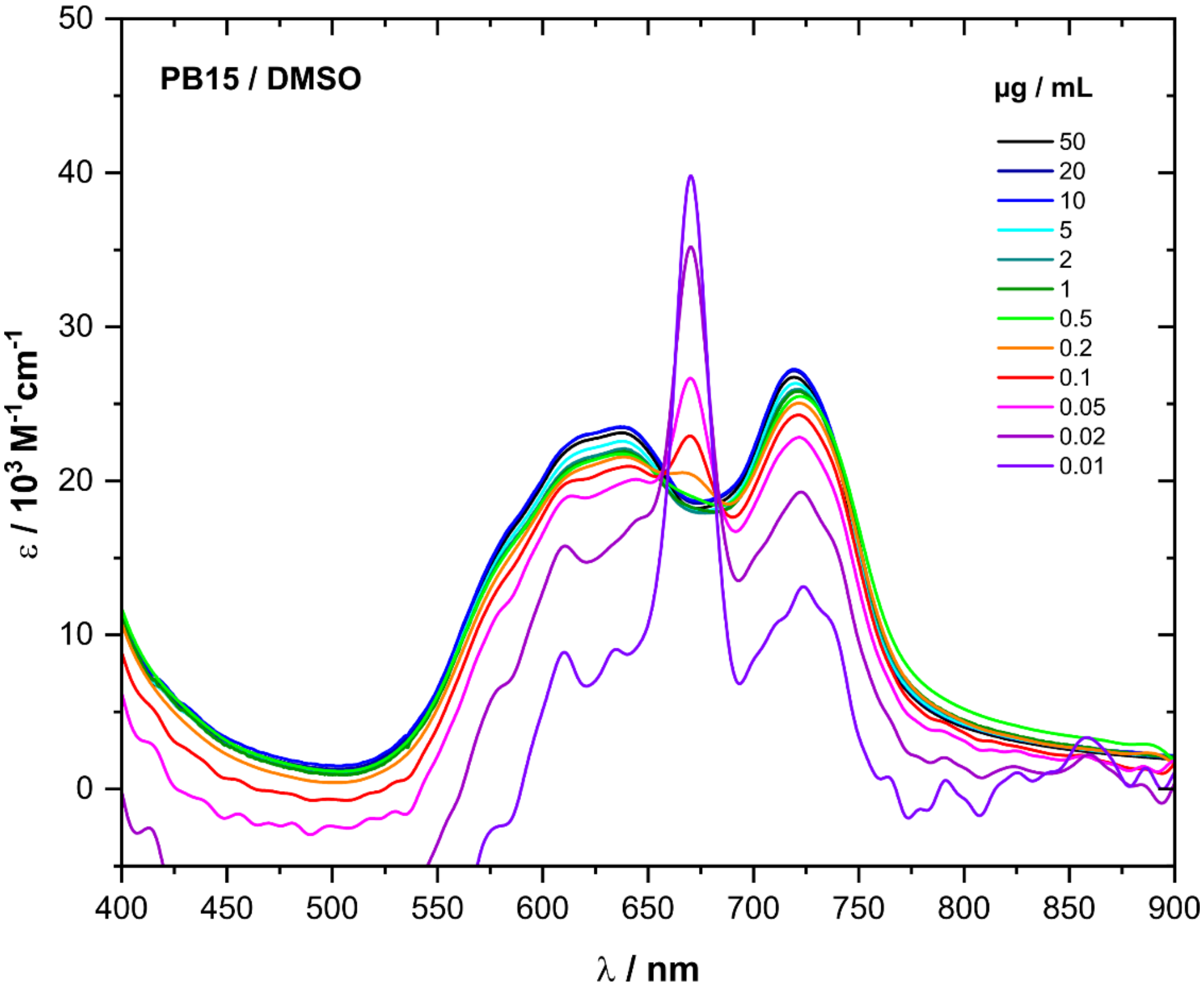


**Figure S72**. Iterative dilution procedure of PB15 in DMSO, measured in a 2 cm quartz cuvette. The measurement sequence starts with a dispersion (20 µg/mL) and tracks the UV-vis spectral evolution down to 0.01 µg/mL. Due to the low concentrations the sample reaches the instrumental detection limits before the transition point. Solubility could only be determined as an upper bound (<0.01 µg/mL).

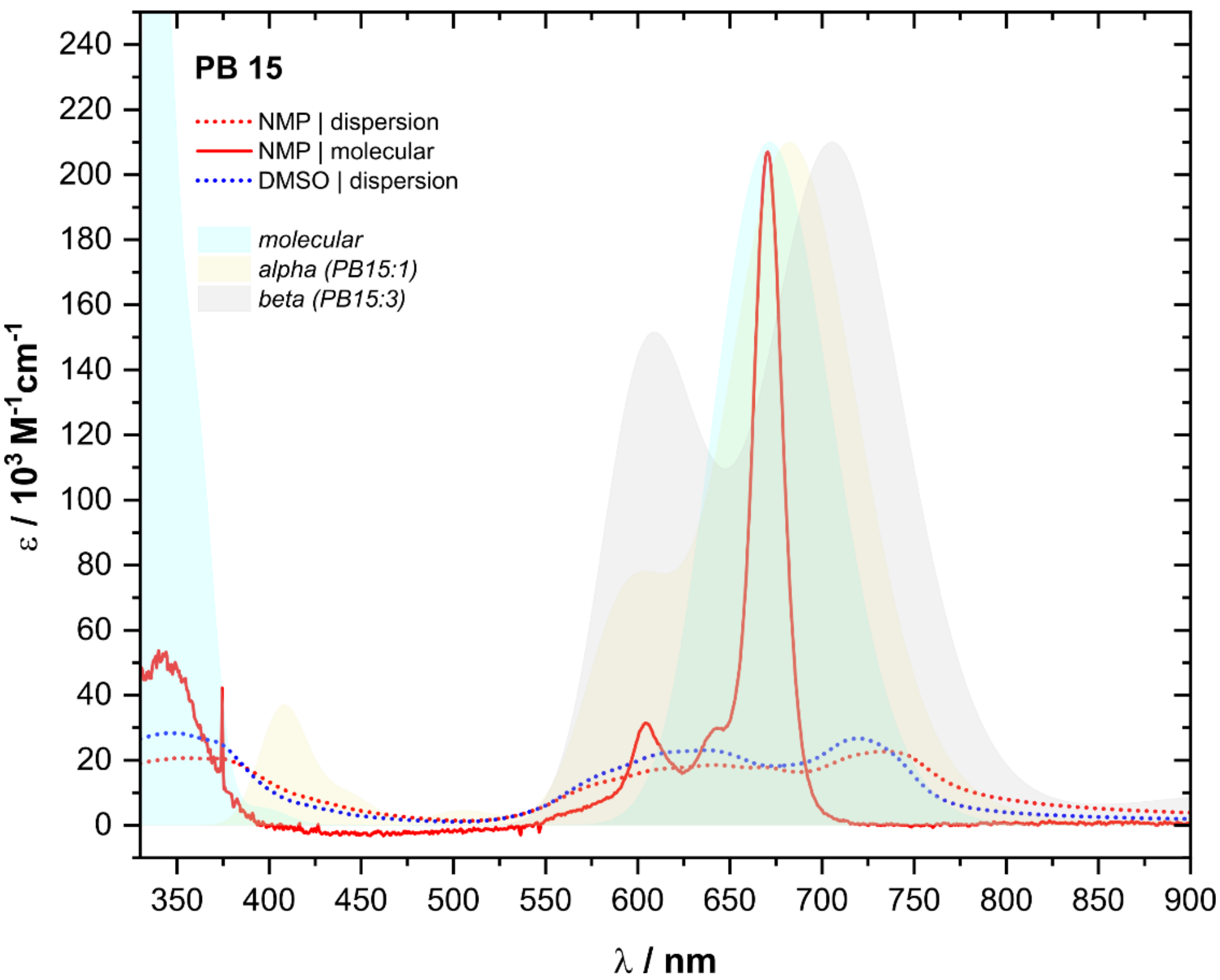


**Figure S73**. Final molecular UV-vis spectrum of PB15 in NMP (solid line), contrasted with corresponding dispersion spectra and normalized TDDFT simulations. TDDFT simulations were shifted by -0.50 eV, respectively; peak type is Gaussian, 0.2 eV FWHM.

### 2.5.2 Pigment Green 7 (PG7)

Due to the high molecular weight of PG7 no TDDFT study was feasible with the computational resources available. A geometry optimization of the PG7 molecule and a TDDFT analysis were thus not performed.

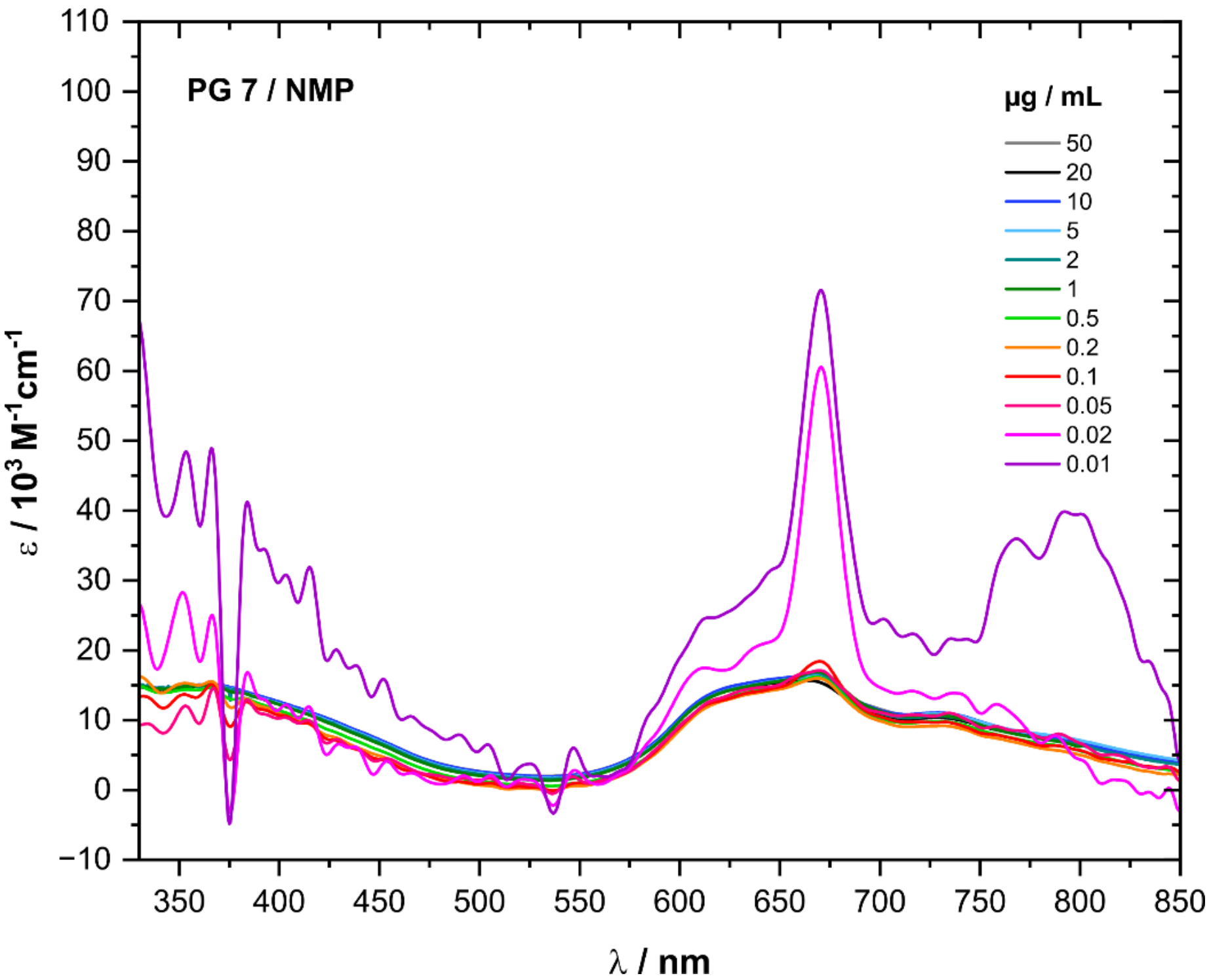


**Figure S74**. Iterative dilution procedure of PG7 in NMP, measured in a 2 cm quartz cuvette. The measurement sequence starts with a dispersion (50 µg/mL) and tracks the UV-vis spectral evolution down to 0.01 µg/mL. Instrumental detection limits reached before the transition point. Solubility could only be determined as an upper bound (<0.01 µg/mL).

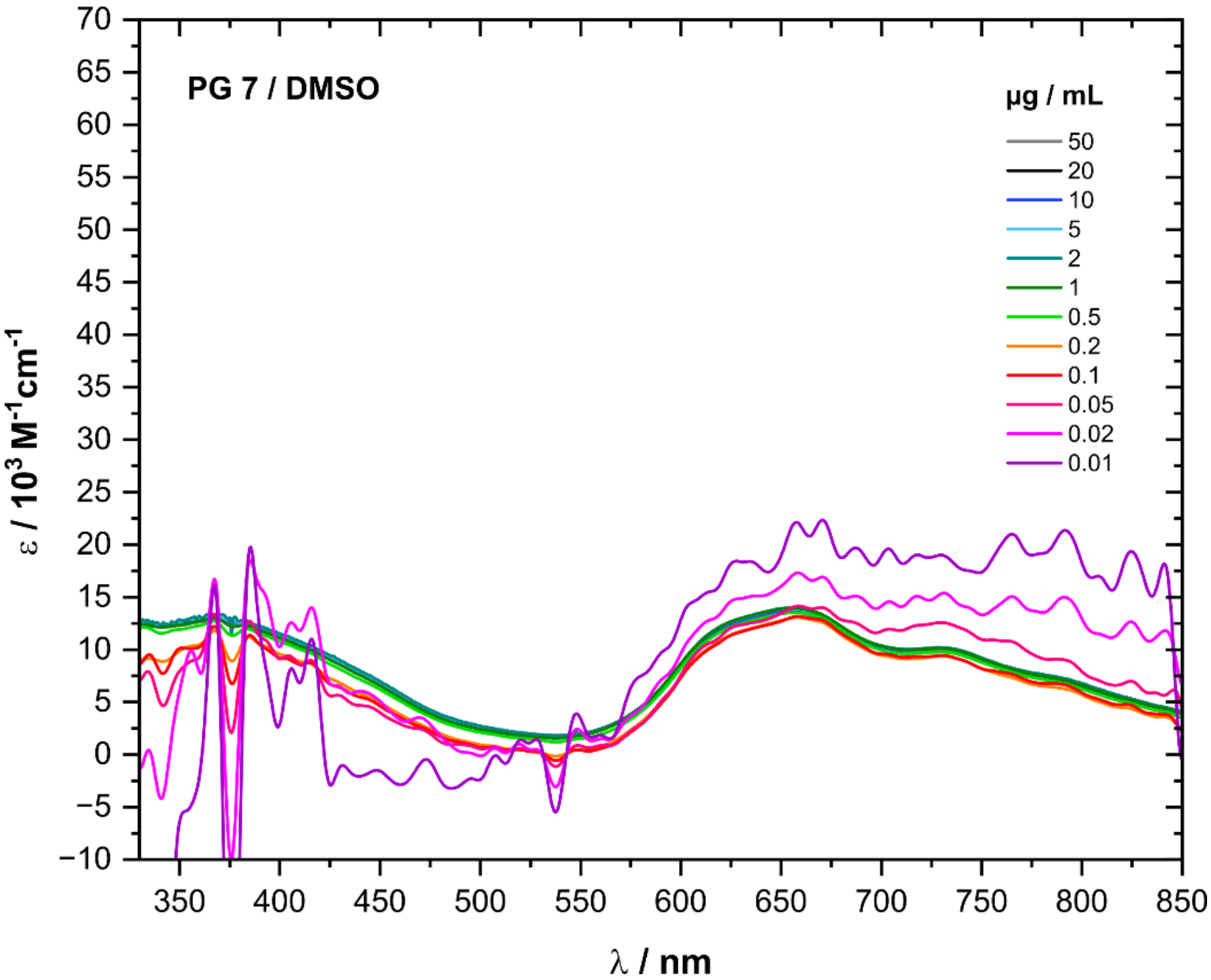


**Figure S75**. Iterative dilution procedure of PG7 in DMSO, measured in a 2 cm quartz cuvette. The measurement sequence starts with a dispersion (50 µg/mL) and tracks the UV-vis spectral evolution down to 0.01 µg/mL. Instrumental detection limits reached before the transition point. Solubility could only be determined as an upper bound (<0.01 µg/mL).

## 2.6 Quinophthalone and Anthraquinone Pigments

### 2.6.1 Pigment Yellow 138 (PY138)

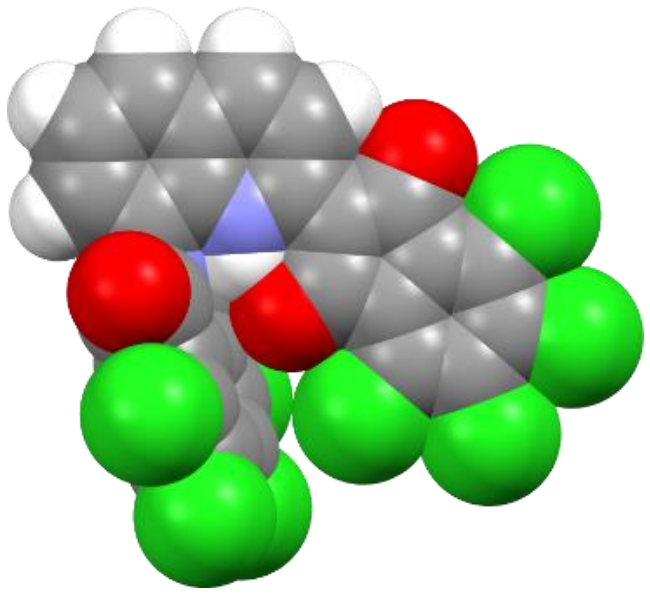

**Figure S76**. Molecular structure of PY138 after geometrical optimization in vacuo (DFT: r$^2$SCAN-3c).

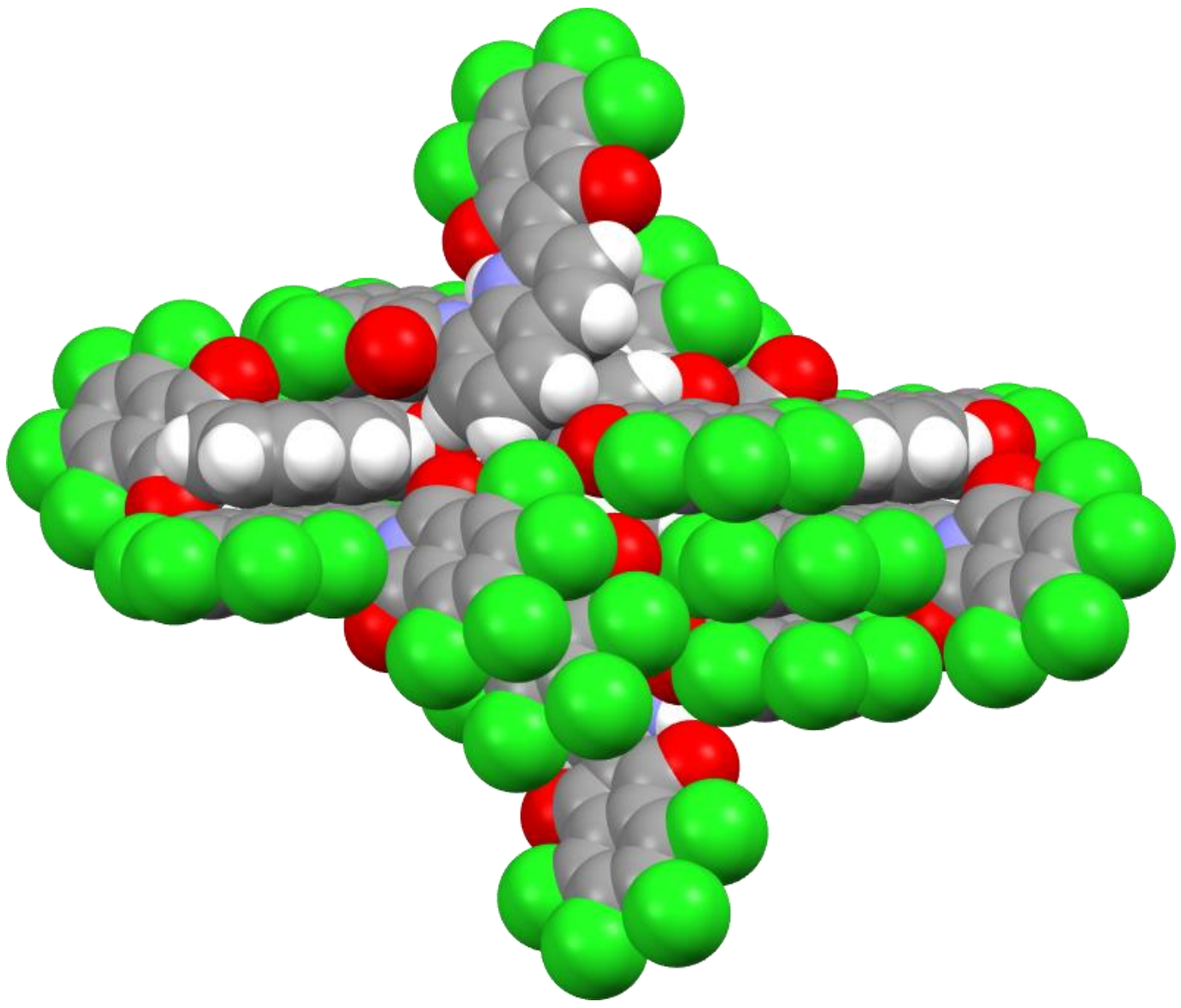

**Figure S77**. Pigment molecule ensemble (PY138) extracted from respective literature-known crystal structure CCDC-1421397.[21] Depicted cut-out (8 pigment molecules) was used for TDDFT simulations, see Computational Details.

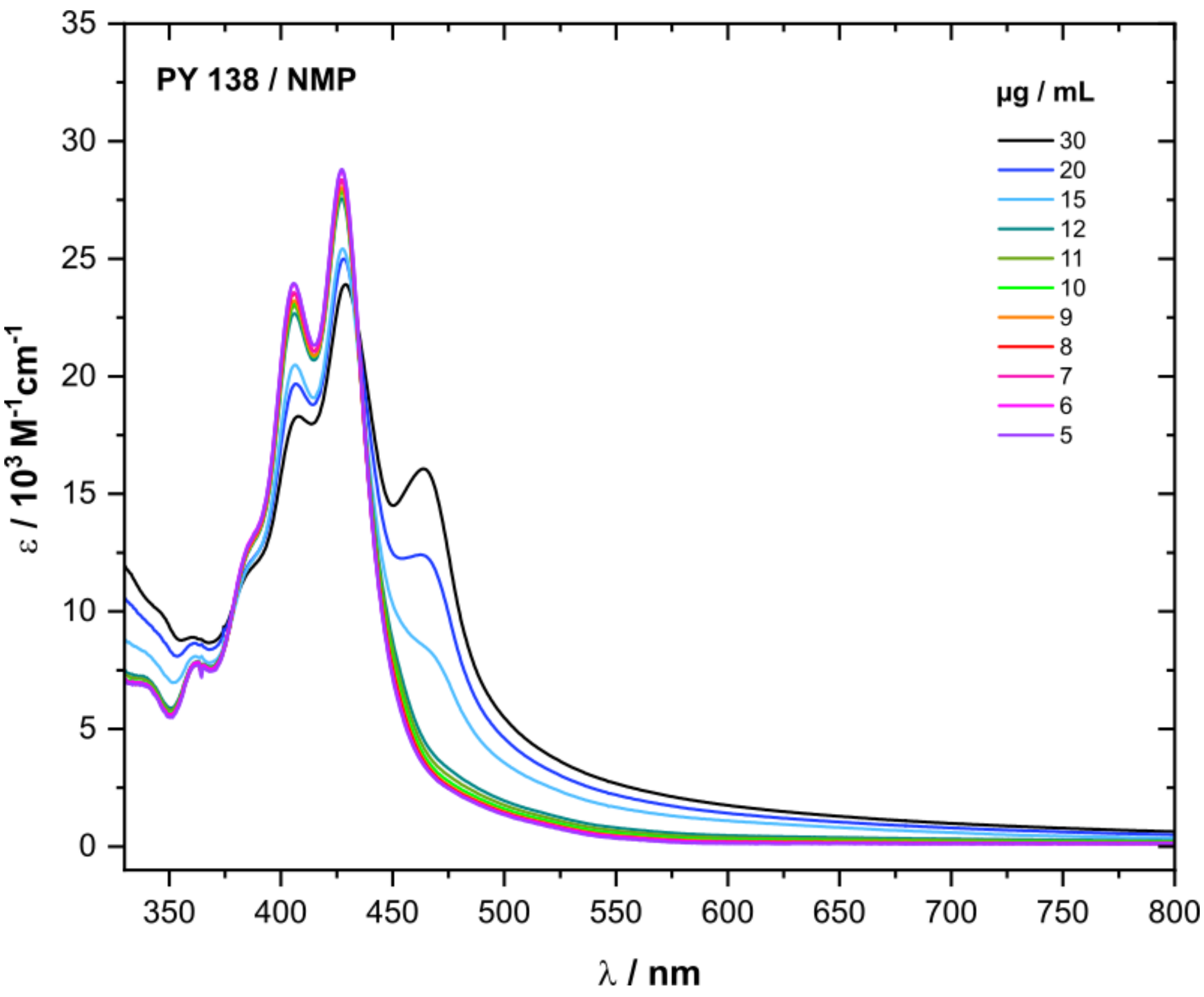


**Figure S78**. Iterative dilution procedure of PY138 in NMP, measured in 1 and 2 cm quartz cuvettes. The measurement sequence starts with a dispersion (30 µg/mL) and tracks the UV-vis spectral evolution down to 5 µg/mL.

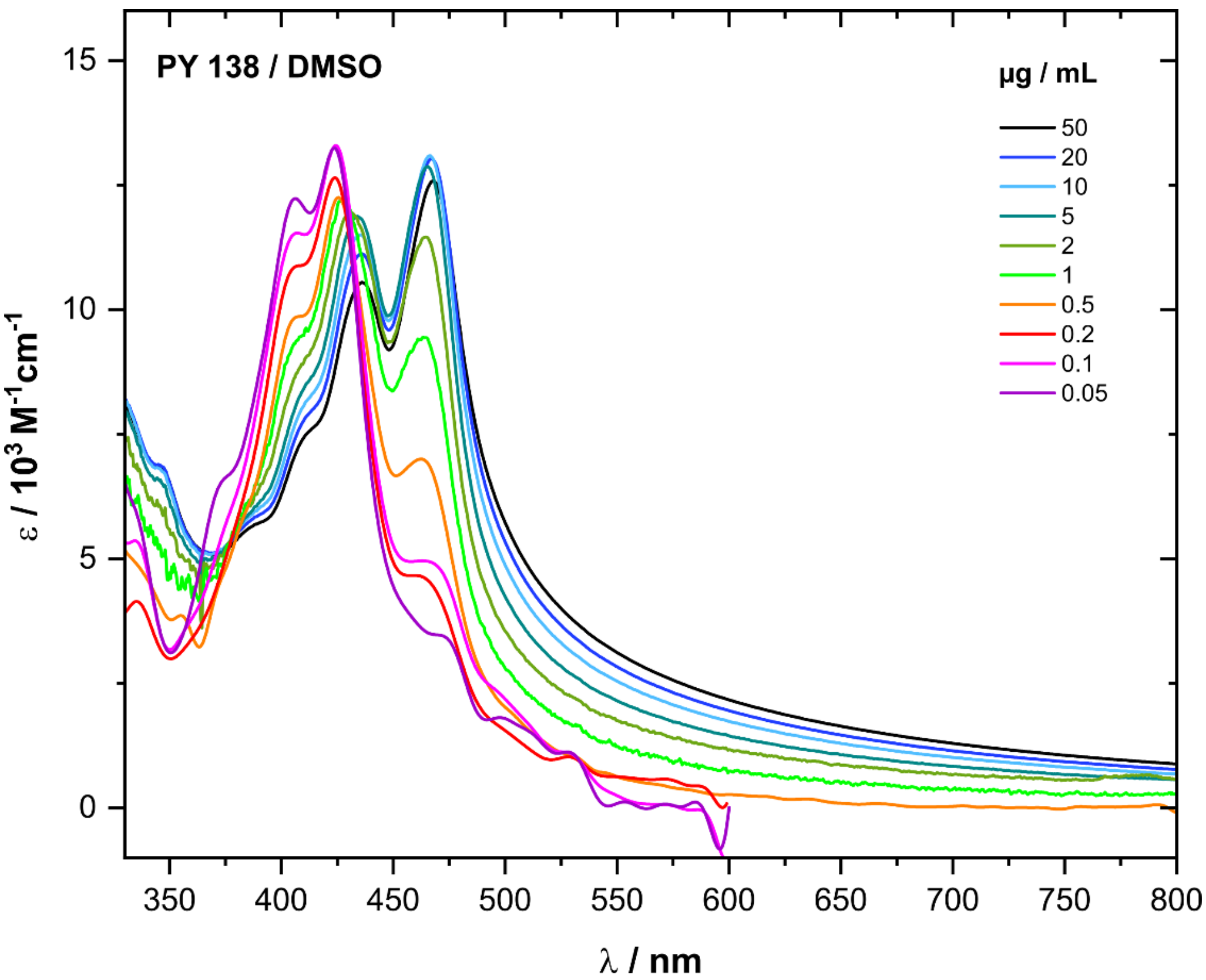


**Figure S79**. Iterative dilution procedure of PY138 in DMSO, measured in a 2 cm quartz cuvette. The measurement sequence starts with a dispersion (50 µg/mL) and tracks the UV-vis spectral evolution down to 0.05 µg/mL. Due to the low concentrations the sample reaches the instrumental detection limits before the transition point. Solubility could only be determined as an upper bound (<0.05 µg/mL).

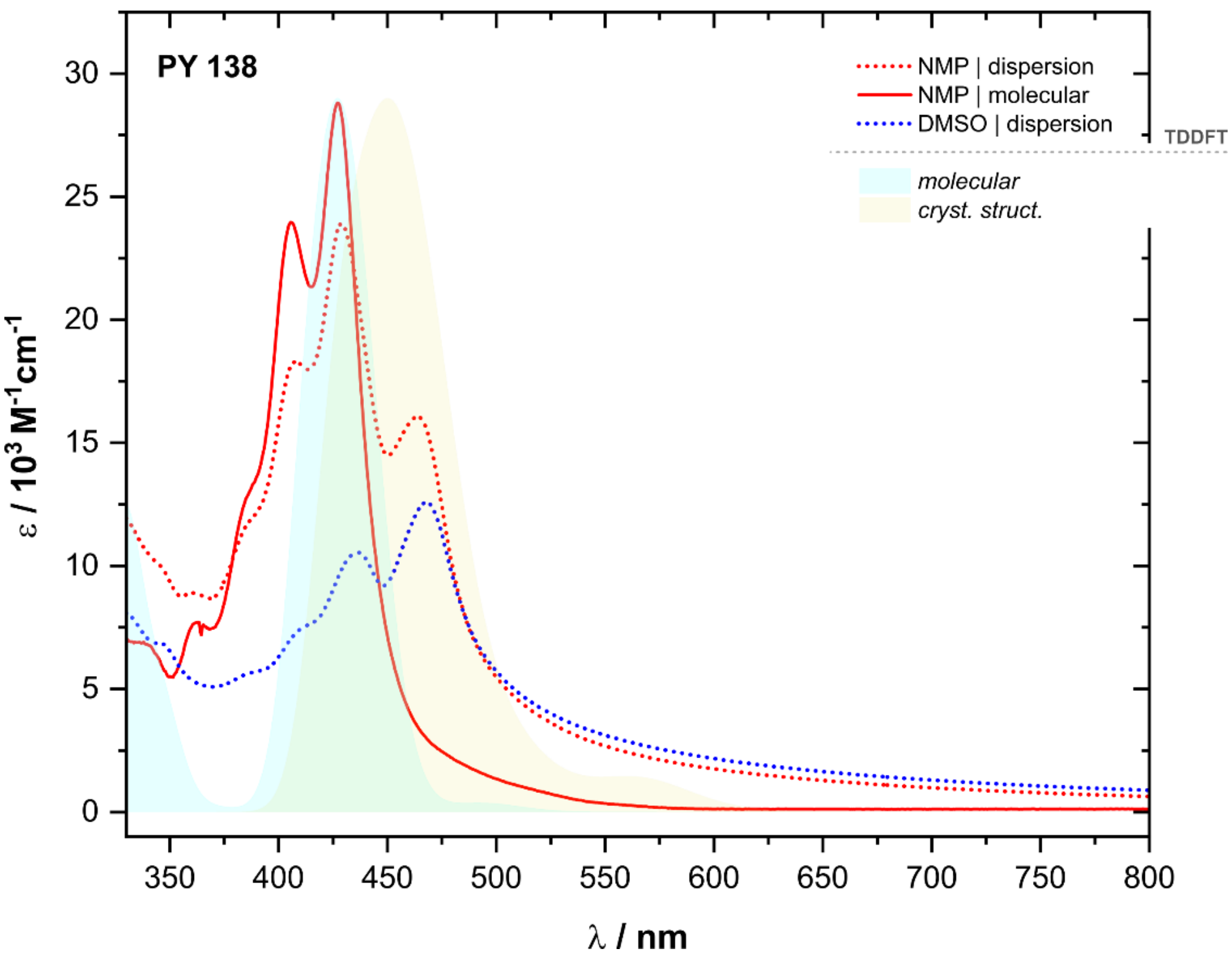


**Figure S80**. Final molecular UV-vis spectrum of PY138 in NMP (solid line), contrasted with corresponding dispersion spectra and normalized TDDFT simulations. TDDFT simulations were shifted by -0.40 eV, respectively; peak type is Gaussian, 0.2 eV FWHM.

### 2.6.2 Pigment Blue 60 (PB60)

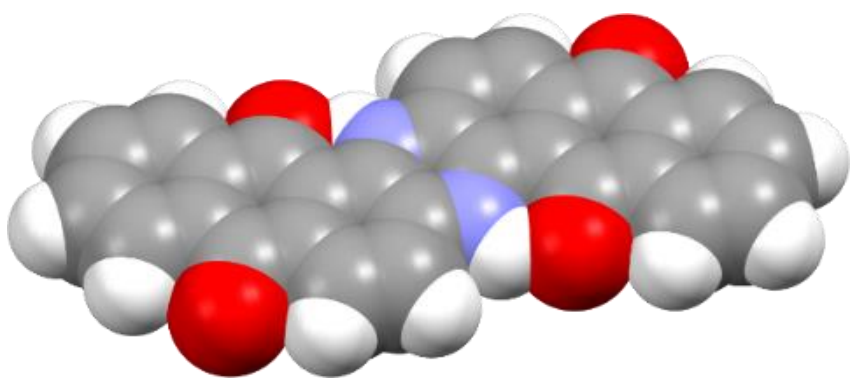

**Figure S81**. Molecular structure of PB60 after geometrical optimization in vacuo (DFT: $r^2$SCAN-3c).

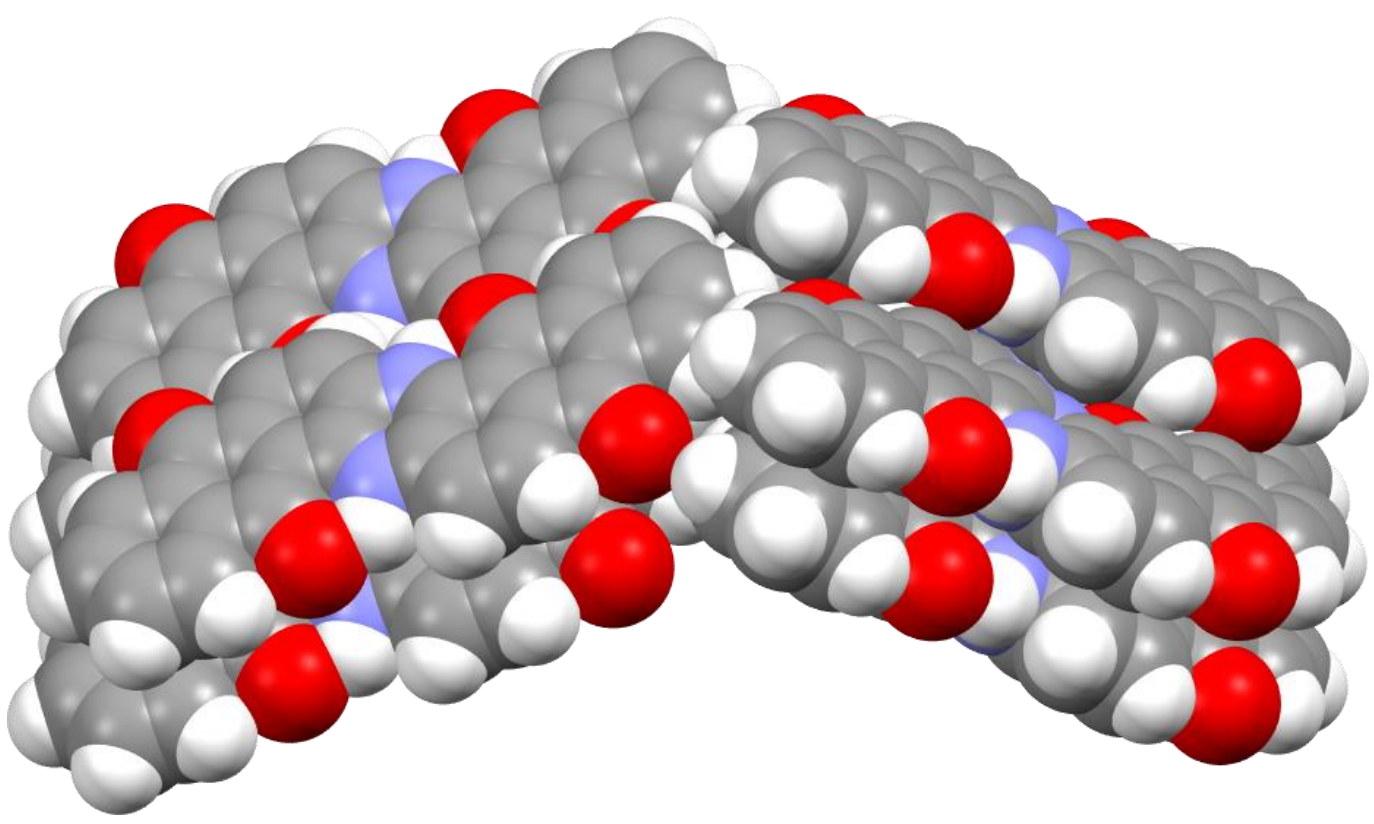

**Figure S82**. Pigment molecule ensemble (PB 60) extracted from respective literature-known crystal structure CCDC-1180353.[22] Depicted cut-out (8 pigment molecules) was used for TDDFT simulations, see Computational Details.

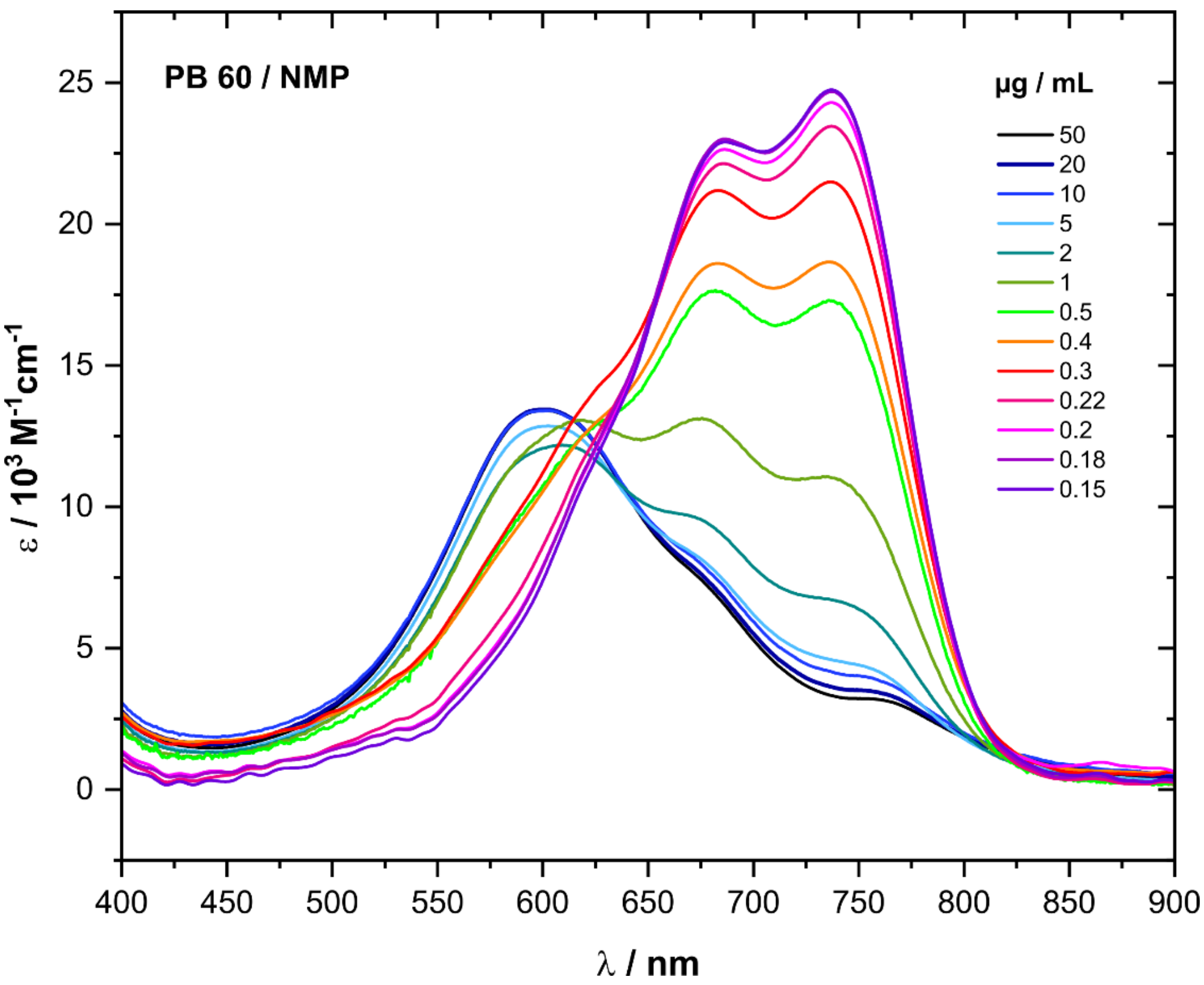


**Figure S83**. Iterative dilution procedure of PB60 in NMP, measured in a 2 cm quartz cuvette. The measurement sequence starts with a dispersion (50 μg/mL) and tracks the UV-vis spectral evolution down to 0.15 μg/mL.

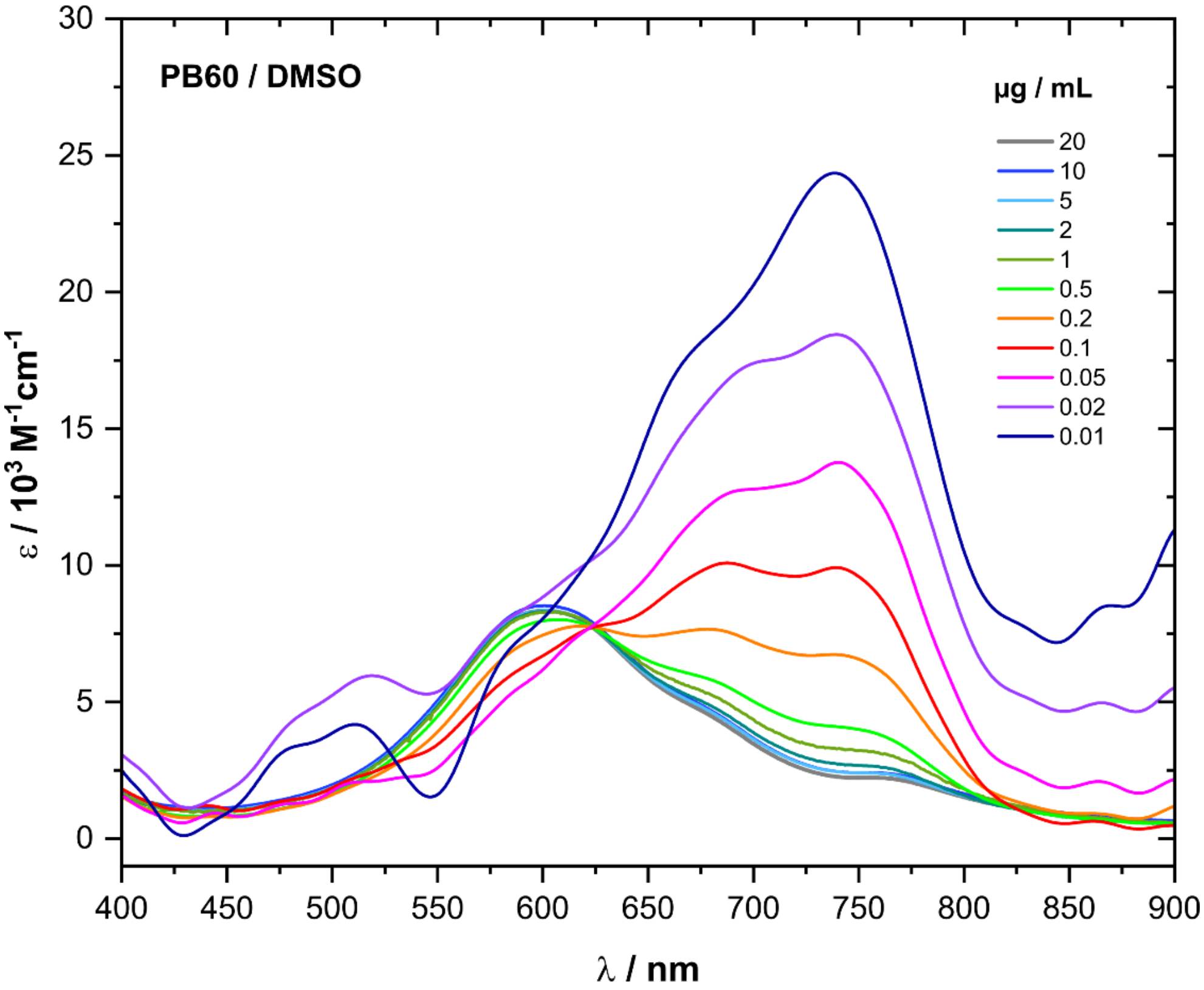


**Figure S84**. Iterative dilution procedure of PB60 in DMSO, measured in a 2 cm quartz cuvette. The measurement sequence starts with a dispersion (20 μg/mL) and tracks the UV-vis spectral evolution down to 0.01 μg/mL. Due to the low concentrations the sample reaches the instrumental detection limits before the transition point. Solubility could only be determined as an upper bound (<0.01 μg/mL).

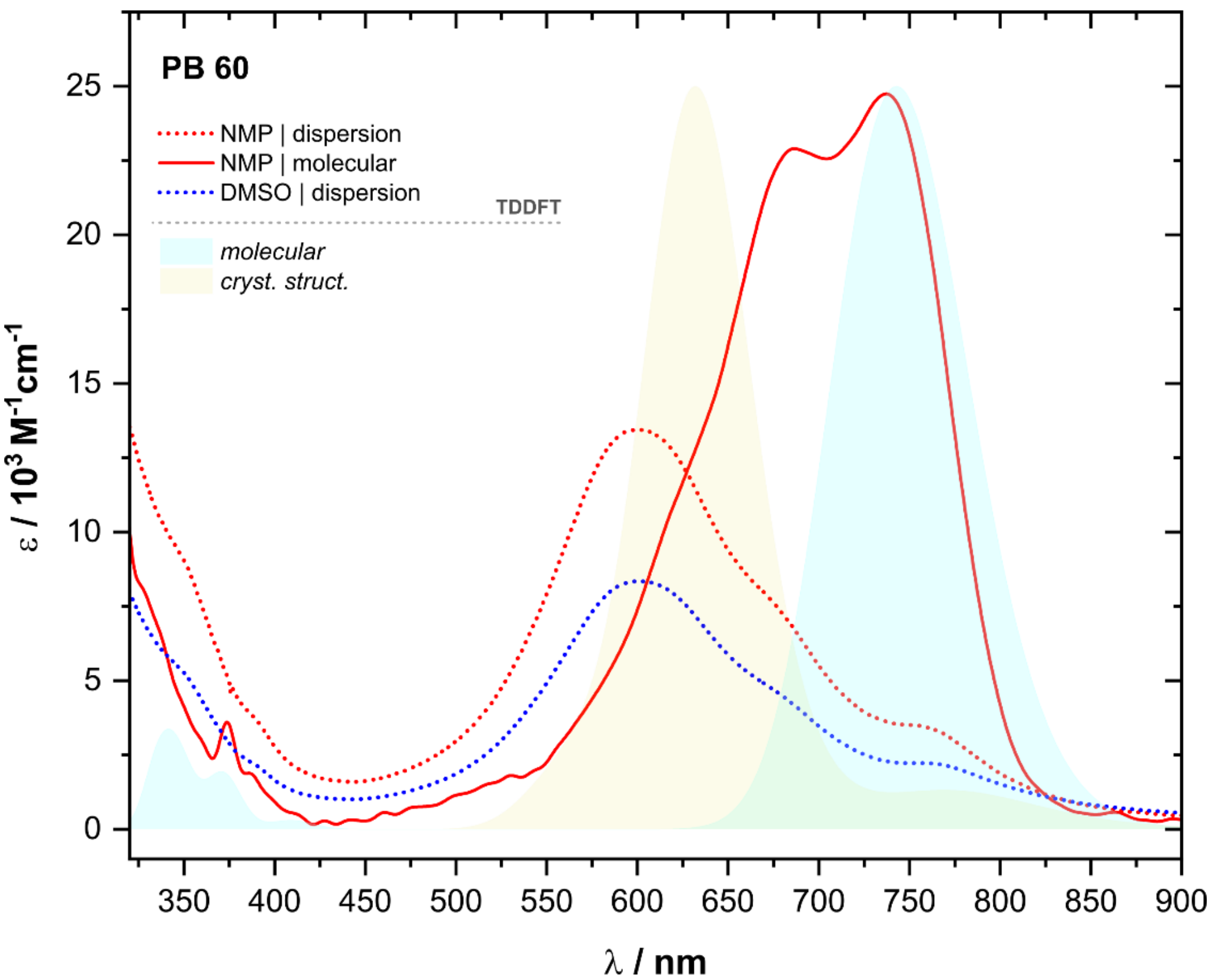


**Figure S85**. Final molecular UV-vis spectrum of PB60 in NMP (solid line), contrasted with corresponding dispersion spectra and normalized TDDFT simulations. TDDFT simulations were shifted by -0.46 eV, respectively; peak type is Gaussian, 0.2 eV FWHM.

# 3. Solvation Modelling

## 3.1 Solute Descriptors

The following tables present the solute descriptors derived from the group contribution method *SoluteGC* and the machine-learning method *SoluteML*, respectively, provided free-of-charge by the groups of W.H. Green (MIT) and R.H. West (Northeastern University).[25,30] Due to model incompatibility, PB15 and PG7 were parametrized as their copper-free, protonated analogues ($H_2Pc$). This substitution introduces two additional NH groups (increasing the *A* descriptor) while reducing the *E* descriptor, both of which act to overestimate water and cytosol solubilities which is consistent with the conservative upper-bound approach applied throughout this work.

**Table S1. Solute descriptors obtained from the *SoluteGC* model.**

| Pigment | *E* | *S* | *A* | *B* | *L* | *V* |
|---|---|---|---|---|---|---|
| **PR170** | 3.5527 | 3.4402 | 1.2266 | 1.7545 | 19.0288 | 3.3696 |
| **PR266** | 3.595 | 3.4768 | 1.2266 | 1.7308 | 18.6188 | 3.2287 |
| **PR22** | 3.4215 | 2.9262 | 0.8093 | 1.2011 | 17.2737 | 3.0878 |
| **PY151** | 2.7463 | 2.8847 | 1.7157 | 1.6606 | 15.0283 | 2.6385 |
| **PY138** | 4.7994 | 4.4638 | 0.1657 | 1.1898 | 23.0323 | 3.889 |
| **PR202** | 3.5837 | 3.0666 | 0.3783 | 0.9508 | 16.3589 | 2.4724 |
| **PR122** | 3.3316 | 2.884 | 0.3783 | 1.0583 | 15.9697 | 2.5094 |
| **PV19** | 3.3124 | 2.8692 | 0.3783 | 1.0133 | 15.1214 | 2.2276 |
| **PO13** | 4.5618 | 3.3266 | 0 | 2.3094 | 23.0376 | 4.3524 |
| **PY14** | 3.9327 | 5.0184 | 0.8256 | 2.539 | 24.9359 | 4.7692 |
| **PY83** | 4.4332 | 6.1342 | 0.8256 | 3.0595 | 29.0517 | 5.5306 |
| **PR264** | 3.9532 | 3.2183 | 0.4378 | 1.7331 | 18.2237 | 3.313 |
| **PR254** | 2.7723 | 2.4225 | 0.4378 | 1.3771 | 13.6904 | 2.3422 |
| **PR255** | 2.501 | 2.225 | 0.4378 | 1.4396 | 12.4529 | 2.0974 |
| **PO73** | 2.5118 | 2.2599 | 0.4378 | 1.6264 | 15.9764 | 3.2246 |
| **PB60** | 4.2577 | 3.6521 | 0.3349 | 1.492 | 19.0354 | 3.04 |
| **PB15** (no Cu) | 5.6083 | 4.4065 | 0.2114 | 2.4321 | 23.5282 | 3.6214 |
| **PG7** (no Cu) | 7.7559 | 6.0388 | 0.2114 | 1.9653 | 34.1439 | 5.5798 |

**Table S2. Solute descriptors obtained from the *SoluteML* model.**

| Pigment | *E* | *S* | *A* | *B* | *L* | *V* |
|---|---|---|---|---|---|---|
| **PR170** | 3.3402 | 2.9684 | 0.6974 | 1.9165 | 19.6185 | 3.3696 |
| **PR266** | 3.3395 | 2.9721 | 0.6609 | 1.8632 | 19.1496 | 3.2287 |
| **PR22** | 3.2474 | 2.8004 | 0.4747 | 1.2868 | 18.7171 | 3.0878 |
| **PY151** | 3.0961 | 2.9476 | 1.3847 | 1.8886 | 16.037 | 2.6385 |
| **PY138** | 3.7462 | 3.6197 | 0.1945 | 1.4643 | 20.4428 | 3.889 |
| **PR202** | 3.4757 | 2.7574 | 0.4228 | 1.1713 | 15.4329 | 2.4724 |
| **PR122** | 3.2027 | 2.6822 | 0.3531 | 1.3783 | 15.1958 | 2.5094 |
| **PV19** | 3.1448 | 2.5889 | 0.416 | 1.2984 | 14.0371 | 2.2276 |
| **PO13** | 3.7983 | 3.2335 | 0.1121 | 1.7694 | 22.7963 | 4.3524 |
| **PY14** | 3.5903 | 3.6259 | 0.478 | 2.2104 | 23.5872 | 4.7692 |
| **PY83** | 3.6456 | 3.8833 | 0.4166 | 2.6611 | 24.3605 | 5.5306 |
| **PR264** | 4.0113 | 3.2544 | 0.4561 | 1.5977 | 19.9176 | 3.313 |
| **PR254** | 2.8012 | 2.8312 | 0.6266 | 1.2411 | 13.309 | 2.3422 |
| **PR255** | 2.5742 | 2.6763 | 0.6587 | 1.3695 | 12.0151 | 2.0974 |
| **PO73** | 2.6171 | 2.7875 | 0.4158 | 1.6068 | 16.7887 | 3.2246 |
| **PB60** | 4.1962 | 3.4003 | 0.4124 | 1.7517 | 20.1003 | 3.04 |
| **PB15** (no Cu) | 4.9876 | 3.331 | 0.4309 | 1.7346 | 23.2695 | 3.6214 |
| **PG7** (no Cu) | 4.6412 | 3.3249 | 0.6057 | 1.5816 | 23.1895 | 5.5798 |

## 3.2 Solvent Descriptors

**Table S3. Solvent descriptors used for solubility transformations.**[26]

**NMP**

| *c* | *e* | *s* | *a* | *b* | *v* |
|---|---|---|---|---|---|
| 0.147 | 0.532 | 0.225 | 0.84 | -4.794 | 3.674 |

**DMSO**

| *c* | *e* | *s* | *a* | *b* | *v* |
|---|---|---|---|---|---|
| -0.194 | 0.327 | 0.791 | 1.26 | -4.54 | 3.361 |

## 3.3 Tissue Descriptors

**Table S4. Tissue descriptors used for water-to-cytosol transformations.**

| *c* | *e* | *s* | *a* | *b* | *v* | |
|---|---|---|---|---|---|---|
| 0.14 | 0.36 | -0.26 | 0.37 | -3.23 | 2.82 | **BSA/water** [27] |
| -0.65 | 0.51 | -0.51 | 0.26 | -2.98 | 3.01 | **Other Protein/water** [28] |
| 0.29 | 0.74 | -0.72 | 0.11 | -3.63 | 3.3 | **Phospholipid/water** [28] |
| -0.07 | 0.7 | -1.08 | -1.72 | -4.14 | 4.11 | **Storage lipid/water** [28] |

## 3.4 *SoluteGC-SoluteML* Correlation Plots

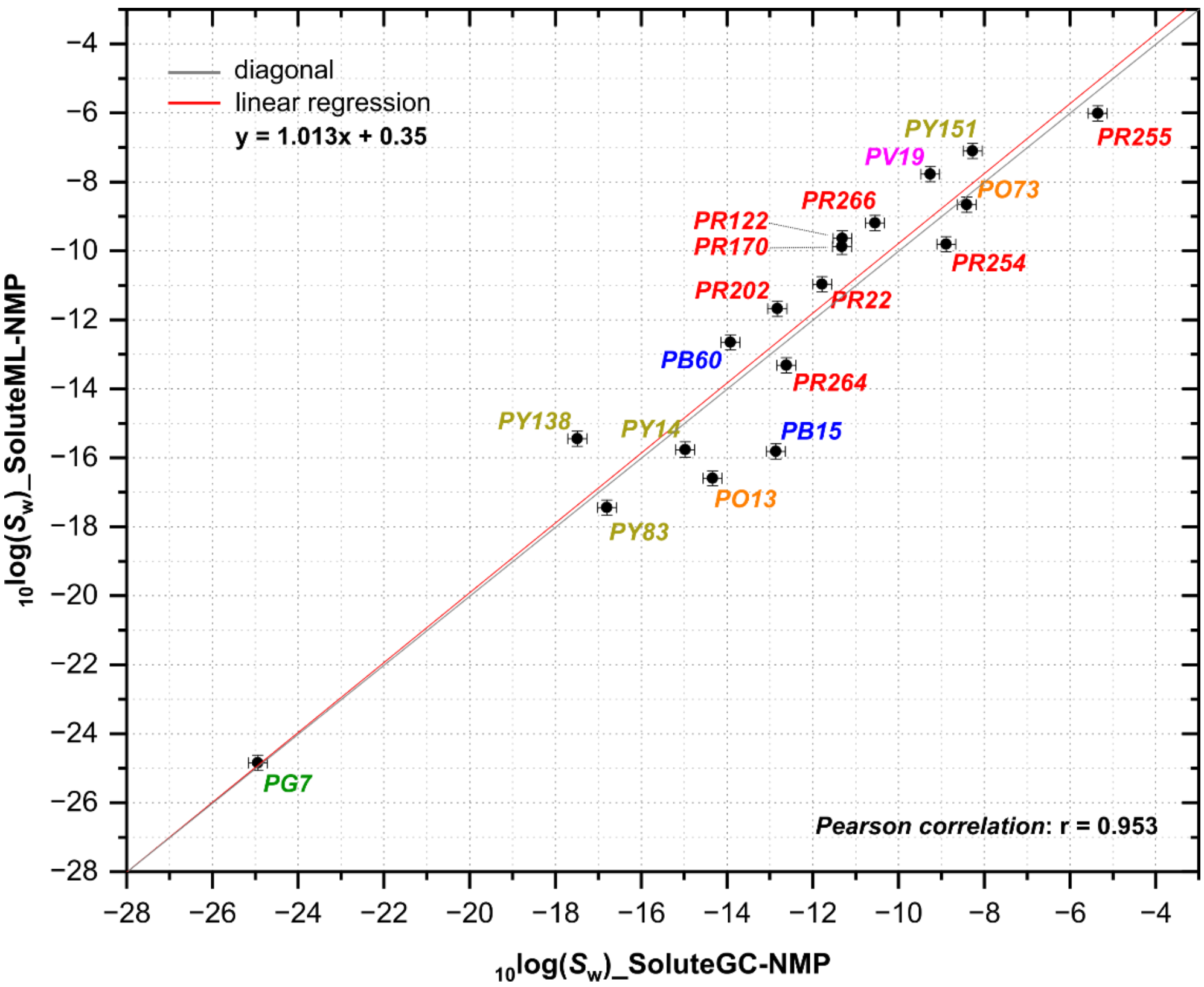


**Figure S86**. Correlation plot displaying logarithmic water solubilities $S_w$ from NMP-to-water transformations employing *SoluteGC*- and *SoluteML*-derived solute descriptors, respectively. Error bars represent combined uncertainties as detailed in Chapter 4.1. Values for PG7 have upper bound quality.

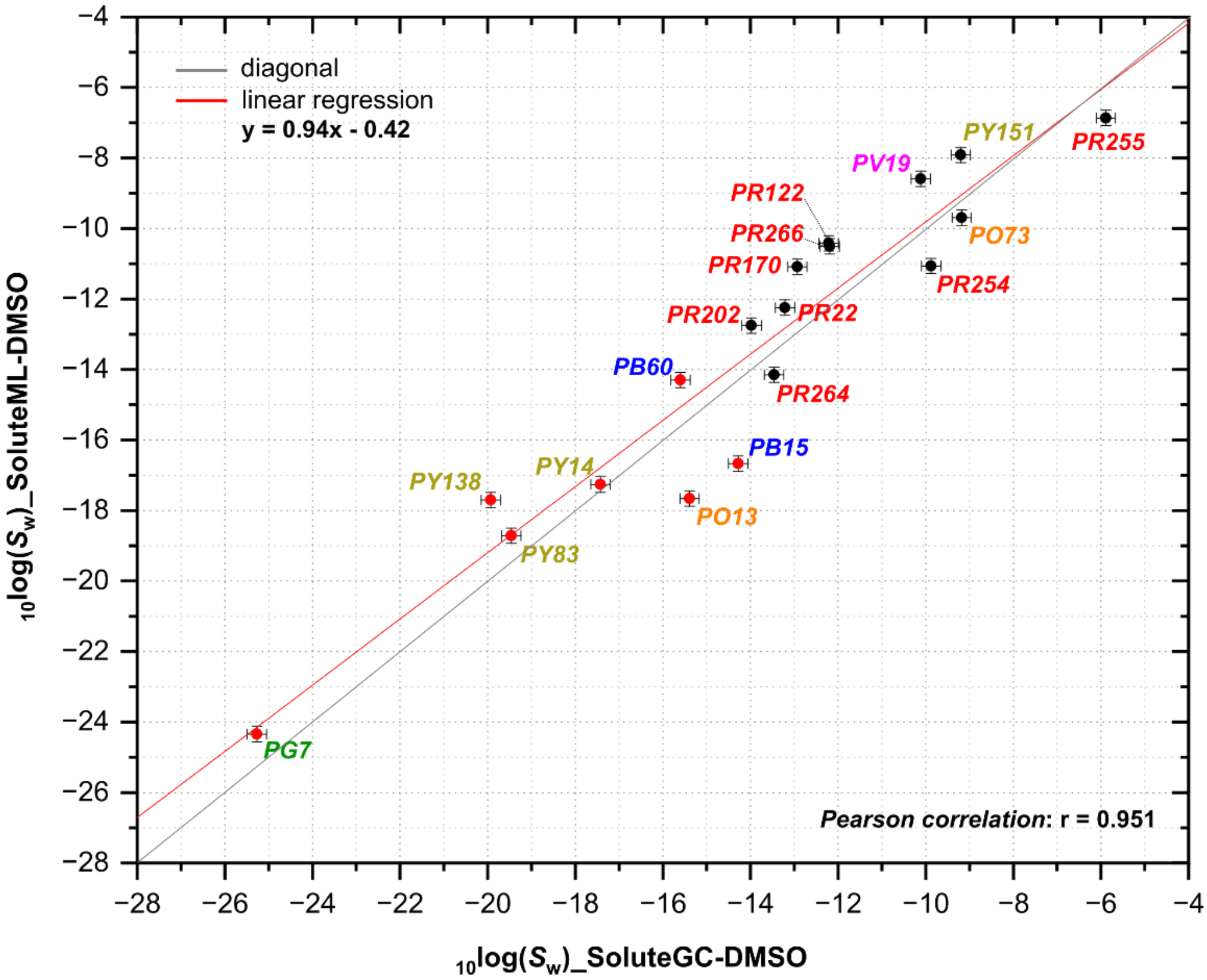


**Figure S87**. Correlation plot displaying logarithmic water solubilities $S_w$ from DMSO-to-water transformations employing *SoluteGC*- and *SoluteML*-derived solute descriptors, respectively. Error bars represent combined uncertainties as detailed in Chapter 4.1. Pigments illustrated with red dots refer to upper bound solubilities.

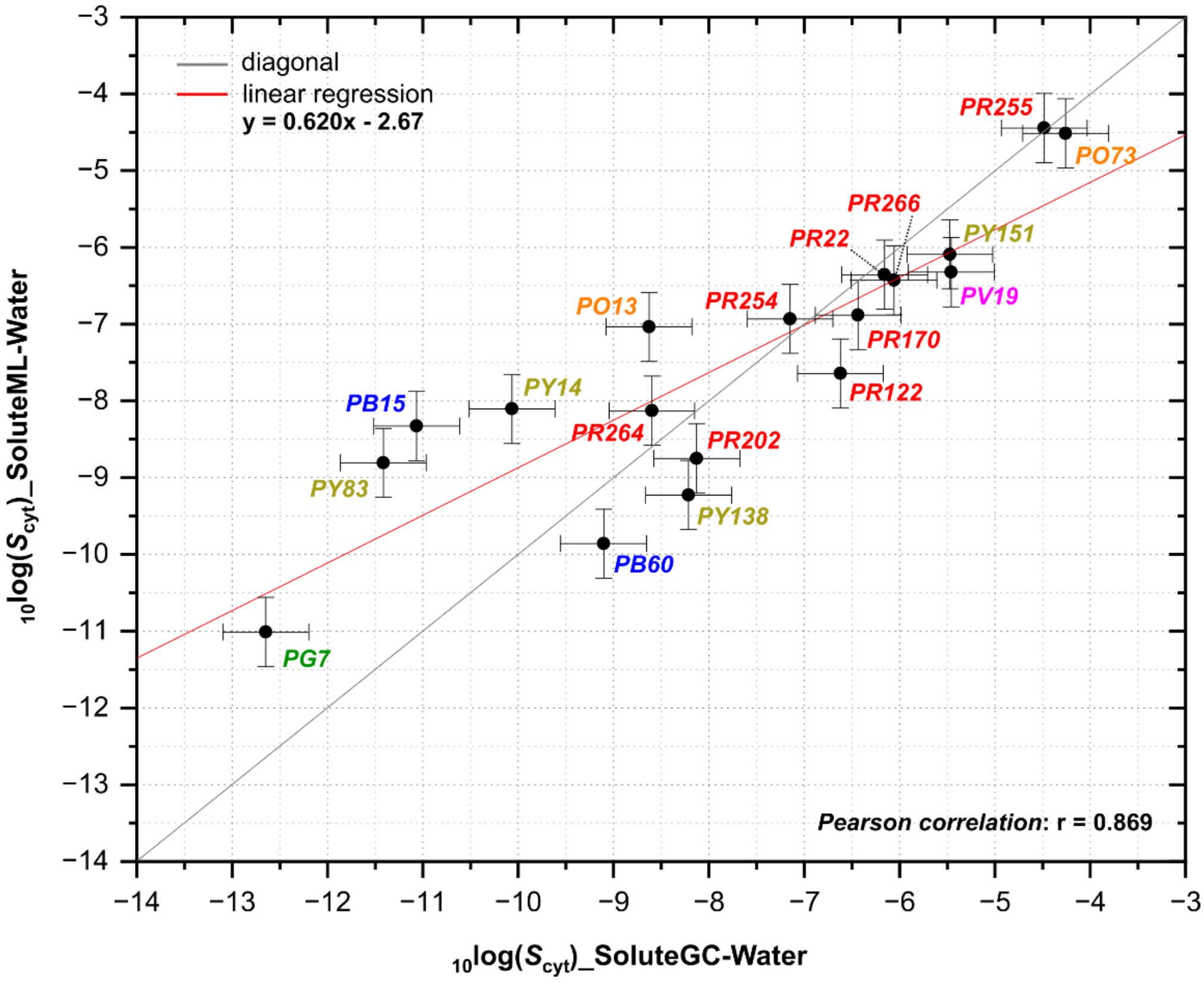


**Figure S88**. Correlation plot displaying logarithmic cytosol solubilities $S_{cyt}$ from water-to-cytosol transformations employing *SoluteGC*- and *SoluteML*-derived solute descriptors, respectively. Error bars represent combined uncertainties as detailed in Chapter 4.1.

## 3.5 *Water Solubilities*

**Table S5. Water solubilities $S_w$ [µM] derived from pp-LFER transformations with two descriptor models (*SoluteGC*, *SoluteML*) using experimental solubilities in NMP and DMSO. *SoluteAV* values are averaged in log space and given with an error range according to Chapter 4.2. NMP values were used for further water-to-cytosol transformations in accordance with the conservative approach. Red values indicate upper-bound values. PB15 and PG7 were modeled without copper due to model incompatibility (see Section 3.1).**

| | *SoluteGC* | | *SoluteML* | | *SoluteAV* | | | |
|---|---|---|---|---|---|---|---|---|
| **Pigment** | NMP $S_w$ [µM] | DMSO $S_w$ [µM] | NMP $S_w$ [µM] | DMSO $S_w$ [µM] | NMP $S_w$ [µM] | NMP error range $\sigma_w$ [µM] | DMSO $S_w$ [µM] | DMSO error range $\sigma_w$ [µM] |
| **PR170** | 4.77E-06 | 1.18E-07 | 1.31E-04 | 8.23E-06 | 2.50E-05 | 4.43E-06 - 1.41E-04 | 9.84E-07 | 1.11E-07 - 8.71E-06 |
| **PR266** | 2.82E-05 | 6.08E-07 | 6.46E-04 | 3.81E-05 | 1.35E-04 | 2.61E-05 - 6.97E-04 | 4.81E-06 | 5.74E-07 - 4.04E-05 |
| **PR22** | 1.65E-06 | 6.14E-08 | 1.08E-05 | 5.69E-07 | 4.22E-06 | 1.46E-06 - 1.22E-05 | 1.87E-07 | 5.52E-08 - 6.32E-07 |
| **PY151** | 5.34E-03 | 6.30E-04 | 7.92E-02 | 1.22E-02 | 2.06E-02 | 4.89E-03 - 8.65E-02 | 2.78E-03 | 5.81E-04 - 1.33E-02 |
| **PY138** | 3.23E-12 | 1.18E-14 | 3.55E-10 | 1.97E-12 | 3.39E-11 | 3.06E-12 - 3.74E-10 | 1.53E-13 | 1.13E-14 - 2.07E-12 |
| **PR202** | 1.49E-07 | 1.05E-08 | 2.10E-06 | 1.77E-07 | 5.60E-07 | 1.37E-07 - 2.29E-06 | 4.32E-08 | 9.68E-09 - 1.92E-07 |
| **PR122** | 4.91E-06 | 6.44E-07 | 2.29E-04 | 3.13E-05 | 3.36E-05 | 4.61E-06 - 2.44E-04 | 4.48E-06 | 6.05E-07 - 3.33E-05 |
| **PV19** | 5.47E-04 | 7.70E-05 | 1.68E-02 | 2.57E-03 | 3.03E-03 | 5.10E-04 - 1.80E-02 | 4.45E-04 | 7.19E-05 - 2.75E-03 |
| **PO13** | 4.60E-09 | 4.07E-10 | 2.55E-11 | 2.19E-12 | 3.42E-10 | 2.43E-11 - 4.82E-09 | 2.98E-11 | 2.09E-12 - 4.26E-10 |
| **PY14** | 1.06E-09 | 3.78E-12 | 1.73E-10 | 5.47E-12 | 4.28E-10 | 1.52E-10 - 1.20E-09 | 4.55E-12 | 2.68E-12 - 7.72E-12 |
| **PY83** | 1.58E-11 | 3.48E-14 | 3.61E-12 | 1.93E-13 | 7.54E-12 | 3.10E-12 - 1.84E-11 | 8.20E-14 | 3.05E-14 - 2.21E-13 |
| **PR264** | 2.43E-07 | 3.45E-08 | 4.81E-08 | 7.11E-09 | 1.08E-07 | 4.18E-08 - 2.79E-07 | 1.57E-08 | 6.16E-09 - 3.98E-08 |
| **PR254** | 1.30E-03 | 1.33E-04 | 1.57E-04 | 8.62E-06 | 4.51E-04 | 1.40E-04 - 1.45E-03 | 3.39E-05 | 7.90E-06 - 1.45E-04 |
| **PR255** | 4.41E+00 | 1.29E+00 | 9.61E-01 | 1.36E-01 | 2.06E+00 | 8.30E-01 - 5.11E+00 | 4.20E-01 | 1.23E-01 - 1.44E+00 |
| **PO73** | 3.89E-03 | 6.64E-04 | 2.19E-03 | 2.04E-04 | 2.91E-03 | 1.64E-03 - 5.17E-03 | 3.68E-04 | 1.70E-04 - 7.95E-04 |
| **PB60** | 1.20E-08 | 2.52E-10 | 2.22E-07 | 5.04E-09 | 5.16E-08 | 1.10E-08 - 2.41E-07 | 1.13E-09 | 2.33E-10 - 5.45E-09 |
| **PB15** (no Cu) | 1.38E-07 | 5.22E-09 | 1.53E-10 | 2.13E-11 | 4.59E-09 | 1.47E-10 - 1.43E-07 | 3.34E-10 | 2.04E-11 - 5.46E-09 |
| **PG7** (no Cu) | 1.15E-19 | 5.39E-20 | 1.44E-19 | 4.55E-19 | 1.28E-19 | 7.72E-20 - 2.13E-19 | 1.57E-19 | 4.83E-20 - 5.08E-19 |

## 3.6 *Cytosol Solubilities*

**Table S6. Cytosol solubilities [µM] derived from pp-LFER transformations with two descriptor models (*SoluteGC*, *SoluteML*) using the NMP-to-water values (*SoluteAV*) from Table S5. The Max column presents the final, conservative solubilities depicted in Figure 16 as highest values of both models. The choice of highest values and the transformation from log to linear space result in asymmetric uncertainties expressed via an error range $\sigma_{cyt}$. Red values indicate upper-bound values. PB15 and PG7 were modeled without copper due to model incompatibility (see Section 3.1).**

| | *SoluteGC* | *SoluteML* | *Max* | Error range |
|---|---|---|---|---|
| **Pigment** | $S_{cyt}$ [µM] | $S_{cyt}$ [µM] | $S_{cyt}$ [µM] | $\sigma_{cyt}$ [µM] |
| **PR170** | 3.67E-01 | 1.31E-01 | 3.67E-01 | 4.01E-02 - 2.60E+00 |
| **PR266** | 8.74E-01 | 3.73E-01 | 8.74E-01 | 1.11E-01 - 5.74E+00 |
| **PR22** | 6.96E-01 | 4.41E-01 | 6.96E-01 | 1.59E-01 - 2.83E+00 |
| **PY151** | 3.37E+00 | 8.11E-01 | 3.37E+00 | 3.65E-01 - 1.86E+01 |
| **PY138** | 6.13E-03 | 5.95E-04 | 6.13E-03 | 1.90E-04 - 8.03E-02 |
| **PR202** | 7.46E-03 | 1.78E-03 | 7.46E-03 | 8.17E-04 - 4.02E-02 |
| **PR122** | 2.40E-01 | 2.28E-02 | 2.40E-01 | 9.64E-03 - 2.14E+00 |
| **PV19** | 3.48E+00 | 4.76E-01 | 3.48E+00 | 2.06E-01 - 2.59E+01 |
| **PO13** | 2.36E-03 | 9.20E-02 | 9.20E-02 | 9.15E-04 - 1.51E+00 |
| **PY14** | 8.63E-05 | 7.88E-03 | 7.88E-03 | 7.01E-05 - 3.15E-02 |
| **PY83** | 3.85E-06 | 1.56E-03 | 1.56E-03 | 3.37E-06 - 5.59E-03 |
| **PR264** | 2.53E-03 | 7.47E-03 | 7.47E-03 | 1.35E-03 - 2.81E-02 |
| **PR254** | 7.11E-02 | 1.17E-01 | 1.17E-01 | 2.44E-02 - 5.18E-01 |
| **PR255** | 3.29E+01 | 3.59E+01 | 3.59E+01 | 9.80E+00 - 1.31E+02 |
| **PO73** | 5.53E+01 | 3.05E+01 | 5.53E+01 | 1.60E+01 - 1.64E+02 |
| **PB60** | 7.89E-04 | 1.38E-04 | 7.89E-04 | 6.46E-05 - 4.76E-03 |
| **PB15** (no Cu) | 8.62E-06 | 4.70E-03 | 4.70E-03 | 3.38E-06 - 1.65E-01 |
| **PG7** (no Cu) | 2.26E-07 | 9.75E-06 | 9.75E-06 | 1.96E-07 - 2.79E-05 |

## 4. Uncertainty Analysis of pp-LFER Solubility Conversions

### 4.1 *Propagated Uncertainties in Descriptor Model Comparisons*

Each predicted water solubility ${}_{10}\log S_w$ carries two independent uncertainty contributions: the experimental reproducibility (±20%, corresponding to $\sigma_{exp}$ = ±0.08 log units) and the pp-LFER calibration standard deviation $SD_w$ from the NMP- and DMSO-to-water system coefficients (SD ≤ 0.2 log units for solvent–water systems)[29]. Propagation in quadrature yields a combined uncertainty of:

$\sigma({}_{10}\log S_w) = \sqrt{(\sigma_{exp}^2 + SD_w^2)} = \sqrt{(0.08^2 + 0.2^2)} \approx 0.22$ log units

This value applies uniformly to all data points in Figures S85 and S86.

Similarly, when the second transformation from water to cytosol is applied, another pp-LFER calibration standard deviation $SD_{bio}$ comes into play which carries an RMSE of 0.32-0.53 log units for biological phases.[28]

$\sigma({}_{10}\log S_{cyt}) = \sqrt{(\sigma_{exp}^2 + SD_w^2 + SD_{bio}^2)} = \sqrt{(0.08^2 + 0.2^2 + 0.4^2)} \approx 0.45$ log units

where $SD_{bio}$ = 0.4 log units (mid-range estimate from Endo et al.).[28]

### 4.2 *Pigment-Specific Uncertainties in Water Solubility Estimates*

In the cross-validation of NMP- and DMSO-derived water solubilities (Figure 15), each axis carries a pigment-specific uncertainty composed of three independent contributions: the experimental reproducibility ($\sigma_{exp}$ = 0.08 log units), the pp-LFER calibration standard deviation ($SD_w$ = 0.2 log units), and the descriptor model divergence, quantified as half the absolute difference between *SoluteGC* and *SoluteML* predictions for each pigment *i* ($\sigma_{i,desc} = |\Delta_{i,w}|/2$, reflecting the reduction by averaging). The combined uncertainty per axis is:

$\sigma_{i,w}({}_{10}\log S_w) = \sqrt{(0.08^2 + 0.2^2 + (\Delta_{i,w}/2)^2)}$

### 4.3 *Pigment-Specific Asymmetric Uncertainties in Cytosolic Solubility Estimates*

Cytosolic solubilities (Figure 16) were derived by combining water solubilities with tissue-composition-based pp-LFER partition coefficients following the model of Endo, Brown and Goss,[28] which carries an RMSE of 0.32-0.53 log units for biological phases. Since the highest values from both descriptor models were selected as a conservative upper bound, asymmetric error bars are applied. The upward uncertainty for each single pigment *i* reflects only the formal propagated contributions:

$\sigma_{i,up} = \sqrt{(\sigma_{i,w}^2({}_{10}\log S_w) + SD_{bio}^2)}$

where $SD_{bio}$ = 0.4 log units (mid-range estimate from Endo et al.).[28] The downward uncertainty additionally includes the full descriptor divergence in the cytosol conversion step:

$\sigma_{i,down} = \sqrt{(\sigma_{i,w}^2({}_{10}\log S_w) + SD_{bio}^2 + (\Delta_{i,cyt})^2)}$

where $\Delta_{i,cyt} = |{}_{10}\log S_{cyt}(\textit{SoluteGC}) - {}_{10}\log S_{cyt}(\textit{SoluteML})|$.

## 4.4 *Solvation and Transfer Enthalpies for Temperature Adjustment (298 K → 310 K)*

**Table S7. Solvation enthalpies in NMP and water predicted by the *DirectML* model for all 18 pigments,[30] along with derived transfer enthalpies $\Delta\Delta H_{solv} = \Delta H_{solv}(H_2O) - \Delta H_{solv}(NMP)$ and the resulting temperature corrections $\Delta \log S_w$ for the 298 K → 310 K shift via the van't Hoff relation: $\Delta \log S_w = \Delta\Delta H_{solv} / (2.303 \cdot R) \times (1/298 - 1/310)$. Reported uncertainties σ represent the epistemic model uncertainties provided by *DirectML* and were propagated to $\Delta \log S_w$ via Gaussian error calculus. All $\Delta \log S_w$ values fall below 0.3 log units and are within the divergence between the *SoluteGC* and *SoluteML* descriptor models used for the primary solubility conversions. PB15 and PG7 were modeled without copper due to model incompatibility.**

| **Pigment** | **$\Delta H_{solv}$(NMP) [kJ]** | error σ ($\Delta H_{solv}$(NMP)) [kJ] | **$\Delta H_{solv}$($H_2O$) [kJ]** | error σ ($\Delta H_{solv}$($H_2O$)) [kJ] | **$\Delta\Delta H_{solv}$ (Transfer) [kJ]** | **$\Delta \log S_w$** | error σ ($\Delta \log S_w$) |
|---|---|---|---|---|---|---|---|
| **PR170** | -157.77 | 11.96 | -141.05 | 15.21 | 16.72 | **0.11** | 0.13 |
| **PR266** | -156.59 | 11.5 | -139.66 | 15.45 | 16.93 | **0.11** | 0.13 |
| **PR22** | -135.76 | 13.14 | -118.22 | 13.13 | 17.54 | **0.12** | 0.13 |
| **PY151** | -141.25 | 16.34 | -133.86 | 16.62 | 7.39 | **0.05** | 0.16 |
| **PY138** | -141.87 | 21.84 | -127.45 | 21.55 | 14.42 | **0.10** | 0.21 |
| **PR202** | -135.19 | 11 | -112.91 | 13.64 | 22.28 | **0.15** | 0.12 |
| **PR122** | -128.58 | 13.76 | -110.89 | 18.08 | 17.69 | **0.12** | 0.15 |
| **PV19** | -127.11 | 11.34 | -108.3 | 15.17 | 18.81 | **0.13** | 0.13 |
| **PO13** | -166.91 | 28.33 | -148.76 | 25.31 | 18.15 | **0.12** | 0.26 |
| **PY14** | -168.35 | 23.46 | -156.5 | 21.89 | 11.85 | **0.08** | 0.22 |
| **PY83** | -204.54 | 46.75 | -197.96 | 48.26 | 6.58 | **0.04** | 0.46 |
| **PR264** | -156.27 | 21.95 | -139.05 | 23.97 | 17.22 | **0.12** | 0.22 |
| **PR254** | -119.74 | 20.59 | -102.58 | 15.72 | 17.16 | **0.12** | 0.18 |
| **PR255** | -114.66 | 26.59 | -98.68 | 19.4 | 15.98 | **0.11** | 0.22 |
| **PO73** | -128.04 | 24.27 | -114.38 | 17.41 | 13.66 | **0.09** | 0.20 |
| **PB60** | -157.37 | 17.63 | -141.07 | 25.21 | 16.3 | **0.11** | 0.21 |
| **PB15** (no Cu) | -172.05 | 35.73 | -162.22 | 30.58 | 9.83 | **0.07** | 0.32 |
| **PG7** (no Cu) | -195.96 | 70.37 | -185.55 | 57.95 | 10.41 | **0.07** | 0.62 |

# 5. DLS Archive

**Experimental Details:** Dynamic light scattering (DLS) measurements were performed on a Malvern Zetasizer Nano ZS equipped with a 633 nm laser in 173° backscatter geometry (NIBS) at 20 °C. Samples were allowed to equilibrate thermally for 1 min prior to each measurement. For each replicate, the instrument automatically collected 10–18 sub-runs which were averaged internally. Three replicates were measured per sample. Samples were prepared at concentrations 30% above and 30% below the solubility limit as determined by UV-vis spectroscopy, referred to as dispersion (D) and solution (Sol) samples, respectively. The discrepancy between the visually apparent and software-reported intercept values, specifically at solution samples, is attributed to the extrapolation of the fitted autocorrelation function to $\tau = 0$. Blank samples containing only NMP and DMSO, respectively, were measured to compare background noise from an undefined particle load and are presented in Figures S88 and S89.
It should be noted that the refractive index contrast in our solvent systems is inherently low. Experimental refractive indices for organic pigments are not comprehensively available and vary with crystalline anisotropy and wavelength. Literature values for the pigments used and related organic semiconductors typically range between $n_{pig} = 1.6 - 1.8$. Taking $n_{pig} \approx 1.7$ as a representative midpoint yields a refractive index contrast $\Delta n^2 = (n_{pig} - n_{solv})^2 \approx 0.05$ for NMP ($n = 1.47$) and DMSO ($n = 1.48$), roughly threefold lower than in aqueous media ($\Delta n^2 \approx 0.14$, $n_{water} = 1.33$). Since scattering intensity scales approximately with $\Delta n^2$, the detection sensitivity for particles is substantially reduced under the experimental conditions. At the same time, DLS remains hypersensitive to any scattering object in the beam path, including undefined foreign particles (dust etc.) that are inevitably present at trace levels. The combination of a diminished sensitivity toward the analyte particles of interest and the undiminished sensitivity toward contaminant scatterers, represents a systematic limitation that should be considered when interpreting the correlograms presented below.

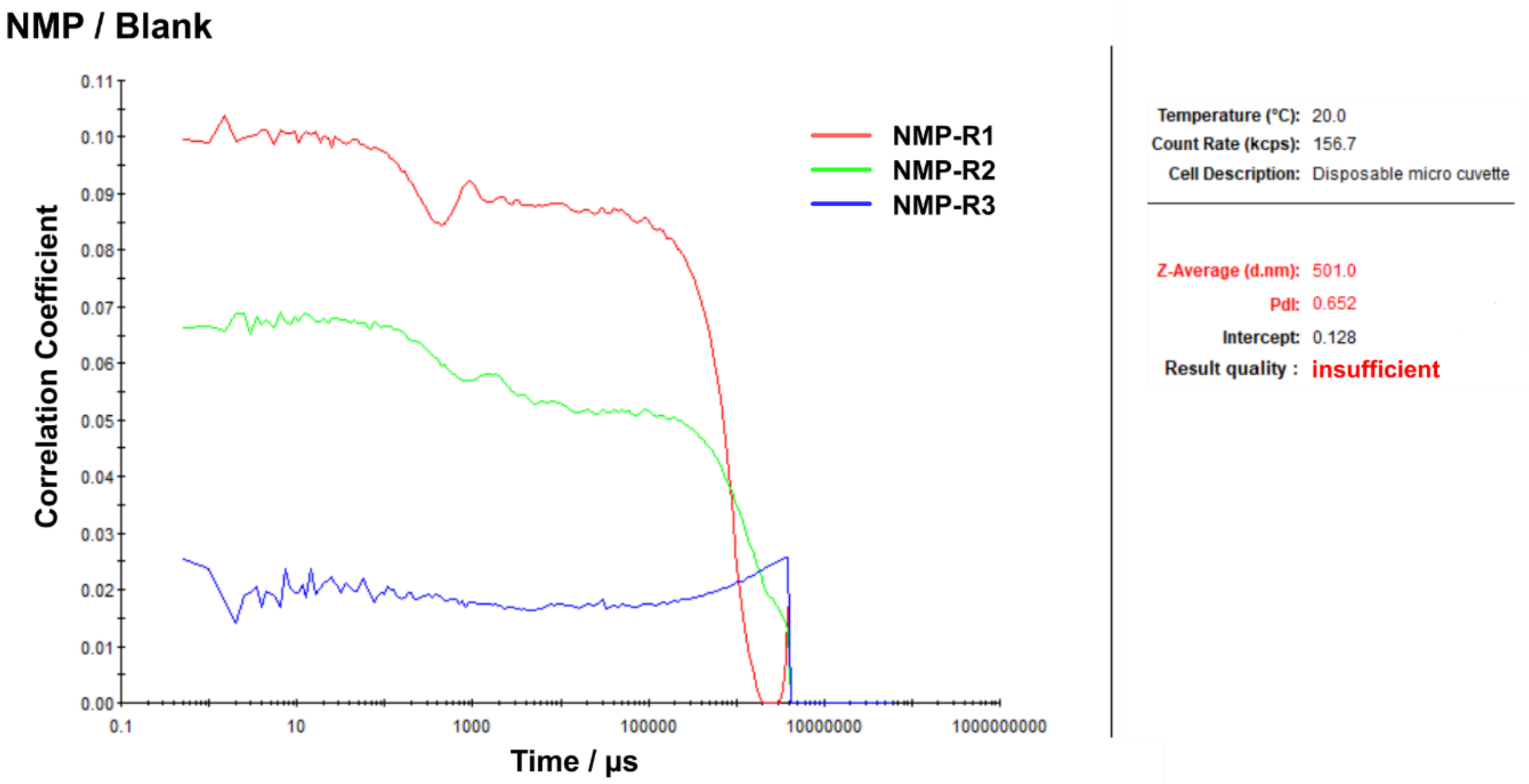


**Figure S89**. Autocorrelation functions from DLS measurements of neat NMP, serving as solvent blank to assess the background scattering contribution to the pigment sample measurements.

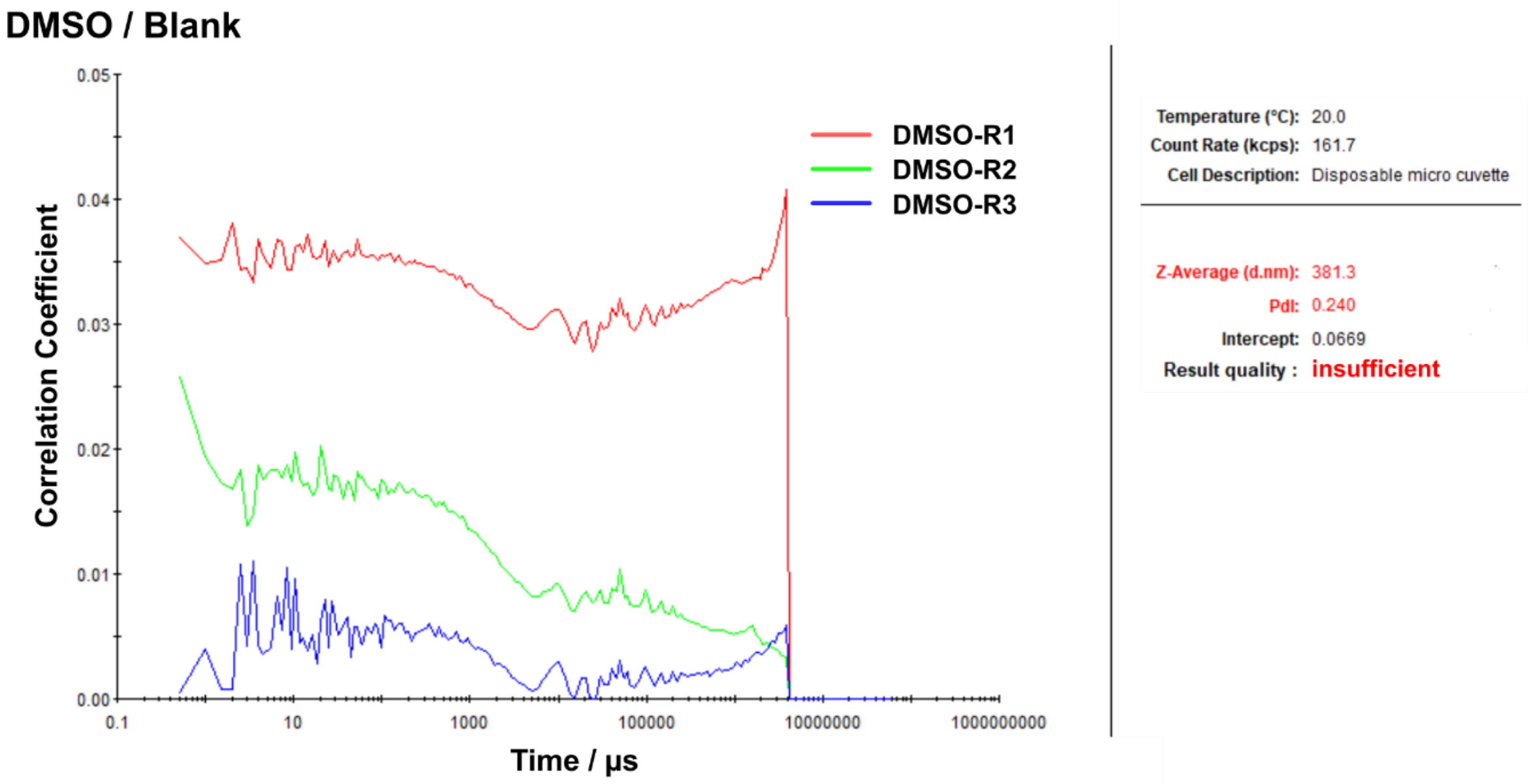


**Figure S90**. Autocorrelation functions from DLS measurements of neat DMSO, serving as solvent blank to assess the background scattering contribution to the pigment sample measurements.

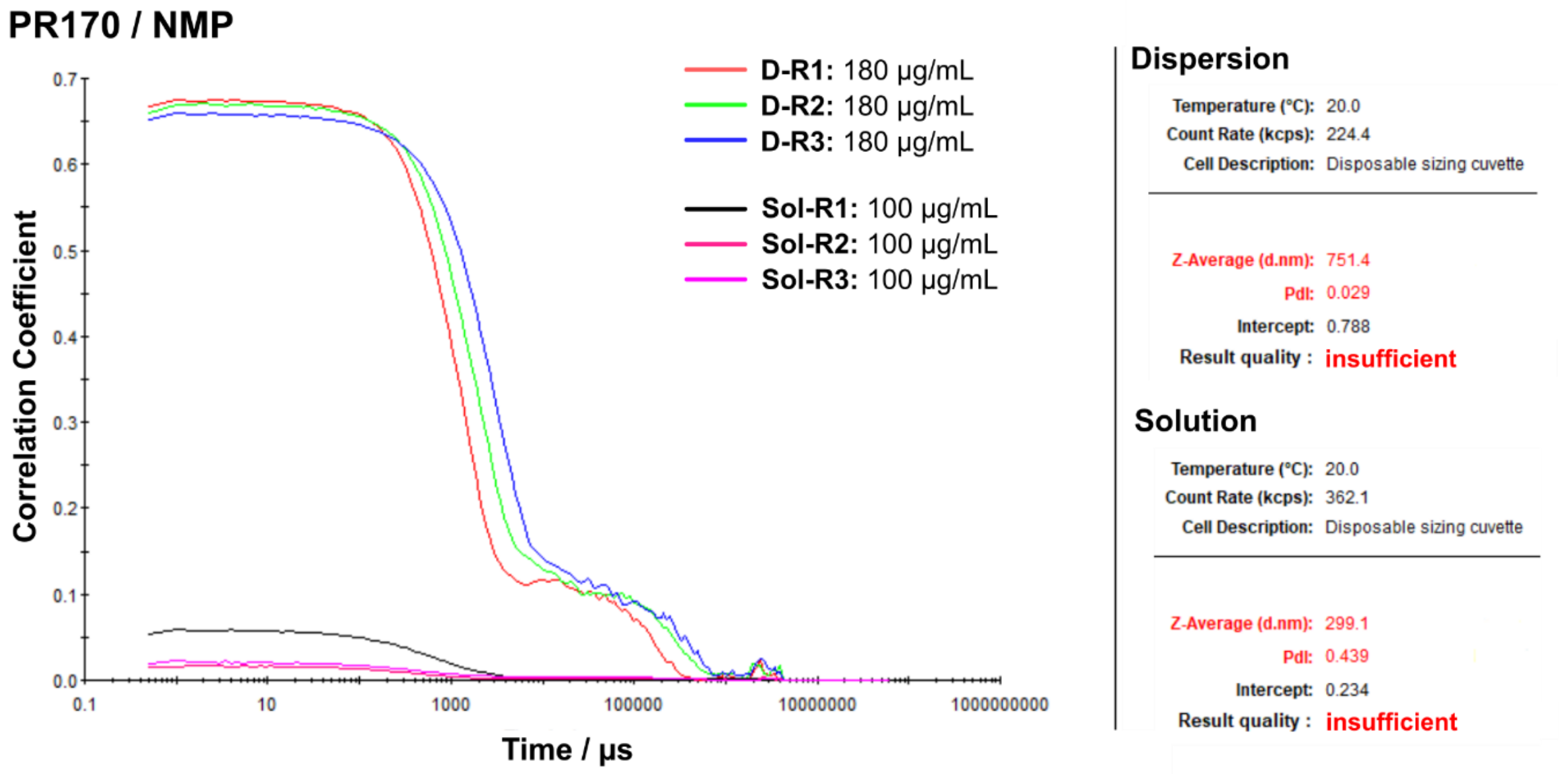


**Figure S91**. Autocorrelation functions from DLS measurements of PR170 in NMP at concentrations 30% above (dispersion, D) and 30% below (solution, Sol) the solubility limit of 140 µg/mL as determined by UV-vis spectroscopy. For all samples, particle size determination was not possible due to inconsistent autocorrelation functions, elevated polydispersity indices, or insufficient scattering intensity.

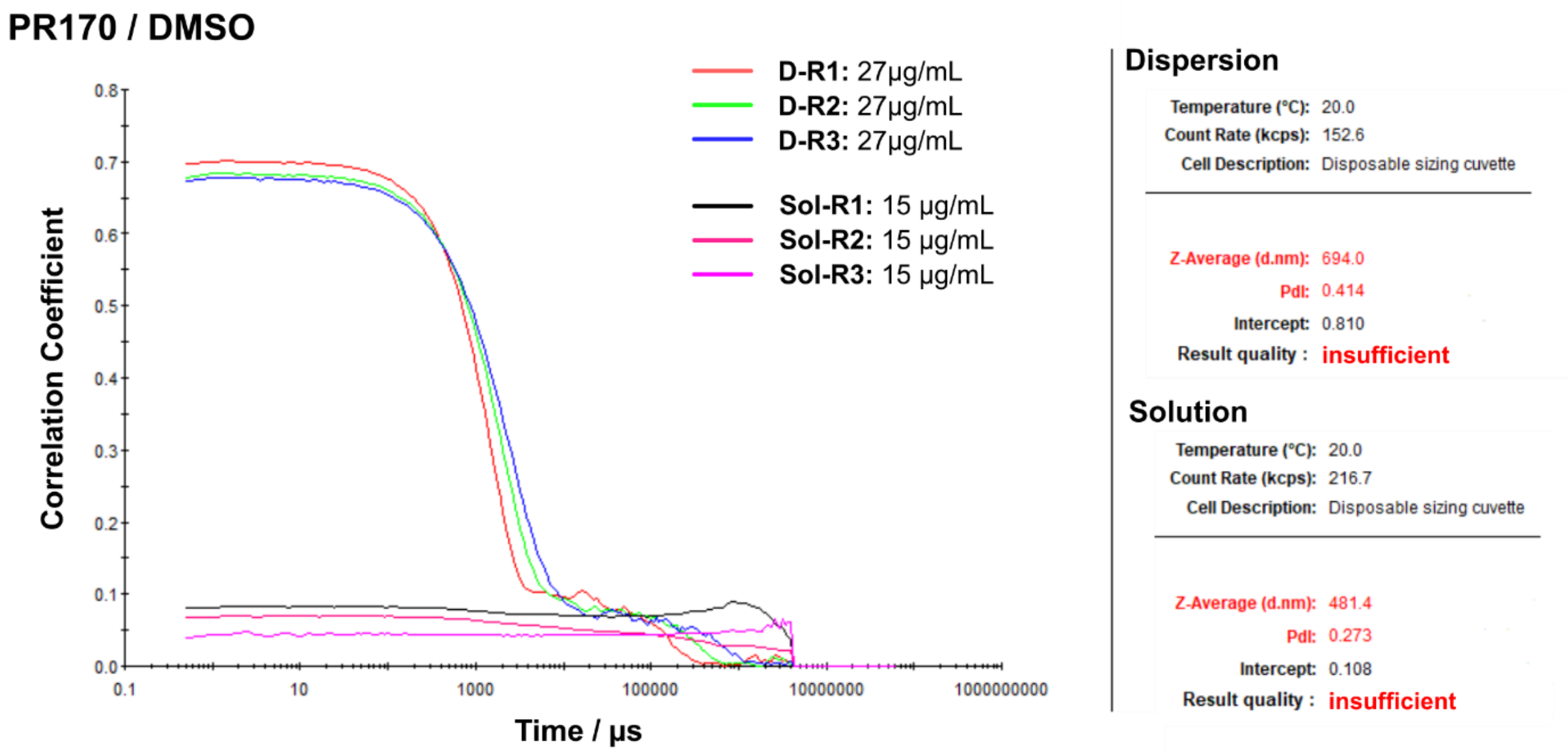


**Figure S92**. Autocorrelation functions from DLS measurements of PR170 in DMSO at concentrations 30% above (dispersion, D) and 30% below (solution, Sol) the solubility limit of 21 µg/mL as determined by UV-vis spectroscopy. Three replicate measurements are shown for each sample. For all samples, particle size determination was not possible due to inconsistent autocorrelation functions, elevated polydispersity indices, or insufficient scattering intensity.

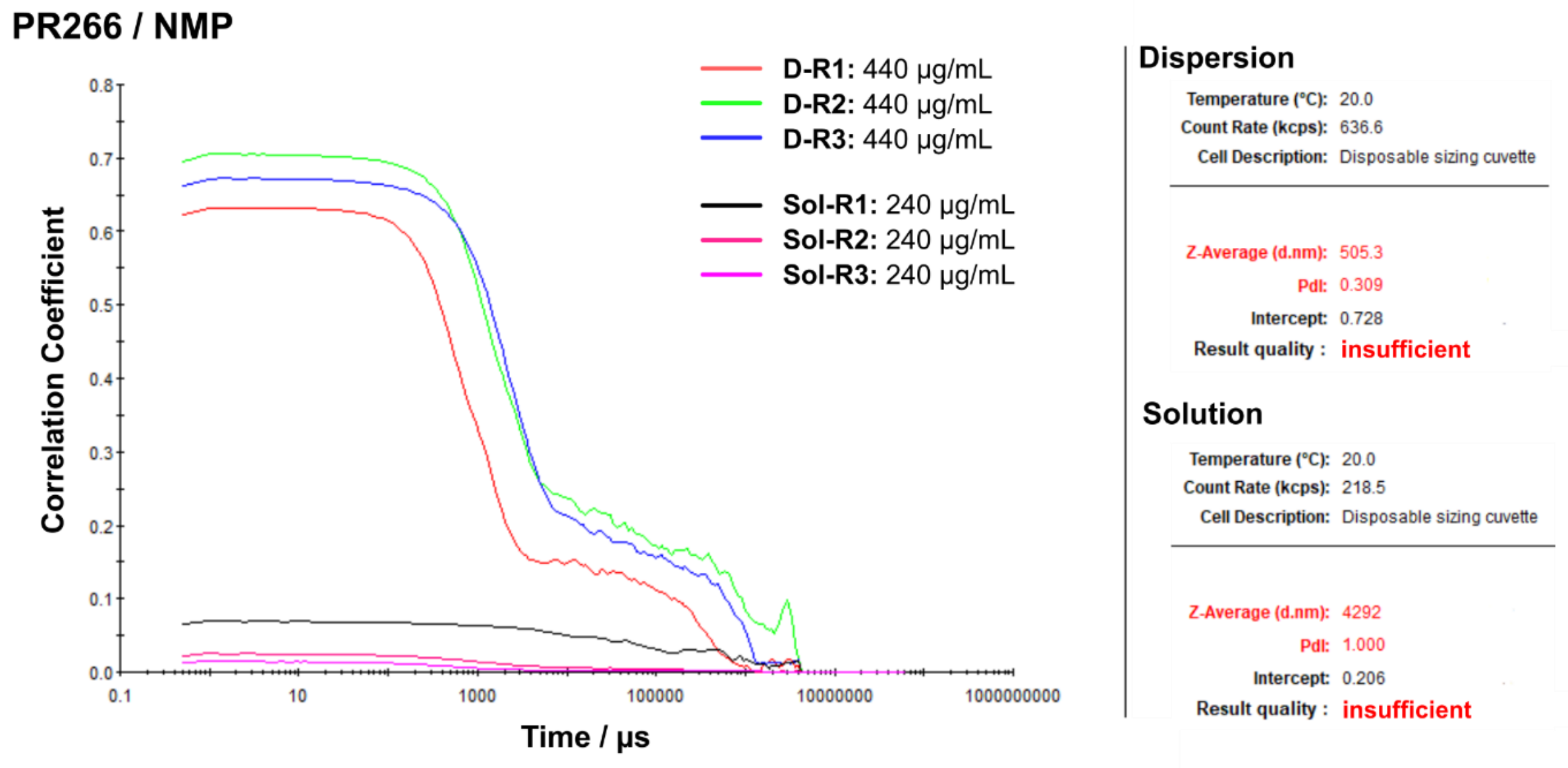


**Figure S93**. Autocorrelation functions from DLS measurements of PR266 in NMP at concentrations 30% above (dispersion, D) and 30% below (solution, Sol) the solubility limit of 340 µg/mL as determined by UV-vis spectroscopy. For all samples, particle size determination was not possible due to inconsistent autocorrelation functions, elevated polydispersity indices, or insufficient scattering intensity.

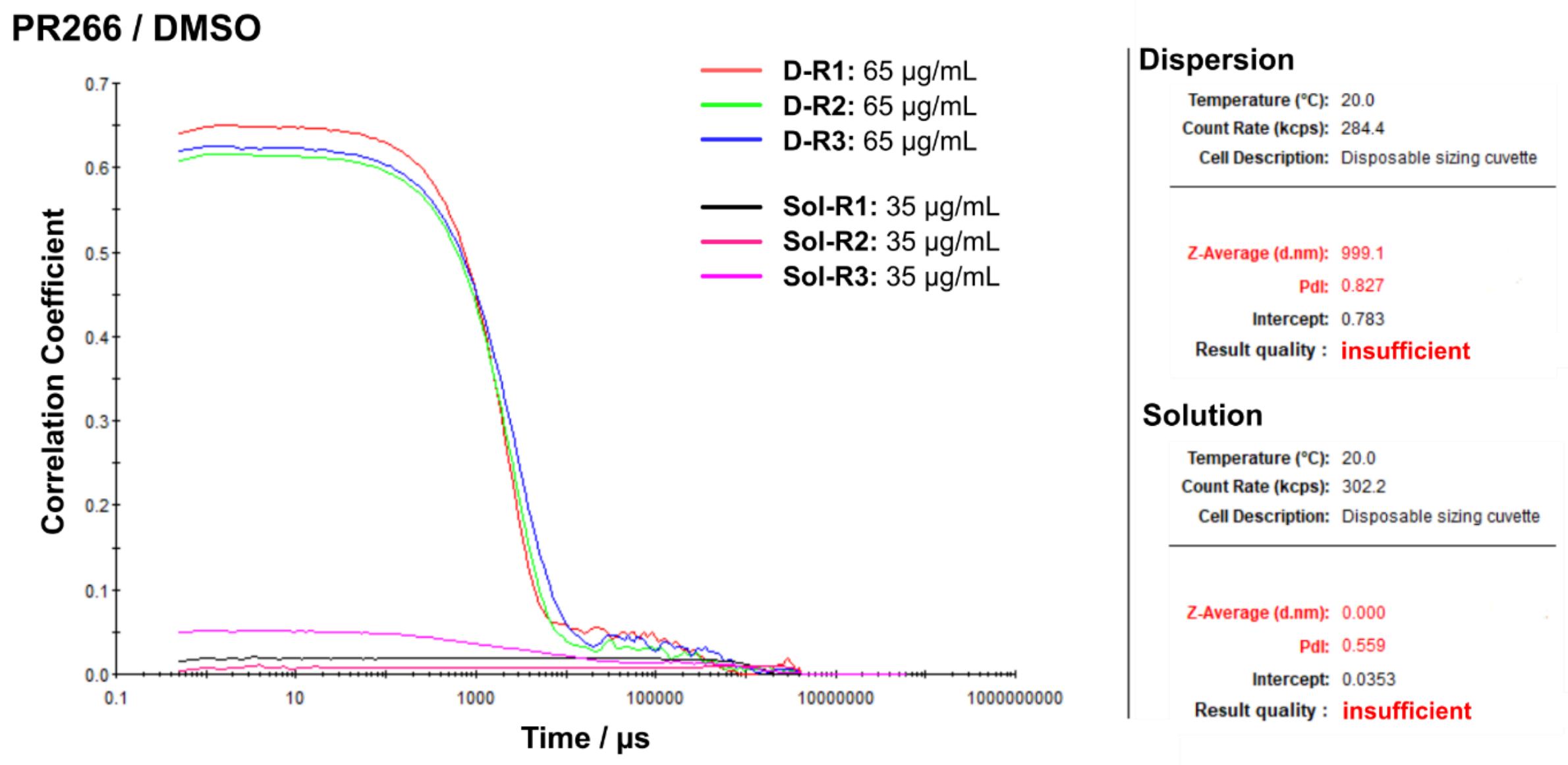


**Figure S94**. Autocorrelation functions from DLS measurements of PR266 in DMSO at concentrations 30% above (dispersion, D) and 30% below (solution, Sol) the solubility limit of 50 µg/mL as determined by UV-vis spectroscopy. For all samples, particle size determination was not possible due to inconsistent autocorrelation functions, elevated polydispersity indices, or insufficient scattering intensity.

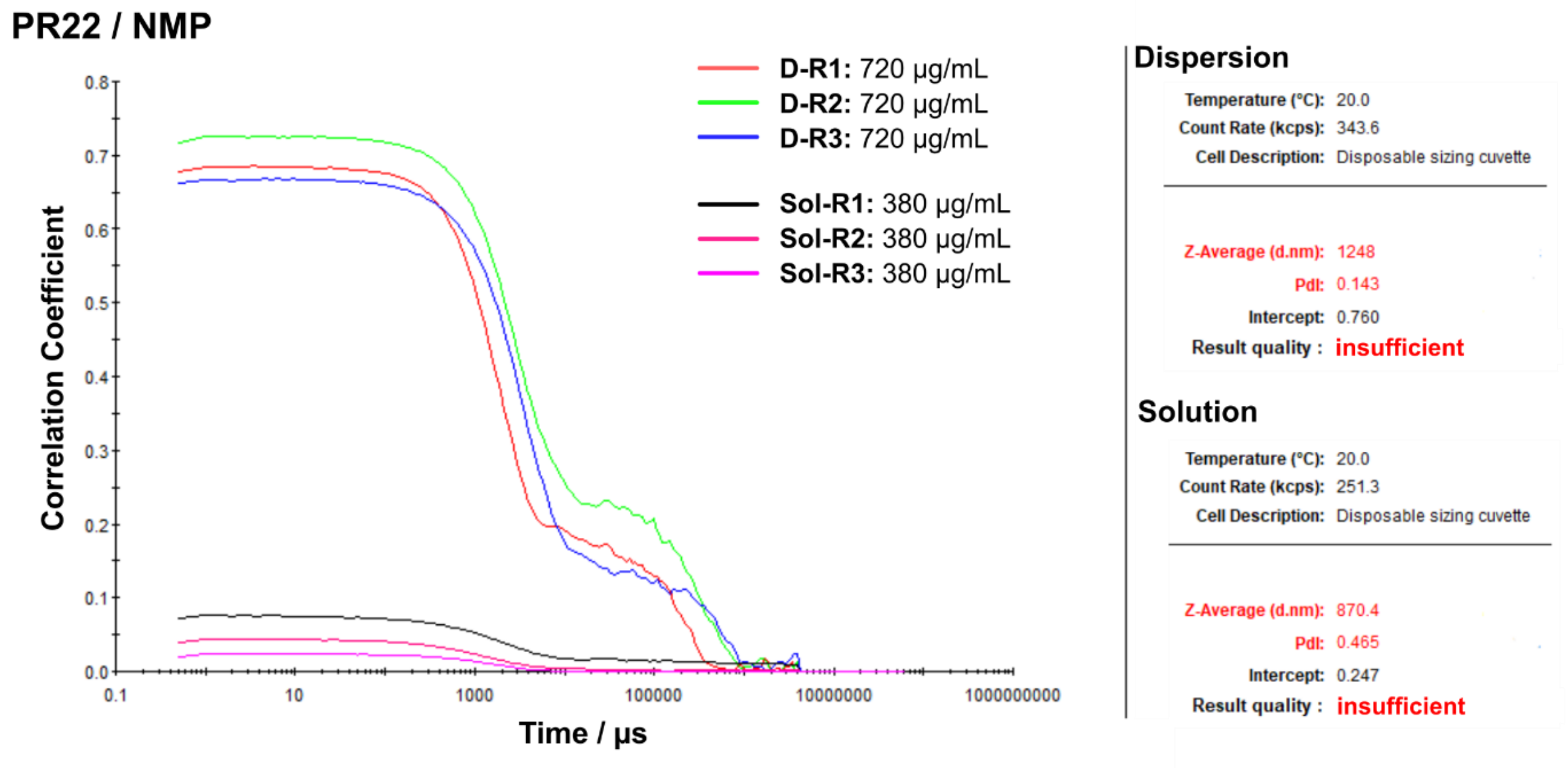


**Figure S95**. Autocorrelation functions from DLS measurements of PR22 in NMP at concentrations 30% above (dispersion, D) and 30% below (solution, Sol) the solubility limit of 550 µg/mL as determined by UV-vis spectroscopy. For all samples, particle size determination was not possible due to inconsistent autocorrelation functions, elevated polydispersity indices, or insufficient scattering intensity.

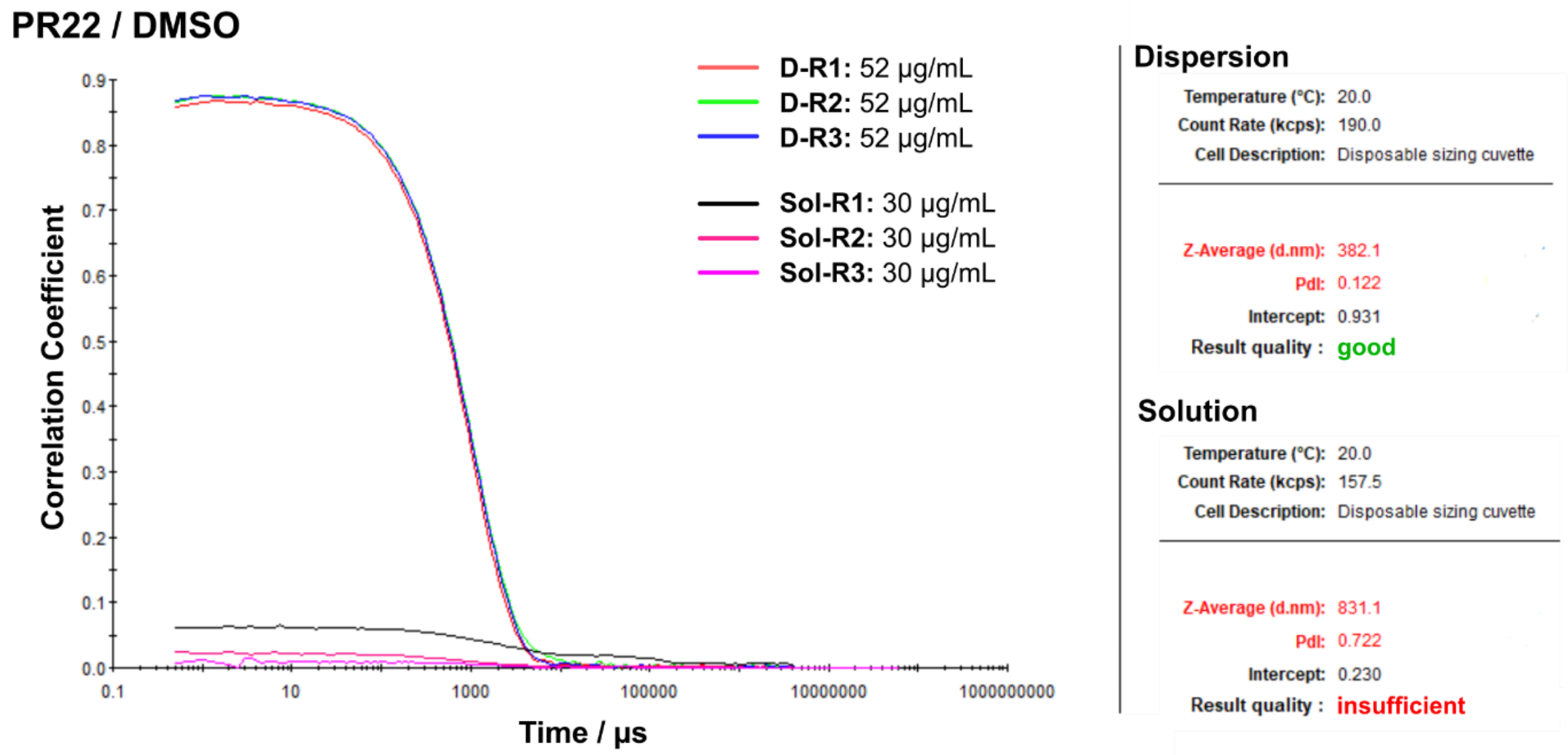


**Figure S96**. Autocorrelation functions from DLS measurements of PR22 in DMSO at concentrations 30% above (dispersion, D) and 30% below (solution, Sol) the solubility limit of 40 µg/mL as determined by UV-vis spectroscopy. For the solution sample, particle size determination was not possible due to inconsistent autocorrelation functions, elevated polydispersity indices, or insufficient scattering intensity. For the dispersion sample, the data quality was sufficient giving an average particle size of 382.1 nm with a PdI of 0.122.

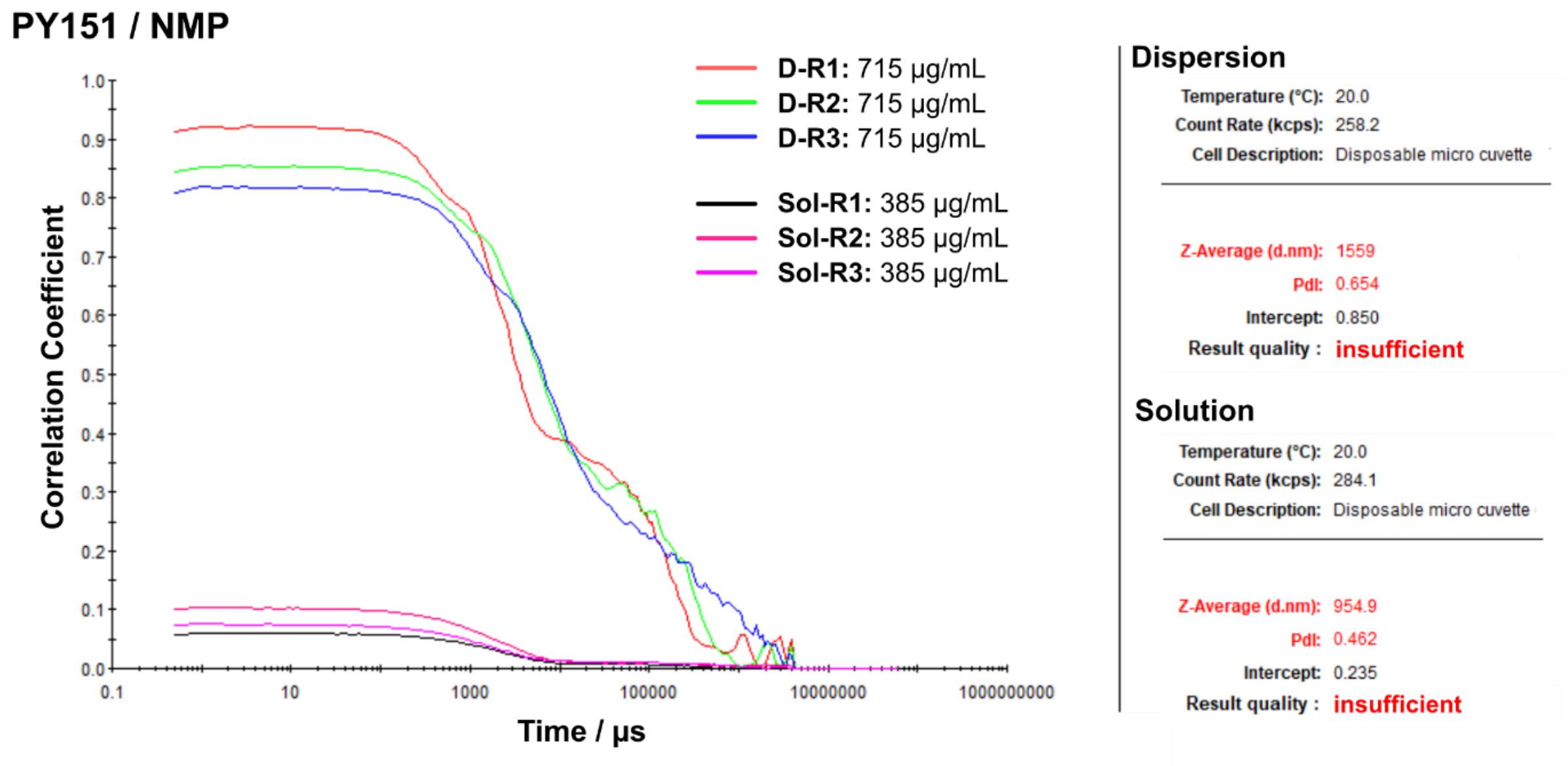


**Figure S97**. Autocorrelation functions from DLS measurements of PY151 in NMP at concentrations 30% above (dispersion, D) and 30% below (solution, Sol) the solubility limit of 550 µg/mL as determined by UV-vis spectroscopy. For all samples, particle size determination was not possible due to inconsistent autocorrelation functions, elevated polydispersity indices, or insufficient scattering intensity.

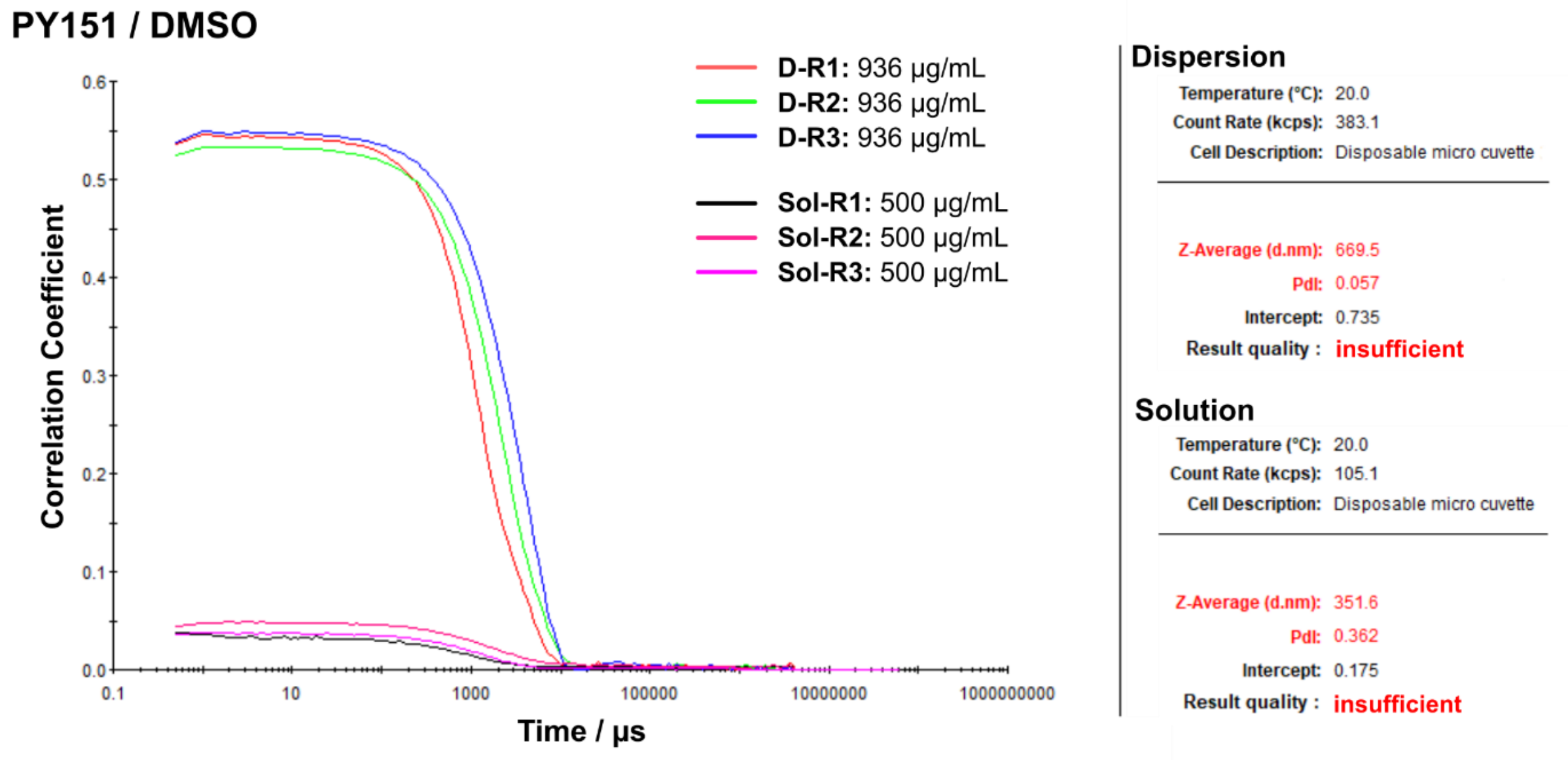


**Figure S98**. Autocorrelation functions from DLS measurements of PY151 in DMSO at concentrations 30% above (dispersion, D) and 30% below (solution, Sol) the solubility limit of 720 µg/mL as determined by UV-vis spectroscopy. For all samples, particle size determination was not possible due to inconsistent autocorrelation functions, elevated polydispersity indices, or insufficient scattering intensity.

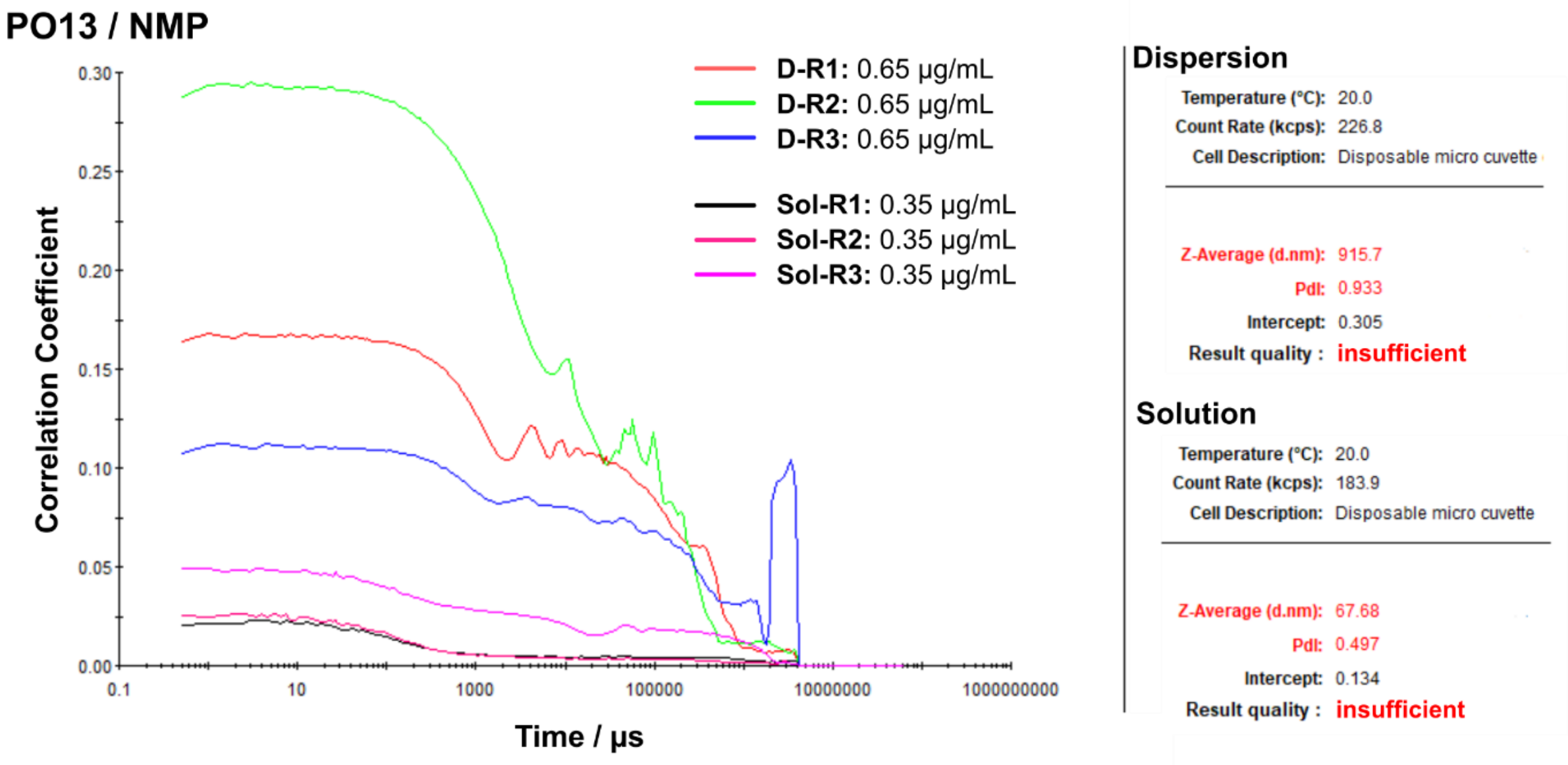


**Figure S99**. Autocorrelation functions from DLS measurements of PO13 in NMP at concentrations 30% above (dispersion, D) and 30% below (solution, Sol) the solubility limit of 0.5 µg/mL as determined by UV-vis spectroscopy. For all samples, particle size determination was not possible due to inconsistent autocorrelation functions, elevated polydispersity indices, or insufficient scattering intensity.

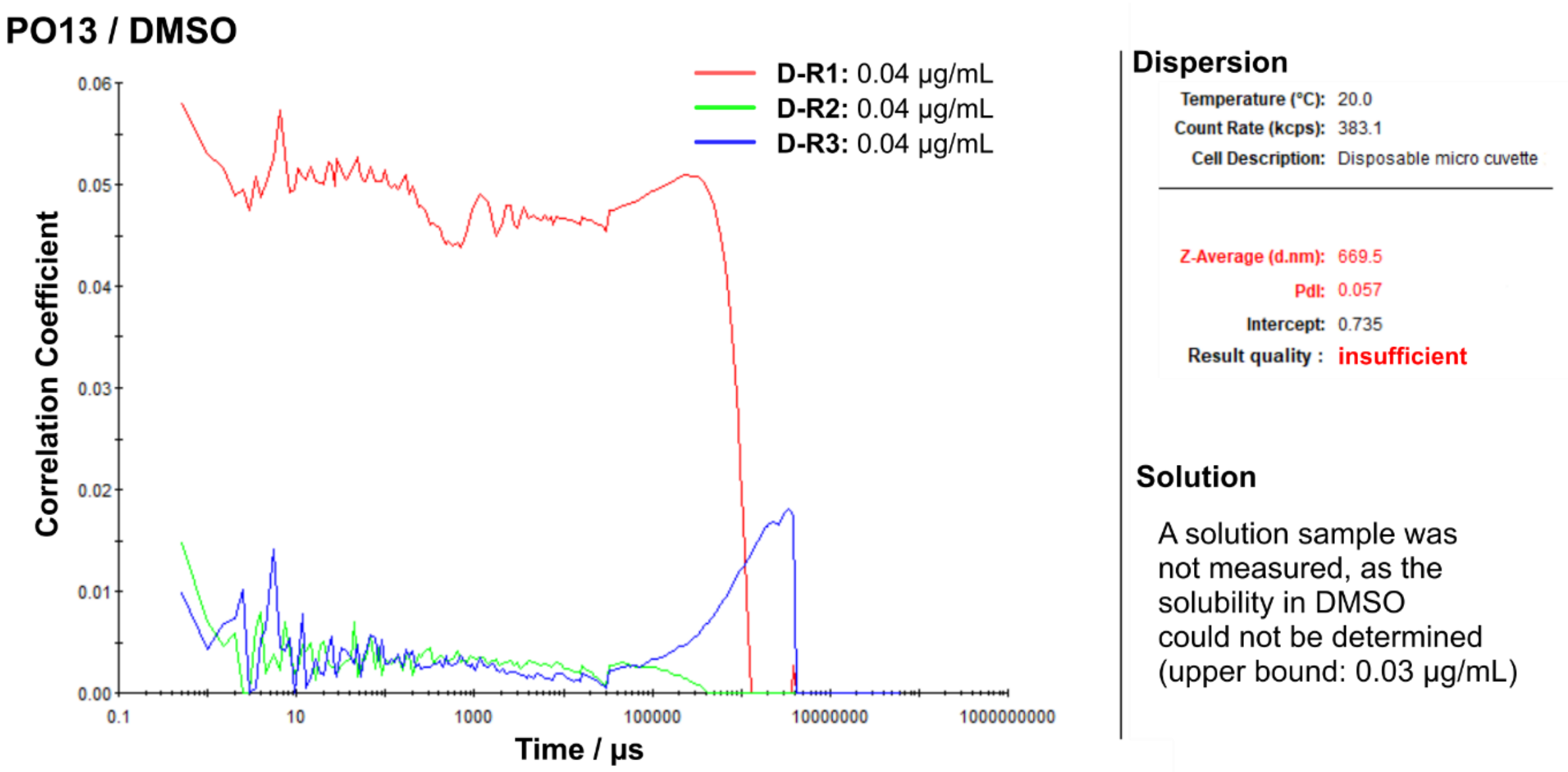


**Figure S100**. Autocorrelation functions from DLS measurements of PO13 in DMSO at concentrations 30% above the upper bound solubility limit of 0.03 µg/mL as determined by UV-vis spectroscopy. For all samples, particle size determination was not possible due to inconsistent autocorrelation functions, elevated polydispersity indices, or insufficient scattering intensity.

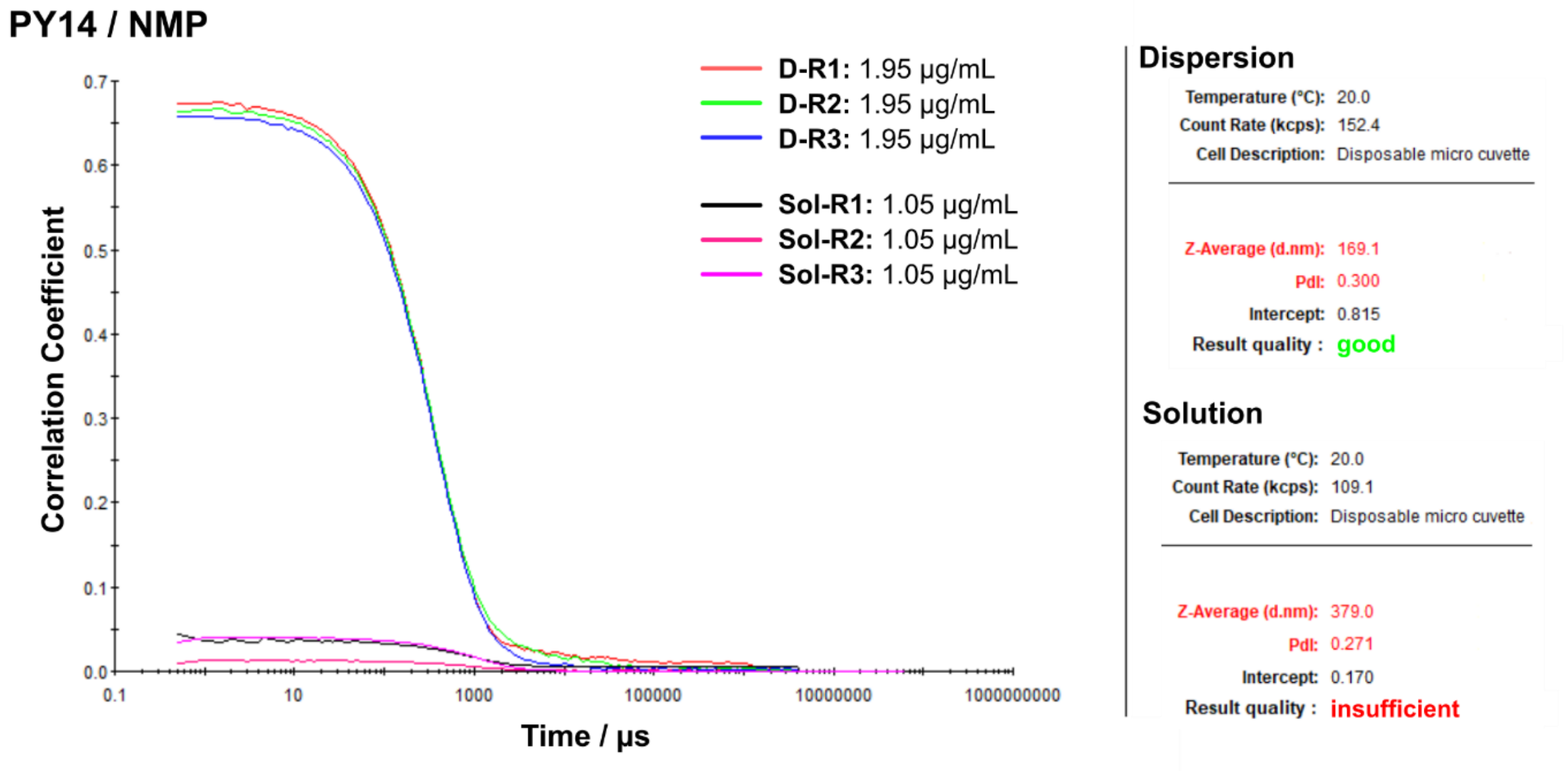


**Figure S101**. Autocorrelation functions from DLS measurements of PY14 in NMP at concentrations 30% above (dispersion, D) and 30% below (solution, Sol) the solubility limit of 1.5 µg/mL as determined by UV-vis spectroscopy. For the solution sample, particle size determination was not possible due to inconsistent autocorrelation functions, elevated polydispersity indices, or insufficient scattering intensity. For the dispersion sample, the data quality was sufficient giving an average particle size of 169.1 nm with a PdI of 0.300.

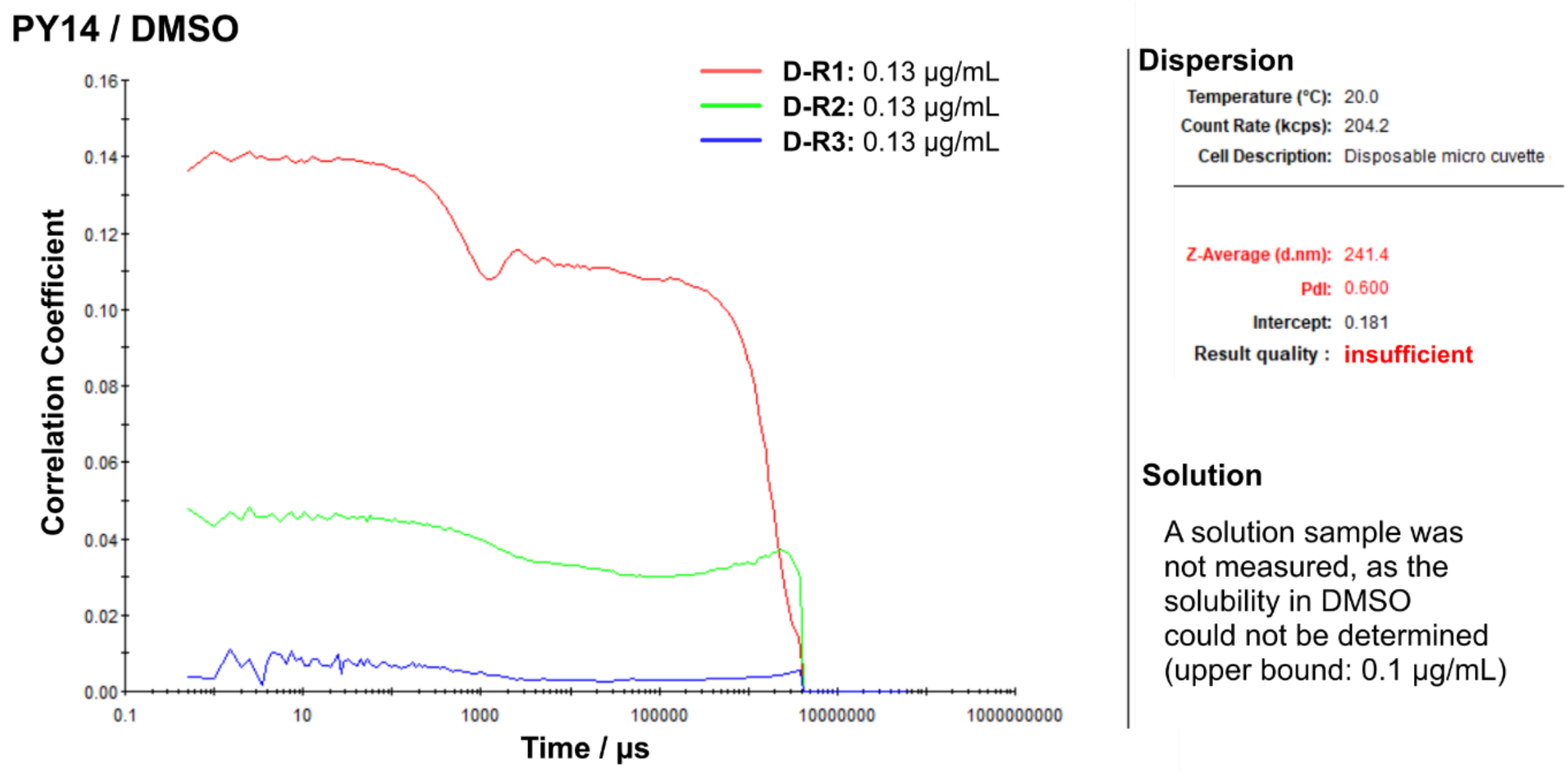


**Figure S102**. Autocorrelation functions from DLS measurements of PY14 in DMSO at concentrations 30% above the upper bound solubility limit of 0.1 µg/mL as determined by UV-vis spectroscopy. For all samples, particle size determination was not possible due to inconsistent autocorrelation functions, elevated polydispersity indices, or insufficient scattering intensity.

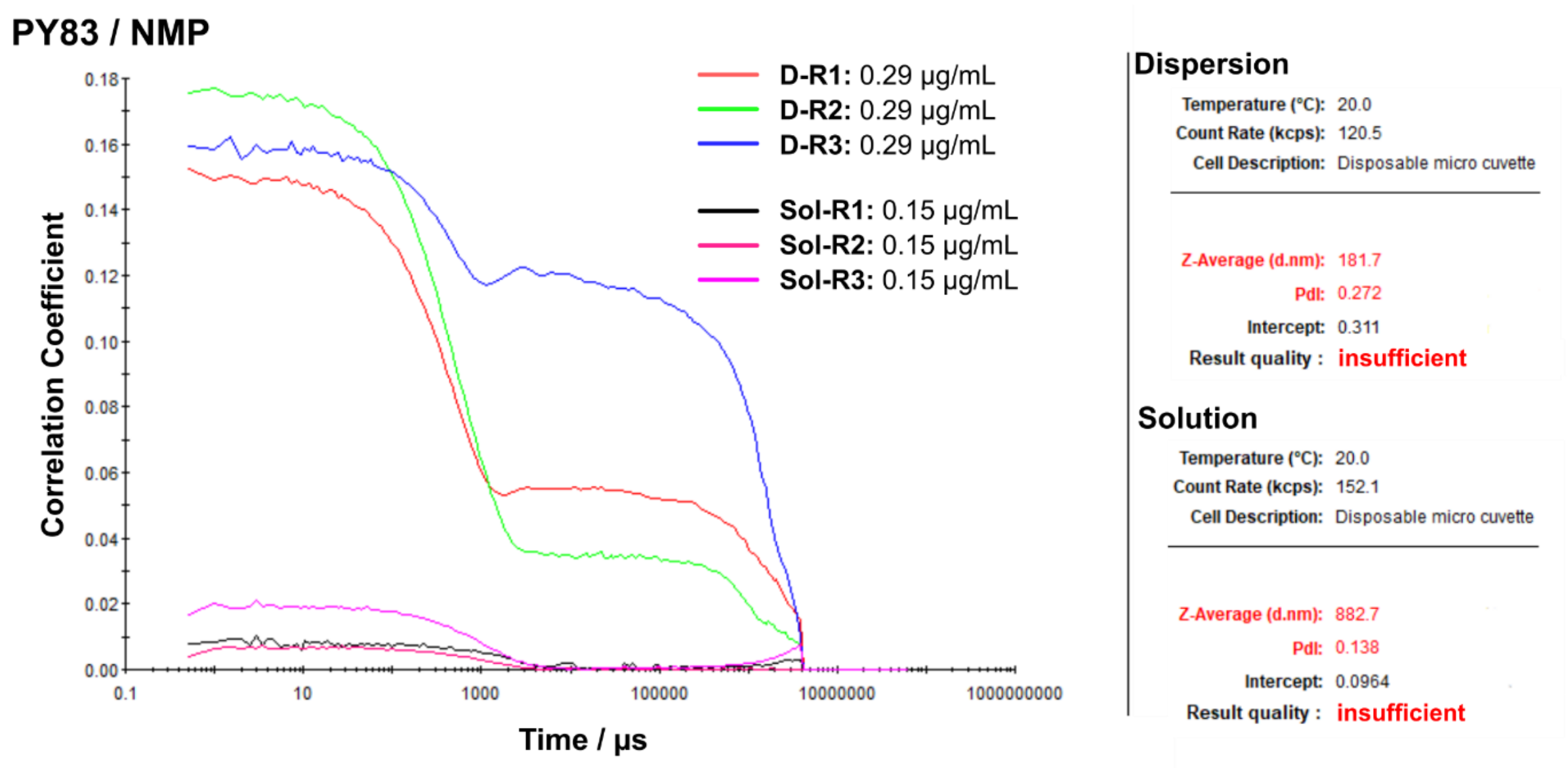


**Figure S103**. Autocorrelation functions from DLS measurements of PY83 in NMP at concentrations 30% above (dispersion, D) and 30% below (solution, Sol) the solubility limit of 0.22 µg/mL as determined by UV-vis spectroscopy. For all samples, particle size determination was not possible due to inconsistent autocorrelation functions, elevated polydispersity indices, or insufficient scattering intensity.

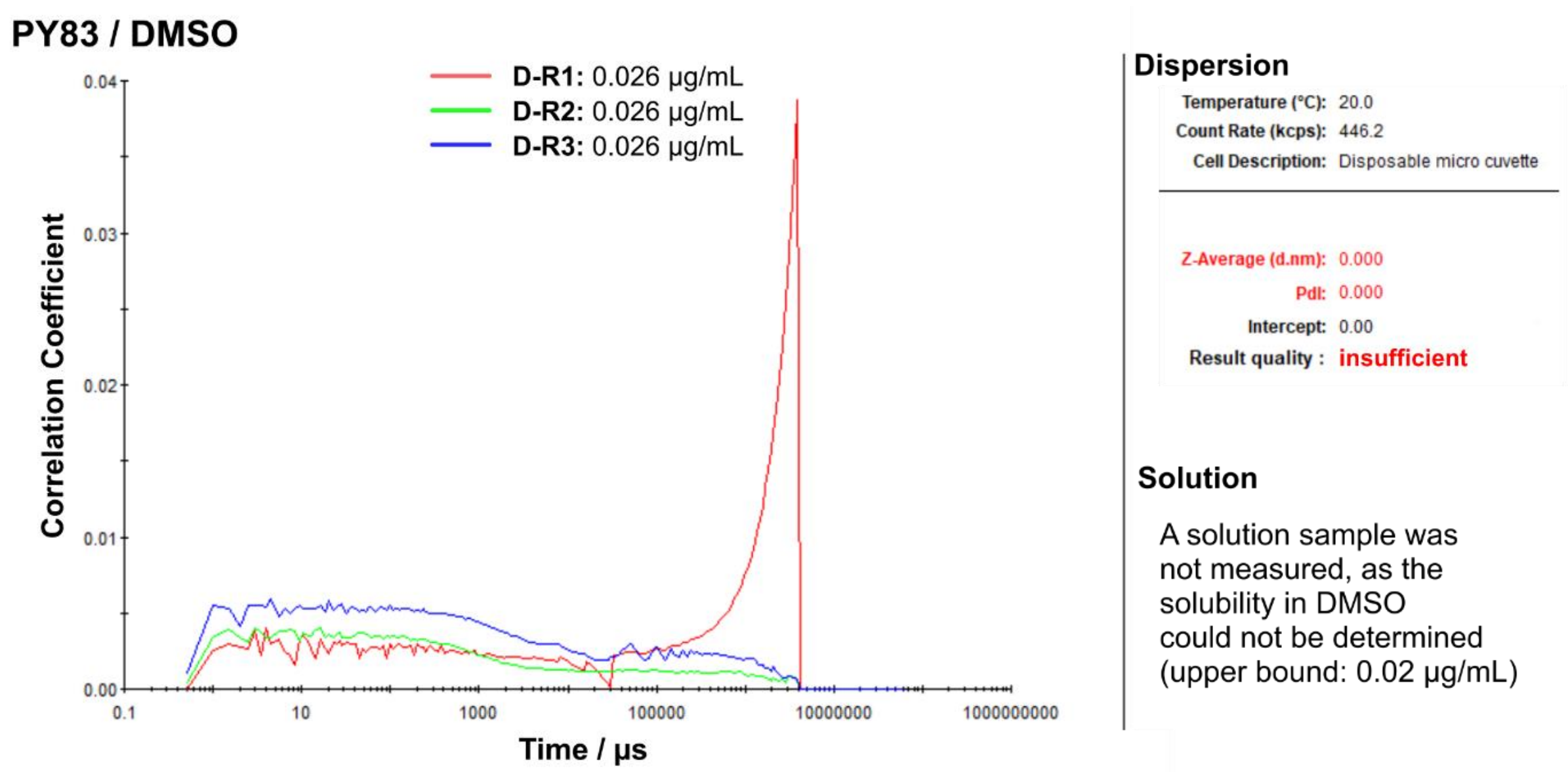


**Figure S104**. Autocorrelation functions from DLS measurements of PY83 in DMSO at concentrations 30% above the upper bound solubility limit of 0.02 µg/mL as determined by UV-vis spectroscopy. For all samples, particle size determination was not possible due to inconsistent autocorrelation functions, elevated polydispersity indices, or insufficient scattering intensity.

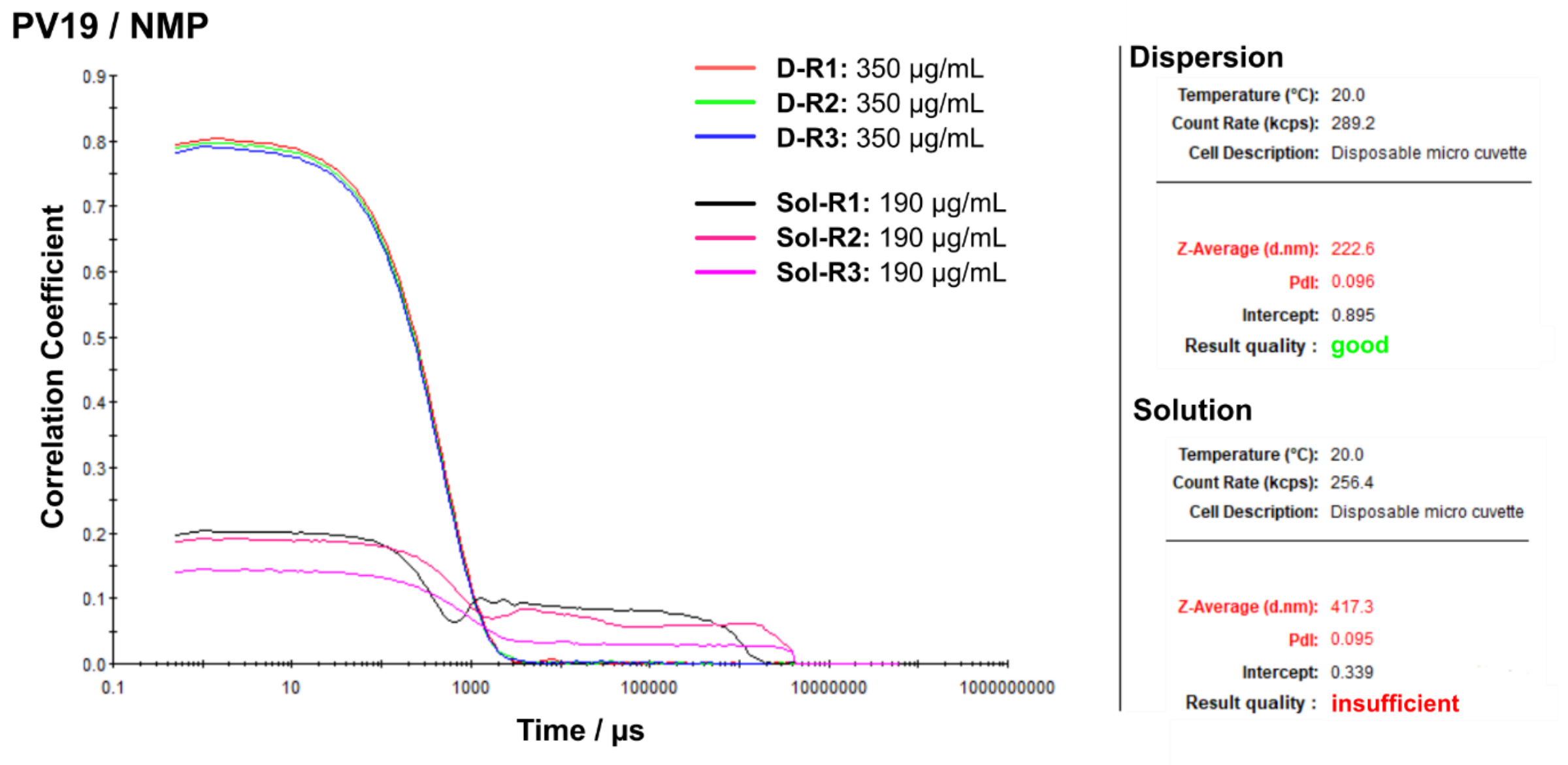


**Figure S105**. Autocorrelation functions from DLS measurements of PV19 in NMP at concentrations 30% above (dispersion, D) and 30% below (solution, Sol) the solubility limit of 270 µg/mL as determined by UV-vis spectroscopy. For the solution sample, particle size determination was not possible due to inconsistent autocorrelation functions, elevated polydispersity indices, or insufficient scattering intensity. For the dispersion sample, the data quality was sufficient giving an average particle size of 222.6 nm with a PdI of 0.096.

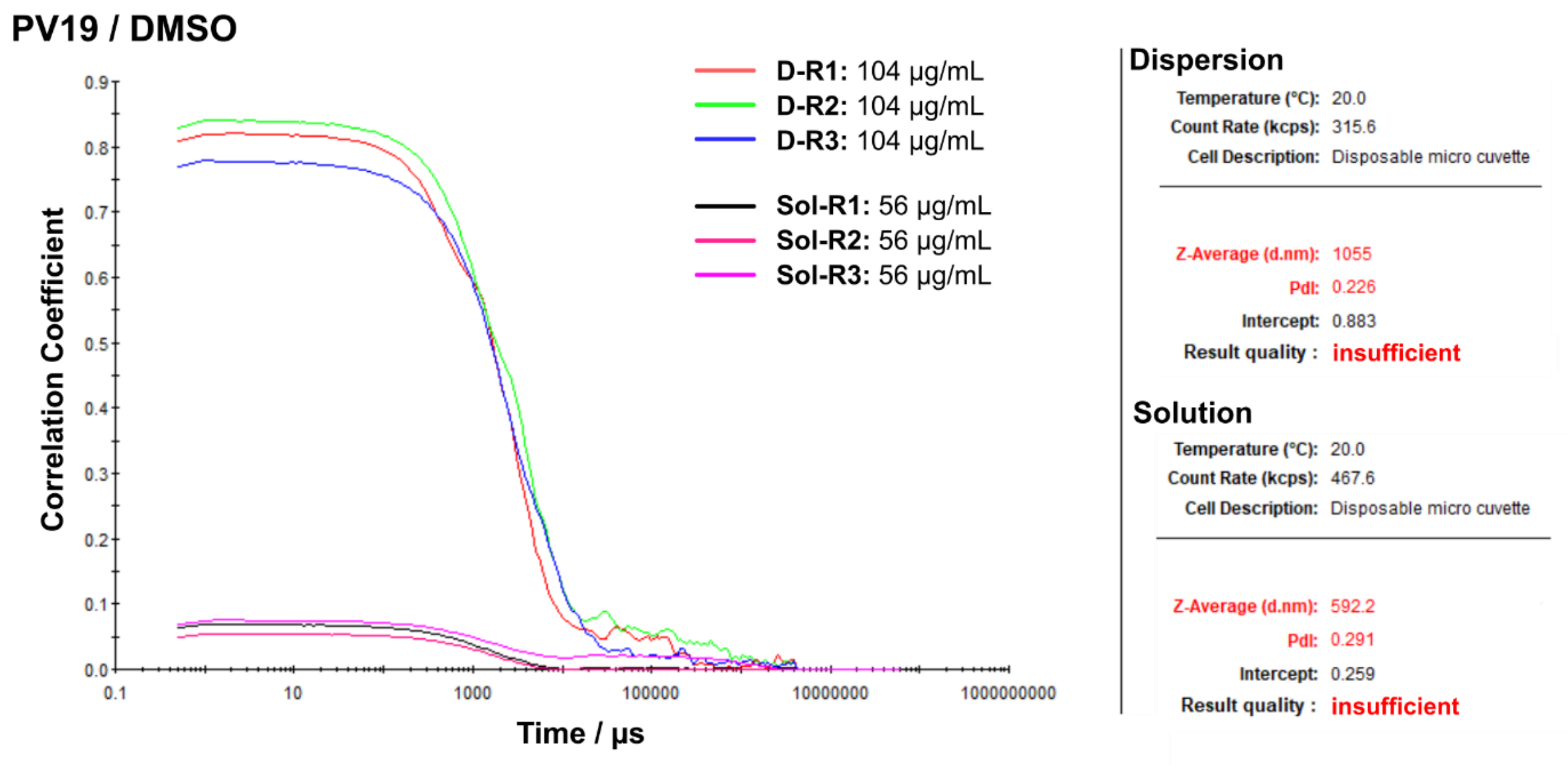


**Figure S106**. Autocorrelation functions from DLS measurements of PV19 in DMSO at concentrations 30% above (dispersion, D) and 30% below (solution, Sol) the solubility limit of 80 µg/mL as determined by UV-vis spectroscopy. For all samples, particle size determination was not possible due to inconsistent autocorrelation functions, elevated polydispersity indices, or insufficient scattering intensity.

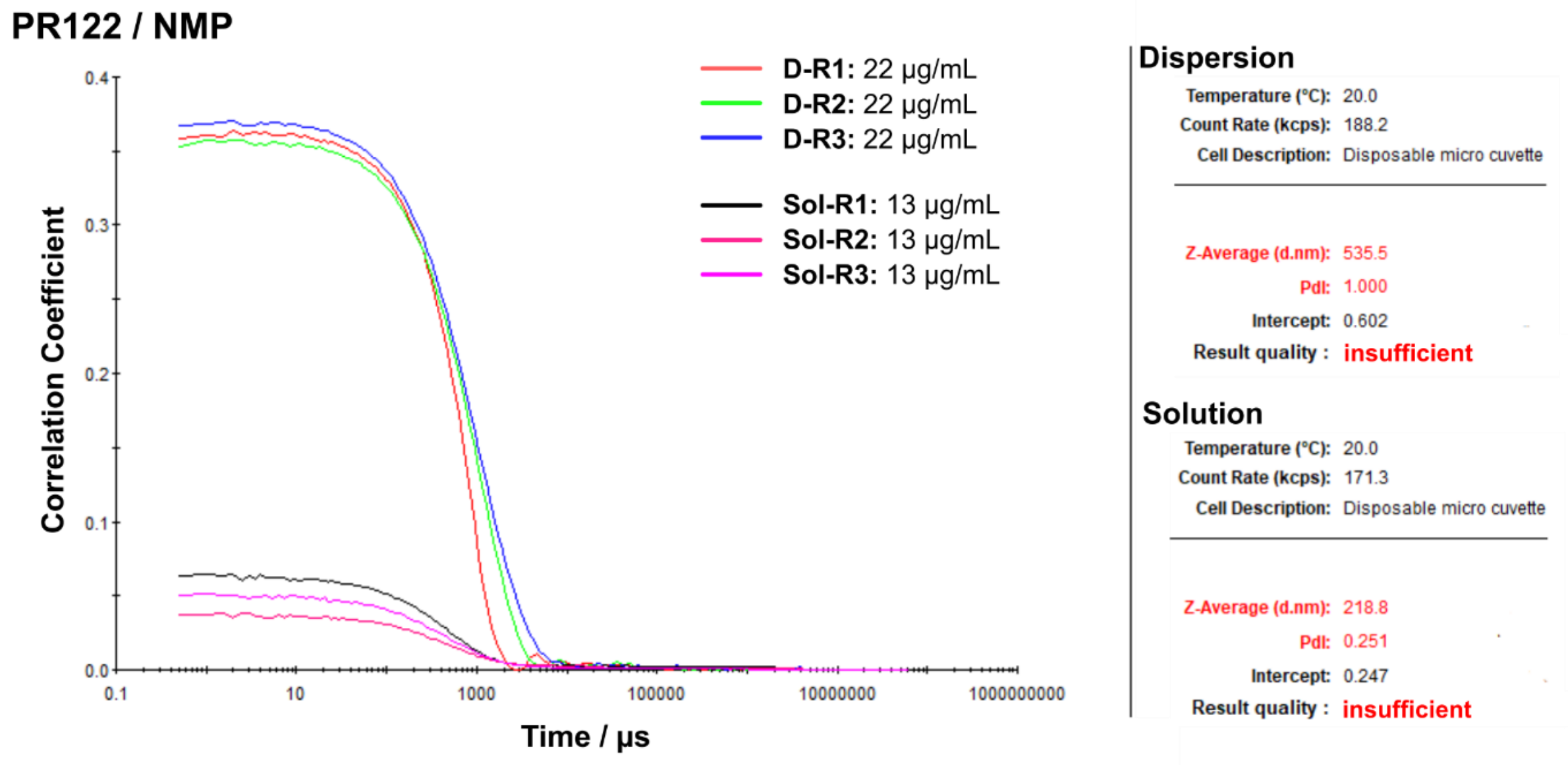


**Figure S107**. Autocorrelation functions from DLS measurements of PR122 in NMP at concentrations 30% above (dispersion, D) and 30% below (solution, Sol) the solubility limit of 18 µg/mL as determined by UV-vis spectroscopy. For all samples, particle size determination was not possible due to inconsistent autocorrelation functions, elevated polydispersity indices, or insufficient scattering intensity.

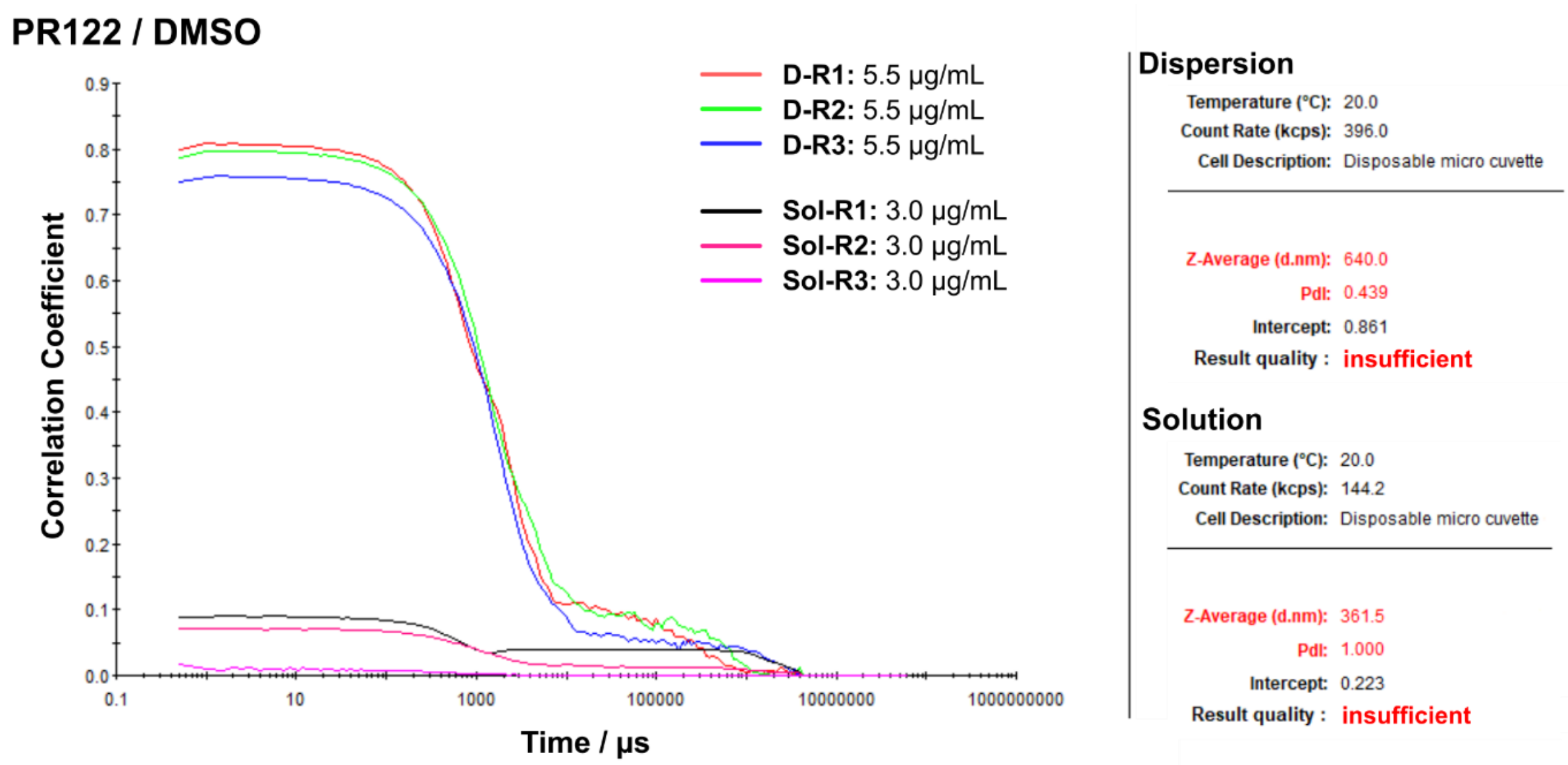


**Figure S108**. Autocorrelation functions from DLS measurements of PR122 in DMSO at concentrations 30% above (dispersion, D) and 30% below (solution, Sol) the solubility limit of 4.2 µg/mL as determined by UV-vis spectroscopy. For all samples, particle size determination was not possible due to inconsistent autocorrelation functions, elevated polydispersity indices, or insufficient scattering intensity.

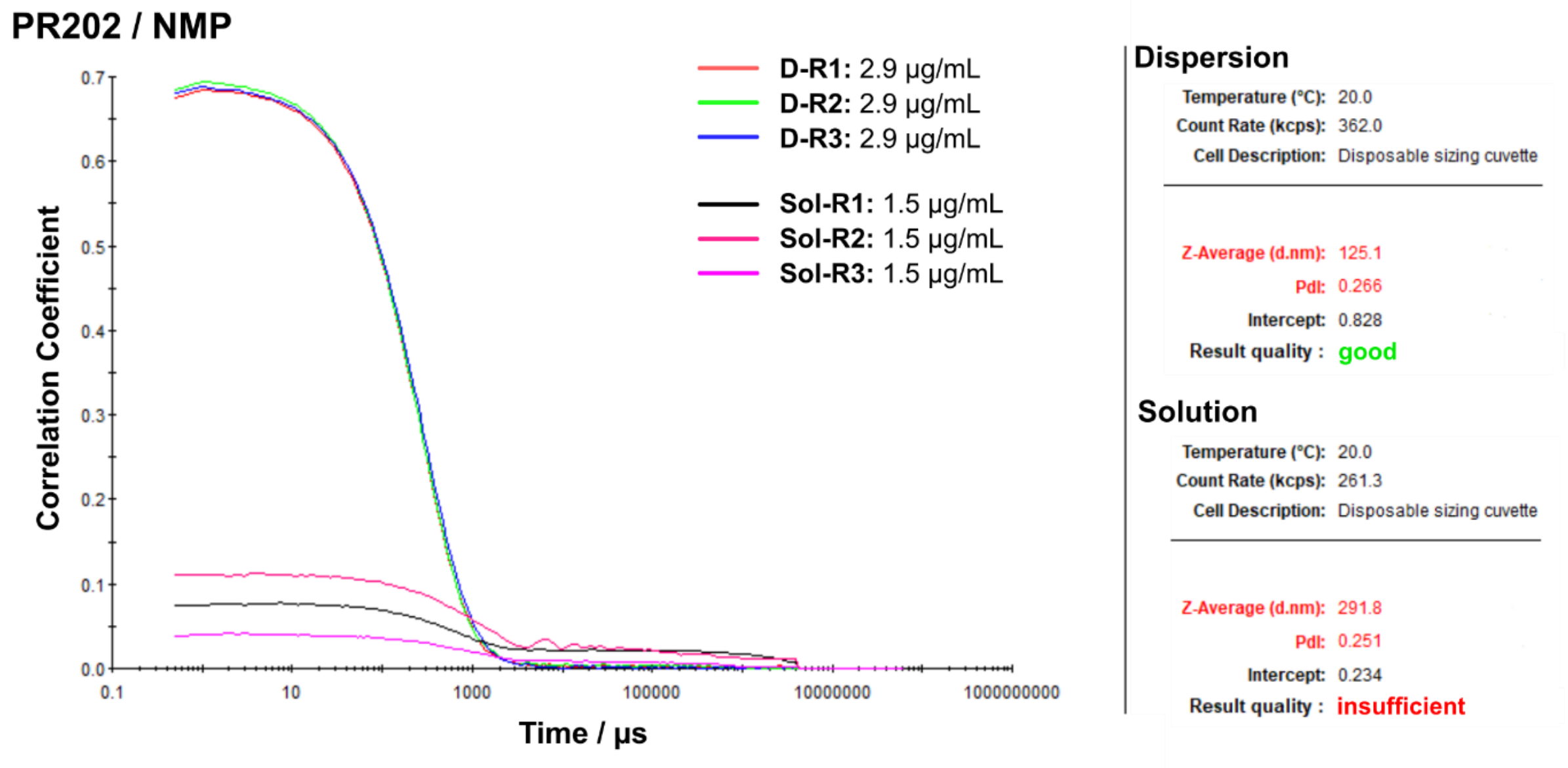


**Figure S109**. Autocorrelation functions from DLS measurements of PR202 in NMP at concentrations 30% above (dispersion, D) and 30% below (solution, Sol) the solubility limit of 2.2 µg/mL as determined by UV-vis spectroscopy. For the solution sample, particle size determination was not possible due to inconsistent autocorrelation functions, elevated polydispersity indices, or insufficient scattering intensity. For the dispersion sample, the data quality was sufficient giving an average particle size of 125.1 nm with a PdI of 0.266.

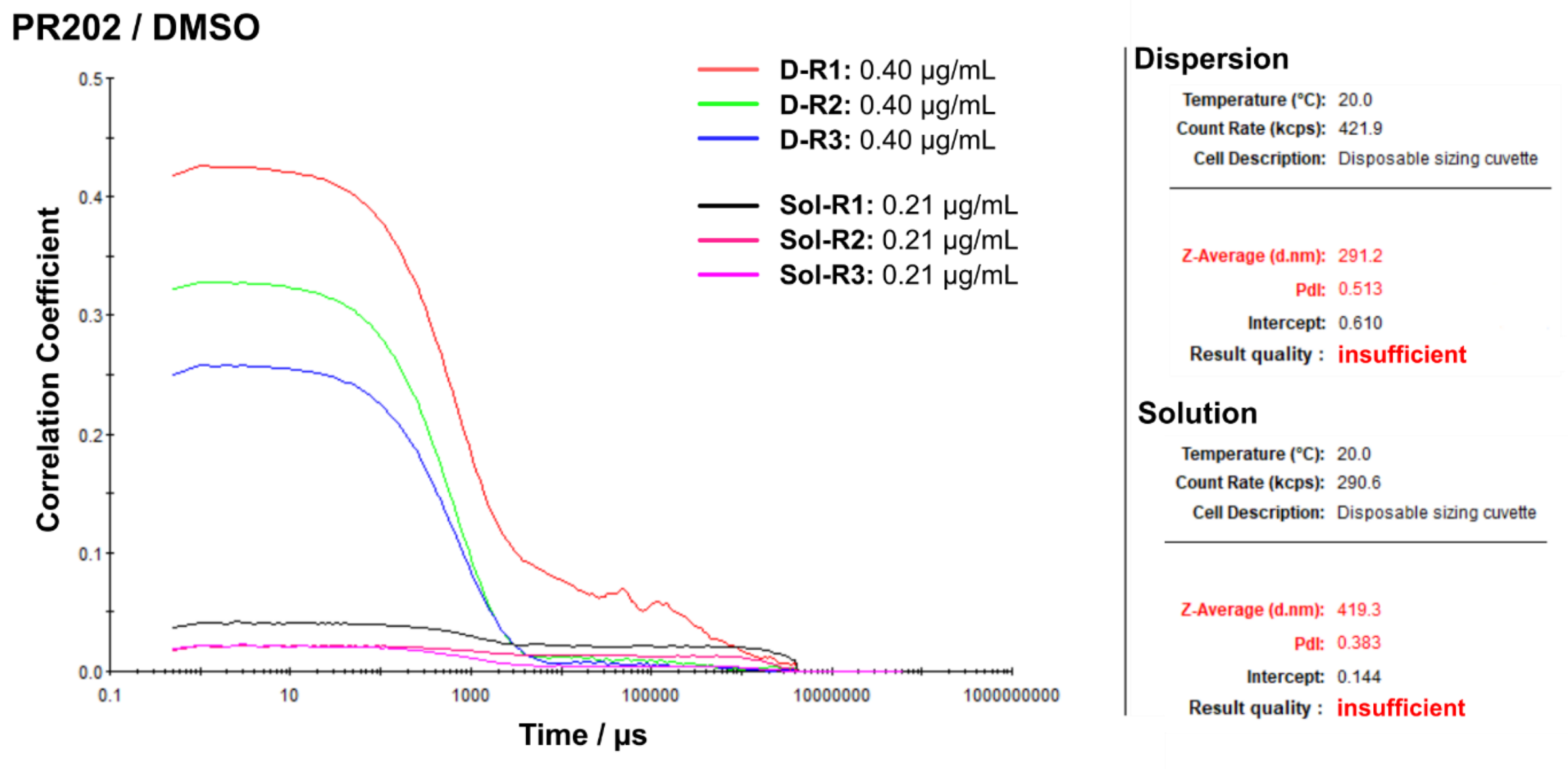


**Figure S110**. Autocorrelation functions from DLS measurements of PR202 in DMSO at concentrations 30% above (dispersion, D) and 30% below (solution, Sol) the solubility limit of 0.3 µg/mL as determined by UV-vis spectroscopy. For all samples, particle size determination was not possible due to inconsistent autocorrelation functions, elevated polydispersity indices, or insufficient scattering intensity.

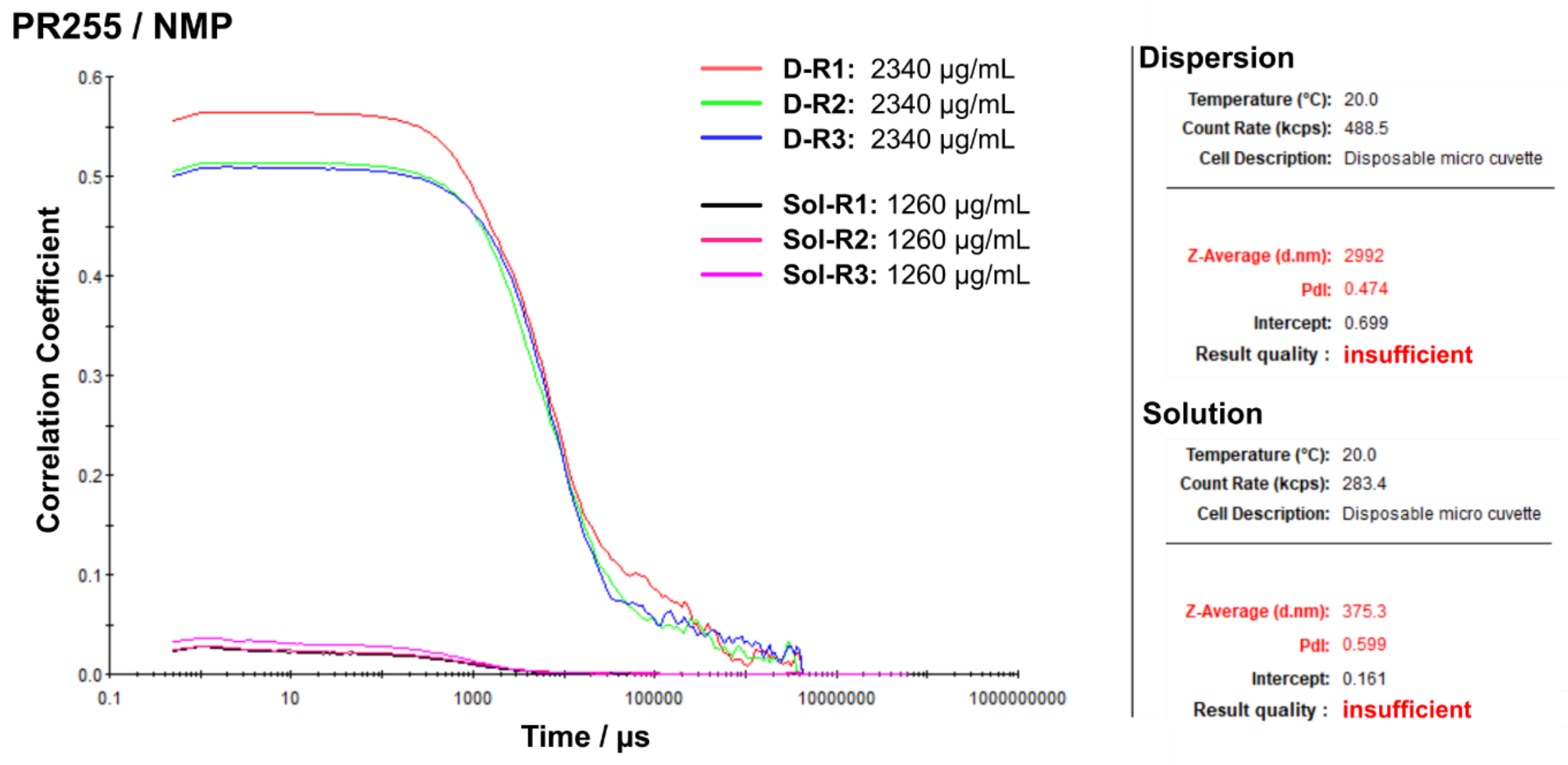


**Figure S111**. Autocorrelation functions from DLS measurements of PR255 in NMP at concentrations 30% above (dispersion, D) and 30% below (solution, Sol) the solubility limit of 1800 µg/mL as determined by UV-vis spectroscopy. For all samples, particle size determination was not possible due to inconsistent autocorrelation functions, elevated polydispersity indices, or insufficient scattering intensity.

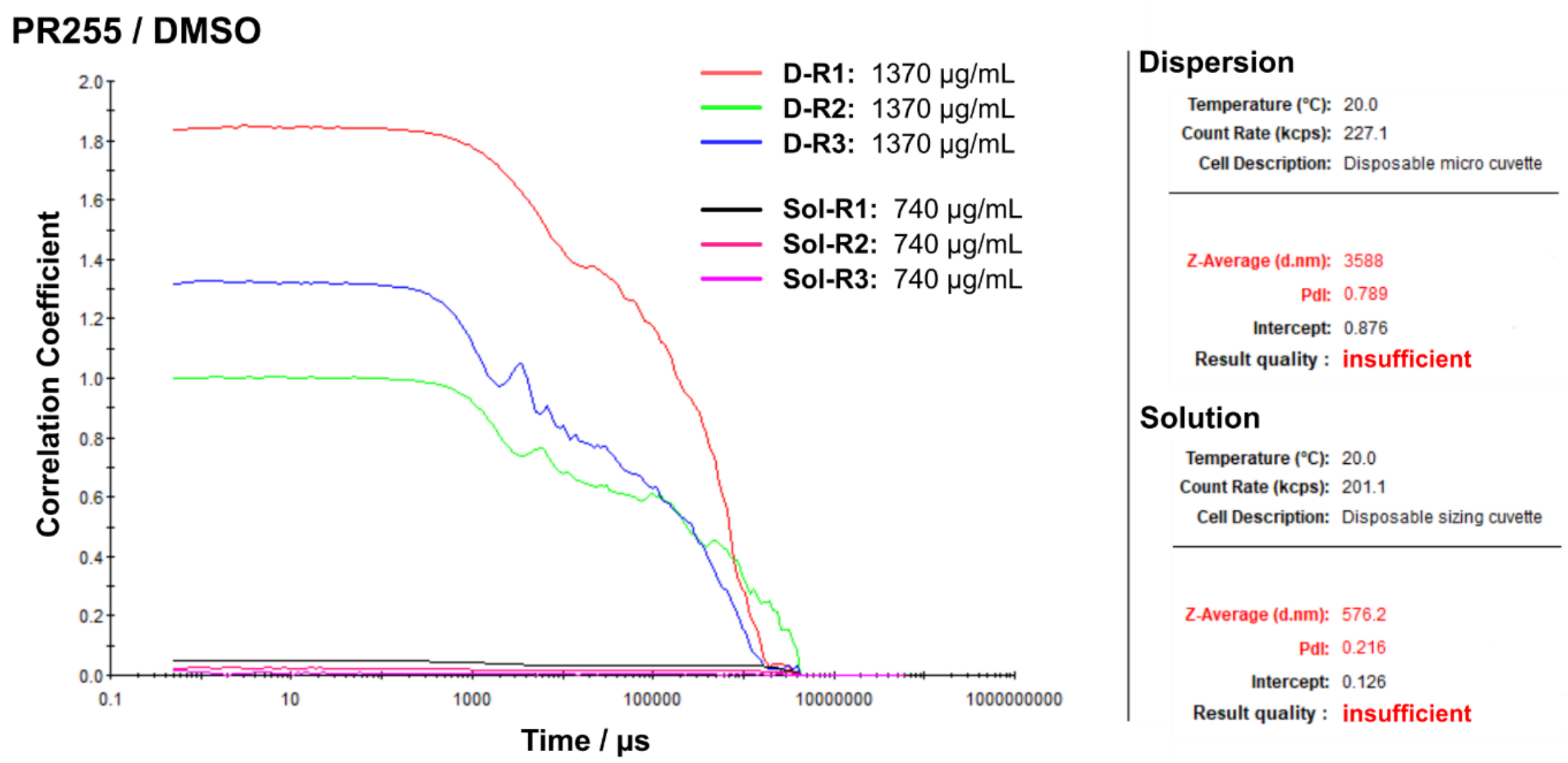


**Figure S112**. Autocorrelation functions from DLS measurements of PR255 in DMSO at concentrations 30% above (dispersion, D) and 30% below (solution, Sol) the solubility limit of 1050 µg/mL as determined by UV-vis spectroscopy. For all samples, particle size determination was not possible due to inconsistent autocorrelation functions, elevated polydispersity indices, or insufficient scattering intensity.

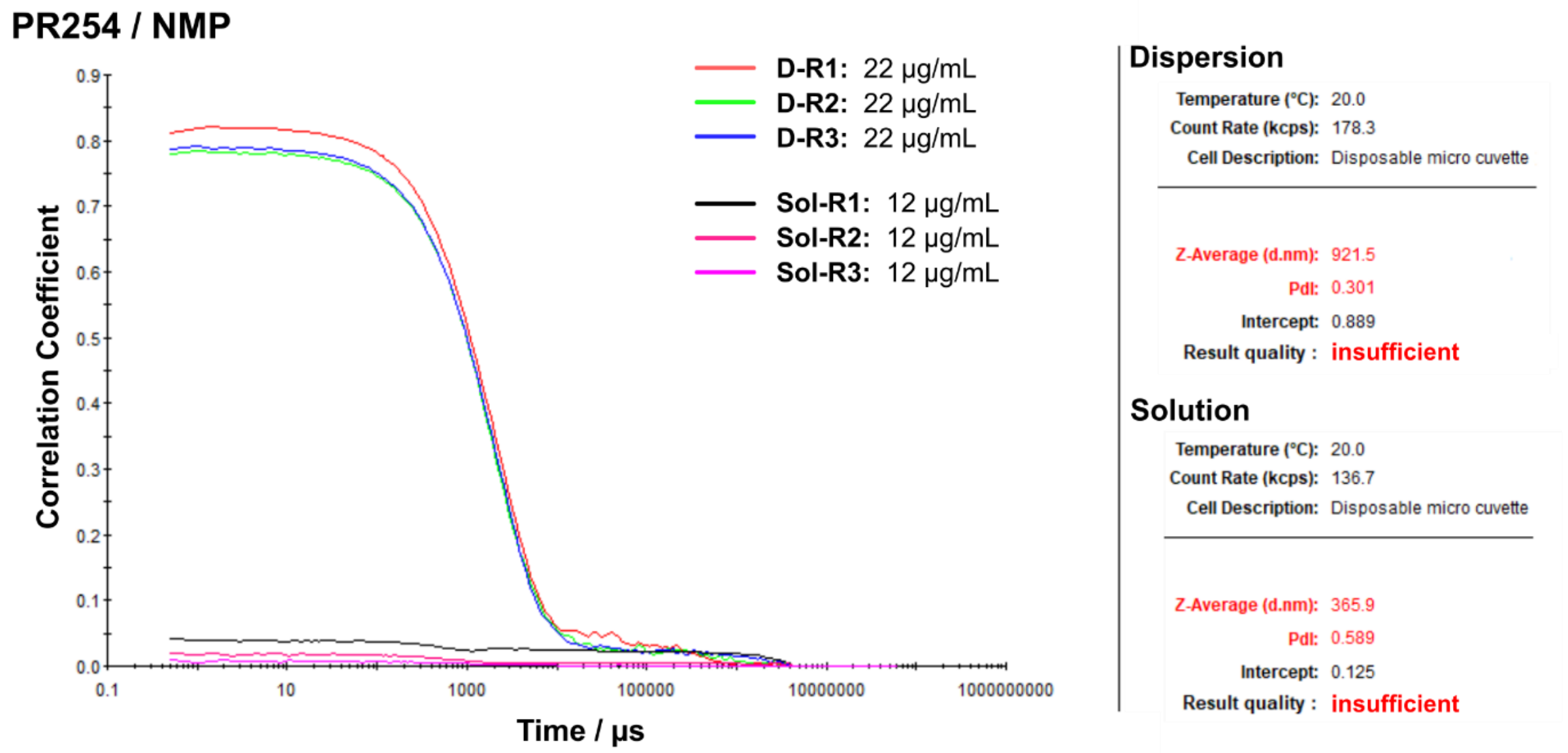


**Figure S113**. Autocorrelation functions from DLS measurements of PR254 in NMP at concentrations 30% above (dispersion, D) and 30% below (solution, Sol) the solubility limit of 17 µg/mL as determined by UV-vis spectroscopy. For all samples, particle size determination was not possible due to inconsistent autocorrelation functions, elevated polydispersity indices, or insufficient scattering intensity.

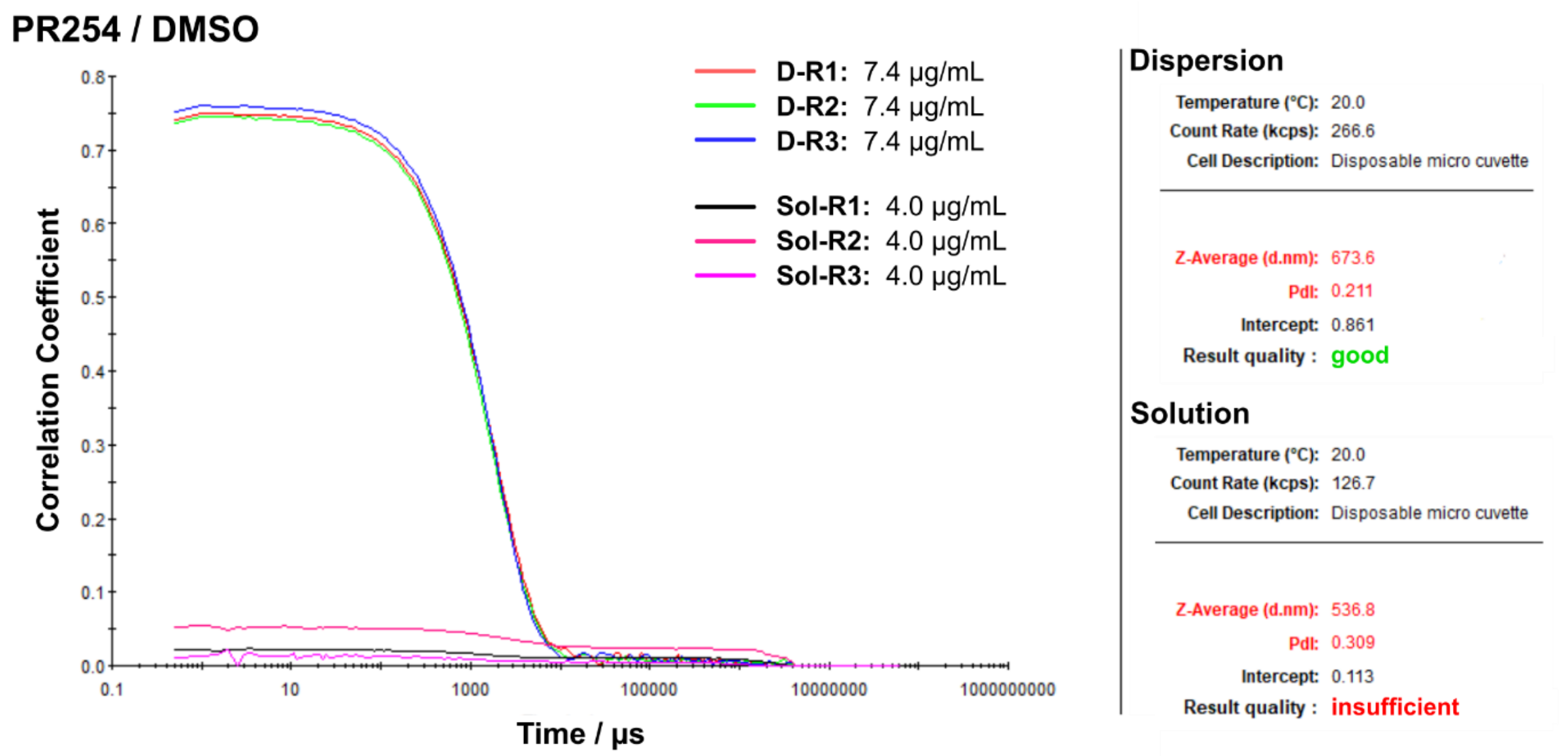


**Figure S114**. Autocorrelation functions from DLS measurements of PR254 in DMSO at concentrations 30% above (dispersion, D) and 30% below (solution, Sol) the solubility limit of 5.7 µg/mL as determined by UV-vis spectroscopy. For the solution sample, particle size determination was not possible due to inconsistent autocorrelation functions, elevated polydispersity indices, or insufficient scattering intensity. For the dispersion sample, the data quality was sufficient giving an average particle size of 673.6 nm with a PdI of 0.211.

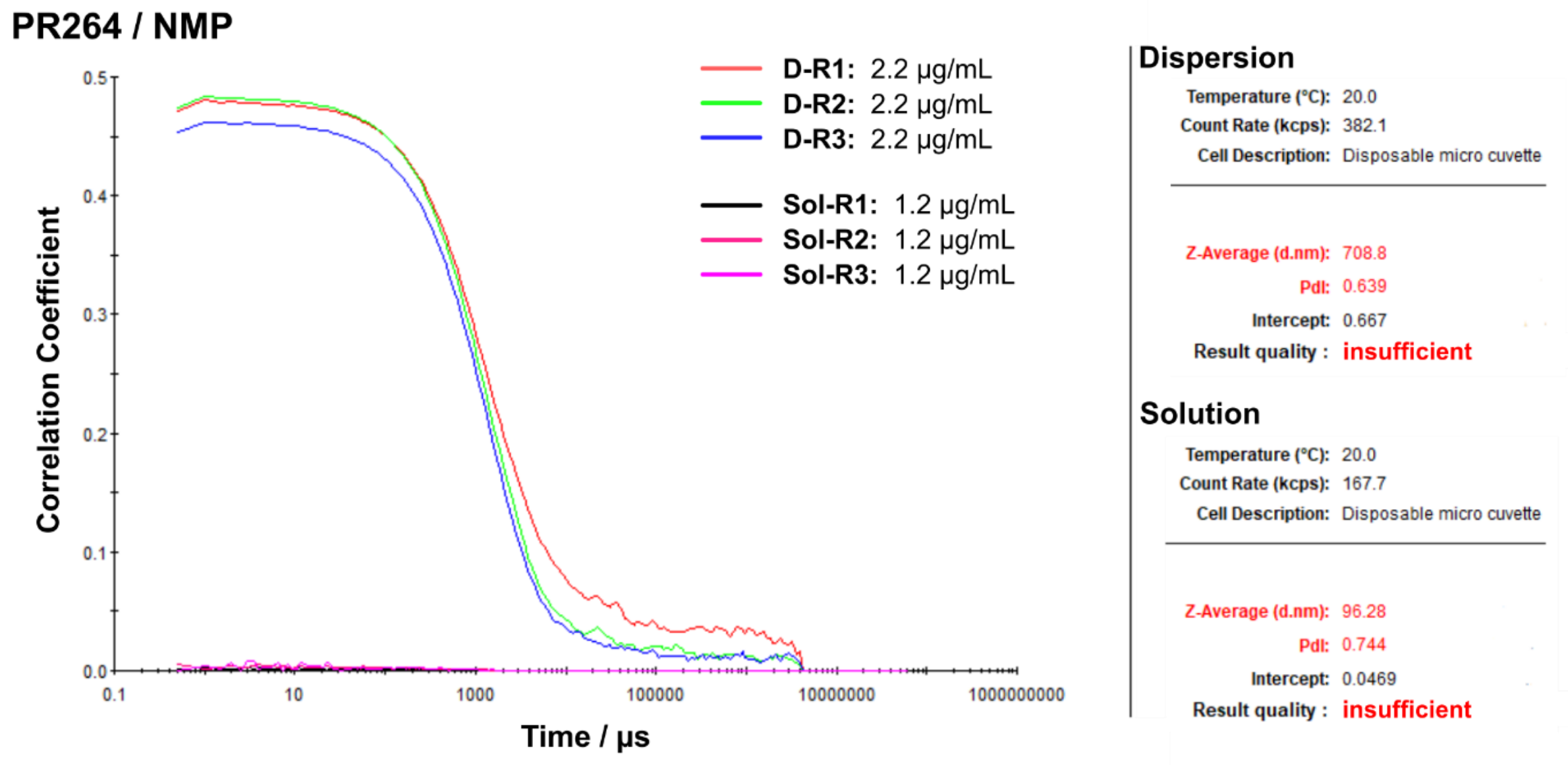


**Figure S115**. Autocorrelation functions from DLS measurements of PR264 in NMP at concentrations 30% above (dispersion, D) and 30% below (solution, Sol) the solubility limit of 1.7 µg/mL as determined by UV-vis spectroscopy. For all samples, particle size determination was not possible due to inconsistent autocorrelation functions, elevated polydispersity indices, or insufficient scattering intensity.

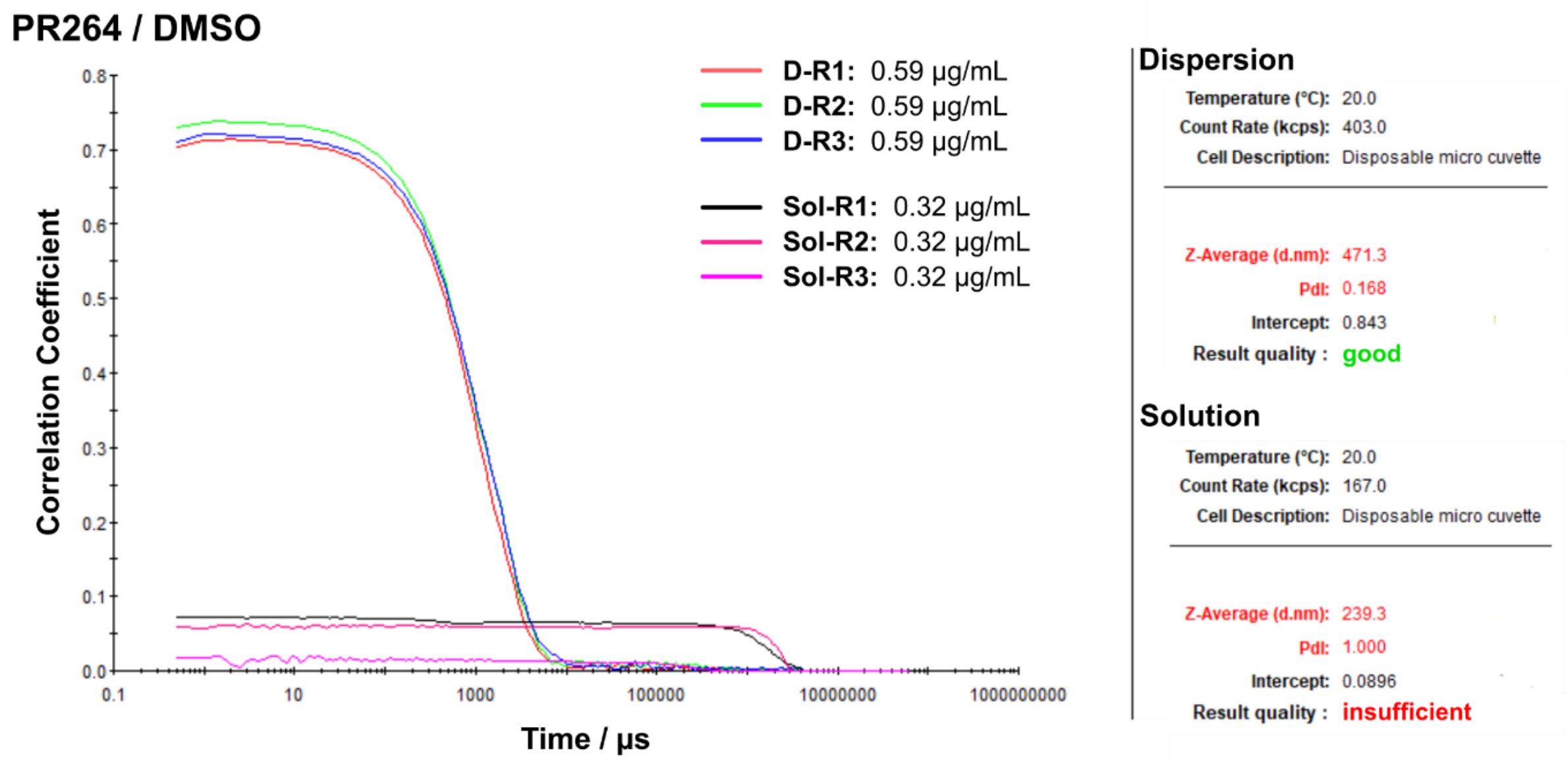


**Figure S116**. Autocorrelation functions from DLS measurements of PR264 in DMSO at concentrations 30% above (dispersion, D) and 30% below (solution, Sol) the solubility limit of 0.45 µg/mL as determined by UV-vis spectroscopy. For the solution sample, particle size determination was not possible due to inconsistent autocorrelation functions, elevated polydispersity indices, or insufficient scattering intensity. For the dispersion sample, the data quality was sufficient giving an average particle size of 471.3 nm with a PdI of 0.168.

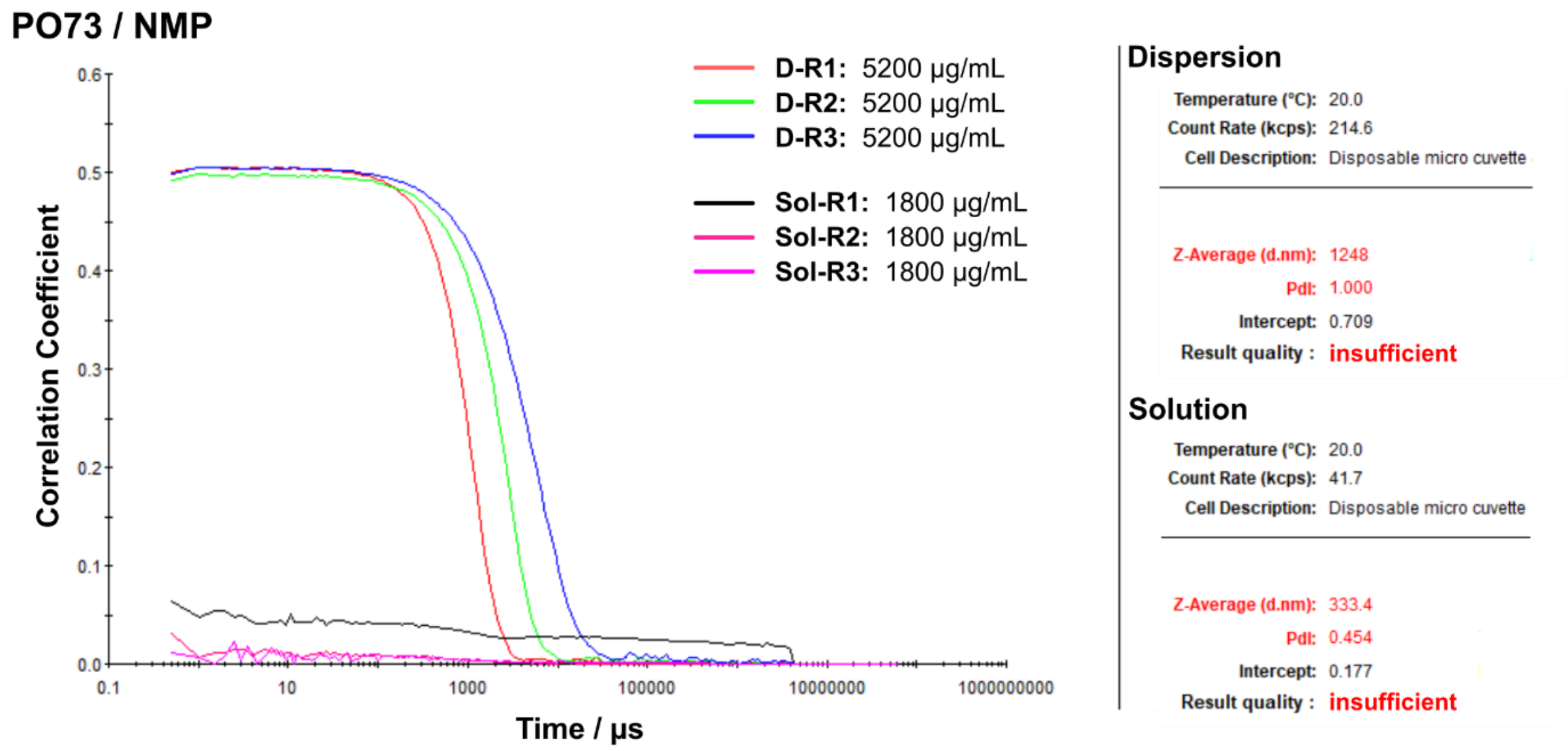


**Figure S117**. Autocorrelation functions from DLS measurements of PO73 in NMP at concentrations 30% above (dispersion, D) and 30% below (solution, Sol) the solubility limit of 4000 µg/mL as determined by UV-vis spectroscopy. For all samples, particle size determination was not possible due to inconsistent autocorrelation functions, elevated polydispersity indices, or insufficient scattering intensity.

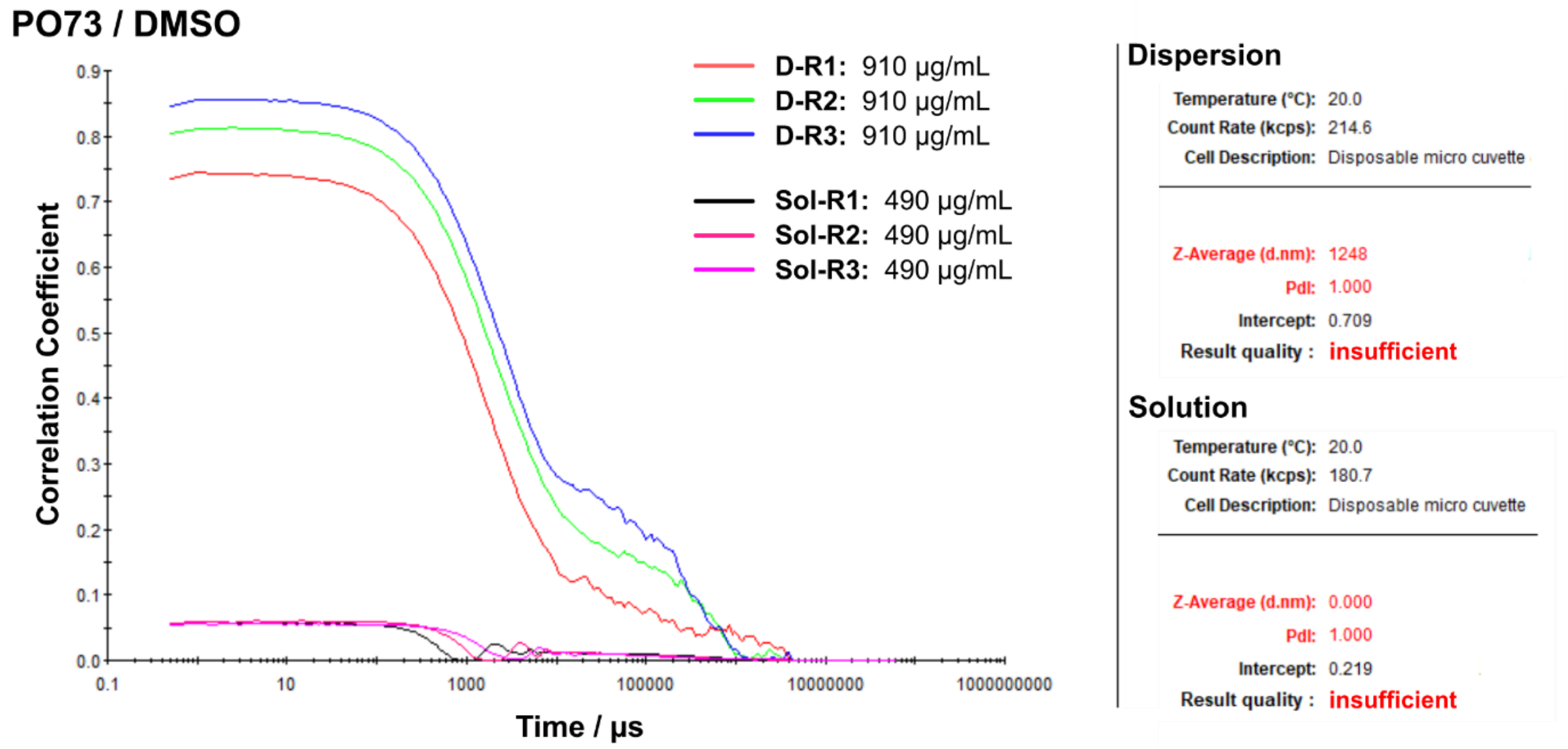


**Figure S118**. Autocorrelation functions from DLS measurements of PO73 in DMSO at concentrations 30% above (dispersion, D) and 30% below (solution, Sol) the solubility limit of 700 µg/mL as determined by UV-vis spectroscopy. For all samples, particle size determination was not possible due to inconsistent autocorrelation functions, elevated polydispersity indices, or insufficient scattering intensity.

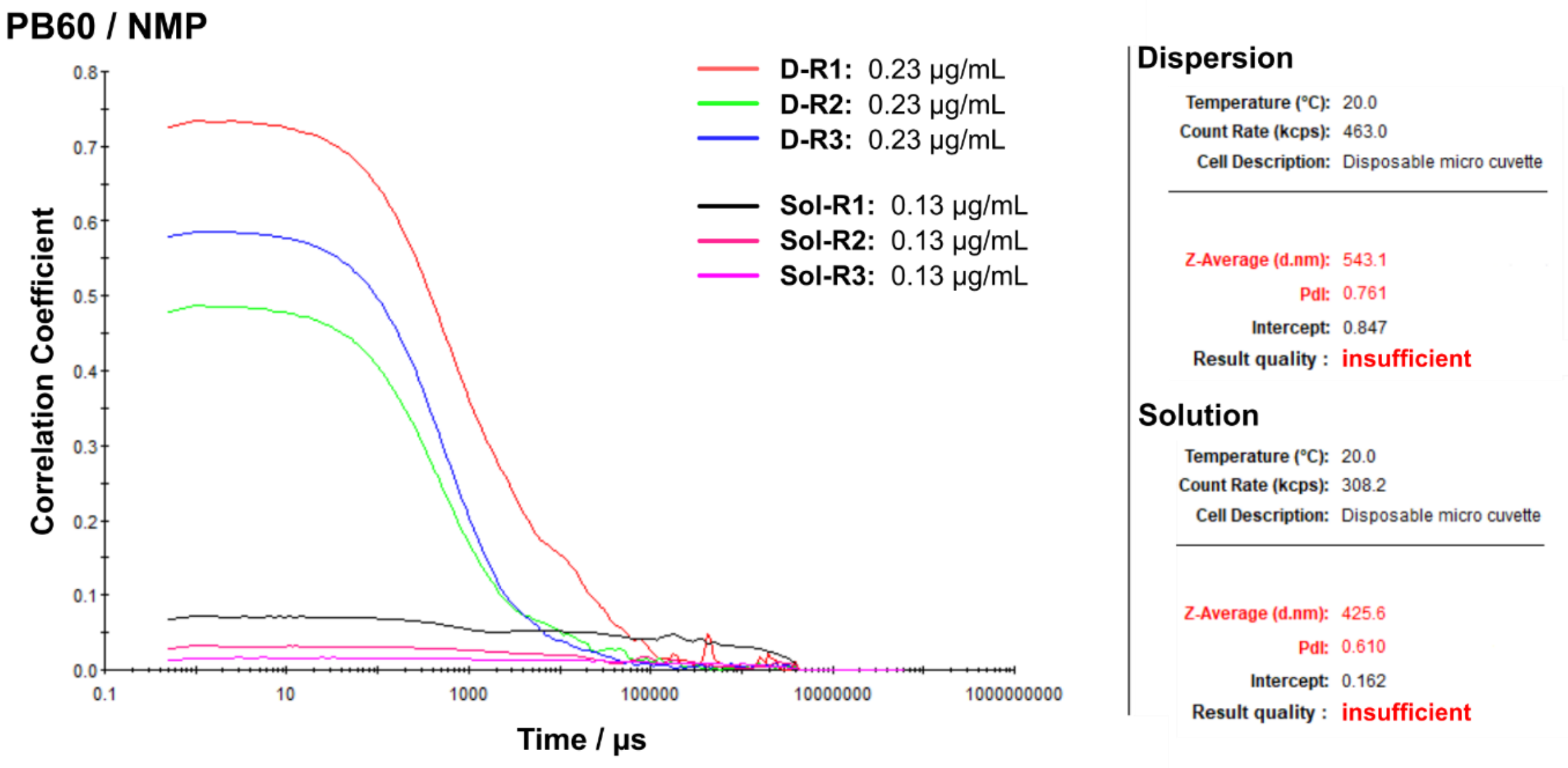


**Figure S119**. Autocorrelation functions from DLS measurements of PB60 in NMP at concentrations 30% above (dispersion, D) and 30% below (solution, Sol) the solubility limit of 0.18 µg/mL as determined by UV-vis spectroscopy. For all samples, particle size determination was not possible due to inconsistent autocorrelation functions, elevated polydispersity indices, or insufficient scattering intensity.

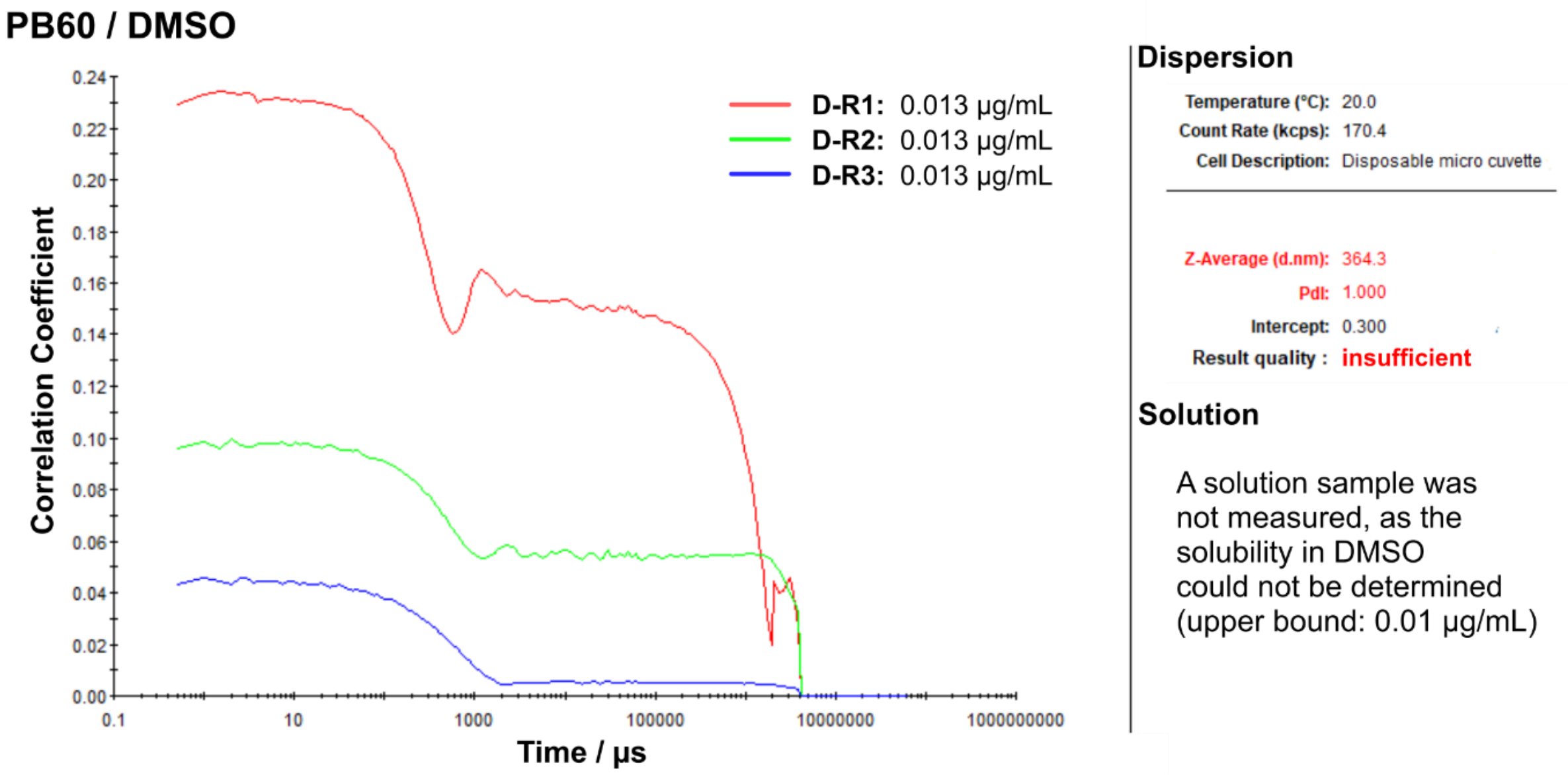


**Figure S120**. Autocorrelation functions from DLS measurements of PB60 in DMSO at concentrations 30% above the upper bound solubility limit of 0.01 µg/mL as determined by UV-vis spectroscopy. For all samples, particle size determination was not possible due to inconsistent autocorrelation functions, elevated polydispersity indices, or insufficient scattering intensity.

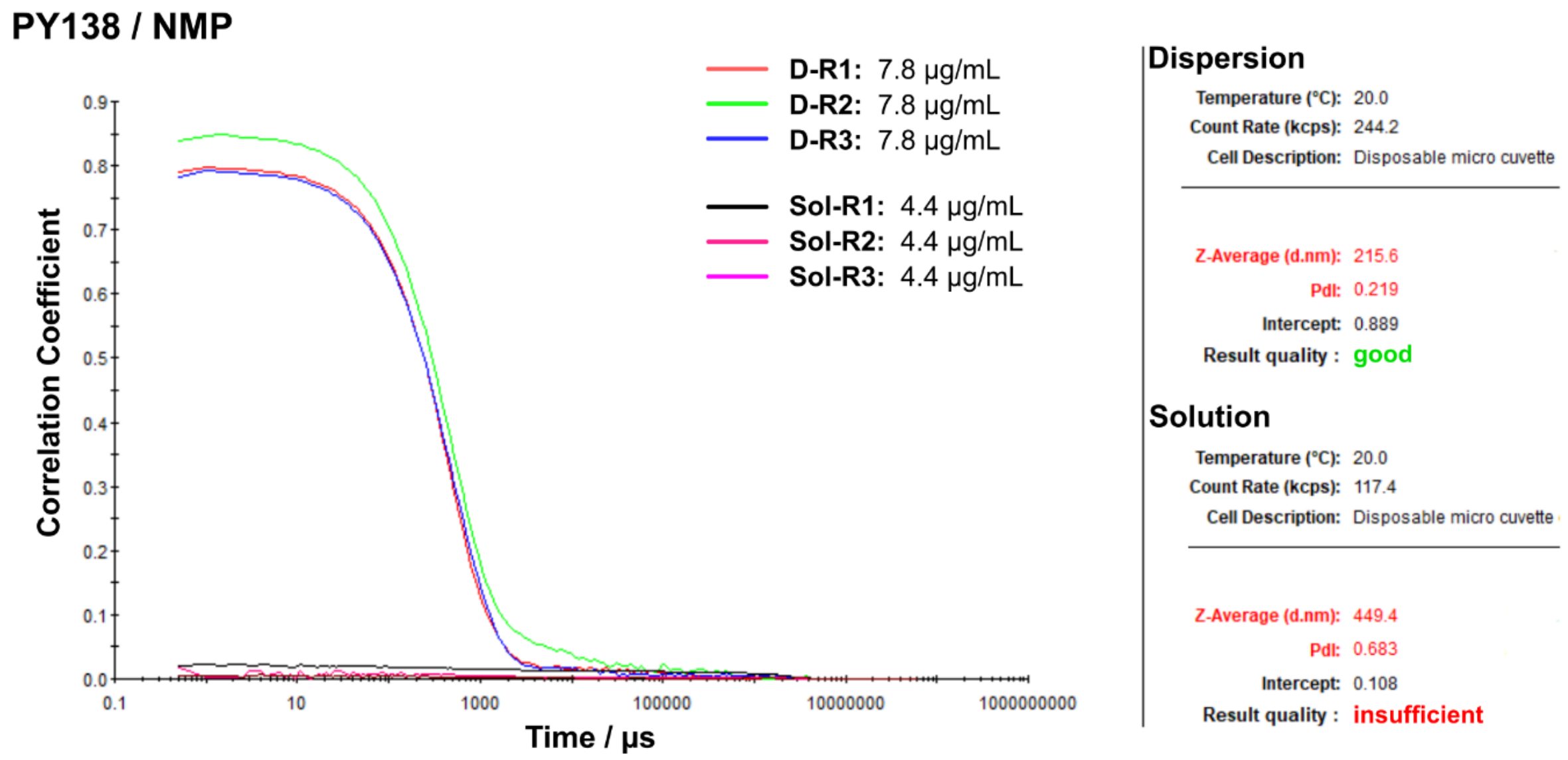


**Figure S121**. Autocorrelation functions from DLS measurements of PY138 in NMP at concentrations 30% above (dispersion, D) and 30% below (solution, Sol) the solubility limit of 6 µg/mL as determined by UV-vis spectroscopy. For the solution sample, particle size determination was not possible due to inconsistent autocorrelation functions, elevated polydispersity indices, or insufficient scattering intensity. For the dispersion sample, the data quality was sufficient giving an average particle size of 215.6 nm with a PdI of 0.219.

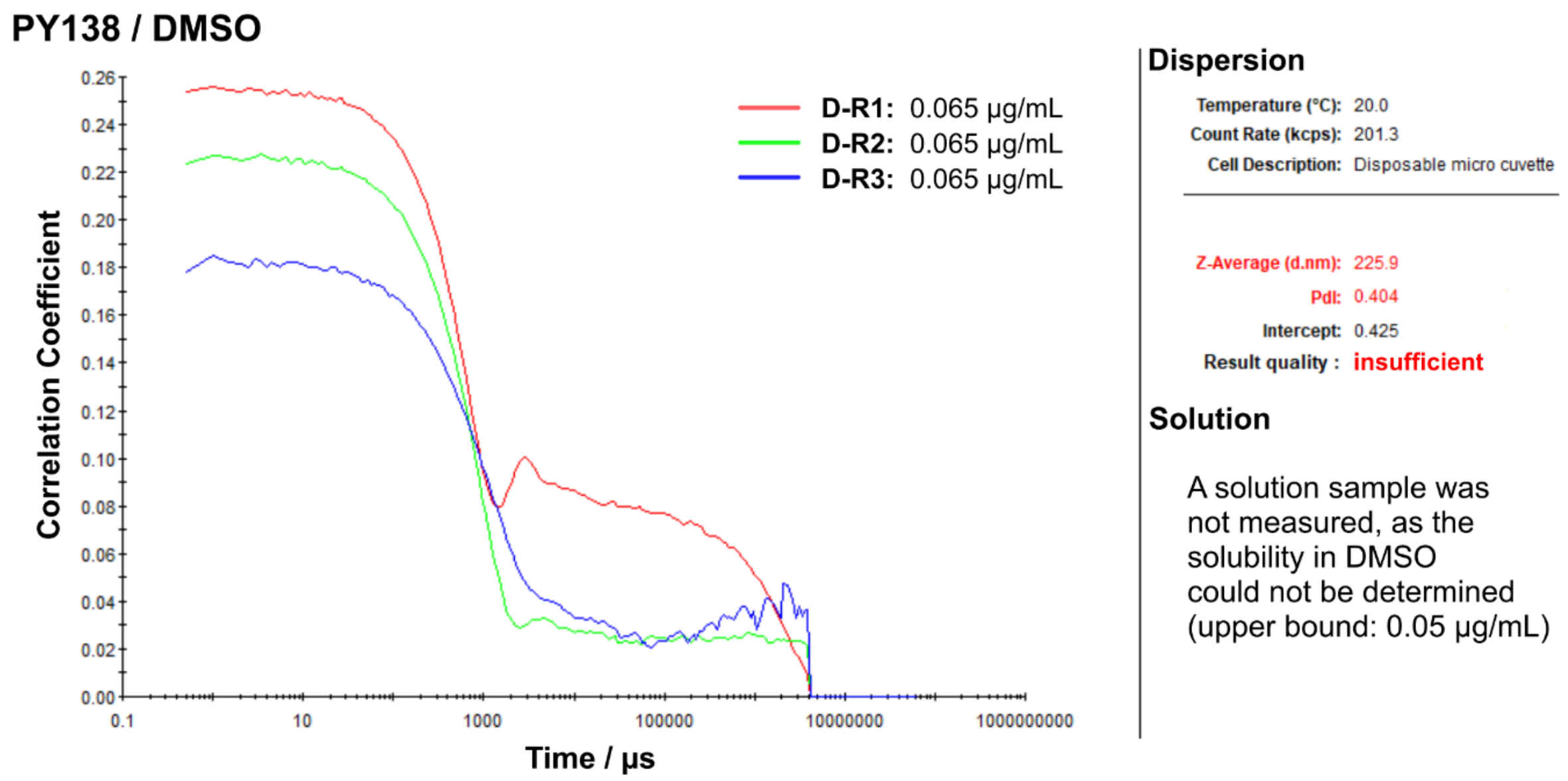


**Figure S122**. Autocorrelation functions from DLS measurements of PY138 in DMSO at concentrations 30% above the upper bound solubility limit of 0.05 µg/mL as determined by UV-vis spectroscopy. For all samples, particle size determination was not possible due to inconsistent autocorrelation functions, elevated polydispersity indices, or insufficient scattering intensity.

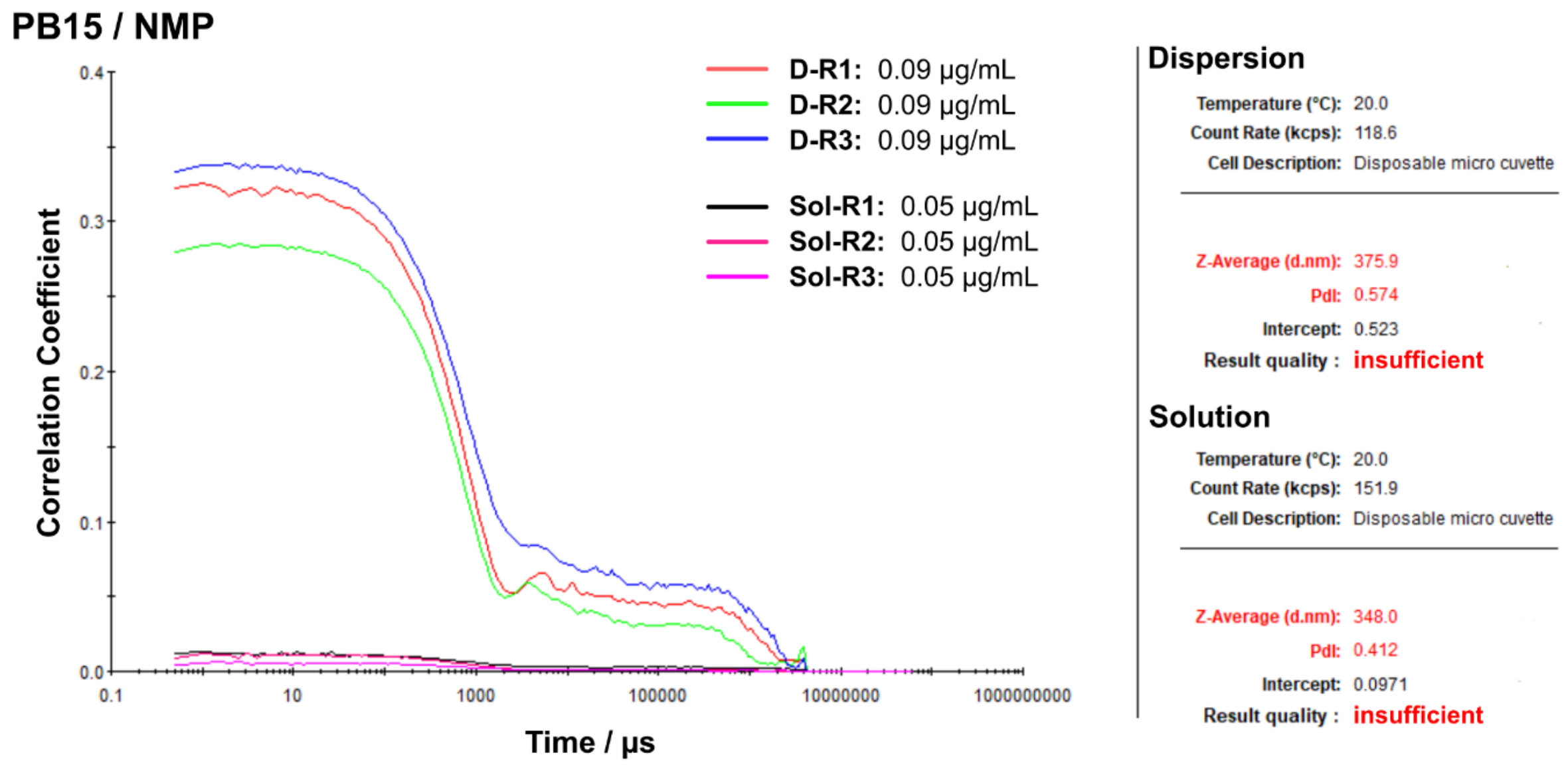


**Figure S123**. Autocorrelation functions from DLS measurements of PB15 in NMP at concentrations 30% above (dispersion, D) and 30% below (solution, Sol) the solubility limit of 0.07 µg/mL as determined by UV-vis spectroscopy. For all samples, particle size determination was not possible due to inconsistent autocorrelation functions, elevated polydispersity indices, or insufficient scattering intensity.

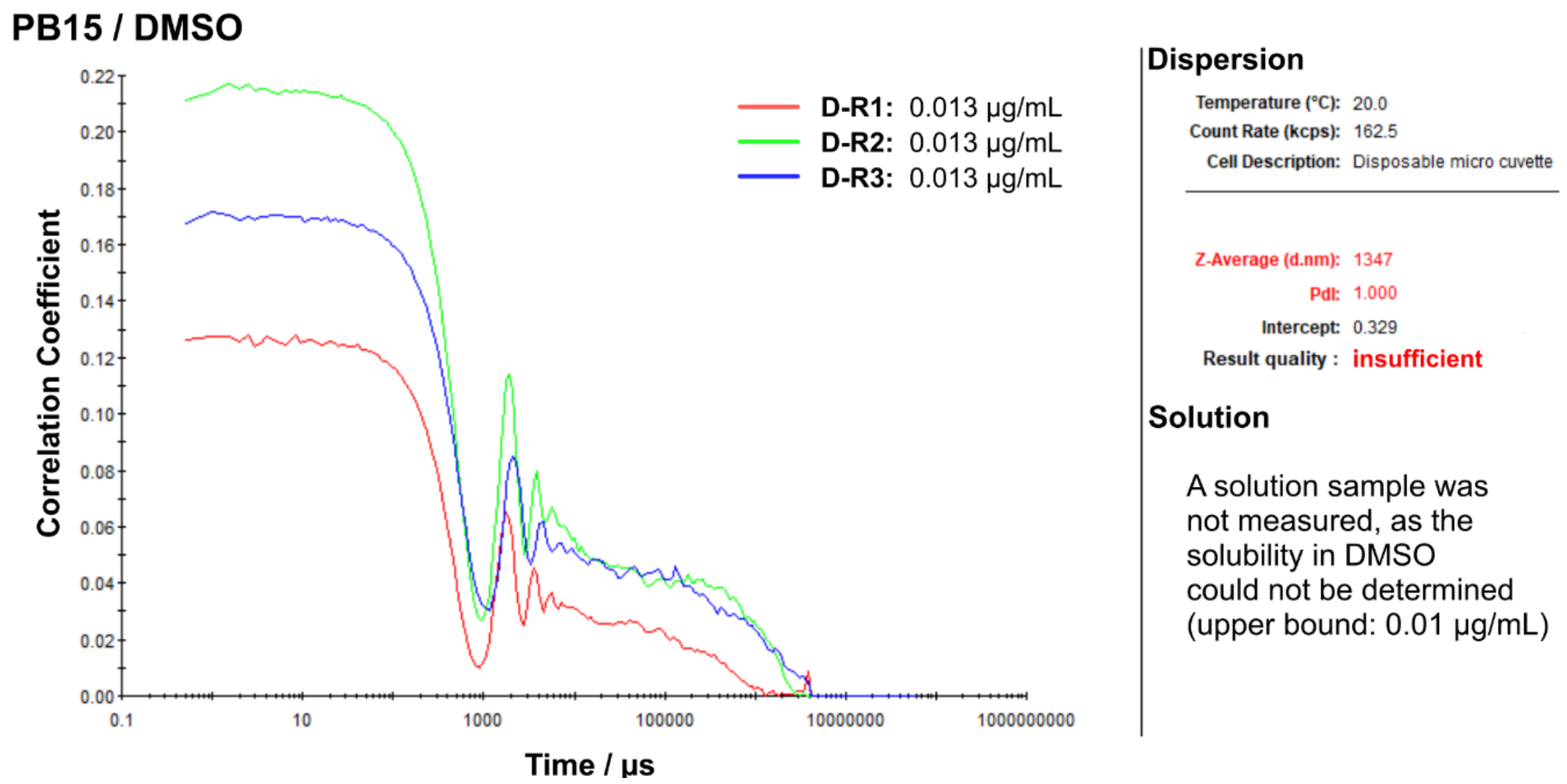


**Figure S124**. Autocorrelation functions from DLS measurements of PB15 in DMSO at concentrations 30% above the upper bound solubility limit of 0.01 µg/mL as determined by UV-vis spectroscopy. For all samples, particle size determination was not possible due to inconsistent autocorrelation functions, elevated polydispersity indices, or insufficient scattering intensity.

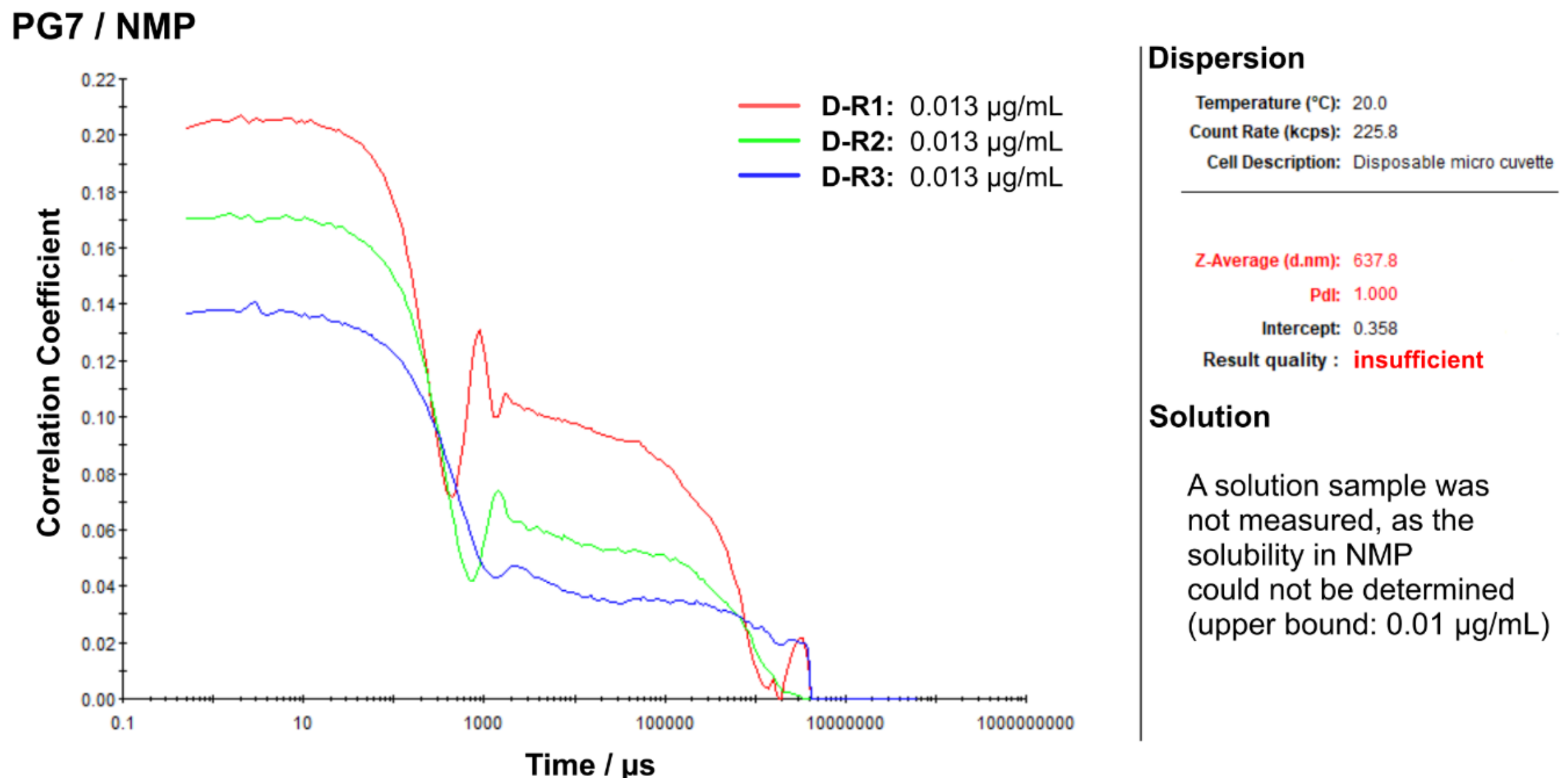


**Figure S125**. Autocorrelation functions from DLS measurements of PG7 in NMP at concentrations 30% above the upper bound solubility limit of 0.01 µg/mL as determined by UV-vis spectroscopy. For all samples, particle size determination was not possible due to inconsistent autocorrelation functions, elevated polydispersity indices, or insufficient scattering intensity.

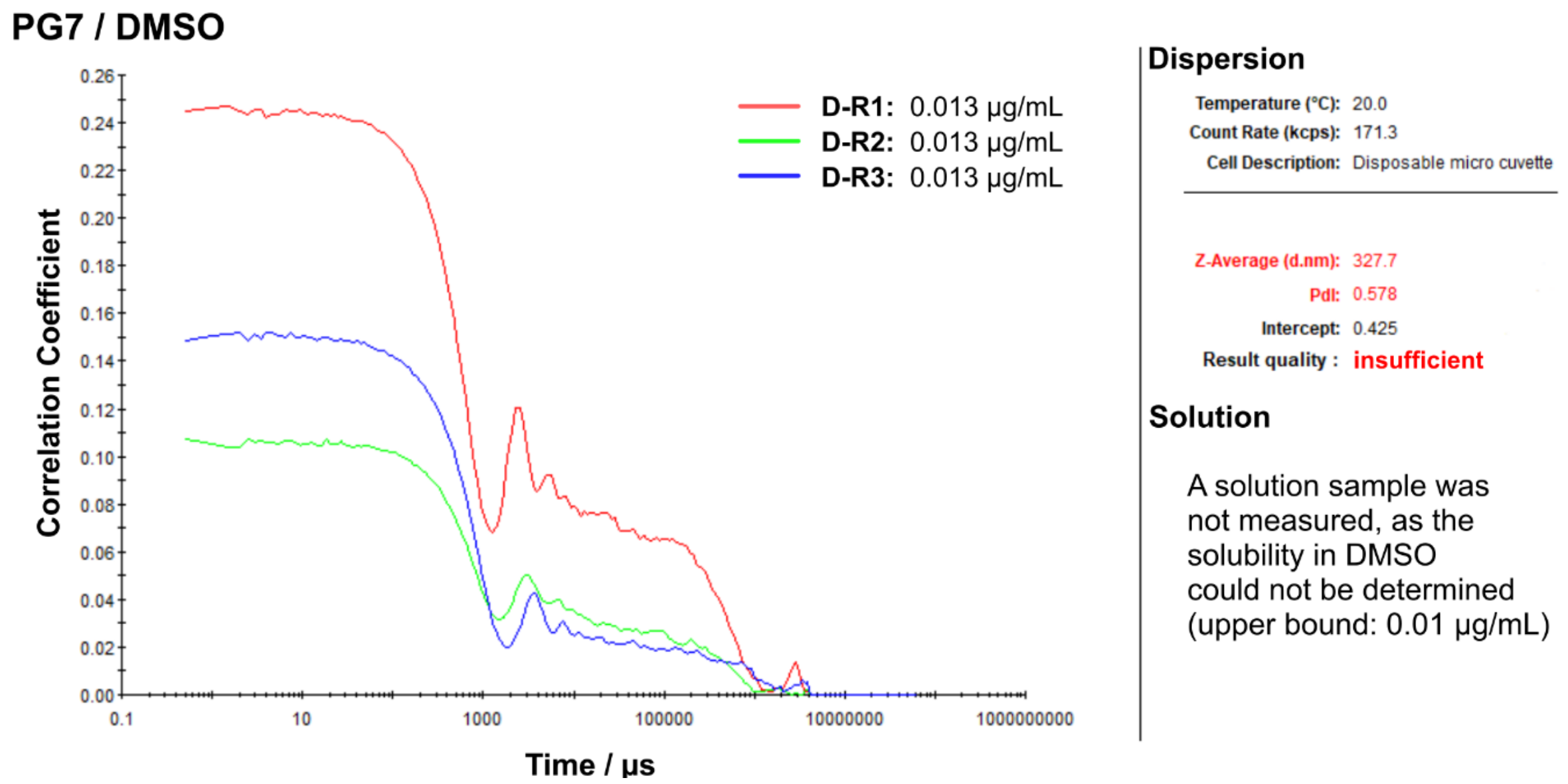


**Figure S126**. Autocorrelation functions from DLS measurements of PG7 in DMSO at concentrations 30% above the upper bound solubility limit of 0.01 µg/mL as determined by UV-vis spectroscopy. For all samples, particle size determination was not possible due to inconsistent autocorrelation functions, elevated polydispersity indices, or insufficient scattering intensity.

## 6. References


(1) Neese, F. Software Update: The ORCA Program System—Version 5.0. *WIREs Comput. Mol. Sci.* **2022**, *12*, e1606.

(2) Grimme, S.; Hansen, A.; Ehlert, S.; Mewes, J.-M. r²SCAN-3c: a “Swiss Army Knife” Composite Electronic-Structure Method. *J. Chem. Phys.* **2021**, *154*, 064103.

(3) (a) Adamo, C.; Barone, V. Toward Reliable Density Functional Methods Without Adjustable Parameters: The PBE0 Model. *J. Chem. Phys.* **1999**, *110*, 6158–6170; (b) Adamo, C.; Scuseria, G. E.; Barone, V. Accurate Excitation Energies from Time-Dependent Density Functional Theory: Assessing the PBE0 Model. *J. Chem. Phys.* **1999**, *111*, 2889–2899.

(4) Schäfer, A.; Horn, H.; Ahlrichs, R. Fully Optimized Contracted Gaussian Basis Sets for Atoms Li to Kr. *J. Chem. Phys.* **1992**, *97*, 2571–2577.

(5) Jacquemin, D.; Planchat, A; Adamo, C.; Mennucci, B. TD-DFT Assessment of Functionals for Optical 0–0 Transitions in Solvated Dyes. *J. Chem. Theory Comput.* **2012**, *8*, 2359–2372.

(6) Shao, Y.; Mei, Y.; Sundholm, D.; Kaila, V. R. I. Benchmarking the Performance of Time-Dependent Density Functional Theory for Calculating Electronic Circular Dichroism Spectra. *J. Chem. Theory Comput.* **2020**, *16*, 587–600.

(7) Laurent, A. D.; Jacquemin, D. TD-DFT benchmarks: A review. *Int. J. Quantum Chem.* **2013**, *113*, 2019–2039.

(8) Schmidt, M. U.; Hofmann, D. W. M.; Buchsbaum, C.; Metz, H. Crystal Engineering of Organic Pigments. *Angew. Chem. Int. Ed.* **2006**, *45*, 1313–1317.

(9) Chang, C.-H.; Christie, R. M.; Rosair, G. M. C.I. Pigment Red 266. *Acta Cryst.* **2003**, C*59*, 556–558.

(10) van de Streek, J.; Brüning, J.; Ivashevskaya, S. N.; Ermrich, M.; Paulus, E. F.; Bolte, M.; Schmidt, M. U. Structures of Six Industrial Benzimidazolone Pigments from Laboratory Powder Diffraction Data. *Acta Cryst.* **2009**, B*65*, 200–211.

(11) Gorelik, T. E.; Beko, S. L.; Teteruk, J.; Heyse, W.; Schmidt, M. U. Analysis of Diffuse Scattering in Electron Diffraction Data for the Crystal Structure Determination of Pigment Orange 13, $C_{32}H_{24}Cl_2N_8O_2$. *Acta Cryst.* **2023**, B*79*, 122–137.

(12) Schmidt, M. U.; Dinnebier, R. E.; Kalkhof, H. Crystal Engineering on Industrial Diaryl Pigments Using Lattice Energy Minimizations and X-ray Powder Diffraction. *J. Phys. Chem. B* **2007**, *111*, 9722–9732.

(13) Paulus, E. F.; Leusen, F. J. J.; Schmidt, M. U. Crystal Structures of Quinacridones *CrystEngComm* **2007**, *9*, 131–143.

(14) Mizuguchi, J.; Senju, T.; Sakai, M. Crystal structure of 5,12-dihydro-2,9-dimethylquino[2,3-b]acridine-7,14-dione, $C_{22}H_{16}N_2O_2$, at 123 K. *Z. Kristallogr. – New Cryst. Struct.* **2002**, *217*, 525–526.

(15) Senju, T.; Hoki, T.; Mizuguchi, J. 2,9-Di-chloro-5,12-di-hydro-quino-[2,3-b]-acridine-7,14-dione. *Acta Cryst.* **2005**, E*61*, 1061–1063.

(16) Senju, T.; Nishimura, N.; Hoki, T.; Mizuguchi, J. 2,9-Di-chloro-5,12-di-hydro-quino-[2,3-b]-acridine-7,14-dione (Red Phase) *Acta Cryst.* **2005**, E*61*, 2596–2598.

(17) Mizuguchi, J.; Grubenmann, A.; Wooden, G.; Rihs, G. Structures of 3,6-diphenylpyrrolo[3,4-c]pyrrole-1,4-dione and 2,5-dimethyl-3,6-diphenylpyrrolo[3,4-c]pyrrole-1,4-dione *Acta Cryst.* **1992**, B*48*, 696–700.

(18) Mizuguchi, J.; Grubenmann, A.; Rihs, G. Structures of 3,6-bis(3-chlorophenyl)pyrrolo[3,4-c]pyrrole-1,4-dione and 3,6-bis(4-chlorophenyl)pyrrolo[3,4-c]pyrrole-1,4-dione *Acta Cryst.* **1993**, B*49*, 1056–1060.

(19) Mizuguchi, J.; Miyazaki, T. Crystal structure of 3,6-bis(4-biphenylyl)pyrrolo[3,4-c]pyrrole-1,4-dione, $C_{30}H_{20}N_2O_2$. J. *Z. Kristallogr. – New Cryst. Struct.* **2002**, *217*, 43-44.
(20) Mizuguchi, J. 3,6-Bis(4-tert-butylphenyl)-1,2,4,5-tetrahydropyrrolo[3,4-c]pyrrole-1,4-dione. *Acta Cryst.* **1998,** *C54*, 1482–1484.
(21) Gumbert, S. D.; Körbitzer, M.; Alig, E.; Schmidt, M. U.; Chierotti, M. R.; Gobetto, R.; Li, X.; van de Streek, J. Crystal Structure and Tautomerism of Pigment Yellow 138 Determined by X-ray Powder Diffraction and Solid-State NMR. *Dyes Pigments* **2016**, *131*, 364–372.
(22) Bailey, M. The Crystal Structure of Indanthrone. *Acta Cryst.* **1955,** *8*, 182–185.
(23) Hoshino, A.; Takenaka, Y.; Miyaji, H. Redetermination of the Crystal Structure of α-Copper Phthalocyanine Grown on KCl. *Acta Cryst.* **2003**, B*59*, 393–403.
(24) Brown, C. J. Crystal Structure of β-Copper Phthalocyanine. *J. Chem. Soc. A* **1968**, 2488–2493.
(25) https://rmg.mit.edu/database/solvation/soluteSearch/
(26) Sinha, S.; Yang, C.; Wu, E.; Acree Jr., W. E. Abraham Solvation Parameter Model: Examination of Possible Intramolecular Hydrogen-Bonding Using Calculated Solute Descriptors. *Liquids* **2022**, *2*, 131-146.
(27) Endo, S.; Goss, K.-U. Serum Albumin Binding of Structurally Diverse Neutral Organic Compounds: Data and Models. *Chem. Res. Toxicol.* **2011**, *24*, 2293–2301.
(28) Endo, S.; Brown, T. N.; Goss, K.-U. General Model for Estimating Partition Coefficients to Organisms and Their Tissues Using the Biological Compositions and Polyparameter Linear Free Energy Relationships. *Environ. Sci. Technol.* **2013**, *47*, 6630–6639.
(29) Endo, S.; Goss, K.-U. Applications of Polyparameter Linear Free Energy Relationships in Environmental Chemistry *Environ. Sci. Technol.* **2014**, *48*, 12477–12491.
(30) (a) Chung, Y.; Vermeire, F. H.; Wu, H.; Walker, P. J.; Abraham, M. H.; Green, W. H. Group Contribution and Machine Learning Approaches to Predict Abraham Solute Parameters, Solvation Free Energy, and Solvation Enthalpy. *J. Chem. Inf. Model.* **2022**, *62*, 433–446.
(b) https://rmg.mit.edu/database/solvation/searchML/
